# Predicting RNA 5-Methylcytosine Sites by Using Essential Sequence Features and Distributions

**DOI:** 10.1155/2022/4035462

**Published:** 2022-01-13

**Authors:** Lei Chen, ZhanDong Li, ShiQi Zhang, Yu-Hang Zhang, Tao Huang, Yu-Dong Cai

**Affiliations:** ^1^School of Life Sciences, Shanghai University, Shanghai 200444, China; ^2^College of Information Engineering, Shanghai Maritime University, Shanghai 201306, China; ^3^College of Food Engineering, Jilin Engineering Normal University, Changchun, China; ^4^Department of Biostatistics, University of Copenhagen, Copenhagen 2099, Denmark; ^5^Channing Division of Network Medicine, Brigham and Women's Hospital, Harvard Medical School, Boston, MA, USA; ^6^Bio-Med Big Data Center, CAS Key Laboratory of Computational Biology, Shanghai Institute of Nutrition and Health, University of Chinese Academy of Sciences, Chinese Academy of Sciences, Shanghai 200031, China; ^7^CAS Key Laboratory of Tissue Microenvironment and Tumor, Shanghai Institute of Nutrition and Health, University of Chinese Academy of Sciences, Chinese Academy of Sciences, Shanghai 200031, China

## Abstract

Methylation is one of the most common and considerable modifications in biological systems mediated by multiple enzymes. Recent studies have shown that methylation has been widely identified in different RNA molecules. RNA methylation modifications have various kinds, such as 5-methylcytosine (m^5^C). However, for individual methylation sites, their functions still remain to be elucidated. Testing of all methylation sites relies heavily on high-throughput sequencing technology, which is expensive and labor consuming. Thus, computational prediction approaches could serve as a substitute. In this study, multiple machine learning models were used to predict possible RNA m^5^C sites on the basis of mRNA sequences in human and mouse. Each site was represented by several features derived from *k*-mers of an RNA subsequence containing such site as center. The powerful max-relevance and min-redundancy (mRMR) feature selection method was employed to analyse these features. The outcome feature list was fed into incremental feature selection method, incorporating four classification algorithms, to build efficient models. Furthermore, the sites related to features used in the models were also investigated.

## 1. Introduction

Methylation is one of the most common and considerable modifications in biological systems mediated by multiple enzymes. The substrates of biological methylation are diverse, with DNA as the most common one. Previous studies on methylation mostly focused on DNA methylation, revealing its specific role in transcriptional activity regulation during development, aging, and pathogenesis [1]. However, recent studies have widely identified methylation among different RNA molecules, including mRNA, snoRNA, miRNA, and rRNA (not restricted to functional mRNAs) [2]. RNA methylation enables the posttranscriptional control of gene expression by changing how RNA interacts with other components of the cell as an important part of epitranscriptome [3]. RNA methylation is actively involved in posttranscriptional regulatory bioprocesses, like RNA splicing, transport, stability, and translatability, and it has strong relationships with mammalian development and diseases [4–6].

Among the various kinds of RNA methylation modifications, N^6^-methyladenosine (m^6^A), the methylation modification on the nitrogen at the sixth position of the adenosine base, is the most prevalent internal mRNA modification, accounting for 50% of the total methylated ribonucleotides [2, 7]. M^6^A broadly affects mRNA metabolism, and it is widely distributed in all kinds of RNA transcripts, including coding and noncoding regions. The deposition of m^6^A modification in the transcriptome has its unique pattern: the m^6^A modification sites have a typical consensus sequence DRACH (D = G, A, or U; R = G or A; H = A, C, or U), which is widely dispersed over coding sequence and untranslated region (UTR) and highly enriched near the stop codon area [8]. Recent evidence has proven that m^6^A RNA methylation plays a vital role in pre-mRNA splicing, mRNA stability regulation, mRNA export, mRNA degradation, translation regulation, and miRNA processing [9–11]. M^6^A modification is dynamic, it could be reversible, and it may vary between different genes and different tissues [12, 13]. With the increase in the number of m^6^A mapping studies, the list of specific genes containing a disproportionately high level of m^6^A was revealed. For example, Han et al. found a series of m^6^A methylated genes related to the presynaptic membrane, the postsynaptic membrane, and the synaptic growth in Alzheimer's disease (AD) mouse models, suggesting that m^6^A may be involved in the occurrence of AD [14]. While the function of m^6^A modification is context-dependent and dynamic, many m^6^A sites are evolutionally conserved among species. One-third of mammalian mRNAs share the same m^6^A modifications, and many of them are conserved with single-nucleotide specificity [15].

Another kind of RNA methylation modification, namely, 5-methylcytosine (m^5^C), which is the methylation of carbon 5 in cytosine, also acts as an important regulator in gene expression, including RNA localization, ribosome assembly, translation regulation, and mRNA stabilization. Among all the mRNA methylation sites, the proportion of m^5^C could be up to 20% in human cells [16]. The distribution of m^5^C sites in mRNA is not random; in HeLa and mouse cells, m^5^C methylation were found to be enriched in 5′ and 3′ UTRs rather than coding regions [16]. Like m^6^A, m^5^C acts its function in dynamic ways. M^5^C methylation occurs dynamically during testis development and helps maintain the stability of maternal mRNA in embryonic development [17].

Though RNA methylation plays a pivotal role in bioprocess and is of great importance to posttranscriptional regulation, their functions in individual methylation sites still remain to be elucidated. Testing of all the methylation sites relies heavily on high-throughput sequencing technology, which is expensive and labor consuming; thus, computational prediction approaches could serve as a substitute [18]. As mentioned above, the distribution of m^5^C in mRNA has its own enrichment pattern and is not random. With adequate datasets and statistic method, predicting accurate m^5^C RNA methylation sites and gaining an enhanced understanding of their functions are doable.

In this study, multiple machine learning models were applied to predict the possible m^5^C RNA methylation sites in mRNA sequences of human and mouse. For each m^5^C, a subsequence containing such site as center was extracted from the RNA sequence. The features of *k*-mers yielded by RNA2Vec [19] were refined to represent the subsequence. The powerful max-relevance and min-redundancy (mRMR) feature selection method [20] was employed to analyse all features. Obtained feature list was fed into incremental feature selection (IFS) [21] method, incorporating four classification algorithms, to build efficient models. In addition to prediction models, we also investigated the sites related to features used in the models, trying to discover special patterns around mouse and human m^5^C sites. Comparison of those prediction results may help obtain a dynamic RNA methylation profile and build relationships between the RNA methylation sites and human diseases.

## 2. Materials and Methods

### 2.1. Data

M^5^C is a common RNA modification in mammals. Human and mouse m^5^C data were downloaded from one previous study (iRNA-m^5^C, http://lin-group.cn/server/iRNA-m5C/download.html) [22]. In fact, the human m^5^C data was first used in [23], which was extracted from the original data retrieved from RMBase database [24]. The original data was processed by CD-HIT program [25] so that the sequence similarity of any remaining sequences was less than 0.7. As a result, 120 positive and 120 negative m^5^C sites were obtained. As for mouse m^5^C data, it was constructed in [22]. It was directly retrieved from RMBase database [24] and was not processed by CD-HIT program [25] because its size was so small. The mouse data consisted of 97 positive and 97 negative m^5^C sites. As the sites around the m^5^C sites have some special patterns, which can help to identify m^5^C sites in RNA sequence, 20 upstream sites and 20 downstream sites were picked up. These sites together with the m^5^C site at the center constructed a subsequence with 41 bp. Some features would be extracted from this subsequence to represent the m^5^C site.

### 2.2. Problem Description and Study Design

For a given RNA sequence, it is essential to identify m^5^C sites in it. The machine learning models can give a deep investigation on current known m^5^C sites and learn a special pattern to make prediction. The prediction procedure can be deemed as a function *f*, formulated by

(1) $$\begin{array}{c} f:\Psi ⟶\left\{+,-\right\}, \end{array}$$

where Ψ denoted the site set for human or mouse RNA sequences and +(−) represented whether the input site was an m^5^C site or not.

Generally, we want to discover an optimal function such that its loss was smallest. Because machine learning algorithms were employed to design such function, we adopted the following steps:

1. For any site in the human or mouse m^5^C data, sites around it were picked up to comprise a subsequence, which can indicate the surrounding information of the investigated site. This step was described in section “Feature Engineering”
2. Each subsequence was represented by a number of features, which can reflect its essential information. This step was described in section “Feature Engineering”
3. A feature selection method was adopted to analyse all features and produce a feature list. This step was described in section “Max-Relevance and Min-Redundancy (mRMR) Feature Selection”
4. The IFS method was applied on such feature list to find out which classification algorithm and which features can yield the best performance (smallest loss). This step was described in section “Incremental Feature Selection (IFS).” The descriptions of four classification algorithms used in IFS method can be found in section “Classification Algorithm.” The loss was determined by one measurement listed in section “Performance Measurement”

### 2.3. Feature Engineering

To build efficient models for identifying m^5^C site in RNA sequence, it is very important to extract essential features from the subsequence consisting of this site, 20 upstream sites and 20 downstream sites. This study adopted a natural language processing approach to extract features, which were further used to represent the subsequence containing m^5^C site.

RNA2Vec [19] was adopted to extract sequence features for each *k*-mers (subsequences of length *k*). In detail, this method employed the whole human genome as corpus. A sliding window technique was used to split the RNA sequence into several fix-length words. If an RNA sequence with length *L* was formulated by

(2) $$\begin{array}{c} S=R_{1}R_{2}\cdots R_{i}\cdots R_{L-1}R_{L}, \end{array}$$

it was split into *L* − *k* + 1 words, say *R*_1_*R*_2_ ⋯ *R*_*k*_, *R*_2_*R*_3_ ⋯ *R*_*k*+1_, ⋯, *R*_*L*−*k*+1_*R*_*L*−*k*+2_ ⋯ *R*_*L*_. All obtained words were fed into GloVe algorithm [26], a type of Word2vec method, to extract features of words, i.e., features of *k*-mers. Here, we selected *k* = 4. Features of 4-mers were directly retrieved from https://github.com/HsiaoYetGun/MiRLocator/blob/master/RNA2Vec/RNAVectors.txt. Each 4-mers was represented by 30 features.

Given a 41 bp long RNA subsequence *SS*, formulated by

(3) $$\begin{array}{c} SS=R_{1}R_{2}\cdots R_{20}R_{21}R_{22}\cdots R_{40}R_{41}, \end{array}$$

where *R*_21_ was the m^5^C site, we extracted all 4-mers from this subsequence. Because the *R*_21_ was always same for all investigated subsequences, the 4-mers containing this site were discarded. 34 4-mers can be obtained from each RNA subsequence. Their 30 features obtained by RNA2Vec were collected together to represent the subsequence *SS*. Accordingly, 1020 (34 × 30) features were adopted to encode each subsequence with 41 bp.

### 2.4. Max-Relevance and Min-Redundancy (mRMR) Feature Selection

The mRMR is a powerful feature selection method [20, 27–30], which evaluates the importance of features from two aspects: (1) relevance to class labels and (2) redundancies to other features. The mutual information (MI) is used to quantify the relevance and redundancy. For two variables *x* and *y*, their MI is computed by

(4) $$\begin{array}{c} MI\left(x,y\right)=\iint p\left(x,y\right)\log\frac{p\left(x,y\right)}{p\left(x\right)p\left(y\right)}dxdy, \end{array}$$

where *p*(*x*) and *p*(*y*) stand for the marginal probabilistic densities of *x* and *y*, respectively, and *p*(*x*, *y*) stands for the joint probabilistic density of *x* and *y*. Generally, a high MI indicates the strong relevance or high redundancy of two variables. The mRMR method tries to keep features with high relevance to class labels and low redundancies to other features. However, this is a NP-hard problem. The mRMR method employed a heuristic way to evaluate features, which sorts all investigated features in a list, namely, mRMR feature list. At the beginning, this list is empty. For each feature *f* that is not in this list, compute its relevance to class labels, measured by *MI*(*f*, *c*), where *c* is a variable representing class labels, and redundancies to features that are already in the list, measured by the average MI between *f* and features in the current list. The difference of these two values is computed. The feature with highest difference is selected and appended to the list. When all features have been in the list, the procedures stop. Feature ranks in this list indicate the importance of features. Generally, features with high ranks are more important than those with low ranks.

The mRMR program used in this study was downloaded from http://penglab.janelia.org/proj/mRMR/. For convenience, it was executed using its default parameters.

### 2.5. Incremental Feature Selection (IFS)

Although mRMR method produced a feature list, it is still a problem that which features should be selected to construct the model. In view of this, this study employed the IFS method [21], which can aid to choose proper features for any given classification algorithm. In detail, on the basis of the mRMR feature list, IFS produces several feature subsets with a step interval as one. For instance, the first feature subset has the top feature in the mRMR list, and the second feature subset has the first two features, and so on. Then, a model based on a certain classification algorithm can be constructed on the training data, where samples are represented by feature in each feature subset. All constructed models are assessed by one cross-validation method [31]. The model yielding the best performance is picked up and called the optimum model. The feature subset used in this model is termed as the optimum feature subset.

### 2.6. Classification Algorithm

As mentioned above, IFS method needs one classification algorithm. Here, four classification algorithms were used, including (1) random forest (RF) [32], (2) support vector machine (SVM) [33], (3) *K*-nearest neighbor (kNN) [34], and (4) decision tree (DT) [35]. These algorithms have been widely used to tackle various medical problems [36–48]. Their brief descriptions are as follows.

#### 2.6.1. Random Forest

RF is a powerful and classic classification algorithm. In fact, it is an ensemble algorithm that contains several DTs. Each DT is built using two random selection procedures. The first procedure is to select samples, whereas the second procedure is for the selection of features. Given a query sample, each DT yields the prediction. RF integrates these predictions with majority voting. Although DT is a quite weak classification algorithm, RF is much more robust. Thus, it is always an important candidate for constructing prediction models.

#### 2.6.2. Support Vector Machine

SVM is another powerful and classic classification algorithm. Its main idea is to find out a hyperplane for separating samples in two classes. However, such hyperplane does not exist in many cases. SVM maps the original data with nonlinear pattern in low-dimensional space to a new data with linear pattern in high-dimensional space. Then, the hyperplane is constructed in such new space by maximizing interval between samples in two classes. Finally, it predicts the class label of a new sample according to which side of hyperplane this new data point belongs to.

#### 2.6.3. *K*-Nearest Neighbor

kNN is a simple but also efficient classification algorithm. It is not a strict machine learning algorithm because there is no training procedures. Several computational steps are conducted to determine the class of a test sample, such as computing the distance between the test sample and all training samples, ranking all training samples by those distances, selecting the *k* high-ranked training samples (i.e., nearest *k* neighbors), estimating the class label distribution of such *k* samples, and predicting the class label of the test sample as the one with the highest distribution frequency.

#### 2.6.4. Decision Tree

It aims to learn the human understanding classification and regression models. It generally uses IF–TEHN format to describe individual features' roles and weights in classification or regression models, thereby providing interpretative rules in a white box model. To date, several types of DT have been proposed. In this work, the CART algorithm with the Gini index was adopted to build DT model.

To quickly implement above-mentioned four classification algorithms, we employed corresponding packages collected in Scikit-learn (https://scikit-learn.org/stable/). They were executed using their default parameters.

### 2.7. Performance Measurement

In this study, the MCC [49] within 10-fold cross-validation [31] was used to evaluate each model's performance. A two-class classification model was obviously built here; thus, the MCC for binary problem was used as follows:

(5) $$\begin{array}{c} \text{MCC}=\frac{\text{TP}\times \text{TN}‐\text{FP}\times \text{FN}}{\sqrt{\left(\text{TP}+\text{FP}\right)\left(\text{TP}+\text{FN}\right)\left(\text{TN}+\text{FP}\right)\left(\text{TN}+\text{FN}\right)}}, \end{array}$$

where TP, TN, FP, and FN represent the sample numbers with true-positive, true-negative, false-positive, and false-negative predictions, respectively. The MCC value ranges from −1 to +1. When one classification model has the best performance, its MCC achieves +1.

Besides, we further computed other measurements to fully assess the performance of models, including sensitivity (SN) (same as recall), specificity (SP), accuracy (ACC), precision, and *F*1-measure. They can be calculated by

(6) $$\begin{array}{c} \left\{\begin{array}{c} \text{SN}=\text{Recall}=\frac{\text{TP}}{\text{TP}+\text{FN}},\\ \text{SP}=\frac{\text{TN}}{\text{TN}+\text{FP}},\\ \text{ACC}=\frac{\text{TP}+\text{TN}}{\text{TP}+\text{FN}+\text{TN}+\text{FP}},\\ \text{Precision}=\frac{\text{TP}}{\text{TP}+\text{FP}},\\ F1‐\text{measure}=\frac{2\times \text{Recall}\times \text{Precision}}{\text{Recall}+\text{Precision}}. \end{array}\right. \end{array}$$

### 2.8. Feature Frequency Visualization

Each feature was related to four sites in the sequence to understand the biological meaning of the extracted sequence features. After the optimum features for one classification algorithm were obtained, the related sites of each feature were picked up, and the frequency of each site was counted and plotted as a bar illustration.

## 3. Results

**Figure 1:**
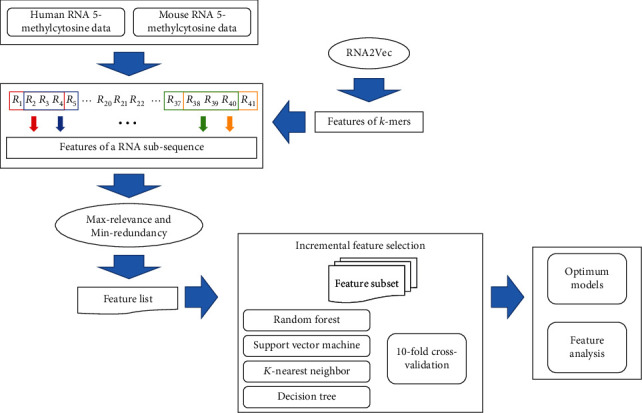
Flow chart to construct models for the prediction of m^5^C sites. A subsequence with 41 bp is used to represent each m^5^C site. Features of *k*-mers obtained by RNA2Vec are adopted to constitute features of the subsequence. All features are analysed by max-relevance and min-redundancy method. The outcome feature list is fed into incremental feature selection, incorporating four classification algorithms and 10-fold cross-validation, to construct optimum models.

In this study, we adopted the features of *k*-mers yielded by RNA2Vec to represent m^5^C sites. Some machine learning algorithms were employed to analyse these features and further build efficient models for identifying m^5^C site in RNA sequences. The whole procedures are shown in Figure 1. The detailed results were described in this section.

### 3.1. Selection of m^5^C Methylation-Associated Features for Mouse

**Figure 2:**
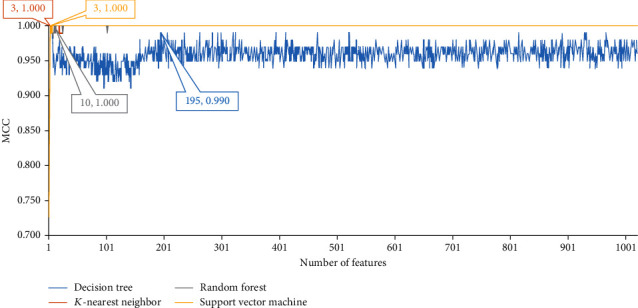
IFS curves with different classifiers on different numbers of sequence features on mouse m^5^C data.

For mouse m^5^C data, the mRMR method was first employed to analyse all 1020 features. An mRMR feature list was obtained. This list was fed into the IFS method that integrated one of four classification algorithms. On each feature subset, a model was built based on one classification algorithm and was further evaluated by 10-fold cross-validation. The performance of each model, including SN, SP, ACC, MCC, precision, and *F*1-measurem is provided in Supplementary file S1. MCC was selected as the key measurement. Accordingly, a curve is plotted in Figure 2 for each classification algorithm, which defined MCC as *y*-axis and number of features as the *x*-axis. For kNN, RF, and SVM, they can provide perfect performance with MCC = 1 when top 3, 10, and 3 features were adopted. The corresponding optimum kNN/RF/SVM model can be built with these features. The detailed performance of these models is listed in Table 1. All measurements reached the maximum of 1.000. For DT, the highest MCC was 0.990, which can be obtained by using top 195 features. Accordingly, the optimum DT model was set up with these features. Its detailed performance is listed in Table 1. It can be observed that all measurements were very high. All these indicated that the models with features yielded by RNA2Vec were quite efficient for identification of mouse m^5^C sites, also confirming the utility of these features to predict mouse m^5^C sites.

### 3.2. Selection of m^5^C Methylation-Associated Features for Human

**Figure 3:**
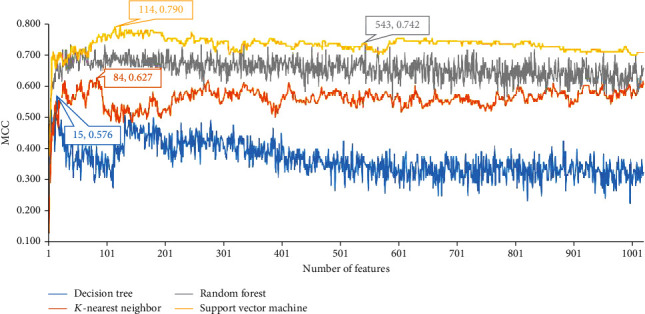
IFS curves with different classifiers on different numbers of sequence features on human m5C data.

For human m^5^C data, the same procedures were conducted. The performance of four classification algorithms on all possible feature subsets is provided in Supplementary file S2. Similarly, one curve was plotted for each classification algorithm (as shown in Figure 3). It can be observed that four classification algorithms yielded the highest MCC values of 0.576, 0.627, 0.742, and 0.790, respectively. Such performance was obtained by using top 15, 84, 543, and 114 features. Accordingly, optimum DT/kNN/RF/SVM model can be set up with these features. The detailed performance of these models is listed in Table 2. Evidently, the performance of these models was much lower than that of models for mouse.

### 3.3. Feature Frequency Analysis

The purpose of this study was not only to set up efficient models for prediction of m^5^C sites but also to discover novel patterns around the m^5^C sites, thereby providing more biological insights. Thus, we conducted feature frequency analysis in this section.

**Figure 4:**
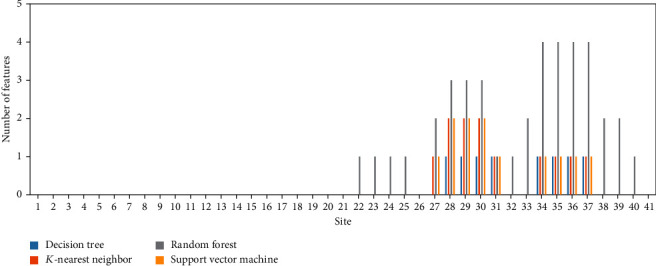
Frequency visualization for sequence features related to mouse m^5^C.

For mouse m^5^C data, four optimum models were built, which adopted some top features in the list. For each model, the number of selected features related to each site was counted. A bar chart was plotted to display such number of each site (as shown in Figure 4). Detailed discussion would be given in section “m5C Methylation-Associated Features in Mouse.”

**Figure 5:**
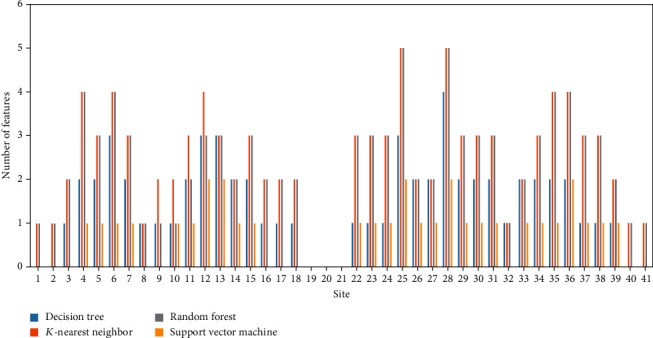
Frequency visualization for sequence features related to human m^5^C.

For human m^5^C data, we conducted the same operations. For each optimum model, the number of selected features related to each site is shown in Figure 5. Evidently, Figures 4 and 5 displayed quite different patterns, indicating the difference between mouse and human m^5^C sites. In section “m5C Methylation-Associated Features in Human,” a discussion would be given.

### 3.4. Comparison with Previous Models

This study used the mouse and human m^5^C data reported in [22]. In that study, several models with different classification algorithms were built and evaluated by 10-fold cross-validation, including DT, RF, SVM, Naïve Bayes, Bayes net, and logistic regression. The performance of models with DT, RF, and SVM is listed in Tables 3 and 4. For easy comparison, the performance of our models with same classification algorithms is also provided in these two tables. For mouse m^5^C data, our model with DT was slightly superior to the model in [22] with the same classification algorithm. As for other two classification algorithms, all models with one of them gave perfect performance. For human m^5^C data, DT provided better performance in our model than the model in [22], whereas other two classification algorithms generated lower performance in our model than the model in [22]. However, the gap was not very big. As a whole, our models and those in [22] were almost at the same level.

As mentioned in the above section, the purpose of this study further included the discovery of special patterns around m^5^C sites. This was the exclusive contributions of this study compared with the previous study.

## 4. Discussion

Multiple machine learning models were used to distinguish samples/sites with or without a different kind of RNA methylation (human or mouse), focusing on the significant pattern of RNA methylation as m^5^C [50–52]. With the help of IFS, the optimal number of essential features was selected for RNA methylation prediction. The distribution of predicted features in the 41 nt sequence was summarized to evaluate the discriminative contributions of different RNA loci for RNA methylation [53]. The detailed analyses on the results of m^5^C methylation in mouse or human tissues could be seen below, along with their respective distribution patterns.

### 4.1. m^5^C Methylation-Associated Features in Mouse

Multiple physiochemical features were used to encode the 41 nt sequence [53] of RNA. For the evaluation of the differential contribution of RNA sites for m^5^C methylation, four machine learning models were applied (DT, KNN, RF, and SVM) to identify the optimal combination of features for m^5^C methylation prediction. The distribution of features' respective RNA loci is shown in Figure 4. As identified from the feature distribution, all the selected features belong to the back end of the selected sequence, from 23 nt to 41 nt, just behind the candidate m^5^C methylation site (21 nt). In particular, two regions (27–31 nt and 34–37 nt) were predicted by at least three machine learning models to be associated with m^5^C methylation. According to recent publications based on the biological functions of m^5^C, the two kinds of m^5^C sites in multiple subgroups of RNAs are (1) type I m^5^C, which is followed by a G-rich triplet motif, and (2) type II m^5^C, which is adjacent to a downstream UCCA motif; both have specific sequence characteristics in the following region of m^5^C methylation loci [54], which corresponded with the prediction results in the present study. Further studies have also confirmed that specific regions in the downstream of m^5^C loci may have different sequence contexts, indicating that the feature-enriched regions in the prediction list in the present study could definitely be associated with m^5^C methylation efficiency. In 2019, a systematic analyses on mRNA 5-methylcytosine in mammals identified that the sequence context at the downstream of the captured m^5^C loci was alternate with different m^5^C locus methylation status, regulated by a specific 5-methylcytosine methyltransferase called NSUN2 [55, 56]. For comparison, the sequence before the m^5^C loci in mouse did not considerably change with NSUN2 wild-type, knock-out, or rescue status, implying that the m^5^C loci and their downstream sequence, especially for the following 10 nt sequence [55, 56], which corresponded with the prediction distribution in the present study. In addition, another similar 5-methylcytosine methyltransferase NSUN6 in mouse functioned as an mRNA m^5^C methyltransferase [54]. As a methyltransferase of type II m^5^C, the m^5^C targets of such gene have a symbolic downstream UCCA tail located at the first ambiguous peak (only predicted via RF method) in the prediction result of the present study (1–4 nt following the methylation region) [54]. Furthermore, different from the biological regulatory effects of NSUN2, the flanking regions around 15 nt were found to have another low base-pairing regions, which include more variants, by using the same procedure that detects the sequences with methyltransferase knock-out, rescue, and wild-type statuses [54]. This finding indicated the importance of sequence around such region. All in all, the predicted distribution of m^5^C methylation-associated loci has been validated by recent publications.

### 4.2. m^5^C Methylation-Associated Features in Human

The m^5^C-associated feature distribution among 40 flanking sequences (20 downstream and 20 upstream) from human tissues was also identified. According to the same publications [54, 55], the following 1–4 nt (22–26 nt) and 13–15 nt (34–37 nt) were also associated with the efficacy of m^5^C methylation, which corresponded with the prediction of the present study. As seen in Figures 4 and 5, the feature peaks in the downstream region (21–41 nt) were quite similar between the human and mouse data, reflecting the similarity of m^5^C methylation-associated patterns among different species. However, obvious differences were also observed, implying the presence of biological differences in m^5^C methylation among different species. In human beings, recent publications revealed that the distribution of RBP (RNA-binding protein) target density, which reflects the binding efficacy of the related region, was significant at the m^5^C candidate site, and gradually, not suddenly going down in both directions [56, 57]. Therefore, the sequences around m^5^C in each direction may also be not randomized but with specific sequence characteristics. Further, in 2015, an analysis on the regulatory homologous proteins of yeast and human from the same protein family (Nop2/NSUN/NOL family) showed that specific binding domains (e.g., SAM-binding domain) may be located behind the m^5^C loci, and they may affect regulatory effects. Therefore, although they were not directly validated, some nucleotides located before the m^5^C loci may be essential for the prediction of methylation status [58].

### 4.3. Biological Significance of Identified m^5^C Methylation-Associated Features

As summarized above, we identified m^5^C-associated features in mouse and human. The biological significance of identified m^5^C methylation features can be clustered into two parts:

- The specific and diverse distribution of m^5^C associated features in human or mouse. In this part, we identified that mouse m^5^C methylations are generally only associated with 28-31 nt and 34-37 nt regions in the 41 nt subsequence, while in human tissues, apart from 19-21 nt regions, most positions of the 41 nt sequence are associated with m^5^C methylation. These results identified key regulatory regions associated with m^5^C methylation and the differences between regulatory effects on m^5^C methylation in different species, reflecting the evolution conservation of m^5^C methylation regulatory mechanisms
- The downstream regulatory network associated with m^5^C methylation is essential for gene transcription and translation. Generally, m^5^C methylation can help bind hydrogen with guanine to stabilize the complete RNA structures and fold into unique spatial conformation [59]. According to recent publications, m^5^C regulator *NSUN2* has been shown to alter m^5^C capacity in certain RNA regions. Genes like *p27* (*KIPI*), *CDK1*, *p21*, and *ErbB2* have all been shown to be regulated by m^5^C methylation and further related to tumorigenesis [59, 60]. The sequence loci of m^5^C methylation have been shown to be specifically affects the downstream cell proliferation and inflammation associated pathway [61, 62], indicating the specific biological significance of m^5^C methylation. Therefore, the identification of different contribution of nucleotide from different sequence location can help demonstrate the specific regulatory effects for abnormal m^5^C methylation during different pathogenic conditions

Therefore, the identification of loci-related characters regulating m^5^C methylation between different species can not only help us reveal the consistence and evolution conservation of m^5^C methylation associated sequences but also connect specific sequence loci with significant m^5^C methylation-associated phenotypes or diseases.

## 5. Conclusions

All in all, as discussed above, the top optimal methylation sites in the prediction list have been supported by recent publications. The RNA methylation patterns were validated to be different in multiple species by comparing the results of m^5^C methylation-associated loci in human and mouse tissues. The discriminative feature distribution patterns for different methylation patterns were also detected by comparing the results of m^5^C distribution patterns. Therefore, the results not only evaluated the discriminative contribution of different loci for important RNA methylation patterns but also revealed the site distribution differences of m^5^C methylation types between species (human and mice).

## Figures and Tables

**Table 1:** Performance of models based on different classification algorithms for predicting mouse m^5^C sites.

Classification algorithm	Number of features	SN	SP	ACC	MCC	Precision	*F*1-measure
Decision tree	195	1.000	0.990	0.995	0.990	0.990	0.995
*K*-nearest neighbor	3	1.000	1.000	1.000	1.000	1.000	1.000
Random forest	10	1.000	1.000	1.000	1.000	1.000	1.000
Support vector machine	3	1.000	1.000	1.000	1.000	1.000	1.000

**Table 2:** Performance of models based on different classification algorithms for predicting human m^5^C sites.

Classification algorithm	Number of features	SN	SP	ACC	MCC	Precision	*F*1-measure
Decision tree	15	0.767	0.808	0.788	0.576	0.800	0.783
*K*-nearest neighbor	84	0.683	0.925	0.804	0.627	0.901	0.777
Random forest	543	0.875	0.867	0.871	0.742	0.868	0.871
Support vector machine	114	0.825	0.958	0.892	0.790	0.952	0.884

**Table 3:** Comparison with previous models on mouse m^5^C data.

Classification algorithm	Model	SN	SP	ACC	MCC
Decision tree	Our model	1.000	0.990	0.995	0.990
	Model in [22]	1.000	0.835	0.918	0.847

Random forest	Our model	1.000	1.000	1.000	1.000
	Model in [22]	1.000	1.000	1.000	1.000

Support vector machine	Our model	1.000	1.000	1.000	1.000
	Model in [22]	1.000	1.000	1.000	1.000

**Table 4:** Comparison with previous models on human m^5^C data.

Classification algorithm	Model	SN	SP	ACC	MCC
Decision tree	Our model	0.767	0.808	0.788	0.576
	Model in [22]	0.783	0.783	0.783	0.567

Random forest	Our model	0.875	0.867	0.871	0.742
	Model in [22]	0.900	0.917	0.908	0.817

Support vector machine	Our model	0.825	0.958	0.892	0.790
	Model in [22]	0.842	0.967	0.904	0.815

## Data Availability

The original data used to support the findings of this study are available at iRNA-m^5^C (http://lin-group.cn/server/iRNA-m5C/download.html).

## Supplementary Materials

Supplementary Materials Supplementary Material S1: performance of IFS for mouse m^5^C sites. Supplementary Material S2: performance of IFS for human m^5^C sites.
