# Supplementary material for: Predicting RNA 5-Methylcytosine Sites by Using Essential Sequence Features and Distributions
Source: Biomed Res Int. 2022 Jan 13;2022:4035462. doi: 10.1155/2022/4035462 (PMC8776474; doi:10.1155/2022/4035462)
Supplement: Supplementary Materials — Supplementary Material S1: performance of IFS for mouse m5C sites. Supplementary Material S2: performance of IFS for human m5C sites. [file 4035462.f1.zip › Supp-S1 (1).pdf]

Supplementary Material S1. Performance of IFS for mouse m^5C sites

(1) Decision tree

Number of features	SN	SP	ACC	MCC	Precision	F1-measure
1	1.000	0.938	0.969	0.940	0.942	0.970
2	1.000	0.979	0.990	0.980	0.980	0.990
3	1.000	0.979	0.990	0.980	0.980	0.990
4	1.000	0.979	0.990	0.980	0.980	0.990
5	1.000	0.979	0.990	0.980	0.980	0.990
6	1.000	0.979	0.990	0.980	0.980	0.990
7	1.000	0.979	0.990	0.980	0.980	0.990
8	1.000	0.938	0.969	0.940	0.942	0.970
9	1.000	0.959	0.979	0.960	0.960	0.980
10	1.000	0.938	0.969	0.940	0.942	0.970
11	1.000	0.928	0.964	0.930	0.933	0.965
12	1.000	0.948	0.974	0.950	0.951	0.975
13	1.000	0.948	0.974	0.950	0.951	0.975
14	1.000	0.959	0.979	0.960	0.960	0.980
15	1.000	0.948	0.974	0.950	0.951	0.975
16	1.000	0.948	0.974	0.950	0.951	0.975
17	1.000	0.969	0.985	0.970	0.970	0.985
18	1.000	0.959	0.979	0.960	0.960	0.980
19	1.000	0.979	0.990	0.980	0.980	0.990
20	1.000	0.938	0.969	0.940	0.942	0.970
21	1.000	0.948	0.974	0.950	0.951	0.975
22	1.000	0.938	0.969	0.940	0.942	0.970
23	1.000	0.969	0.985	0.970	0.970	0.985
24	1.000	0.928	0.964	0.930	0.933	0.965
25	1.000	0.938	0.969	0.940	0.942	0.970
26	1.000	0.959	0.979	0.960	0.960	0.980
27	1.000	0.948	0.974	0.950	0.951	0.975
28	1.000	0.938	0.969	0.940	0.942	0.970
29	1.000	0.948	0.974	0.950	0.951	0.975
30	1.000	0.948	0.974	0.950	0.951	0.975
31	1.000	0.928	0.964	0.930	0.933	0.965
32	1.000	0.959	0.979	0.960	0.960	0.980
33	1.000	0.959	0.979	0.960	0.960	0.980
34	1.000	0.959	0.979	0.960	0.960	0.980
35	1.000	0.959	0.979	0.960	0.960	0.980
36	1.000	0.928	0.964	0.930	0.933	0.965
37	1.000	0.969	0.985	0.970	0.970	0.985
38	1.000	0.959	0.979	0.960	0.960	0.980

39	1.000	0.959	0.979	0.960	0.960	0.980
40	1.000	0.948	0.974	0.950	0.951	0.975
41	1.000	0.938	0.969	0.940	0.942	0.970
42	1.000	0.959	0.979	0.960	0.960	0.980
43	1.000	0.948	0.974	0.950	0.951	0.975
44	1.000	0.959	0.979	0.960	0.960	0.980
45	1.000	0.969	0.985	0.970	0.970	0.985
46	1.000	0.938	0.969	0.940	0.942	0.970
47	1.000	0.948	0.974	0.950	0.951	0.975
48	1.000	0.948	0.974	0.950	0.951	0.975
49	1.000	0.959	0.979	0.960	0.960	0.980
50	1.000	0.948	0.974	0.950	0.951	0.975
51	1.000	0.959	0.979	0.960	0.960	0.980
52	1.000	0.938	0.969	0.940	0.942	0.970
53	1.000	0.948	0.974	0.950	0.951	0.975
54	1.000	0.928	0.964	0.930	0.933	0.965
55	1.000	0.959	0.979	0.960	0.960	0.980
56	1.000	0.948	0.974	0.950	0.951	0.975
57	1.000	0.948	0.974	0.950	0.951	0.975
58	1.000	0.948	0.974	0.950	0.951	0.975
59	1.000	0.969	0.985	0.970	0.970	0.985
60	1.000	0.948	0.974	0.950	0.951	0.975
61	1.000	0.959	0.979	0.960	0.960	0.980
62	1.000	0.938	0.969	0.940	0.942	0.970
63	1.000	0.948	0.974	0.950	0.951	0.975
64	1.000	0.938	0.969	0.940	0.942	0.970
65	1.000	0.938	0.969	0.940	0.942	0.970
66	1.000	0.948	0.974	0.950	0.951	0.975
67	1.000	0.948	0.974	0.950	0.951	0.975
68	1.000	0.948	0.974	0.950	0.951	0.975
69	1.000	0.959	0.979	0.960	0.960	0.980
70	1.000	0.938	0.969	0.940	0.942	0.970
71	1.000	0.948	0.974	0.950	0.951	0.975
72	1.000	0.948	0.974	0.950	0.951	0.975
73	1.000	0.948	0.974	0.950	0.951	0.975
74	1.000	0.959	0.979	0.960	0.960	0.980
75	1.000	0.928	0.964	0.930	0.933	0.965
76	1.000	0.938	0.969	0.940	0.942	0.970
77	1.000	0.948	0.974	0.950	0.951	0.975
78	1.000	0.948	0.974	0.950	0.951	0.975
79	1.000	0.969	0.985	0.970	0.970	0.985
80	1.000	0.928	0.964	0.930	0.933	0.965

81	1.000	0.928	0.964	0.930	0.933	0.965
82	1.000	0.969	0.985	0.970	0.970	0.985
83	1.000	0.918	0.959	0.921	0.924	0.960
84	1.000	0.959	0.979	0.960	0.960	0.980
85	1.000	0.928	0.964	0.930	0.933	0.965
86	1.000	0.918	0.959	0.921	0.924	0.960
87	1.000	0.948	0.974	0.950	0.951	0.975
88	1.000	0.928	0.964	0.930	0.933	0.965
89	1.000	0.928	0.964	0.930	0.933	0.965
90	1.000	0.959	0.979	0.960	0.960	0.980
91	1.000	0.938	0.969	0.940	0.942	0.970
92	1.000	0.948	0.974	0.950	0.951	0.975
93	1.000	0.938	0.969	0.940	0.942	0.970
94	1.000	0.928	0.964	0.930	0.933	0.965
95	1.000	0.959	0.979	0.960	0.960	0.980
96	1.000	0.907	0.954	0.911	0.915	0.956
97	1.000	0.938	0.969	0.940	0.942	0.970
98	1.000	0.959	0.979	0.960	0.960	0.980
99	1.000	0.928	0.964	0.930	0.933	0.965
100	1.000	0.938	0.969	0.940	0.942	0.970
101	1.000	0.928	0.964	0.930	0.933	0.965
102	1.000	0.959	0.979	0.960	0.960	0.980
103	1.000	0.918	0.959	0.921	0.924	0.960
104	1.000	0.948	0.974	0.950	0.951	0.975
105	1.000	0.918	0.959	0.921	0.924	0.960
106	1.000	0.959	0.979	0.960	0.960	0.980
107	1.000	0.938	0.969	0.940	0.942	0.970
108	1.000	0.959	0.979	0.960	0.960	0.980
109	1.000	0.928	0.964	0.930	0.933	0.965
110	1.000	0.948	0.974	0.950	0.951	0.975
111	1.000	0.948	0.974	0.950	0.951	0.975
112	1.000	0.918	0.959	0.921	0.924	0.960
113	1.000	0.918	0.959	0.921	0.924	0.960
114	1.000	0.928	0.964	0.930	0.933	0.965
115	1.000	0.918	0.959	0.921	0.924	0.960
116	1.000	0.948	0.974	0.950	0.951	0.975
117	1.000	0.959	0.979	0.960	0.960	0.980
118	1.000	0.918	0.959	0.921	0.924	0.960
119	1.000	0.928	0.964	0.930	0.933	0.965
120	1.000	0.948	0.974	0.950	0.951	0.975
121	1.000	0.928	0.964	0.930	0.933	0.965
122	1.000	0.918	0.959	0.921	0.924	0.960

123	1.000	0.938	0.969	0.940	0.942	0.970
124	1.000	0.938	0.969	0.940	0.942	0.970
125	1.000	0.918	0.959	0.921	0.924	0.960
126	1.000	0.938	0.969	0.940	0.942	0.970
127	1.000	0.948	0.974	0.950	0.951	0.975
128	1.000	0.948	0.974	0.950	0.951	0.975
129	1.000	0.948	0.974	0.950	0.951	0.975
130	1.000	0.948	0.974	0.950	0.951	0.975
131	1.000	0.959	0.979	0.960	0.960	0.980
132	1.000	0.948	0.974	0.950	0.951	0.975
133	1.000	0.938	0.969	0.940	0.942	0.970
134	1.000	0.959	0.979	0.960	0.960	0.980
135	1.000	0.928	0.964	0.930	0.933	0.965
136	1.000	0.938	0.969	0.940	0.942	0.970
137	1.000	0.959	0.979	0.960	0.960	0.980
138	1.000	0.928	0.964	0.930	0.933	0.965
139	1.000	0.948	0.974	0.950	0.951	0.975
140	1.000	0.918	0.959	0.921	0.924	0.960
141	1.000	0.938	0.969	0.940	0.942	0.970
142	1.000	0.938	0.969	0.940	0.942	0.970
143	1.000	0.907	0.954	0.911	0.915	0.956
144	1.000	0.948	0.974	0.950	0.951	0.975
145	1.000	0.948	0.974	0.950	0.951	0.975
146	1.000	0.948	0.974	0.950	0.951	0.975
147	1.000	0.928	0.964	0.930	0.933	0.965
148	1.000	0.948	0.974	0.950	0.951	0.975
149	1.000	0.928	0.964	0.930	0.933	0.965
150	1.000	0.928	0.964	0.930	0.933	0.965
151	1.000	0.938	0.969	0.940	0.942	0.970
152	1.000	0.959	0.979	0.960	0.960	0.980
153	1.000	0.948	0.974	0.950	0.951	0.975
154	1.000	0.938	0.969	0.940	0.942	0.970
155	1.000	0.938	0.969	0.940	0.942	0.970
156	1.000	0.959	0.979	0.960	0.960	0.980
157	1.000	0.928	0.964	0.930	0.933	0.965
158	1.000	0.979	0.990	0.980	0.980	0.990
159	1.000	0.948	0.974	0.950	0.951	0.975
160	1.000	0.959	0.979	0.960	0.960	0.980
161	1.000	0.948	0.974	0.950	0.951	0.975
162	1.000	0.959	0.979	0.960	0.960	0.980
163	1.000	0.959	0.979	0.960	0.960	0.980
164	1.000	0.959	0.979	0.960	0.960	0.980

165	1.000	0.948	0.974	0.950	0.951	0.975
166	1.000	0.948	0.974	0.950	0.951	0.975
167	1.000	0.969	0.985	0.970	0.970	0.985
168	1.000	0.969	0.985	0.970	0.970	0.985
169	1.000	0.969	0.985	0.970	0.970	0.985
170	1.000	0.948	0.974	0.950	0.951	0.975
171	1.000	0.948	0.974	0.950	0.951	0.975
172	1.000	0.969	0.985	0.970	0.970	0.985
173	1.000	0.959	0.979	0.960	0.960	0.980
174	1.000	0.979	0.990	0.980	0.980	0.990
175	1.000	0.959	0.979	0.960	0.960	0.980
176	1.000	0.959	0.979	0.960	0.960	0.980
177	1.000	0.959	0.979	0.960	0.960	0.980
178	1.000	0.969	0.985	0.970	0.970	0.985
179	1.000	0.969	0.985	0.970	0.970	0.985
180	1.000	0.959	0.979	0.960	0.960	0.980
181	1.000	0.948	0.974	0.950	0.951	0.975
182	1.000	0.969	0.985	0.970	0.970	0.985
183	1.000	0.948	0.974	0.950	0.951	0.975
184	1.000	0.969	0.985	0.970	0.970	0.985
185	1.000	0.979	0.990	0.980	0.980	0.990
186	1.000	0.969	0.985	0.970	0.970	0.985
187	1.000	0.948	0.974	0.950	0.951	0.975
188	1.000	0.948	0.974	0.950	0.951	0.975
189	1.000	0.948	0.974	0.950	0.951	0.975
190	1.000	0.979	0.990	0.980	0.980	0.990
191	1.000	0.938	0.969	0.940	0.942	0.970
192	1.000	0.979	0.990	0.980	0.980	0.990
193	1.000	0.948	0.974	0.950	0.951	0.975
194	1.000	0.948	0.974	0.950	0.951	0.975
195	1.000	0.990	0.995	0.990	0.990	0.995
196	1.000	0.948	0.974	0.950	0.951	0.975
197	1.000	0.979	0.990	0.980	0.980	0.990
198	1.000	0.959	0.979	0.960	0.960	0.980
199	1.000	0.969	0.985	0.970	0.970	0.985
200	1.000	0.979	0.990	0.980	0.980	0.990
201	1.000	0.969	0.985	0.970	0.970	0.985
202	1.000	0.959	0.979	0.960	0.960	0.980
203	1.000	0.959	0.979	0.960	0.960	0.980
204	1.000	0.948	0.974	0.950	0.951	0.975
205	1.000	0.969	0.985	0.970	0.970	0.985
206	1.000	0.990	0.995	0.990	0.990	0.995

207	1.000	0.969	0.985	0.970	0.970	0.985
208	1.000	0.938	0.969	0.940	0.942	0.970
209	1.000	0.979	0.990	0.980	0.980	0.990
210	1.000	0.959	0.979	0.960	0.960	0.980
211	1.000	0.959	0.979	0.960	0.960	0.980
212	1.000	0.948	0.974	0.950	0.951	0.975
213	1.000	0.979	0.990	0.980	0.980	0.990
214	1.000	0.948	0.974	0.950	0.951	0.975
215	1.000	0.959	0.979	0.960	0.960	0.980
216	1.000	0.979	0.990	0.980	0.980	0.990
217	1.000	0.948	0.974	0.950	0.951	0.975
218	1.000	0.948	0.974	0.950	0.951	0.975
219	1.000	0.948	0.974	0.950	0.951	0.975
220	1.000	0.979	0.990	0.980	0.980	0.990
221	1.000	0.959	0.979	0.960	0.960	0.980
222	1.000	0.959	0.979	0.960	0.960	0.980
223	1.000	0.959	0.979	0.960	0.960	0.980
224	1.000	0.969	0.985	0.970	0.970	0.985
225	1.000	0.969	0.985	0.970	0.970	0.985
226	1.000	0.948	0.974	0.950	0.951	0.975
227	1.000	0.969	0.985	0.970	0.970	0.985
228	1.000	0.969	0.985	0.970	0.970	0.985
229	1.000	0.969	0.985	0.970	0.970	0.985
230	1.000	0.948	0.974	0.950	0.951	0.975
231	1.000	0.979	0.990	0.980	0.980	0.990
232	1.000	0.969	0.985	0.970	0.970	0.985
233	1.000	0.938	0.969	0.940	0.942	0.970
234	1.000	0.959	0.979	0.960	0.960	0.980
235	1.000	0.990	0.995	0.990	0.990	0.995
236	1.000	0.969	0.985	0.970	0.970	0.985
237	1.000	0.969	0.985	0.970	0.970	0.985
238	1.000	0.959	0.979	0.960	0.960	0.980
239	1.000	0.959	0.979	0.960	0.960	0.980
240	1.000	0.948	0.974	0.950	0.951	0.975
241	1.000	0.969	0.985	0.970	0.970	0.985
242	1.000	0.948	0.974	0.950	0.951	0.975
243	1.000	0.959	0.979	0.960	0.960	0.980
244	1.000	0.948	0.974	0.950	0.951	0.975
245	1.000	0.969	0.985	0.970	0.970	0.985
246	1.000	0.948	0.974	0.950	0.951	0.975
247	1.000	0.969	0.985	0.970	0.970	0.985
248	1.000	0.948	0.974	0.950	0.951	0.975

249	1.000	0.959	0.979	0.960	0.960	0.980
250	1.000	0.948	0.974	0.950	0.951	0.975
251	1.000	0.948	0.974	0.950	0.951	0.975
252	1.000	0.959	0.979	0.960	0.960	0.980
253	1.000	0.969	0.985	0.970	0.970	0.985
254	1.000	0.948	0.974	0.950	0.951	0.975
255	1.000	0.959	0.979	0.960	0.960	0.980
256	1.000	0.948	0.974	0.950	0.951	0.975
257	1.000	0.979	0.990	0.980	0.980	0.990
258	1.000	0.948	0.974	0.950	0.951	0.975
259	1.000	0.948	0.974	0.950	0.951	0.975
260	1.000	0.948	0.974	0.950	0.951	0.975
261	1.000	0.959	0.979	0.960	0.960	0.980
262	1.000	0.990	0.995	0.990	0.990	0.995
263	1.000	0.948	0.974	0.950	0.951	0.975
264	1.000	0.959	0.979	0.960	0.960	0.980
265	1.000	0.948	0.974	0.950	0.951	0.975
266	1.000	0.959	0.979	0.960	0.960	0.980
267	1.000	0.959	0.979	0.960	0.960	0.980
268	1.000	0.969	0.985	0.970	0.970	0.985
269	1.000	0.948	0.974	0.950	0.951	0.975
270	1.000	0.948	0.974	0.950	0.951	0.975
271	1.000	0.969	0.985	0.970	0.970	0.985
272	1.000	0.969	0.985	0.970	0.970	0.985
273	1.000	0.979	0.990	0.980	0.980	0.990
274	1.000	0.969	0.985	0.970	0.970	0.985
275	1.000	0.979	0.990	0.980	0.980	0.990
276	1.000	0.948	0.974	0.950	0.951	0.975
277	1.000	0.948	0.974	0.950	0.951	0.975
278	1.000	0.979	0.990	0.980	0.980	0.990
279	1.000	0.969	0.985	0.970	0.970	0.985
280	1.000	0.948	0.974	0.950	0.951	0.975
281	1.000	0.959	0.979	0.960	0.960	0.980
282	1.000	0.969	0.985	0.970	0.970	0.985
283	1.000	0.969	0.985	0.970	0.970	0.985
284	1.000	0.990	0.995	0.990	0.990	0.995
285	1.000	0.948	0.974	0.950	0.951	0.975
286	1.000	0.948	0.974	0.950	0.951	0.975
287	1.000	0.959	0.979	0.960	0.960	0.980
288	1.000	0.969	0.985	0.970	0.970	0.985
289	1.000	0.969	0.985	0.970	0.970	0.985
290	1.000	0.969	0.985	0.970	0.970	0.985

291	1.000	0.948	0.974	0.950	0.951	0.975
292	1.000	0.948	0.974	0.950	0.951	0.975
293	1.000	0.990	0.995	0.990	0.990	0.995
294	1.000	0.979	0.990	0.980	0.980	0.990
295	1.000	0.938	0.969	0.940	0.942	0.970
296	1.000	0.969	0.985	0.970	0.970	0.985
297	1.000	0.948	0.974	0.950	0.951	0.975
298	1.000	0.948	0.974	0.950	0.951	0.975
299	1.000	0.969	0.985	0.970	0.970	0.985
300	1.000	0.990	0.995	0.990	0.990	0.995
301	1.000	0.948	0.974	0.950	0.951	0.975
302	1.000	0.979	0.990	0.980	0.980	0.990
303	1.000	0.959	0.979	0.960	0.960	0.980
304	1.000	0.969	0.985	0.970	0.970	0.985
305	1.000	0.969	0.985	0.970	0.970	0.985
306	1.000	0.969	0.985	0.970	0.970	0.985
307	1.000	0.959	0.979	0.960	0.960	0.980
308	1.000	0.979	0.990	0.980	0.980	0.990
309	1.000	0.948	0.974	0.950	0.951	0.975
310	1.000	0.969	0.985	0.970	0.970	0.985
311	1.000	0.948	0.974	0.950	0.951	0.975
312	1.000	0.948	0.974	0.950	0.951	0.975
313	1.000	0.948	0.974	0.950	0.951	0.975
314	1.000	0.948	0.974	0.950	0.951	0.975
315	1.000	0.969	0.985	0.970	0.970	0.985
316	1.000	0.948	0.974	0.950	0.951	0.975
317	1.000	0.959	0.979	0.960	0.960	0.980
318	1.000	0.959	0.979	0.960	0.960	0.980
319	1.000	0.969	0.985	0.970	0.970	0.985
320	1.000	0.990	0.995	0.990	0.990	0.995
321	1.000	0.948	0.974	0.950	0.951	0.975
322	1.000	0.959	0.979	0.960	0.960	0.980
323	1.000	0.948	0.974	0.950	0.951	0.975
324	1.000	0.990	0.995	0.990	0.990	0.995
325	1.000	0.959	0.979	0.960	0.960	0.980
326	1.000	0.959	0.979	0.960	0.960	0.980
327	1.000	0.969	0.985	0.970	0.970	0.985
328	1.000	0.969	0.985	0.970	0.970	0.985
329	1.000	0.969	0.985	0.970	0.970	0.985
330	1.000	0.959	0.979	0.960	0.960	0.980
331	1.000	0.979	0.990	0.980	0.980	0.990
332	1.000	0.969	0.985	0.970	0.970	0.985

333	1.000	0.948	0.974	0.950	0.951	0.975
334	1.000	0.969	0.985	0.970	0.970	0.985
335	1.000	0.979	0.990	0.980	0.980	0.990
336	1.000	0.948	0.974	0.950	0.951	0.975
337	1.000	0.959	0.979	0.960	0.960	0.980
338	1.000	0.959	0.979	0.960	0.960	0.980
339	1.000	0.959	0.979	0.960	0.960	0.980
340	1.000	0.969	0.985	0.970	0.970	0.985
341	1.000	0.969	0.985	0.970	0.970	0.985
342	1.000	0.959	0.979	0.960	0.960	0.980
343	1.000	0.969	0.985	0.970	0.970	0.985
344	1.000	0.969	0.985	0.970	0.970	0.985
345	1.000	0.959	0.979	0.960	0.960	0.980
346	1.000	0.948	0.974	0.950	0.951	0.975
347	1.000	0.969	0.985	0.970	0.970	0.985
348	1.000	0.948	0.974	0.950	0.951	0.975
349	1.000	0.948	0.974	0.950	0.951	0.975
350	1.000	0.959	0.979	0.960	0.960	0.980
351	1.000	0.969	0.985	0.970	0.970	0.985
352	1.000	0.969	0.985	0.970	0.970	0.985
353	1.000	0.948	0.974	0.950	0.951	0.975
354	1.000	0.948	0.974	0.950	0.951	0.975
355	1.000	0.948	0.974	0.950	0.951	0.975
356	1.000	0.969	0.985	0.970	0.970	0.985
357	1.000	0.969	0.985	0.970	0.970	0.985
358	1.000	0.959	0.979	0.960	0.960	0.980
359	1.000	0.948	0.974	0.950	0.951	0.975
360	1.000	0.969	0.985	0.970	0.970	0.985
361	1.000	0.959	0.979	0.960	0.960	0.980
362	1.000	0.969	0.985	0.970	0.970	0.985
363	1.000	0.948	0.974	0.950	0.951	0.975
364	1.000	0.959	0.979	0.960	0.960	0.980
365	1.000	0.959	0.979	0.960	0.960	0.980
366	1.000	0.938	0.969	0.940	0.942	0.970
367	1.000	0.979	0.990	0.980	0.980	0.990
368	1.000	0.959	0.979	0.960	0.960	0.980
369	1.000	0.969	0.985	0.970	0.970	0.985
370	1.000	0.948	0.974	0.950	0.951	0.975
371	1.000	0.969	0.985	0.970	0.970	0.985
372	1.000	0.959	0.979	0.960	0.960	0.980
373	1.000	0.938	0.969	0.940	0.942	0.970
374	1.000	0.969	0.985	0.970	0.970	0.985

375	1.000	0.969	0.985	0.970	0.970	0.985
376	1.000	0.969	0.985	0.970	0.970	0.985
377	1.000	0.948	0.974	0.950	0.951	0.975
378	1.000	0.948	0.974	0.950	0.951	0.975
379	1.000	0.948	0.974	0.950	0.951	0.975
380	1.000	0.959	0.979	0.960	0.960	0.980
381	1.000	0.969	0.985	0.970	0.970	0.985
382	1.000	0.959	0.979	0.960	0.960	0.980
383	1.000	0.959	0.979	0.960	0.960	0.980
384	1.000	0.948	0.974	0.950	0.951	0.975
385	1.000	0.969	0.985	0.970	0.970	0.985
386	1.000	0.959	0.979	0.960	0.960	0.980
387	1.000	0.969	0.985	0.970	0.970	0.985
388	1.000	0.948	0.974	0.950	0.951	0.975
389	1.000	0.938	0.969	0.940	0.942	0.970
390	1.000	0.948	0.974	0.950	0.951	0.975
391	1.000	0.959	0.979	0.960	0.960	0.980
392	1.000	0.979	0.990	0.980	0.980	0.990
393	1.000	0.959	0.979	0.960	0.960	0.980
394	1.000	0.990	0.995	0.990	0.990	0.995
395	1.000	0.959	0.979	0.960	0.960	0.980
396	1.000	0.969	0.985	0.970	0.970	0.985
397	1.000	0.969	0.985	0.970	0.970	0.985
398	1.000	0.959	0.979	0.960	0.960	0.980
399	1.000	0.969	0.985	0.970	0.970	0.985
400	1.000	0.969	0.985	0.970	0.970	0.985
401	1.000	0.948	0.974	0.950	0.951	0.975
402	1.000	0.948	0.974	0.950	0.951	0.975
403	1.000	0.959	0.979	0.960	0.960	0.980
404	1.000	0.969	0.985	0.970	0.970	0.985
405	1.000	0.969	0.985	0.970	0.970	0.985
406	1.000	0.959	0.979	0.960	0.960	0.980
407	1.000	0.948	0.974	0.950	0.951	0.975
408	1.000	0.938	0.969	0.940	0.942	0.970
409	1.000	0.959	0.979	0.960	0.960	0.980
410	1.000	0.948	0.974	0.950	0.951	0.975
411	1.000	0.990	0.995	0.990	0.990	0.995
412	1.000	0.948	0.974	0.950	0.951	0.975
413	1.000	0.979	0.990	0.980	0.980	0.990
414	1.000	0.979	0.990	0.980	0.980	0.990
415	1.000	0.969	0.985	0.970	0.970	0.985
416	1.000	0.959	0.979	0.960	0.960	0.980

417	1.000	0.969	0.985	0.970	0.970	0.985
418	1.000	0.948	0.974	0.950	0.951	0.975
419	1.000	0.969	0.985	0.970	0.970	0.985
420	1.000	0.948	0.974	0.950	0.951	0.975
421	1.000	0.979	0.990	0.980	0.980	0.990
422	1.000	0.979	0.990	0.980	0.980	0.990
423	1.000	0.959	0.979	0.960	0.960	0.980
424	1.000	0.959	0.979	0.960	0.960	0.980
425	1.000	0.948	0.974	0.950	0.951	0.975
426	1.000	0.959	0.979	0.960	0.960	0.980
427	1.000	0.969	0.985	0.970	0.970	0.985
428	1.000	0.948	0.974	0.950	0.951	0.975
429	1.000	0.969	0.985	0.970	0.970	0.985
430	1.000	0.948	0.974	0.950	0.951	0.975
431	1.000	0.969	0.985	0.970	0.970	0.985
432	1.000	0.969	0.985	0.970	0.970	0.985
433	1.000	0.959	0.979	0.960	0.960	0.980
434	1.000	0.969	0.985	0.970	0.970	0.985
435	1.000	0.969	0.985	0.970	0.970	0.985
436	1.000	0.959	0.979	0.960	0.960	0.980
437	1.000	0.948	0.974	0.950	0.951	0.975
438	1.000	0.948	0.974	0.950	0.951	0.975
439	1.000	0.959	0.979	0.960	0.960	0.980
440	1.000	0.969	0.985	0.970	0.970	0.985
441	1.000	0.959	0.979	0.960	0.960	0.980
442	1.000	0.948	0.974	0.950	0.951	0.975
443	1.000	0.969	0.985	0.970	0.970	0.985
444	1.000	0.948	0.974	0.950	0.951	0.975
445	1.000	0.969	0.985	0.970	0.970	0.985
446	1.000	0.969	0.985	0.970	0.970	0.985
447	1.000	0.969	0.985	0.970	0.970	0.985
448	1.000	0.959	0.979	0.960	0.960	0.980
449	1.000	0.959	0.979	0.960	0.960	0.980
450	1.000	0.969	0.985	0.970	0.970	0.985
451	1.000	0.969	0.985	0.970	0.970	0.985
452	1.000	0.969	0.985	0.970	0.970	0.985
453	1.000	0.959	0.979	0.960	0.960	0.980
454	1.000	0.959	0.979	0.960	0.960	0.980
455	1.000	0.969	0.985	0.970	0.970	0.985
456	1.000	0.948	0.974	0.950	0.951	0.975
457	1.000	0.969	0.985	0.970	0.970	0.985
458	1.000	0.969	0.985	0.970	0.970	0.985

459	1.000	0.969	0.985	0.970	0.970	0.985
460	1.000	0.938	0.969	0.940	0.942	0.970
461	1.000	0.948	0.974	0.950	0.951	0.975
462	1.000	0.959	0.979	0.960	0.960	0.980
463	1.000	0.948	0.974	0.950	0.951	0.975
464	1.000	0.959	0.979	0.960	0.960	0.980
465	1.000	0.969	0.985	0.970	0.970	0.985
466	1.000	0.990	0.995	0.990	0.990	0.995
467	1.000	0.979	0.990	0.980	0.980	0.990
468	1.000	0.948	0.974	0.950	0.951	0.975
469	1.000	0.938	0.969	0.940	0.942	0.970
470	1.000	0.969	0.985	0.970	0.970	0.985
471	1.000	0.959	0.979	0.960	0.960	0.980
472	1.000	0.959	0.979	0.960	0.960	0.980
473	1.000	0.938	0.969	0.940	0.942	0.970
474	1.000	0.948	0.974	0.950	0.951	0.975
475	1.000	0.969	0.985	0.970	0.970	0.985
476	1.000	0.948	0.974	0.950	0.951	0.975
477	1.000	0.948	0.974	0.950	0.951	0.975
478	1.000	0.948	0.974	0.950	0.951	0.975
479	1.000	0.959	0.979	0.960	0.960	0.980
480	1.000	0.938	0.969	0.940	0.942	0.970
481	1.000	0.959	0.979	0.960	0.960	0.980
482	1.000	0.959	0.979	0.960	0.960	0.980
483	1.000	0.969	0.985	0.970	0.970	0.985
484	1.000	0.969	0.985	0.970	0.970	0.985
485	1.000	0.969	0.985	0.970	0.970	0.985
486	1.000	0.938	0.969	0.940	0.942	0.970
487	1.000	0.959	0.979	0.960	0.960	0.980
488	1.000	0.959	0.979	0.960	0.960	0.980
489	1.000	0.969	0.985	0.970	0.970	0.985
490	1.000	0.938	0.969	0.940	0.942	0.970
491	1.000	0.959	0.979	0.960	0.960	0.980
492	1.000	0.969	0.985	0.970	0.970	0.985
493	1.000	0.959	0.979	0.960	0.960	0.980
494	1.000	0.969	0.985	0.970	0.970	0.985
495	1.000	0.979	0.990	0.980	0.980	0.990
496	1.000	0.959	0.979	0.960	0.960	0.980
497	1.000	0.948	0.974	0.950	0.951	0.975
498	1.000	0.938	0.969	0.940	0.942	0.970
499	1.000	0.959	0.979	0.960	0.960	0.980
500	1.000	0.948	0.974	0.950	0.951	0.975

501	1.000	0.948	0.974	0.950	0.951	0.975
502	1.000	0.948	0.974	0.950	0.951	0.975
503	1.000	0.959	0.979	0.960	0.960	0.980
504	1.000	0.979	0.990	0.980	0.980	0.990
505	1.000	0.938	0.969	0.940	0.942	0.970
506	1.000	0.948	0.974	0.950	0.951	0.975
507	1.000	0.959	0.979	0.960	0.960	0.980
508	1.000	0.969	0.985	0.970	0.970	0.985
509	1.000	0.969	0.985	0.970	0.970	0.985
510	1.000	0.969	0.985	0.970	0.970	0.985
511	1.000	0.948	0.974	0.950	0.951	0.975
512	1.000	0.959	0.979	0.960	0.960	0.980
513	1.000	0.938	0.969	0.940	0.942	0.970
514	1.000	0.969	0.985	0.970	0.970	0.985
515	1.000	0.969	0.985	0.970	0.970	0.985
516	1.000	0.959	0.979	0.960	0.960	0.980
517	1.000	0.969	0.985	0.970	0.970	0.985
518	1.000	0.959	0.979	0.960	0.960	0.980
519	1.000	0.969	0.985	0.970	0.970	0.985
520	1.000	0.948	0.974	0.950	0.951	0.975
521	1.000	0.990	0.995	0.990	0.990	0.995
522	1.000	0.948	0.974	0.950	0.951	0.975
523	1.000	0.948	0.974	0.950	0.951	0.975
524	1.000	0.948	0.974	0.950	0.951	0.975
525	1.000	0.948	0.974	0.950	0.951	0.975
526	1.000	0.969	0.985	0.970	0.970	0.985
527	1.000	0.948	0.974	0.950	0.951	0.975
528	1.000	0.969	0.985	0.970	0.970	0.985
529	1.000	0.969	0.985	0.970	0.970	0.985
530	1.000	0.959	0.979	0.960	0.960	0.980
531	1.000	0.969	0.985	0.970	0.970	0.985
532	1.000	0.959	0.979	0.960	0.960	0.980
533	1.000	0.979	0.990	0.980	0.980	0.990
534	1.000	0.938	0.969	0.940	0.942	0.970
535	1.000	0.959	0.979	0.960	0.960	0.980
536	1.000	0.959	0.979	0.960	0.960	0.980
537	1.000	0.959	0.979	0.960	0.960	0.980
538	1.000	0.969	0.985	0.970	0.970	0.985
539	1.000	0.948	0.974	0.950	0.951	0.975
540	1.000	0.948	0.974	0.950	0.951	0.975
541	1.000	0.969	0.985	0.970	0.970	0.985
542	1.000	0.938	0.969	0.940	0.942	0.970

543	1.000	0.959	0.979	0.960	0.960	0.980
544	1.000	0.948	0.974	0.950	0.951	0.975
545	1.000	0.969	0.985	0.970	0.970	0.985
546	1.000	0.948	0.974	0.950	0.951	0.975
547	1.000	0.959	0.979	0.960	0.960	0.980
548	1.000	0.938	0.969	0.940	0.942	0.970
549	0.990	0.948	0.969	0.939	0.950	0.970
550	1.000	0.969	0.985	0.970	0.970	0.985
551	1.000	0.948	0.974	0.950	0.951	0.975
552	1.000	0.948	0.974	0.950	0.951	0.975
553	1.000	0.969	0.985	0.970	0.970	0.985
554	1.000	0.959	0.979	0.960	0.960	0.980
555	1.000	0.948	0.974	0.950	0.951	0.975
556	1.000	0.969	0.985	0.970	0.970	0.985
557	1.000	0.959	0.979	0.960	0.960	0.980
558	1.000	0.959	0.979	0.960	0.960	0.980
559	1.000	0.969	0.985	0.970	0.970	0.985
560	1.000	0.938	0.969	0.940	0.942	0.970
561	1.000	0.959	0.979	0.960	0.960	0.980
562	1.000	0.959	0.979	0.960	0.960	0.980
563	1.000	0.969	0.985	0.970	0.970	0.985
564	1.000	0.948	0.974	0.950	0.951	0.975
565	1.000	0.948	0.974	0.950	0.951	0.975
566	1.000	0.959	0.979	0.960	0.960	0.980
567	1.000	0.979	0.990	0.980	0.980	0.990
568	1.000	0.979	0.990	0.980	0.980	0.990
569	1.000	0.969	0.985	0.970	0.970	0.985
570	1.000	0.959	0.979	0.960	0.960	0.980
571	1.000	0.969	0.985	0.970	0.970	0.985
572	1.000	0.948	0.974	0.950	0.951	0.975
573	1.000	0.959	0.979	0.960	0.960	0.980
574	1.000	0.969	0.985	0.970	0.970	0.985
575	1.000	0.969	0.985	0.970	0.970	0.985
576	1.000	0.959	0.979	0.960	0.960	0.980
577	1.000	0.959	0.979	0.960	0.960	0.980
578	1.000	0.969	0.985	0.970	0.970	0.985
579	1.000	0.969	0.985	0.970	0.970	0.985
580	1.000	0.969	0.985	0.970	0.970	0.985
581	1.000	0.959	0.979	0.960	0.960	0.980
582	1.000	0.959	0.979	0.960	0.960	0.980
583	1.000	0.948	0.974	0.950	0.951	0.975
584	1.000	0.948	0.974	0.950	0.951	0.975

585	1.000	0.959	0.979	0.960	0.960	0.980
586	1.000	0.948	0.974	0.950	0.951	0.975
587	1.000	0.948	0.974	0.950	0.951	0.975
588	1.000	0.938	0.969	0.940	0.942	0.970
589	1.000	0.969	0.985	0.970	0.970	0.985
590	1.000	0.948	0.974	0.950	0.951	0.975
591	1.000	0.959	0.979	0.960	0.960	0.980
592	1.000	0.948	0.974	0.950	0.951	0.975
593	1.000	0.959	0.979	0.960	0.960	0.980
594	1.000	0.959	0.979	0.960	0.960	0.980
595	1.000	0.959	0.979	0.960	0.960	0.980
596	1.000	0.948	0.974	0.950	0.951	0.975
597	1.000	0.948	0.974	0.950	0.951	0.975
598	1.000	0.959	0.979	0.960	0.960	0.980
599	1.000	0.959	0.979	0.960	0.960	0.980
600	1.000	0.948	0.974	0.950	0.951	0.975
601	1.000	0.938	0.969	0.940	0.942	0.970
602	1.000	0.969	0.985	0.970	0.970	0.985
603	1.000	0.969	0.985	0.970	0.970	0.985
604	1.000	0.959	0.979	0.960	0.960	0.980
605	1.000	0.979	0.990	0.980	0.980	0.990
606	1.000	0.969	0.985	0.970	0.970	0.985
607	1.000	0.959	0.979	0.960	0.960	0.980
608	1.000	0.959	0.979	0.960	0.960	0.980
609	1.000	0.948	0.974	0.950	0.951	0.975
610	1.000	0.948	0.974	0.950	0.951	0.975
611	1.000	0.959	0.979	0.960	0.960	0.980
612	1.000	0.979	0.990	0.980	0.980	0.990
613	1.000	0.959	0.979	0.960	0.960	0.980
614	1.000	0.948	0.974	0.950	0.951	0.975
615	1.000	0.959	0.979	0.960	0.960	0.980
616	1.000	0.969	0.985	0.970	0.970	0.985
617	1.000	0.959	0.979	0.960	0.960	0.980
618	1.000	0.969	0.985	0.970	0.970	0.985
619	1.000	0.959	0.979	0.960	0.960	0.980
620	1.000	0.979	0.990	0.980	0.980	0.990
621	1.000	0.959	0.979	0.960	0.960	0.980
622	1.000	0.959	0.979	0.960	0.960	0.980
623	1.000	0.979	0.990	0.980	0.980	0.990
624	1.000	0.938	0.969	0.940	0.942	0.970
625	1.000	0.959	0.979	0.960	0.960	0.980
626	1.000	0.959	0.979	0.960	0.960	0.980

627	1.000	0.969	0.985	0.970	0.970	0.985
628	1.000	0.959	0.979	0.960	0.960	0.980
629	1.000	0.959	0.979	0.960	0.960	0.980
630	1.000	0.969	0.985	0.970	0.970	0.985
631	1.000	0.969	0.985	0.970	0.970	0.985
632	1.000	0.959	0.979	0.960	0.960	0.980
633	1.000	0.959	0.979	0.960	0.960	0.980
634	1.000	0.948	0.974	0.950	0.951	0.975
635	1.000	0.948	0.974	0.950	0.951	0.975
636	1.000	0.948	0.974	0.950	0.951	0.975
637	1.000	0.969	0.985	0.970	0.970	0.985
638	1.000	0.948	0.974	0.950	0.951	0.975
639	1.000	0.948	0.974	0.950	0.951	0.975
640	1.000	0.969	0.985	0.970	0.970	0.985
641	1.000	0.969	0.985	0.970	0.970	0.985
642	1.000	0.948	0.974	0.950	0.951	0.975
643	1.000	0.959	0.979	0.960	0.960	0.980
644	1.000	0.948	0.974	0.950	0.951	0.975
645	1.000	0.959	0.979	0.960	0.960	0.980
646	1.000	0.948	0.974	0.950	0.951	0.975
647	1.000	0.948	0.974	0.950	0.951	0.975
648	1.000	0.948	0.974	0.950	0.951	0.975
649	1.000	0.938	0.969	0.940	0.942	0.970
650	1.000	0.979	0.990	0.980	0.980	0.990
651	1.000	0.948	0.974	0.950	0.951	0.975
652	1.000	0.948	0.974	0.950	0.951	0.975
653	1.000	0.948	0.974	0.950	0.951	0.975
654	1.000	0.969	0.985	0.970	0.970	0.985
655	1.000	0.969	0.985	0.970	0.970	0.985
656	1.000	0.969	0.985	0.970	0.970	0.985
657	1.000	0.948	0.974	0.950	0.951	0.975
658	1.000	0.959	0.979	0.960	0.960	0.980
659	1.000	0.979	0.990	0.980	0.980	0.990
660	1.000	0.938	0.969	0.940	0.942	0.970
661	1.000	0.969	0.985	0.970	0.970	0.985
662	1.000	0.969	0.985	0.970	0.970	0.985
663	1.000	0.948	0.974	0.950	0.951	0.975
664	1.000	0.969	0.985	0.970	0.970	0.985
665	1.000	0.959	0.979	0.960	0.960	0.980
666	1.000	0.938	0.969	0.940	0.942	0.970
667	1.000	0.959	0.979	0.960	0.960	0.980
668	1.000	0.938	0.969	0.940	0.942	0.970

669	1.000	0.948	0.974	0.950	0.951	0.975
670	1.000	0.948	0.974	0.950	0.951	0.975
671	1.000	0.979	0.990	0.980	0.980	0.990
672	1.000	0.948	0.974	0.950	0.951	0.975
673	1.000	0.959	0.979	0.960	0.960	0.980
674	1.000	0.959	0.979	0.960	0.960	0.980
675	1.000	0.969	0.985	0.970	0.970	0.985
676	1.000	0.959	0.979	0.960	0.960	0.980
677	1.000	0.948	0.974	0.950	0.951	0.975
678	1.000	0.948	0.974	0.950	0.951	0.975
679	1.000	0.959	0.979	0.960	0.960	0.980
680	1.000	0.969	0.985	0.970	0.970	0.985
681	1.000	0.969	0.985	0.970	0.970	0.985
682	1.000	0.969	0.985	0.970	0.970	0.985
683	1.000	0.969	0.985	0.970	0.970	0.985
684	1.000	0.959	0.979	0.960	0.960	0.980
685	1.000	0.969	0.985	0.970	0.970	0.985
686	1.000	0.959	0.979	0.960	0.960	0.980
687	1.000	0.969	0.985	0.970	0.970	0.985
688	1.000	0.959	0.979	0.960	0.960	0.980
689	1.000	0.948	0.974	0.950	0.951	0.975
690	1.000	0.959	0.979	0.960	0.960	0.980
691	1.000	0.959	0.979	0.960	0.960	0.980
692	1.000	0.969	0.985	0.970	0.970	0.985
693	1.000	0.959	0.979	0.960	0.960	0.980
694	1.000	0.969	0.985	0.970	0.970	0.985
695	1.000	0.959	0.979	0.960	0.960	0.980
696	1.000	0.969	0.985	0.970	0.970	0.985
697	1.000	0.969	0.985	0.970	0.970	0.985
698	1.000	0.959	0.979	0.960	0.960	0.980
699	1.000	0.948	0.974	0.950	0.951	0.975
700	1.000	0.959	0.979	0.960	0.960	0.980
701	1.000	0.979	0.990	0.980	0.980	0.990
702	1.000	0.959	0.979	0.960	0.960	0.980
703	1.000	0.969	0.985	0.970	0.970	0.985
704	1.000	0.969	0.985	0.970	0.970	0.985
705	1.000	0.948	0.974	0.950	0.951	0.975
706	1.000	0.979	0.990	0.980	0.980	0.990
707	1.000	0.959	0.979	0.960	0.960	0.980
708	1.000	0.948	0.974	0.950	0.951	0.975
709	1.000	0.948	0.974	0.950	0.951	0.975
710	1.000	0.969	0.985	0.970	0.970	0.985

711	1.000	0.969	0.985	0.970	0.970	0.985
712	1.000	0.948	0.974	0.950	0.951	0.975
713	1.000	0.948	0.974	0.950	0.951	0.975
714	1.000	0.959	0.979	0.960	0.960	0.980
715	1.000	0.969	0.985	0.970	0.970	0.985
716	1.000	0.959	0.979	0.960	0.960	0.980
717	1.000	0.938	0.969	0.940	0.942	0.970
718	1.000	0.969	0.985	0.970	0.970	0.985
719	1.000	0.959	0.979	0.960	0.960	0.980
720	1.000	0.938	0.969	0.940	0.942	0.970
721	1.000	0.959	0.979	0.960	0.960	0.980
722	1.000	0.948	0.974	0.950	0.951	0.975
723	1.000	0.959	0.979	0.960	0.960	0.980
724	1.000	0.979	0.990	0.980	0.980	0.990
725	1.000	0.969	0.985	0.970	0.970	0.985
726	1.000	0.948	0.974	0.950	0.951	0.975
727	1.000	0.959	0.979	0.960	0.960	0.980
728	1.000	0.959	0.979	0.960	0.960	0.980
729	1.000	0.979	0.990	0.980	0.980	0.990
730	1.000	0.959	0.979	0.960	0.960	0.980
731	1.000	0.979	0.990	0.980	0.980	0.990
732	1.000	0.969	0.985	0.970	0.970	0.985
733	1.000	0.959	0.979	0.960	0.960	0.980
734	1.000	0.969	0.985	0.970	0.970	0.985
735	1.000	0.969	0.985	0.970	0.970	0.985
736	1.000	0.959	0.979	0.960	0.960	0.980
737	1.000	0.948	0.974	0.950	0.951	0.975
738	1.000	0.969	0.985	0.970	0.970	0.985
739	1.000	0.948	0.974	0.950	0.951	0.975
740	1.000	0.969	0.985	0.970	0.970	0.985
741	1.000	0.979	0.990	0.980	0.980	0.990
742	1.000	0.959	0.979	0.960	0.960	0.980
743	1.000	0.959	0.979	0.960	0.960	0.980
744	1.000	0.938	0.969	0.940	0.942	0.970
745	1.000	0.948	0.974	0.950	0.951	0.975
746	1.000	0.948	0.974	0.950	0.951	0.975
747	1.000	0.969	0.985	0.970	0.970	0.985
748	1.000	0.948	0.974	0.950	0.951	0.975
749	1.000	0.959	0.979	0.960	0.960	0.980
750	0.990	0.959	0.974	0.949	0.960	0.975
751	1.000	0.969	0.985	0.970	0.970	0.985
752	1.000	0.959	0.979	0.960	0.960	0.980

753	1.000	0.948	0.974	0.950	0.951	0.975
754	1.000	0.979	0.990	0.980	0.980	0.990
755	1.000	0.979	0.990	0.980	0.980	0.990
756	1.000	0.959	0.979	0.960	0.960	0.980
757	1.000	0.969	0.985	0.970	0.970	0.985
758	1.000	0.979	0.990	0.980	0.980	0.990
759	1.000	0.959	0.979	0.960	0.960	0.980
760	1.000	0.969	0.985	0.970	0.970	0.985
761	1.000	0.948	0.974	0.950	0.951	0.975
762	1.000	0.959	0.979	0.960	0.960	0.980
763	1.000	0.969	0.985	0.970	0.970	0.985
764	1.000	0.979	0.990	0.980	0.980	0.990
765	1.000	0.969	0.985	0.970	0.970	0.985
766	1.000	0.948	0.974	0.950	0.951	0.975
767	1.000	0.948	0.974	0.950	0.951	0.975
768	1.000	0.948	0.974	0.950	0.951	0.975
769	1.000	0.959	0.979	0.960	0.960	0.980
770	1.000	0.969	0.985	0.970	0.970	0.985
771	1.000	0.959	0.979	0.960	0.960	0.980
772	1.000	0.979	0.990	0.980	0.980	0.990
773	1.000	0.969	0.985	0.970	0.970	0.985
774	1.000	0.959	0.979	0.960	0.960	0.980
775	1.000	0.948	0.974	0.950	0.951	0.975
776	1.000	0.948	0.974	0.950	0.951	0.975
777	0.990	0.948	0.969	0.939	0.950	0.970
778	1.000	0.959	0.979	0.960	0.960	0.980
779	1.000	0.938	0.969	0.940	0.942	0.970
780	1.000	0.969	0.985	0.970	0.970	0.985
781	1.000	0.969	0.985	0.970	0.970	0.985
782	1.000	0.948	0.974	0.950	0.951	0.975
783	1.000	0.969	0.985	0.970	0.970	0.985
784	1.000	0.959	0.979	0.960	0.960	0.980
785	1.000	0.969	0.985	0.970	0.970	0.985
786	1.000	0.959	0.979	0.960	0.960	0.980
787	1.000	0.969	0.985	0.970	0.970	0.985
788	1.000	0.969	0.985	0.970	0.970	0.985
789	1.000	0.959	0.979	0.960	0.960	0.980
790	1.000	0.979	0.990	0.980	0.980	0.990
791	1.000	0.969	0.985	0.970	0.970	0.985
792	1.000	0.959	0.979	0.960	0.960	0.980
793	1.000	0.979	0.990	0.980	0.980	0.990
794	1.000	0.948	0.974	0.950	0.951	0.975

795	1.000	0.948	0.974	0.950	0.951	0.975
796	1.000	0.969	0.985	0.970	0.970	0.985
797	1.000	0.969	0.985	0.970	0.970	0.985
798	1.000	0.979	0.990	0.980	0.980	0.990
799	1.000	0.969	0.985	0.970	0.970	0.985
800	1.000	0.969	0.985	0.970	0.970	0.985
801	1.000	0.959	0.979	0.960	0.960	0.980
802	1.000	0.959	0.979	0.960	0.960	0.980
803	1.000	0.959	0.979	0.960	0.960	0.980
804	1.000	0.959	0.979	0.960	0.960	0.980
805	1.000	0.959	0.979	0.960	0.960	0.980
806	1.000	0.948	0.974	0.950	0.951	0.975
807	1.000	0.969	0.985	0.970	0.970	0.985
808	1.000	0.969	0.985	0.970	0.970	0.985
809	1.000	0.959	0.979	0.960	0.960	0.980
810	1.000	0.969	0.985	0.970	0.970	0.985
811	1.000	0.959	0.979	0.960	0.960	0.980
812	1.000	0.969	0.985	0.970	0.970	0.985
813	1.000	0.948	0.974	0.950	0.951	0.975
814	1.000	0.948	0.974	0.950	0.951	0.975
815	1.000	0.990	0.995	0.990	0.990	0.995
816	1.000	0.959	0.979	0.960	0.960	0.980
817	1.000	0.959	0.979	0.960	0.960	0.980
818	1.000	0.948	0.974	0.950	0.951	0.975
819	1.000	0.969	0.985	0.970	0.970	0.985
820	1.000	0.969	0.985	0.970	0.970	0.985
821	1.000	0.979	0.990	0.980	0.980	0.990
822	1.000	0.959	0.979	0.960	0.960	0.980
823	1.000	0.948	0.974	0.950	0.951	0.975
824	1.000	0.969	0.985	0.970	0.970	0.985
825	1.000	0.959	0.979	0.960	0.960	0.980
826	1.000	0.969	0.985	0.970	0.970	0.985
827	1.000	0.948	0.974	0.950	0.951	0.975
828	1.000	0.948	0.974	0.950	0.951	0.975
829	1.000	0.959	0.979	0.960	0.960	0.980
830	1.000	0.959	0.979	0.960	0.960	0.980
831	1.000	0.959	0.979	0.960	0.960	0.980
832	1.000	0.959	0.979	0.960	0.960	0.980
833	1.000	0.948	0.974	0.950	0.951	0.975
834	1.000	0.948	0.974	0.950	0.951	0.975
835	1.000	0.969	0.985	0.970	0.970	0.985
836	1.000	0.969	0.985	0.970	0.970	0.985

837	1.000	0.969	0.985	0.970	0.970	0.985
838	1.000	0.959	0.979	0.960	0.960	0.980
839	1.000	0.969	0.985	0.970	0.970	0.985
840	1.000	0.959	0.979	0.960	0.960	0.980
841	1.000	0.979	0.990	0.980	0.980	0.990
842	1.000	0.959	0.979	0.960	0.960	0.980
843	1.000	0.959	0.979	0.960	0.960	0.980
844	1.000	0.979	0.990	0.980	0.980	0.990
845	1.000	0.969	0.985	0.970	0.970	0.985
846	1.000	0.948	0.974	0.950	0.951	0.975
847	1.000	0.959	0.979	0.960	0.960	0.980
848	1.000	0.969	0.985	0.970	0.970	0.985
849	1.000	0.969	0.985	0.970	0.970	0.985
850	1.000	0.938	0.969	0.940	0.942	0.970
851	1.000	0.948	0.974	0.950	0.951	0.975
852	1.000	0.959	0.979	0.960	0.960	0.980
853	1.000	0.979	0.990	0.980	0.980	0.990
854	1.000	0.959	0.979	0.960	0.960	0.980
855	1.000	0.948	0.974	0.950	0.951	0.975
856	1.000	0.979	0.990	0.980	0.980	0.990
857	1.000	0.969	0.985	0.970	0.970	0.985
858	1.000	0.948	0.974	0.950	0.951	0.975
859	1.000	0.979	0.990	0.980	0.980	0.990
860	1.000	0.969	0.985	0.970	0.970	0.985
861	1.000	0.948	0.974	0.950	0.951	0.975
862	1.000	0.948	0.974	0.950	0.951	0.975
863	1.000	0.969	0.985	0.970	0.970	0.985
864	1.000	0.938	0.969	0.940	0.942	0.970
865	1.000	0.959	0.979	0.960	0.960	0.980
866	1.000	0.938	0.969	0.940	0.942	0.970
867	1.000	0.948	0.974	0.950	0.951	0.975
868	1.000	0.969	0.985	0.970	0.970	0.985
869	1.000	0.948	0.974	0.950	0.951	0.975
870	1.000	0.959	0.979	0.960	0.960	0.980
871	1.000	0.948	0.974	0.950	0.951	0.975
872	1.000	0.979	0.990	0.980	0.980	0.990
873	1.000	0.979	0.990	0.980	0.980	0.990
874	1.000	0.948	0.974	0.950	0.951	0.975
875	1.000	0.969	0.985	0.970	0.970	0.985
876	1.000	0.969	0.985	0.970	0.970	0.985
877	1.000	0.959	0.979	0.960	0.960	0.980
878	1.000	0.959	0.979	0.960	0.960	0.980

879	1.000	0.969	0.985	0.970	0.970	0.985
880	1.000	0.979	0.990	0.980	0.980	0.990
881	1.000	0.969	0.985	0.970	0.970	0.985
882	1.000	0.948	0.974	0.950	0.951	0.975
883	1.000	0.969	0.985	0.970	0.970	0.985
884	1.000	0.959	0.979	0.960	0.960	0.980
885	1.000	0.969	0.985	0.970	0.970	0.985
886	1.000	0.969	0.985	0.970	0.970	0.985
887	1.000	0.979	0.990	0.980	0.980	0.990
888	1.000	0.938	0.969	0.940	0.942	0.970
889	1.000	0.979	0.990	0.980	0.980	0.990
890	1.000	0.990	0.995	0.990	0.990	0.995
891	1.000	0.959	0.979	0.960	0.960	0.980
892	1.000	0.938	0.969	0.940	0.942	0.970
893	1.000	0.969	0.985	0.970	0.970	0.985
894	1.000	0.938	0.969	0.940	0.942	0.970
895	1.000	0.959	0.979	0.960	0.960	0.980
896	1.000	0.948	0.974	0.950	0.951	0.975
897	1.000	0.959	0.979	0.960	0.960	0.980
898	1.000	0.959	0.979	0.960	0.960	0.980
899	1.000	0.969	0.985	0.970	0.970	0.985
900	1.000	0.948	0.974	0.950	0.951	0.975
901	1.000	0.979	0.990	0.980	0.980	0.990
902	1.000	0.938	0.969	0.940	0.942	0.970
903	1.000	0.969	0.985	0.970	0.970	0.985
904	1.000	0.979	0.990	0.980	0.980	0.990
905	1.000	0.969	0.985	0.970	0.970	0.985
906	1.000	0.969	0.985	0.970	0.970	0.985
907	1.000	0.969	0.985	0.970	0.970	0.985
908	1.000	0.959	0.979	0.960	0.960	0.980
909	1.000	0.979	0.990	0.980	0.980	0.990
910	1.000	0.948	0.974	0.950	0.951	0.975
911	1.000	0.959	0.979	0.960	0.960	0.980
912	1.000	0.969	0.985	0.970	0.970	0.985
913	1.000	0.979	0.990	0.980	0.980	0.990
914	1.000	0.969	0.985	0.970	0.970	0.985
915	1.000	0.969	0.985	0.970	0.970	0.985
916	1.000	0.959	0.979	0.960	0.960	0.980
917	1.000	0.959	0.979	0.960	0.960	0.980
918	1.000	0.969	0.985	0.970	0.970	0.985
919	1.000	0.969	0.985	0.970	0.970	0.985
920	1.000	0.969	0.985	0.970	0.970	0.985

921	1.000	0.948	0.974	0.950	0.951	0.975
922	1.000	0.979	0.990	0.980	0.980	0.990
923	1.000	0.979	0.990	0.980	0.980	0.990
924	1.000	0.969	0.985	0.970	0.970	0.985
925	1.000	0.948	0.974	0.950	0.951	0.975
926	1.000	0.948	0.974	0.950	0.951	0.975
927	1.000	0.969	0.985	0.970	0.970	0.985
928	1.000	0.969	0.985	0.970	0.970	0.985
929	1.000	0.990	0.995	0.990	0.990	0.995
930	1.000	0.979	0.990	0.980	0.980	0.990
931	1.000	0.990	0.995	0.990	0.990	0.995
932	1.000	0.990	0.995	0.990	0.990	0.995
933	1.000	0.969	0.985	0.970	0.970	0.985
934	1.000	0.948	0.974	0.950	0.951	0.975
935	1.000	0.969	0.985	0.970	0.970	0.985
936	1.000	0.959	0.979	0.960	0.960	0.980
937	1.000	0.979	0.990	0.980	0.980	0.990
938	1.000	0.938	0.969	0.940	0.942	0.970
939	1.000	0.969	0.985	0.970	0.970	0.985
940	1.000	0.959	0.979	0.960	0.960	0.980
941	1.000	0.948	0.974	0.950	0.951	0.975
942	1.000	0.948	0.974	0.950	0.951	0.975
943	1.000	0.969	0.985	0.970	0.970	0.985
944	1.000	0.948	0.974	0.950	0.951	0.975
945	1.000	0.948	0.974	0.950	0.951	0.975
946	1.000	0.948	0.974	0.950	0.951	0.975
947	0.990	0.948	0.969	0.939	0.950	0.970
948	1.000	0.959	0.979	0.960	0.960	0.980
949	1.000	0.948	0.974	0.950	0.951	0.975
950	1.000	0.938	0.969	0.940	0.942	0.970
951	1.000	0.969	0.985	0.970	0.970	0.985
952	1.000	0.979	0.990	0.980	0.980	0.990
953	1.000	0.959	0.979	0.960	0.960	0.980
954	1.000	0.959	0.979	0.960	0.960	0.980
955	1.000	0.979	0.990	0.980	0.980	0.990
956	1.000	0.948	0.974	0.950	0.951	0.975
957	1.000	0.959	0.979	0.960	0.960	0.980
958	1.000	0.969	0.985	0.970	0.970	0.985
959	1.000	0.948	0.974	0.950	0.951	0.975
960	1.000	0.959	0.979	0.960	0.960	0.980
961	1.000	0.959	0.979	0.960	0.960	0.980
962	1.000	0.948	0.974	0.950	0.951	0.975

963	1.000	0.948	0.974	0.950	0.951	0.975
964	1.000	0.979	0.990	0.980	0.980	0.990
965	1.000	0.969	0.985	0.970	0.970	0.985
966	1.000	0.990	0.995	0.990	0.990	0.995
967	1.000	0.979	0.990	0.980	0.980	0.990
968	1.000	0.969	0.985	0.970	0.970	0.985
969	1.000	0.979	0.990	0.980	0.980	0.990
970	1.000	0.969	0.985	0.970	0.970	0.985
971	1.000	0.959	0.979	0.960	0.960	0.980
972	0.990	0.969	0.979	0.959	0.970	0.980
973	1.000	0.948	0.974	0.950	0.951	0.975
974	1.000	0.969	0.985	0.970	0.970	0.985
975	1.000	0.959	0.979	0.960	0.960	0.980
976	1.000	0.969	0.985	0.970	0.970	0.985
977	1.000	0.948	0.974	0.950	0.951	0.975
978	1.000	0.959	0.979	0.960	0.960	0.980
979	1.000	0.969	0.985	0.970	0.970	0.985
980	1.000	0.959	0.979	0.960	0.960	0.980
981	1.000	0.979	0.990	0.980	0.980	0.990
982	0.990	0.969	0.979	0.959	0.970	0.980
983	1.000	0.959	0.979	0.960	0.960	0.980
984	1.000	0.969	0.985	0.970	0.970	0.985
985	1.000	0.948	0.974	0.950	0.951	0.975
986	1.000	0.959	0.979	0.960	0.960	0.980
987	1.000	0.938	0.969	0.940	0.942	0.970
988	1.000	0.959	0.979	0.960	0.960	0.980
989	1.000	0.979	0.990	0.980	0.980	0.990
990	1.000	0.979	0.990	0.980	0.980	0.990
991	1.000	0.948	0.974	0.950	0.951	0.975
992	1.000	0.979	0.990	0.980	0.980	0.990
993	1.000	0.938	0.969	0.940	0.942	0.970
994	1.000	0.969	0.985	0.970	0.970	0.985
995	1.000	0.959	0.979	0.960	0.960	0.980
996	1.000	0.969	0.985	0.970	0.970	0.985
997	1.000	0.948	0.974	0.950	0.951	0.975
998	1.000	0.979	0.990	0.980	0.980	0.990
999	1.000	0.959	0.979	0.960	0.960	0.980
1000	1.000	0.948	0.974	0.950	0.951	0.975
1001	1.000	0.969	0.985	0.970	0.970	0.985
1002	1.000	0.969	0.985	0.970	0.970	0.985
1003	1.000	0.979	0.990	0.980	0.980	0.990
1004	1.000	0.948	0.974	0.950	0.951	0.975

1005	1.000	0.969	0.985	0.970	0.970	0.985
1006	1.000	0.959	0.979	0.960	0.960	0.980
1007	1.000	0.948	0.974	0.950	0.951	0.975
1008	1.000	0.959	0.979	0.960	0.960	0.980
1009	1.000	0.969	0.985	0.970	0.970	0.985
1010	1.000	0.969	0.985	0.970	0.970	0.985
1011	1.000	0.979	0.990	0.980	0.980	0.990
1012	1.000	0.969	0.985	0.970	0.970	0.985
1013	1.000	0.979	0.990	0.980	0.980	0.990
1014	1.000	0.959	0.979	0.960	0.960	0.980
1015	1.000	0.979	0.990	0.980	0.980	0.990
1016	1.000	0.948	0.974	0.950	0.951	0.975
1017	1.000	0.948	0.974	0.950	0.951	0.975
1018	1.000	0.969	0.985	0.970	0.970	0.985
1019	1.000	0.979	0.990	0.980	0.980	0.990
1020	1.000	0.959	0.979	0.960	0.960	0.980

(2) K-nearest neighbor

Number of features	SN	SP	ACC	MCC	Precision	F1-measure
1	1.000	0.938	0.969	0.940	0.942	0.970
2	1.000	0.990	0.995	0.990	0.990	0.995
3	1.000	1.000	1.000	1.000	1.000	1.000
4	1.000	1.000	1.000	1.000	1.000	1.000
5	1.000	1.000	1.000	1.000	1.000	1.000
6	1.000	1.000	1.000	1.000	1.000	1.000
7	1.000	1.000	1.000	1.000	1.000	1.000
8	1.000	1.000	1.000	1.000	1.000	1.000
9	1.000	1.000	1.000	1.000	1.000	1.000
10	1.000	1.000	1.000	1.000	1.000	1.000
11	1.000	1.000	1.000	1.000	1.000	1.000
12	1.000	1.000	1.000	1.000	1.000	1.000
13	1.000	1.000	1.000	1.000	1.000	1.000
14	1.000	1.000	1.000	1.000	1.000	1.000
15	1.000	1.000	1.000	1.000	1.000	1.000
16	1.000	1.000	1.000	1.000	1.000	1.000
17	1.000	1.000	1.000	1.000	1.000	1.000
18	1.000	1.000	1.000	1.000	1.000	1.000
19	1.000	0.990	0.995	0.990	0.990	0.995
20	1.000	0.990	0.995	0.990	0.990	0.995
21	1.000	0.990	0.995	0.990	0.990	0.995
22	1.000	0.990	0.995	0.990	0.990	0.995
23	1.000	0.990	0.995	0.990	0.990	0.995

24	1.000	0.990	0.995	0.990	0.990	0.995
25	1.000	0.990	0.995	0.990	0.990	0.995
26	1.000	1.000	1.000	1.000	1.000	1.000
27	1.000	1.000	1.000	1.000	1.000	1.000
28	1.000	1.000	1.000	1.000	1.000	1.000
29	1.000	1.000	1.000	1.000	1.000	1.000
30	1.000	1.000	1.000	1.000	1.000	1.000
31	1.000	1.000	1.000	1.000	1.000	1.000
32	1.000	1.000	1.000	1.000	1.000	1.000
33	1.000	1.000	1.000	1.000	1.000	1.000
34	1.000	1.000	1.000	1.000	1.000	1.000
35	1.000	1.000	1.000	1.000	1.000	1.000
36	1.000	1.000	1.000	1.000	1.000	1.000
37	1.000	1.000	1.000	1.000	1.000	1.000
38	1.000	1.000	1.000	1.000	1.000	1.000
39	1.000	1.000	1.000	1.000	1.000	1.000
40	1.000	1.000	1.000	1.000	1.000	1.000
41	1.000	1.000	1.000	1.000	1.000	1.000
42	1.000	1.000	1.000	1.000	1.000	1.000
43	1.000	1.000	1.000	1.000	1.000	1.000
44	1.000	1.000	1.000	1.000	1.000	1.000
45	1.000	1.000	1.000	1.000	1.000	1.000
46	1.000	1.000	1.000	1.000	1.000	1.000
47	1.000	1.000	1.000	1.000	1.000	1.000
48	1.000	1.000	1.000	1.000	1.000	1.000
49	1.000	1.000	1.000	1.000	1.000	1.000
50	1.000	1.000	1.000	1.000	1.000	1.000
51	1.000	1.000	1.000	1.000	1.000	1.000
52	1.000	1.000	1.000	1.000	1.000	1.000
53	1.000	1.000	1.000	1.000	1.000	1.000
54	1.000	1.000	1.000	1.000	1.000	1.000
55	1.000	1.000	1.000	1.000	1.000	1.000
56	1.000	1.000	1.000	1.000	1.000	1.000
57	1.000	1.000	1.000	1.000	1.000	1.000
58	1.000	1.000	1.000	1.000	1.000	1.000
59	1.000	1.000	1.000	1.000	1.000	1.000
60	1.000	1.000	1.000	1.000	1.000	1.000
61	1.000	1.000	1.000	1.000	1.000	1.000
62	1.000	1.000	1.000	1.000	1.000	1.000
63	1.000	1.000	1.000	1.000	1.000	1.000
64	1.000	1.000	1.000	1.000	1.000	1.000
65	1.000	1.000	1.000	1.000	1.000	1.000

66	1.000	1.000	1.000	1.000	1.000	1.000
67	1.000	1.000	1.000	1.000	1.000	1.000
68	1.000	1.000	1.000	1.000	1.000	1.000
69	1.000	1.000	1.000	1.000	1.000	1.000
70	1.000	1.000	1.000	1.000	1.000	1.000
71	1.000	1.000	1.000	1.000	1.000	1.000
72	1.000	1.000	1.000	1.000	1.000	1.000
73	1.000	1.000	1.000	1.000	1.000	1.000
74	1.000	1.000	1.000	1.000	1.000	1.000
75	1.000	1.000	1.000	1.000	1.000	1.000
76	1.000	1.000	1.000	1.000	1.000	1.000
77	1.000	1.000	1.000	1.000	1.000	1.000
78	1.000	1.000	1.000	1.000	1.000	1.000
79	1.000	1.000	1.000	1.000	1.000	1.000
80	1.000	1.000	1.000	1.000	1.000	1.000
81	1.000	1.000	1.000	1.000	1.000	1.000
82	1.000	1.000	1.000	1.000	1.000	1.000
83	1.000	1.000	1.000	1.000	1.000	1.000
84	1.000	1.000	1.000	1.000	1.000	1.000
85	1.000	1.000	1.000	1.000	1.000	1.000
86	1.000	1.000	1.000	1.000	1.000	1.000
87	1.000	1.000	1.000	1.000	1.000	1.000
88	1.000	1.000	1.000	1.000	1.000	1.000
89	1.000	1.000	1.000	1.000	1.000	1.000
90	1.000	1.000	1.000	1.000	1.000	1.000
91	1.000	1.000	1.000	1.000	1.000	1.000
92	1.000	1.000	1.000	1.000	1.000	1.000
93	1.000	1.000	1.000	1.000	1.000	1.000
94	1.000	1.000	1.000	1.000	1.000	1.000
95	1.000	1.000	1.000	1.000	1.000	1.000
96	1.000	1.000	1.000	1.000	1.000	1.000
97	1.000	1.000	1.000	1.000	1.000	1.000
98	1.000	1.000	1.000	1.000	1.000	1.000
99	1.000	1.000	1.000	1.000	1.000	1.000
100	1.000	1.000	1.000	1.000	1.000	1.000
101	1.000	1.000	1.000	1.000	1.000	1.000
102	1.000	1.000	1.000	1.000	1.000	1.000
103	1.000	1.000	1.000	1.000	1.000	1.000
104	1.000	1.000	1.000	1.000	1.000	1.000
105	1.000	1.000	1.000	1.000	1.000	1.000
106	1.000	1.000	1.000	1.000	1.000	1.000
107	1.000	1.000	1.000	1.000	1.000	1.000

108	1.000	1.000	1.000	1.000	1.000	1.000
109	1.000	1.000	1.000	1.000	1.000	1.000
110	1.000	1.000	1.000	1.000	1.000	1.000
111	1.000	1.000	1.000	1.000	1.000	1.000
112	1.000	1.000	1.000	1.000	1.000	1.000
113	1.000	1.000	1.000	1.000	1.000	1.000
114	1.000	1.000	1.000	1.000	1.000	1.000
115	1.000	1.000	1.000	1.000	1.000	1.000
116	1.000	1.000	1.000	1.000	1.000	1.000
117	1.000	1.000	1.000	1.000	1.000	1.000
118	1.000	1.000	1.000	1.000	1.000	1.000
119	1.000	1.000	1.000	1.000	1.000	1.000
120	1.000	1.000	1.000	1.000	1.000	1.000
121	1.000	1.000	1.000	1.000	1.000	1.000
122	1.000	1.000	1.000	1.000	1.000	1.000
123	1.000	1.000	1.000	1.000	1.000	1.000
124	1.000	1.000	1.000	1.000	1.000	1.000
125	1.000	1.000	1.000	1.000	1.000	1.000
126	1.000	1.000	1.000	1.000	1.000	1.000
127	1.000	1.000	1.000	1.000	1.000	1.000
128	1.000	1.000	1.000	1.000	1.000	1.000
129	1.000	1.000	1.000	1.000	1.000	1.000
130	1.000	1.000	1.000	1.000	1.000	1.000
131	1.000	1.000	1.000	1.000	1.000	1.000
132	1.000	1.000	1.000	1.000	1.000	1.000
133	1.000	1.000	1.000	1.000	1.000	1.000
134	1.000	1.000	1.000	1.000	1.000	1.000
135	1.000	1.000	1.000	1.000	1.000	1.000
136	1.000	1.000	1.000	1.000	1.000	1.000
137	1.000	1.000	1.000	1.000	1.000	1.000
138	1.000	1.000	1.000	1.000	1.000	1.000
139	1.000	1.000	1.000	1.000	1.000	1.000
140	1.000	1.000	1.000	1.000	1.000	1.000
141	1.000	1.000	1.000	1.000	1.000	1.000
142	1.000	1.000	1.000	1.000	1.000	1.000
143	1.000	1.000	1.000	1.000	1.000	1.000
144	1.000	1.000	1.000	1.000	1.000	1.000
145	1.000	1.000	1.000	1.000	1.000	1.000
146	1.000	1.000	1.000	1.000	1.000	1.000
147	1.000	1.000	1.000	1.000	1.000	1.000
148	1.000	1.000	1.000	1.000	1.000	1.000
149	1.000	1.000	1.000	1.000	1.000	1.000

150	1.000	1.000	1.000	1.000	1.000	1.000
151	1.000	1.000	1.000	1.000	1.000	1.000
152	1.000	1.000	1.000	1.000	1.000	1.000
153	1.000	1.000	1.000	1.000	1.000	1.000
154	1.000	1.000	1.000	1.000	1.000	1.000
155	1.000	1.000	1.000	1.000	1.000	1.000
156	1.000	1.000	1.000	1.000	1.000	1.000
157	1.000	1.000	1.000	1.000	1.000	1.000
158	1.000	1.000	1.000	1.000	1.000	1.000
159	1.000	1.000	1.000	1.000	1.000	1.000
160	1.000	1.000	1.000	1.000	1.000	1.000
161	1.000	1.000	1.000	1.000	1.000	1.000
162	1.000	1.000	1.000	1.000	1.000	1.000
163	1.000	1.000	1.000	1.000	1.000	1.000
164	1.000	1.000	1.000	1.000	1.000	1.000
165	1.000	1.000	1.000	1.000	1.000	1.000
166	1.000	1.000	1.000	1.000	1.000	1.000
167	1.000	1.000	1.000	1.000	1.000	1.000
168	1.000	1.000	1.000	1.000	1.000	1.000
169	1.000	1.000	1.000	1.000	1.000	1.000
170	1.000	1.000	1.000	1.000	1.000	1.000
171	1.000	1.000	1.000	1.000	1.000	1.000
172	1.000	1.000	1.000	1.000	1.000	1.000
173	1.000	1.000	1.000	1.000	1.000	1.000
174	1.000	1.000	1.000	1.000	1.000	1.000
175	1.000	1.000	1.000	1.000	1.000	1.000
176	1.000	1.000	1.000	1.000	1.000	1.000
177	1.000	1.000	1.000	1.000	1.000	1.000
178	1.000	1.000	1.000	1.000	1.000	1.000
179	1.000	1.000	1.000	1.000	1.000	1.000
180	1.000	1.000	1.000	1.000	1.000	1.000
181	1.000	1.000	1.000	1.000	1.000	1.000
182	1.000	1.000	1.000	1.000	1.000	1.000
183	1.000	1.000	1.000	1.000	1.000	1.000
184	1.000	1.000	1.000	1.000	1.000	1.000
185	1.000	1.000	1.000	1.000	1.000	1.000
186	1.000	1.000	1.000	1.000	1.000	1.000
187	1.000	1.000	1.000	1.000	1.000	1.000
188	1.000	1.000	1.000	1.000	1.000	1.000
189	1.000	1.000	1.000	1.000	1.000	1.000
190	1.000	1.000	1.000	1.000	1.000	1.000
191	1.000	1.000	1.000	1.000	1.000	1.000

192	1.000	1.000	1.000	1.000	1.000	1.000
193	1.000	1.000	1.000	1.000	1.000	1.000
194	1.000	1.000	1.000	1.000	1.000	1.000
195	1.000	1.000	1.000	1.000	1.000	1.000
196	1.000	1.000	1.000	1.000	1.000	1.000
197	1.000	1.000	1.000	1.000	1.000	1.000
198	1.000	1.000	1.000	1.000	1.000	1.000
199	1.000	1.000	1.000	1.000	1.000	1.000
200	1.000	1.000	1.000	1.000	1.000	1.000
201	1.000	1.000	1.000	1.000	1.000	1.000
202	1.000	1.000	1.000	1.000	1.000	1.000
203	1.000	1.000	1.000	1.000	1.000	1.000
204	1.000	1.000	1.000	1.000	1.000	1.000
205	1.000	1.000	1.000	1.000	1.000	1.000
206	1.000	1.000	1.000	1.000	1.000	1.000
207	1.000	1.000	1.000	1.000	1.000	1.000
208	1.000	1.000	1.000	1.000	1.000	1.000
209	1.000	1.000	1.000	1.000	1.000	1.000
210	1.000	1.000	1.000	1.000	1.000	1.000
211	1.000	1.000	1.000	1.000	1.000	1.000
212	1.000	1.000	1.000	1.000	1.000	1.000
213	1.000	1.000	1.000	1.000	1.000	1.000
214	1.000	1.000	1.000	1.000	1.000	1.000
215	1.000	1.000	1.000	1.000	1.000	1.000
216	1.000	1.000	1.000	1.000	1.000	1.000
217	1.000	1.000	1.000	1.000	1.000	1.000
218	1.000	1.000	1.000	1.000	1.000	1.000
219	1.000	1.000	1.000	1.000	1.000	1.000
220	1.000	1.000	1.000	1.000	1.000	1.000
221	1.000	1.000	1.000	1.000	1.000	1.000
222	1.000	1.000	1.000	1.000	1.000	1.000
223	1.000	1.000	1.000	1.000	1.000	1.000
224	1.000	1.000	1.000	1.000	1.000	1.000
225	1.000	1.000	1.000	1.000	1.000	1.000
226	1.000	1.000	1.000	1.000	1.000	1.000
227	1.000	1.000	1.000	1.000	1.000	1.000
228	1.000	1.000	1.000	1.000	1.000	1.000
229	1.000	1.000	1.000	1.000	1.000	1.000
230	1.000	1.000	1.000	1.000	1.000	1.000
231	1.000	1.000	1.000	1.000	1.000	1.000
232	1.000	1.000	1.000	1.000	1.000	1.000
233	1.000	1.000	1.000	1.000	1.000	1.000

234	1.000	1.000	1.000	1.000	1.000	1.000
235	1.000	1.000	1.000	1.000	1.000	1.000
236	1.000	1.000	1.000	1.000	1.000	1.000
237	1.000	1.000	1.000	1.000	1.000	1.000
238	1.000	1.000	1.000	1.000	1.000	1.000
239	1.000	1.000	1.000	1.000	1.000	1.000
240	1.000	1.000	1.000	1.000	1.000	1.000
241	1.000	1.000	1.000	1.000	1.000	1.000
242	1.000	1.000	1.000	1.000	1.000	1.000
243	1.000	1.000	1.000	1.000	1.000	1.000
244	1.000	1.000	1.000	1.000	1.000	1.000
245	1.000	1.000	1.000	1.000	1.000	1.000
246	1.000	1.000	1.000	1.000	1.000	1.000
247	1.000	1.000	1.000	1.000	1.000	1.000
248	1.000	1.000	1.000	1.000	1.000	1.000
249	1.000	1.000	1.000	1.000	1.000	1.000
250	1.000	1.000	1.000	1.000	1.000	1.000
251	1.000	1.000	1.000	1.000	1.000	1.000
252	1.000	1.000	1.000	1.000	1.000	1.000
253	1.000	1.000	1.000	1.000	1.000	1.000
254	1.000	1.000	1.000	1.000	1.000	1.000
255	1.000	1.000	1.000	1.000	1.000	1.000
256	1.000	1.000	1.000	1.000	1.000	1.000
257	1.000	1.000	1.000	1.000	1.000	1.000
258	1.000	1.000	1.000	1.000	1.000	1.000
259	1.000	1.000	1.000	1.000	1.000	1.000
260	1.000	1.000	1.000	1.000	1.000	1.000
261	1.000	1.000	1.000	1.000	1.000	1.000
262	1.000	1.000	1.000	1.000	1.000	1.000
263	1.000	1.000	1.000	1.000	1.000	1.000
264	1.000	1.000	1.000	1.000	1.000	1.000
265	1.000	1.000	1.000	1.000	1.000	1.000
266	1.000	1.000	1.000	1.000	1.000	1.000
267	1.000	1.000	1.000	1.000	1.000	1.000
268	1.000	1.000	1.000	1.000	1.000	1.000
269	1.000	1.000	1.000	1.000	1.000	1.000
270	1.000	1.000	1.000	1.000	1.000	1.000
271	1.000	1.000	1.000	1.000	1.000	1.000
272	1.000	1.000	1.000	1.000	1.000	1.000
273	1.000	1.000	1.000	1.000	1.000	1.000
274	1.000	1.000	1.000	1.000	1.000	1.000
275	1.000	1.000	1.000	1.000	1.000	1.000

276	1.000	1.000	1.000	1.000	1.000	1.000
277	1.000	1.000	1.000	1.000	1.000	1.000
278	1.000	1.000	1.000	1.000	1.000	1.000
279	1.000	1.000	1.000	1.000	1.000	1.000
280	1.000	1.000	1.000	1.000	1.000	1.000
281	1.000	1.000	1.000	1.000	1.000	1.000
282	1.000	1.000	1.000	1.000	1.000	1.000
283	1.000	1.000	1.000	1.000	1.000	1.000
284	1.000	1.000	1.000	1.000	1.000	1.000
285	1.000	1.000	1.000	1.000	1.000	1.000
286	1.000	1.000	1.000	1.000	1.000	1.000
287	1.000	1.000	1.000	1.000	1.000	1.000
288	1.000	1.000	1.000	1.000	1.000	1.000
289	1.000	1.000	1.000	1.000	1.000	1.000
290	1.000	1.000	1.000	1.000	1.000	1.000
291	1.000	1.000	1.000	1.000	1.000	1.000
292	1.000	1.000	1.000	1.000	1.000	1.000
293	1.000	1.000	1.000	1.000	1.000	1.000
294	1.000	1.000	1.000	1.000	1.000	1.000
295	1.000	1.000	1.000	1.000	1.000	1.000
296	1.000	1.000	1.000	1.000	1.000	1.000
297	1.000	1.000	1.000	1.000	1.000	1.000
298	1.000	1.000	1.000	1.000	1.000	1.000
299	1.000	1.000	1.000	1.000	1.000	1.000
300	1.000	1.000	1.000	1.000	1.000	1.000
301	1.000	1.000	1.000	1.000	1.000	1.000
302	1.000	1.000	1.000	1.000	1.000	1.000
303	1.000	1.000	1.000	1.000	1.000	1.000
304	1.000	1.000	1.000	1.000	1.000	1.000
305	1.000	1.000	1.000	1.000	1.000	1.000
306	1.000	1.000	1.000	1.000	1.000	1.000
307	1.000	1.000	1.000	1.000	1.000	1.000
308	1.000	1.000	1.000	1.000	1.000	1.000
309	1.000	1.000	1.000	1.000	1.000	1.000
310	1.000	1.000	1.000	1.000	1.000	1.000
311	1.000	1.000	1.000	1.000	1.000	1.000
312	1.000	1.000	1.000	1.000	1.000	1.000
313	1.000	1.000	1.000	1.000	1.000	1.000
314	1.000	1.000	1.000	1.000	1.000	1.000
315	1.000	1.000	1.000	1.000	1.000	1.000
316	1.000	1.000	1.000	1.000	1.000	1.000
317	1.000	1.000	1.000	1.000	1.000	1.000

318	1.000	1.000	1.000	1.000	1.000	1.000
319	1.000	1.000	1.000	1.000	1.000	1.000
320	1.000	1.000	1.000	1.000	1.000	1.000
321	1.000	1.000	1.000	1.000	1.000	1.000
322	1.000	1.000	1.000	1.000	1.000	1.000
323	1.000	1.000	1.000	1.000	1.000	1.000
324	1.000	1.000	1.000	1.000	1.000	1.000
325	1.000	1.000	1.000	1.000	1.000	1.000
326	1.000	1.000	1.000	1.000	1.000	1.000
327	1.000	1.000	1.000	1.000	1.000	1.000
328	1.000	1.000	1.000	1.000	1.000	1.000
329	1.000	1.000	1.000	1.000	1.000	1.000
330	1.000	1.000	1.000	1.000	1.000	1.000
331	1.000	1.000	1.000	1.000	1.000	1.000
332	1.000	1.000	1.000	1.000	1.000	1.000
333	1.000	1.000	1.000	1.000	1.000	1.000
334	1.000	1.000	1.000	1.000	1.000	1.000
335	1.000	1.000	1.000	1.000	1.000	1.000
336	1.000	1.000	1.000	1.000	1.000	1.000
337	1.000	1.000	1.000	1.000	1.000	1.000
338	1.000	1.000	1.000	1.000	1.000	1.000
339	1.000	1.000	1.000	1.000	1.000	1.000
340	1.000	1.000	1.000	1.000	1.000	1.000
341	1.000	1.000	1.000	1.000	1.000	1.000
342	1.000	1.000	1.000	1.000	1.000	1.000
343	1.000	1.000	1.000	1.000	1.000	1.000
344	1.000	1.000	1.000	1.000	1.000	1.000
345	1.000	1.000	1.000	1.000	1.000	1.000
346	1.000	1.000	1.000	1.000	1.000	1.000
347	1.000	1.000	1.000	1.000	1.000	1.000
348	1.000	1.000	1.000	1.000	1.000	1.000
349	1.000	1.000	1.000	1.000	1.000	1.000
350	1.000	1.000	1.000	1.000	1.000	1.000
351	1.000	1.000	1.000	1.000	1.000	1.000
352	1.000	1.000	1.000	1.000	1.000	1.000
353	1.000	1.000	1.000	1.000	1.000	1.000
354	1.000	1.000	1.000	1.000	1.000	1.000
355	1.000	1.000	1.000	1.000	1.000	1.000
356	1.000	1.000	1.000	1.000	1.000	1.000
357	1.000	1.000	1.000	1.000	1.000	1.000
358	1.000	1.000	1.000	1.000	1.000	1.000
359	1.000	1.000	1.000	1.000	1.000	1.000

360	1.000	1.000	1.000	1.000	1.000	1.000
361	1.000	1.000	1.000	1.000	1.000	1.000
362	1.000	1.000	1.000	1.000	1.000	1.000
363	1.000	1.000	1.000	1.000	1.000	1.000
364	1.000	1.000	1.000	1.000	1.000	1.000
365	1.000	1.000	1.000	1.000	1.000	1.000
366	1.000	1.000	1.000	1.000	1.000	1.000
367	1.000	1.000	1.000	1.000	1.000	1.000
368	1.000	1.000	1.000	1.000	1.000	1.000
369	1.000	1.000	1.000	1.000	1.000	1.000
370	1.000	1.000	1.000	1.000	1.000	1.000
371	1.000	1.000	1.000	1.000	1.000	1.000
372	1.000	1.000	1.000	1.000	1.000	1.000
373	1.000	1.000	1.000	1.000	1.000	1.000
374	1.000	1.000	1.000	1.000	1.000	1.000
375	1.000	1.000	1.000	1.000	1.000	1.000
376	1.000	1.000	1.000	1.000	1.000	1.000
377	1.000	1.000	1.000	1.000	1.000	1.000
378	1.000	1.000	1.000	1.000	1.000	1.000
379	1.000	1.000	1.000	1.000	1.000	1.000
380	1.000	1.000	1.000	1.000	1.000	1.000
381	1.000	1.000	1.000	1.000	1.000	1.000
382	1.000	1.000	1.000	1.000	1.000	1.000
383	1.000	1.000	1.000	1.000	1.000	1.000
384	1.000	1.000	1.000	1.000	1.000	1.000
385	1.000	1.000	1.000	1.000	1.000	1.000
386	1.000	1.000	1.000	1.000	1.000	1.000
387	1.000	1.000	1.000	1.000	1.000	1.000
388	1.000	1.000	1.000	1.000	1.000	1.000
389	1.000	1.000	1.000	1.000	1.000	1.000
390	1.000	1.000	1.000	1.000	1.000	1.000
391	1.000	1.000	1.000	1.000	1.000	1.000
392	1.000	1.000	1.000	1.000	1.000	1.000
393	1.000	1.000	1.000	1.000	1.000	1.000
394	1.000	1.000	1.000	1.000	1.000	1.000
395	1.000	1.000	1.000	1.000	1.000	1.000
396	1.000	1.000	1.000	1.000	1.000	1.000
397	1.000	1.000	1.000	1.000	1.000	1.000
398	1.000	1.000	1.000	1.000	1.000	1.000
399	1.000	1.000	1.000	1.000	1.000	1.000
400	1.000	1.000	1.000	1.000	1.000	1.000
401	1.000	1.000	1.000	1.000	1.000	1.000

402	1.000	1.000	1.000	1.000	1.000	1.000
403	1.000	1.000	1.000	1.000	1.000	1.000
404	1.000	1.000	1.000	1.000	1.000	1.000
405	1.000	1.000	1.000	1.000	1.000	1.000
406	1.000	1.000	1.000	1.000	1.000	1.000
407	1.000	1.000	1.000	1.000	1.000	1.000
408	1.000	1.000	1.000	1.000	1.000	1.000
409	1.000	1.000	1.000	1.000	1.000	1.000
410	1.000	1.000	1.000	1.000	1.000	1.000
411	1.000	1.000	1.000	1.000	1.000	1.000
412	1.000	1.000	1.000	1.000	1.000	1.000
413	1.000	1.000	1.000	1.000	1.000	1.000
414	1.000	1.000	1.000	1.000	1.000	1.000
415	1.000	1.000	1.000	1.000	1.000	1.000
416	1.000	1.000	1.000	1.000	1.000	1.000
417	1.000	1.000	1.000	1.000	1.000	1.000
418	1.000	1.000	1.000	1.000	1.000	1.000
419	1.000	1.000	1.000	1.000	1.000	1.000
420	1.000	1.000	1.000	1.000	1.000	1.000
421	1.000	1.000	1.000	1.000	1.000	1.000
422	1.000	1.000	1.000	1.000	1.000	1.000
423	1.000	1.000	1.000	1.000	1.000	1.000
424	1.000	1.000	1.000	1.000	1.000	1.000
425	1.000	1.000	1.000	1.000	1.000	1.000
426	1.000	1.000	1.000	1.000	1.000	1.000
427	1.000	1.000	1.000	1.000	1.000	1.000
428	1.000	1.000	1.000	1.000	1.000	1.000
429	1.000	1.000	1.000	1.000	1.000	1.000
430	1.000	1.000	1.000	1.000	1.000	1.000
431	1.000	1.000	1.000	1.000	1.000	1.000
432	1.000	1.000	1.000	1.000	1.000	1.000
433	1.000	1.000	1.000	1.000	1.000	1.000
434	1.000	1.000	1.000	1.000	1.000	1.000
435	1.000	1.000	1.000	1.000	1.000	1.000
436	1.000	1.000	1.000	1.000	1.000	1.000
437	1.000	1.000	1.000	1.000	1.000	1.000
438	1.000	1.000	1.000	1.000	1.000	1.000
439	1.000	1.000	1.000	1.000	1.000	1.000
440	1.000	1.000	1.000	1.000	1.000	1.000
441	1.000	1.000	1.000	1.000	1.000	1.000
442	1.000	1.000	1.000	1.000	1.000	1.000
443	1.000	1.000	1.000	1.000	1.000	1.000

444	1.000	1.000	1.000	1.000	1.000	1.000
445	1.000	1.000	1.000	1.000	1.000	1.000
446	1.000	1.000	1.000	1.000	1.000	1.000
447	1.000	1.000	1.000	1.000	1.000	1.000
448	1.000	1.000	1.000	1.000	1.000	1.000
449	1.000	1.000	1.000	1.000	1.000	1.000
450	1.000	1.000	1.000	1.000	1.000	1.000
451	1.000	1.000	1.000	1.000	1.000	1.000
452	1.000	1.000	1.000	1.000	1.000	1.000
453	1.000	1.000	1.000	1.000	1.000	1.000
454	1.000	1.000	1.000	1.000	1.000	1.000
455	1.000	1.000	1.000	1.000	1.000	1.000
456	1.000	1.000	1.000	1.000	1.000	1.000
457	1.000	1.000	1.000	1.000	1.000	1.000
458	1.000	1.000	1.000	1.000	1.000	1.000
459	1.000	1.000	1.000	1.000	1.000	1.000
460	1.000	1.000	1.000	1.000	1.000	1.000
461	1.000	1.000	1.000	1.000	1.000	1.000
462	1.000	1.000	1.000	1.000	1.000	1.000
463	1.000	1.000	1.000	1.000	1.000	1.000
464	1.000	1.000	1.000	1.000	1.000	1.000
465	1.000	1.000	1.000	1.000	1.000	1.000
466	1.000	1.000	1.000	1.000	1.000	1.000
467	1.000	1.000	1.000	1.000	1.000	1.000
468	1.000	1.000	1.000	1.000	1.000	1.000
469	1.000	1.000	1.000	1.000	1.000	1.000
470	1.000	1.000	1.000	1.000	1.000	1.000
471	1.000	1.000	1.000	1.000	1.000	1.000
472	1.000	1.000	1.000	1.000	1.000	1.000
473	1.000	1.000	1.000	1.000	1.000	1.000
474	1.000	1.000	1.000	1.000	1.000	1.000
475	1.000	1.000	1.000	1.000	1.000	1.000
476	1.000	1.000	1.000	1.000	1.000	1.000
477	1.000	1.000	1.000	1.000	1.000	1.000
478	1.000	1.000	1.000	1.000	1.000	1.000
479	1.000	1.000	1.000	1.000	1.000	1.000
480	1.000	1.000	1.000	1.000	1.000	1.000
481	1.000	1.000	1.000	1.000	1.000	1.000
482	1.000	1.000	1.000	1.000	1.000	1.000
483	1.000	1.000	1.000	1.000	1.000	1.000
484	1.000	1.000	1.000	1.000	1.000	1.000
485	1.000	1.000	1.000	1.000	1.000	1.000

486	1.000	1.000	1.000	1.000	1.000	1.000
487	1.000	1.000	1.000	1.000	1.000	1.000
488	1.000	1.000	1.000	1.000	1.000	1.000
489	1.000	1.000	1.000	1.000	1.000	1.000
490	1.000	1.000	1.000	1.000	1.000	1.000
491	1.000	1.000	1.000	1.000	1.000	1.000
492	1.000	1.000	1.000	1.000	1.000	1.000
493	1.000	1.000	1.000	1.000	1.000	1.000
494	1.000	1.000	1.000	1.000	1.000	1.000
495	1.000	1.000	1.000	1.000	1.000	1.000
496	1.000	1.000	1.000	1.000	1.000	1.000
497	1.000	1.000	1.000	1.000	1.000	1.000
498	1.000	1.000	1.000	1.000	1.000	1.000
499	1.000	1.000	1.000	1.000	1.000	1.000
500	1.000	1.000	1.000	1.000	1.000	1.000
501	1.000	1.000	1.000	1.000	1.000	1.000
502	1.000	1.000	1.000	1.000	1.000	1.000
503	1.000	1.000	1.000	1.000	1.000	1.000
504	1.000	1.000	1.000	1.000	1.000	1.000
505	1.000	1.000	1.000	1.000	1.000	1.000
506	1.000	1.000	1.000	1.000	1.000	1.000
507	1.000	1.000	1.000	1.000	1.000	1.000
508	1.000	1.000	1.000	1.000	1.000	1.000
509	1.000	1.000	1.000	1.000	1.000	1.000
510	1.000	1.000	1.000	1.000	1.000	1.000
511	1.000	1.000	1.000	1.000	1.000	1.000
512	1.000	1.000	1.000	1.000	1.000	1.000
513	1.000	1.000	1.000	1.000	1.000	1.000
514	1.000	1.000	1.000	1.000	1.000	1.000
515	1.000	1.000	1.000	1.000	1.000	1.000
516	1.000	1.000	1.000	1.000	1.000	1.000
517	1.000	1.000	1.000	1.000	1.000	1.000
518	1.000	1.000	1.000	1.000	1.000	1.000
519	1.000	1.000	1.000	1.000	1.000	1.000
520	1.000	1.000	1.000	1.000	1.000	1.000
521	1.000	1.000	1.000	1.000	1.000	1.000
522	1.000	1.000	1.000	1.000	1.000	1.000
523	1.000	1.000	1.000	1.000	1.000	1.000
524	1.000	1.000	1.000	1.000	1.000	1.000
525	1.000	1.000	1.000	1.000	1.000	1.000
526	1.000	1.000	1.000	1.000	1.000	1.000
527	1.000	1.000	1.000	1.000	1.000	1.000

528	1.000	1.000	1.000	1.000	1.000	1.000
529	1.000	1.000	1.000	1.000	1.000	1.000
530	1.000	1.000	1.000	1.000	1.000	1.000
531	1.000	1.000	1.000	1.000	1.000	1.000
532	1.000	1.000	1.000	1.000	1.000	1.000
533	1.000	1.000	1.000	1.000	1.000	1.000
534	1.000	1.000	1.000	1.000	1.000	1.000
535	1.000	1.000	1.000	1.000	1.000	1.000
536	1.000	1.000	1.000	1.000	1.000	1.000
537	1.000	1.000	1.000	1.000	1.000	1.000
538	1.000	1.000	1.000	1.000	1.000	1.000
539	1.000	1.000	1.000	1.000	1.000	1.000
540	1.000	1.000	1.000	1.000	1.000	1.000
541	1.000	1.000	1.000	1.000	1.000	1.000
542	1.000	1.000	1.000	1.000	1.000	1.000
543	1.000	1.000	1.000	1.000	1.000	1.000
544	1.000	1.000	1.000	1.000	1.000	1.000
545	1.000	1.000	1.000	1.000	1.000	1.000
546	1.000	1.000	1.000	1.000	1.000	1.000
547	1.000	1.000	1.000	1.000	1.000	1.000
548	1.000	1.000	1.000	1.000	1.000	1.000
549	1.000	1.000	1.000	1.000	1.000	1.000
550	1.000	1.000	1.000	1.000	1.000	1.000
551	1.000	1.000	1.000	1.000	1.000	1.000
552	1.000	1.000	1.000	1.000	1.000	1.000
553	1.000	1.000	1.000	1.000	1.000	1.000
554	1.000	1.000	1.000	1.000	1.000	1.000
555	1.000	1.000	1.000	1.000	1.000	1.000
556	1.000	1.000	1.000	1.000	1.000	1.000
557	1.000	1.000	1.000	1.000	1.000	1.000
558	1.000	1.000	1.000	1.000	1.000	1.000
559	1.000	1.000	1.000	1.000	1.000	1.000
560	1.000	1.000	1.000	1.000	1.000	1.000
561	1.000	1.000	1.000	1.000	1.000	1.000
562	1.000	1.000	1.000	1.000	1.000	1.000
563	1.000	1.000	1.000	1.000	1.000	1.000
564	1.000	1.000	1.000	1.000	1.000	1.000
565	1.000	1.000	1.000	1.000	1.000	1.000
566	1.000	1.000	1.000	1.000	1.000	1.000
567	1.000	1.000	1.000	1.000	1.000	1.000
568	1.000	1.000	1.000	1.000	1.000	1.000
569	1.000	1.000	1.000	1.000	1.000	1.000

570	1.000	1.000	1.000	1.000	1.000	1.000
571	1.000	1.000	1.000	1.000	1.000	1.000
572	1.000	1.000	1.000	1.000	1.000	1.000
573	1.000	1.000	1.000	1.000	1.000	1.000
574	1.000	1.000	1.000	1.000	1.000	1.000
575	1.000	1.000	1.000	1.000	1.000	1.000
576	1.000	1.000	1.000	1.000	1.000	1.000
577	1.000	1.000	1.000	1.000	1.000	1.000
578	1.000	1.000	1.000	1.000	1.000	1.000
579	1.000	1.000	1.000	1.000	1.000	1.000
580	1.000	1.000	1.000	1.000	1.000	1.000
581	1.000	1.000	1.000	1.000	1.000	1.000
582	1.000	1.000	1.000	1.000	1.000	1.000
583	1.000	1.000	1.000	1.000	1.000	1.000
584	1.000	1.000	1.000	1.000	1.000	1.000
585	1.000	1.000	1.000	1.000	1.000	1.000
586	1.000	1.000	1.000	1.000	1.000	1.000
587	1.000	1.000	1.000	1.000	1.000	1.000
588	1.000	1.000	1.000	1.000	1.000	1.000
589	1.000	1.000	1.000	1.000	1.000	1.000
590	1.000	1.000	1.000	1.000	1.000	1.000
591	1.000	1.000	1.000	1.000	1.000	1.000
592	1.000	1.000	1.000	1.000	1.000	1.000
593	1.000	1.000	1.000	1.000	1.000	1.000
594	1.000	1.000	1.000	1.000	1.000	1.000
595	1.000	1.000	1.000	1.000	1.000	1.000
596	1.000	1.000	1.000	1.000	1.000	1.000
597	1.000	1.000	1.000	1.000	1.000	1.000
598	1.000	1.000	1.000	1.000	1.000	1.000
599	1.000	1.000	1.000	1.000	1.000	1.000
600	1.000	1.000	1.000	1.000	1.000	1.000
601	1.000	1.000	1.000	1.000	1.000	1.000
602	1.000	1.000	1.000	1.000	1.000	1.000
603	1.000	1.000	1.000	1.000	1.000	1.000
604	1.000	1.000	1.000	1.000	1.000	1.000
605	1.000	1.000	1.000	1.000	1.000	1.000
606	1.000	1.000	1.000	1.000	1.000	1.000
607	1.000	1.000	1.000	1.000	1.000	1.000
608	1.000	1.000	1.000	1.000	1.000	1.000
609	1.000	1.000	1.000	1.000	1.000	1.000
610	1.000	1.000	1.000	1.000	1.000	1.000
611	1.000	1.000	1.000	1.000	1.000	1.000

612	1.000	1.000	1.000	1.000	1.000	1.000
613	1.000	1.000	1.000	1.000	1.000	1.000
614	1.000	1.000	1.000	1.000	1.000	1.000
615	1.000	1.000	1.000	1.000	1.000	1.000
616	1.000	1.000	1.000	1.000	1.000	1.000
617	1.000	1.000	1.000	1.000	1.000	1.000
618	1.000	1.000	1.000	1.000	1.000	1.000
619	1.000	1.000	1.000	1.000	1.000	1.000
620	1.000	1.000	1.000	1.000	1.000	1.000
621	1.000	1.000	1.000	1.000	1.000	1.000
622	1.000	1.000	1.000	1.000	1.000	1.000
623	1.000	1.000	1.000	1.000	1.000	1.000
624	1.000	1.000	1.000	1.000	1.000	1.000
625	1.000	1.000	1.000	1.000	1.000	1.000
626	1.000	1.000	1.000	1.000	1.000	1.000
627	1.000	1.000	1.000	1.000	1.000	1.000
628	1.000	1.000	1.000	1.000	1.000	1.000
629	1.000	1.000	1.000	1.000	1.000	1.000
630	1.000	1.000	1.000	1.000	1.000	1.000
631	1.000	1.000	1.000	1.000	1.000	1.000
632	1.000	1.000	1.000	1.000	1.000	1.000
633	1.000	1.000	1.000	1.000	1.000	1.000
634	1.000	1.000	1.000	1.000	1.000	1.000
635	1.000	1.000	1.000	1.000	1.000	1.000
636	1.000	1.000	1.000	1.000	1.000	1.000
637	1.000	1.000	1.000	1.000	1.000	1.000
638	1.000	1.000	1.000	1.000	1.000	1.000
639	1.000	1.000	1.000	1.000	1.000	1.000
640	1.000	1.000	1.000	1.000	1.000	1.000
641	1.000	1.000	1.000	1.000	1.000	1.000
642	1.000	1.000	1.000	1.000	1.000	1.000
643	1.000	1.000	1.000	1.000	1.000	1.000
644	1.000	1.000	1.000	1.000	1.000	1.000
645	1.000	1.000	1.000	1.000	1.000	1.000
646	1.000	1.000	1.000	1.000	1.000	1.000
647	1.000	1.000	1.000	1.000	1.000	1.000
648	1.000	1.000	1.000	1.000	1.000	1.000
649	1.000	1.000	1.000	1.000	1.000	1.000
650	1.000	1.000	1.000	1.000	1.000	1.000
651	1.000	1.000	1.000	1.000	1.000	1.000
652	1.000	1.000	1.000	1.000	1.000	1.000
653	1.000	1.000	1.000	1.000	1.000	1.000

654	1.000	1.000	1.000	1.000	1.000	1.000
655	1.000	1.000	1.000	1.000	1.000	1.000
656	1.000	1.000	1.000	1.000	1.000	1.000
657	1.000	1.000	1.000	1.000	1.000	1.000
658	1.000	1.000	1.000	1.000	1.000	1.000
659	1.000	1.000	1.000	1.000	1.000	1.000
660	1.000	1.000	1.000	1.000	1.000	1.000
661	1.000	1.000	1.000	1.000	1.000	1.000
662	1.000	1.000	1.000	1.000	1.000	1.000
663	1.000	1.000	1.000	1.000	1.000	1.000
664	1.000	1.000	1.000	1.000	1.000	1.000
665	1.000	1.000	1.000	1.000	1.000	1.000
666	1.000	1.000	1.000	1.000	1.000	1.000
667	1.000	1.000	1.000	1.000	1.000	1.000
668	1.000	1.000	1.000	1.000	1.000	1.000
669	1.000	1.000	1.000	1.000	1.000	1.000
670	1.000	1.000	1.000	1.000	1.000	1.000
671	1.000	1.000	1.000	1.000	1.000	1.000
672	1.000	1.000	1.000	1.000	1.000	1.000
673	1.000	1.000	1.000	1.000	1.000	1.000
674	1.000	1.000	1.000	1.000	1.000	1.000
675	1.000	1.000	1.000	1.000	1.000	1.000
676	1.000	1.000	1.000	1.000	1.000	1.000
677	1.000	1.000	1.000	1.000	1.000	1.000
678	1.000	1.000	1.000	1.000	1.000	1.000
679	1.000	1.000	1.000	1.000	1.000	1.000
680	1.000	1.000	1.000	1.000	1.000	1.000
681	1.000	1.000	1.000	1.000	1.000	1.000
682	1.000	1.000	1.000	1.000	1.000	1.000
683	1.000	1.000	1.000	1.000	1.000	1.000
684	1.000	1.000	1.000	1.000	1.000	1.000
685	1.000	1.000	1.000	1.000	1.000	1.000
686	1.000	1.000	1.000	1.000	1.000	1.000
687	1.000	1.000	1.000	1.000	1.000	1.000
688	1.000	1.000	1.000	1.000	1.000	1.000
689	1.000	1.000	1.000	1.000	1.000	1.000
690	1.000	1.000	1.000	1.000	1.000	1.000
691	1.000	1.000	1.000	1.000	1.000	1.000
692	1.000	1.000	1.000	1.000	1.000	1.000
693	1.000	1.000	1.000	1.000	1.000	1.000
694	1.000	1.000	1.000	1.000	1.000	1.000
695	1.000	1.000	1.000	1.000	1.000	1.000

696	1.000	1.000	1.000	1.000	1.000	1.000
697	1.000	1.000	1.000	1.000	1.000	1.000
698	1.000	1.000	1.000	1.000	1.000	1.000
699	1.000	1.000	1.000	1.000	1.000	1.000
700	1.000	1.000	1.000	1.000	1.000	1.000
701	1.000	1.000	1.000	1.000	1.000	1.000
702	1.000	1.000	1.000	1.000	1.000	1.000
703	1.000	1.000	1.000	1.000	1.000	1.000
704	1.000	1.000	1.000	1.000	1.000	1.000
705	1.000	1.000	1.000	1.000	1.000	1.000
706	1.000	1.000	1.000	1.000	1.000	1.000
707	1.000	1.000	1.000	1.000	1.000	1.000
708	1.000	1.000	1.000	1.000	1.000	1.000
709	1.000	1.000	1.000	1.000	1.000	1.000
710	1.000	1.000	1.000	1.000	1.000	1.000
711	1.000	1.000	1.000	1.000	1.000	1.000
712	1.000	1.000	1.000	1.000	1.000	1.000
713	1.000	1.000	1.000	1.000	1.000	1.000
714	1.000	1.000	1.000	1.000	1.000	1.000
715	1.000	1.000	1.000	1.000	1.000	1.000
716	1.000	1.000	1.000	1.000	1.000	1.000
717	1.000	1.000	1.000	1.000	1.000	1.000
718	1.000	1.000	1.000	1.000	1.000	1.000
719	1.000	1.000	1.000	1.000	1.000	1.000
720	1.000	1.000	1.000	1.000	1.000	1.000
721	1.000	1.000	1.000	1.000	1.000	1.000
722	1.000	1.000	1.000	1.000	1.000	1.000
723	1.000	1.000	1.000	1.000	1.000	1.000
724	1.000	1.000	1.000	1.000	1.000	1.000
725	1.000	1.000	1.000	1.000	1.000	1.000
726	1.000	1.000	1.000	1.000	1.000	1.000
727	1.000	1.000	1.000	1.000	1.000	1.000
728	1.000	1.000	1.000	1.000	1.000	1.000
729	1.000	1.000	1.000	1.000	1.000	1.000
730	1.000	1.000	1.000	1.000	1.000	1.000
731	1.000	1.000	1.000	1.000	1.000	1.000
732	1.000	1.000	1.000	1.000	1.000	1.000
733	1.000	1.000	1.000	1.000	1.000	1.000
734	1.000	1.000	1.000	1.000	1.000	1.000
735	1.000	1.000	1.000	1.000	1.000	1.000
736	1.000	1.000	1.000	1.000	1.000	1.000
737	1.000	1.000	1.000	1.000	1.000	1.000

738	1.000	1.000	1.000	1.000	1.000	1.000
739	1.000	1.000	1.000	1.000	1.000	1.000
740	1.000	1.000	1.000	1.000	1.000	1.000
741	1.000	1.000	1.000	1.000	1.000	1.000
742	1.000	1.000	1.000	1.000	1.000	1.000
743	1.000	1.000	1.000	1.000	1.000	1.000
744	1.000	1.000	1.000	1.000	1.000	1.000
745	1.000	1.000	1.000	1.000	1.000	1.000
746	1.000	1.000	1.000	1.000	1.000	1.000
747	1.000	1.000	1.000	1.000	1.000	1.000
748	1.000	1.000	1.000	1.000	1.000	1.000
749	1.000	1.000	1.000	1.000	1.000	1.000
750	1.000	1.000	1.000	1.000	1.000	1.000
751	1.000	1.000	1.000	1.000	1.000	1.000
752	1.000	1.000	1.000	1.000	1.000	1.000
753	1.000	1.000	1.000	1.000	1.000	1.000
754	1.000	1.000	1.000	1.000	1.000	1.000
755	1.000	1.000	1.000	1.000	1.000	1.000
756	1.000	1.000	1.000	1.000	1.000	1.000
757	1.000	1.000	1.000	1.000	1.000	1.000
758	1.000	1.000	1.000	1.000	1.000	1.000
759	1.000	1.000	1.000	1.000	1.000	1.000
760	1.000	1.000	1.000	1.000	1.000	1.000
761	1.000	1.000	1.000	1.000	1.000	1.000
762	1.000	1.000	1.000	1.000	1.000	1.000
763	1.000	1.000	1.000	1.000	1.000	1.000
764	1.000	1.000	1.000	1.000	1.000	1.000
765	1.000	1.000	1.000	1.000	1.000	1.000
766	1.000	1.000	1.000	1.000	1.000	1.000
767	1.000	1.000	1.000	1.000	1.000	1.000
768	1.000	1.000	1.000	1.000	1.000	1.000
769	1.000	1.000	1.000	1.000	1.000	1.000
770	1.000	1.000	1.000	1.000	1.000	1.000
771	1.000	1.000	1.000	1.000	1.000	1.000
772	1.000	1.000	1.000	1.000	1.000	1.000
773	1.000	1.000	1.000	1.000	1.000	1.000
774	1.000	1.000	1.000	1.000	1.000	1.000
775	1.000	1.000	1.000	1.000	1.000	1.000
776	1.000	1.000	1.000	1.000	1.000	1.000
777	1.000	1.000	1.000	1.000	1.000	1.000
778	1.000	1.000	1.000	1.000	1.000	1.000
779	1.000	1.000	1.000	1.000	1.000	1.000

780	1.000	1.000	1.000	1.000	1.000	1.000
781	1.000	1.000	1.000	1.000	1.000	1.000
782	1.000	1.000	1.000	1.000	1.000	1.000
783	1.000	1.000	1.000	1.000	1.000	1.000
784	1.000	1.000	1.000	1.000	1.000	1.000
785	1.000	1.000	1.000	1.000	1.000	1.000
786	1.000	1.000	1.000	1.000	1.000	1.000
787	1.000	1.000	1.000	1.000	1.000	1.000
788	1.000	1.000	1.000	1.000	1.000	1.000
789	1.000	1.000	1.000	1.000	1.000	1.000
790	1.000	1.000	1.000	1.000	1.000	1.000
791	1.000	1.000	1.000	1.000	1.000	1.000
792	1.000	1.000	1.000	1.000	1.000	1.000
793	1.000	1.000	1.000	1.000	1.000	1.000
794	1.000	1.000	1.000	1.000	1.000	1.000
795	1.000	1.000	1.000	1.000	1.000	1.000
796	1.000	1.000	1.000	1.000	1.000	1.000
797	1.000	1.000	1.000	1.000	1.000	1.000
798	1.000	1.000	1.000	1.000	1.000	1.000
799	1.000	1.000	1.000	1.000	1.000	1.000
800	1.000	1.000	1.000	1.000	1.000	1.000
801	1.000	1.000	1.000	1.000	1.000	1.000
802	1.000	1.000	1.000	1.000	1.000	1.000
803	1.000	1.000	1.000	1.000	1.000	1.000
804	1.000	1.000	1.000	1.000	1.000	1.000
805	1.000	1.000	1.000	1.000	1.000	1.000
806	1.000	1.000	1.000	1.000	1.000	1.000
807	1.000	1.000	1.000	1.000	1.000	1.000
808	1.000	1.000	1.000	1.000	1.000	1.000
809	1.000	1.000	1.000	1.000	1.000	1.000
810	1.000	1.000	1.000	1.000	1.000	1.000
811	1.000	1.000	1.000	1.000	1.000	1.000
812	1.000	1.000	1.000	1.000	1.000	1.000
813	1.000	1.000	1.000	1.000	1.000	1.000
814	1.000	1.000	1.000	1.000	1.000	1.000
815	1.000	1.000	1.000	1.000	1.000	1.000
816	1.000	1.000	1.000	1.000	1.000	1.000
817	1.000	1.000	1.000	1.000	1.000	1.000
818	1.000	1.000	1.000	1.000	1.000	1.000
819	1.000	1.000	1.000	1.000	1.000	1.000
820	1.000	1.000	1.000	1.000	1.000	1.000
821	1.000	1.000	1.000	1.000	1.000	1.000

822	1.000	1.000	1.000	1.000	1.000	1.000
823	1.000	1.000	1.000	1.000	1.000	1.000
824	1.000	1.000	1.000	1.000	1.000	1.000
825	1.000	1.000	1.000	1.000	1.000	1.000
826	1.000	1.000	1.000	1.000	1.000	1.000
827	1.000	1.000	1.000	1.000	1.000	1.000
828	1.000	1.000	1.000	1.000	1.000	1.000
829	1.000	1.000	1.000	1.000	1.000	1.000
830	1.000	1.000	1.000	1.000	1.000	1.000
831	1.000	1.000	1.000	1.000	1.000	1.000
832	1.000	1.000	1.000	1.000	1.000	1.000
833	1.000	1.000	1.000	1.000	1.000	1.000
834	1.000	1.000	1.000	1.000	1.000	1.000
835	1.000	1.000	1.000	1.000	1.000	1.000
836	1.000	1.000	1.000	1.000	1.000	1.000
837	1.000	1.000	1.000	1.000	1.000	1.000
838	1.000	1.000	1.000	1.000	1.000	1.000
839	1.000	1.000	1.000	1.000	1.000	1.000
840	1.000	1.000	1.000	1.000	1.000	1.000
841	1.000	1.000	1.000	1.000	1.000	1.000
842	1.000	1.000	1.000	1.000	1.000	1.000
843	1.000	1.000	1.000	1.000	1.000	1.000
844	1.000	1.000	1.000	1.000	1.000	1.000
845	1.000	1.000	1.000	1.000	1.000	1.000
846	1.000	1.000	1.000	1.000	1.000	1.000
847	1.000	1.000	1.000	1.000	1.000	1.000
848	1.000	1.000	1.000	1.000	1.000	1.000
849	1.000	1.000	1.000	1.000	1.000	1.000
850	1.000	1.000	1.000	1.000	1.000	1.000
851	1.000	1.000	1.000	1.000	1.000	1.000
852	1.000	1.000	1.000	1.000	1.000	1.000
853	1.000	1.000	1.000	1.000	1.000	1.000
854	1.000	1.000	1.000	1.000	1.000	1.000
855	1.000	1.000	1.000	1.000	1.000	1.000
856	1.000	1.000	1.000	1.000	1.000	1.000
857	1.000	1.000	1.000	1.000	1.000	1.000
858	1.000	1.000	1.000	1.000	1.000	1.000
859	1.000	1.000	1.000	1.000	1.000	1.000
860	1.000	1.000	1.000	1.000	1.000	1.000
861	1.000	1.000	1.000	1.000	1.000	1.000
862	1.000	1.000	1.000	1.000	1.000	1.000
863	1.000	1.000	1.000	1.000	1.000	1.000

864	1.000	1.000	1.000	1.000	1.000	1.000
865	1.000	1.000	1.000	1.000	1.000	1.000
866	1.000	1.000	1.000	1.000	1.000	1.000
867	1.000	1.000	1.000	1.000	1.000	1.000
868	1.000	1.000	1.000	1.000	1.000	1.000
869	1.000	1.000	1.000	1.000	1.000	1.000
870	1.000	1.000	1.000	1.000	1.000	1.000
871	1.000	1.000	1.000	1.000	1.000	1.000
872	1.000	1.000	1.000	1.000	1.000	1.000
873	1.000	1.000	1.000	1.000	1.000	1.000
874	1.000	1.000	1.000	1.000	1.000	1.000
875	1.000	1.000	1.000	1.000	1.000	1.000
876	1.000	1.000	1.000	1.000	1.000	1.000
877	1.000	1.000	1.000	1.000	1.000	1.000
878	1.000	1.000	1.000	1.000	1.000	1.000
879	1.000	1.000	1.000	1.000	1.000	1.000
880	1.000	1.000	1.000	1.000	1.000	1.000
881	1.000	1.000	1.000	1.000	1.000	1.000
882	1.000	1.000	1.000	1.000	1.000	1.000
883	1.000	1.000	1.000	1.000	1.000	1.000
884	1.000	1.000	1.000	1.000	1.000	1.000
885	1.000	1.000	1.000	1.000	1.000	1.000
886	1.000	1.000	1.000	1.000	1.000	1.000
887	1.000	1.000	1.000	1.000	1.000	1.000
888	1.000	1.000	1.000	1.000	1.000	1.000
889	1.000	1.000	1.000	1.000	1.000	1.000
890	1.000	1.000	1.000	1.000	1.000	1.000
891	1.000	1.000	1.000	1.000	1.000	1.000
892	1.000	1.000	1.000	1.000	1.000	1.000
893	1.000	1.000	1.000	1.000	1.000	1.000
894	1.000	1.000	1.000	1.000	1.000	1.000
895	1.000	1.000	1.000	1.000	1.000	1.000
896	1.000	1.000	1.000	1.000	1.000	1.000
897	1.000	1.000	1.000	1.000	1.000	1.000
898	1.000	1.000	1.000	1.000	1.000	1.000
899	1.000	1.000	1.000	1.000	1.000	1.000
900	1.000	1.000	1.000	1.000	1.000	1.000
901	1.000	1.000	1.000	1.000	1.000	1.000
902	1.000	1.000	1.000	1.000	1.000	1.000
903	1.000	1.000	1.000	1.000	1.000	1.000
904	1.000	1.000	1.000	1.000	1.000	1.000
905	1.000	1.000	1.000	1.000	1.000	1.000

906	1.000	1.000	1.000	1.000	1.000	1.000
907	1.000	1.000	1.000	1.000	1.000	1.000
908	1.000	1.000	1.000	1.000	1.000	1.000
909	1.000	1.000	1.000	1.000	1.000	1.000
910	1.000	1.000	1.000	1.000	1.000	1.000
911	1.000	1.000	1.000	1.000	1.000	1.000
912	1.000	1.000	1.000	1.000	1.000	1.000
913	1.000	1.000	1.000	1.000	1.000	1.000
914	1.000	1.000	1.000	1.000	1.000	1.000
915	1.000	1.000	1.000	1.000	1.000	1.000
916	1.000	1.000	1.000	1.000	1.000	1.000
917	1.000	1.000	1.000	1.000	1.000	1.000
918	1.000	1.000	1.000	1.000	1.000	1.000
919	1.000	1.000	1.000	1.000	1.000	1.000
920	1.000	1.000	1.000	1.000	1.000	1.000
921	1.000	1.000	1.000	1.000	1.000	1.000
922	1.000	1.000	1.000	1.000	1.000	1.000
923	1.000	1.000	1.000	1.000	1.000	1.000
924	1.000	1.000	1.000	1.000	1.000	1.000
925	1.000	1.000	1.000	1.000	1.000	1.000
926	1.000	1.000	1.000	1.000	1.000	1.000
927	1.000	1.000	1.000	1.000	1.000	1.000
928	1.000	1.000	1.000	1.000	1.000	1.000
929	1.000	1.000	1.000	1.000	1.000	1.000
930	1.000	1.000	1.000	1.000	1.000	1.000
931	1.000	1.000	1.000	1.000	1.000	1.000
932	1.000	1.000	1.000	1.000	1.000	1.000
933	1.000	1.000	1.000	1.000	1.000	1.000
934	1.000	1.000	1.000	1.000	1.000	1.000
935	1.000	1.000	1.000	1.000	1.000	1.000
936	1.000	1.000	1.000	1.000	1.000	1.000
937	1.000	1.000	1.000	1.000	1.000	1.000
938	1.000	1.000	1.000	1.000	1.000	1.000
939	1.000	1.000	1.000	1.000	1.000	1.000
940	1.000	1.000	1.000	1.000	1.000	1.000
941	1.000	1.000	1.000	1.000	1.000	1.000
942	1.000	1.000	1.000	1.000	1.000	1.000
943	1.000	1.000	1.000	1.000	1.000	1.000
944	1.000	1.000	1.000	1.000	1.000	1.000
945	1.000	1.000	1.000	1.000	1.000	1.000
946	1.000	1.000	1.000	1.000	1.000	1.000
947	1.000	1.000	1.000	1.000	1.000	1.000

948	1.000	1.000	1.000	1.000	1.000	1.000
949	1.000	1.000	1.000	1.000	1.000	1.000
950	1.000	1.000	1.000	1.000	1.000	1.000
951	1.000	1.000	1.000	1.000	1.000	1.000
952	1.000	1.000	1.000	1.000	1.000	1.000
953	1.000	1.000	1.000	1.000	1.000	1.000
954	1.000	1.000	1.000	1.000	1.000	1.000
955	1.000	1.000	1.000	1.000	1.000	1.000
956	1.000	1.000	1.000	1.000	1.000	1.000
957	1.000	1.000	1.000	1.000	1.000	1.000
958	1.000	1.000	1.000	1.000	1.000	1.000
959	1.000	1.000	1.000	1.000	1.000	1.000
960	1.000	1.000	1.000	1.000	1.000	1.000
961	1.000	1.000	1.000	1.000	1.000	1.000
962	1.000	1.000	1.000	1.000	1.000	1.000
963	1.000	1.000	1.000	1.000	1.000	1.000
964	1.000	1.000	1.000	1.000	1.000	1.000
965	1.000	1.000	1.000	1.000	1.000	1.000
966	1.000	1.000	1.000	1.000	1.000	1.000
967	1.000	1.000	1.000	1.000	1.000	1.000
968	1.000	1.000	1.000	1.000	1.000	1.000
969	1.000	1.000	1.000	1.000	1.000	1.000
970	1.000	1.000	1.000	1.000	1.000	1.000
971	1.000	1.000	1.000	1.000	1.000	1.000
972	1.000	1.000	1.000	1.000	1.000	1.000
973	1.000	1.000	1.000	1.000	1.000	1.000
974	1.000	1.000	1.000	1.000	1.000	1.000
975	1.000	1.000	1.000	1.000	1.000	1.000
976	1.000	1.000	1.000	1.000	1.000	1.000
977	1.000	1.000	1.000	1.000	1.000	1.000
978	1.000	1.000	1.000	1.000	1.000	1.000
979	1.000	1.000	1.000	1.000	1.000	1.000
980	1.000	1.000	1.000	1.000	1.000	1.000
981	1.000	1.000	1.000	1.000	1.000	1.000
982	1.000	1.000	1.000	1.000	1.000	1.000
983	1.000	1.000	1.000	1.000	1.000	1.000
984	1.000	1.000	1.000	1.000	1.000	1.000
985	1.000	1.000	1.000	1.000	1.000	1.000
986	1.000	1.000	1.000	1.000	1.000	1.000
987	1.000	1.000	1.000	1.000	1.000	1.000
988	1.000	1.000	1.000	1.000	1.000	1.000
989	1.000	1.000	1.000	1.000	1.000	1.000

990	1.000	1.000	1.000	1.000	1.000	1.000
991	1.000	1.000	1.000	1.000	1.000	1.000
992	1.000	1.000	1.000	1.000	1.000	1.000
993	1.000	1.000	1.000	1.000	1.000	1.000
994	1.000	1.000	1.000	1.000	1.000	1.000
995	1.000	1.000	1.000	1.000	1.000	1.000
996	1.000	1.000	1.000	1.000	1.000	1.000
997	1.000	1.000	1.000	1.000	1.000	1.000
998	1.000	1.000	1.000	1.000	1.000	1.000
999	1.000	1.000	1.000	1.000	1.000	1.000
1000	1.000	1.000	1.000	1.000	1.000	1.000
1001	1.000	1.000	1.000	1.000	1.000	1.000
1002	1.000	1.000	1.000	1.000	1.000	1.000
1003	1.000	1.000	1.000	1.000	1.000	1.000
1004	1.000	1.000	1.000	1.000	1.000	1.000
1005	1.000	1.000	1.000	1.000	1.000	1.000
1006	1.000	1.000	1.000	1.000	1.000	1.000
1007	1.000	1.000	1.000	1.000	1.000	1.000
1008	1.000	1.000	1.000	1.000	1.000	1.000
1009	1.000	1.000	1.000	1.000	1.000	1.000
1010	1.000	1.000	1.000	1.000	1.000	1.000
1011	1.000	1.000	1.000	1.000	1.000	1.000
1012	1.000	1.000	1.000	1.000	1.000	1.000
1013	1.000	1.000	1.000	1.000	1.000	1.000
1014	1.000	1.000	1.000	1.000	1.000	1.000
1015	1.000	1.000	1.000	1.000	1.000	1.000
1016	1.000	1.000	1.000	1.000	1.000	1.000
1017	1.000	1.000	1.000	1.000	1.000	1.000
1018	1.000	1.000	1.000	1.000	1.000	1.000
1019	1.000	1.000	1.000	1.000	1.000	1.000
1020	1.000	1.000	1.000	1.000	1.000	1.000

(3) Random forest

Number of features	SN	SP	ACC	MCC	Precision	F1-measure
1	1.000	0.948	0.974	0.950	0.951	0.975
2	1.000	0.990	0.995	0.990	0.990	0.995
3	1.000	0.990	0.995	0.990	0.990	0.995
4	1.000	0.990	0.995	0.990	0.990	0.995
5	1.000	0.990	0.995	0.990	0.990	0.995
6	1.000	0.990	0.995	0.990	0.990	0.995
7	1.000	0.979	0.990	0.980	0.980	0.990
8	1.000	0.990	0.995	0.990	0.990	0.995

9	1.000	0.990	0.995	0.990	0.990	0.995
10	1.000	1.000	1.000	1.000	1.000	1.000
11	1.000	0.990	0.995	0.990	0.990	0.995
12	1.000	0.990	0.995	0.990	0.990	0.995
13	1.000	1.000	1.000	1.000	1.000	1.000
14	1.000	1.000	1.000	1.000	1.000	1.000
15	1.000	1.000	1.000	1.000	1.000	1.000
16	1.000	1.000	1.000	1.000	1.000	1.000
17	1.000	1.000	1.000	1.000	1.000	1.000
18	1.000	1.000	1.000	1.000	1.000	1.000
19	1.000	1.000	1.000	1.000	1.000	1.000
20	1.000	1.000	1.000	1.000	1.000	1.000
21	1.000	1.000	1.000	1.000	1.000	1.000
22	1.000	1.000	1.000	1.000	1.000	1.000
23	1.000	1.000	1.000	1.000	1.000	1.000
24	1.000	1.000	1.000	1.000	1.000	1.000
25	1.000	0.990	0.995	0.990	0.990	0.995
26	1.000	1.000	1.000	1.000	1.000	1.000
27	1.000	1.000	1.000	1.000	1.000	1.000
28	1.000	1.000	1.000	1.000	1.000	1.000
29	1.000	1.000	1.000	1.000	1.000	1.000
30	1.000	1.000	1.000	1.000	1.000	1.000
31	1.000	1.000	1.000	1.000	1.000	1.000
32	1.000	1.000	1.000	1.000	1.000	1.000
33	1.000	1.000	1.000	1.000	1.000	1.000
34	1.000	1.000	1.000	1.000	1.000	1.000
35	1.000	1.000	1.000	1.000	1.000	1.000
36	1.000	1.000	1.000	1.000	1.000	1.000
37	1.000	1.000	1.000	1.000	1.000	1.000
38	1.000	1.000	1.000	1.000	1.000	1.000
39	1.000	1.000	1.000	1.000	1.000	1.000
40	1.000	1.000	1.000	1.000	1.000	1.000
41	1.000	1.000	1.000	1.000	1.000	1.000
42	1.000	1.000	1.000	1.000	1.000	1.000
43	1.000	1.000	1.000	1.000	1.000	1.000
44	1.000	1.000	1.000	1.000	1.000	1.000
45	1.000	1.000	1.000	1.000	1.000	1.000
46	1.000	1.000	1.000	1.000	1.000	1.000
47	1.000	1.000	1.000	1.000	1.000	1.000
48	1.000	1.000	1.000	1.000	1.000	1.000
49	1.000	1.000	1.000	1.000	1.000	1.000
50	1.000	1.000	1.000	1.000	1.000	1.000

51	1.000	1.000	1.000	1.000	1.000	1.000
52	1.000	1.000	1.000	1.000	1.000	1.000
53	1.000	1.000	1.000	1.000	1.000	1.000
54	1.000	1.000	1.000	1.000	1.000	1.000
55	1.000	1.000	1.000	1.000	1.000	1.000
56	1.000	1.000	1.000	1.000	1.000	1.000
57	1.000	1.000	1.000	1.000	1.000	1.000
58	1.000	1.000	1.000	1.000	1.000	1.000
59	1.000	1.000	1.000	1.000	1.000	1.000
60	1.000	1.000	1.000	1.000	1.000	1.000
61	1.000	1.000	1.000	1.000	1.000	1.000
62	1.000	1.000	1.000	1.000	1.000	1.000
63	1.000	1.000	1.000	1.000	1.000	1.000
64	1.000	1.000	1.000	1.000	1.000	1.000
65	1.000	1.000	1.000	1.000	1.000	1.000
66	1.000	1.000	1.000	1.000	1.000	1.000
67	1.000	1.000	1.000	1.000	1.000	1.000
68	1.000	1.000	1.000	1.000	1.000	1.000
69	1.000	1.000	1.000	1.000	1.000	1.000
70	1.000	1.000	1.000	1.000	1.000	1.000
71	1.000	1.000	1.000	1.000	1.000	1.000
72	1.000	1.000	1.000	1.000	1.000	1.000
73	1.000	1.000	1.000	1.000	1.000	1.000
74	1.000	1.000	1.000	1.000	1.000	1.000
75	1.000	1.000	1.000	1.000	1.000	1.000
76	1.000	1.000	1.000	1.000	1.000	1.000
77	1.000	1.000	1.000	1.000	1.000	1.000
78	1.000	1.000	1.000	1.000	1.000	1.000
79	1.000	1.000	1.000	1.000	1.000	1.000
80	1.000	1.000	1.000	1.000	1.000	1.000
81	1.000	1.000	1.000	1.000	1.000	1.000
82	1.000	1.000	1.000	1.000	1.000	1.000
83	1.000	1.000	1.000	1.000	1.000	1.000
84	1.000	1.000	1.000	1.000	1.000	1.000
85	1.000	1.000	1.000	1.000	1.000	1.000
86	1.000	1.000	1.000	1.000	1.000	1.000
87	1.000	1.000	1.000	1.000	1.000	1.000
88	1.000	1.000	1.000	1.000	1.000	1.000
89	1.000	1.000	1.000	1.000	1.000	1.000
90	1.000	1.000	1.000	1.000	1.000	1.000
91	1.000	1.000	1.000	1.000	1.000	1.000
92	1.000	1.000	1.000	1.000	1.000	1.000

93	1.000	1.000	1.000	1.000	1.000	1.000
94	1.000	1.000	1.000	1.000	1.000	1.000
95	1.000	1.000	1.000	1.000	1.000	1.000
96	1.000	1.000	1.000	1.000	1.000	1.000
97	1.000	1.000	1.000	1.000	1.000	1.000
98	1.000	1.000	1.000	1.000	1.000	1.000
99	1.000	1.000	1.000	1.000	1.000	1.000
100	1.000	1.000	1.000	1.000	1.000	1.000
101	1.000	1.000	1.000	1.000	1.000	1.000
102	1.000	0.990	0.995	0.990	0.990	0.995
103	1.000	1.000	1.000	1.000	1.000	1.000
104	1.000	1.000	1.000	1.000	1.000	1.000
105	1.000	1.000	1.000	1.000	1.000	1.000
106	1.000	1.000	1.000	1.000	1.000	1.000
107	1.000	1.000	1.000	1.000	1.000	1.000
108	1.000	1.000	1.000	1.000	1.000	1.000
109	1.000	1.000	1.000	1.000	1.000	1.000
110	1.000	1.000	1.000	1.000	1.000	1.000
111	1.000	1.000	1.000	1.000	1.000	1.000
112	1.000	1.000	1.000	1.000	1.000	1.000
113	1.000	1.000	1.000	1.000	1.000	1.000
114	1.000	1.000	1.000	1.000	1.000	1.000
115	1.000	1.000	1.000	1.000	1.000	1.000
116	1.000	1.000	1.000	1.000	1.000	1.000
117	1.000	1.000	1.000	1.000	1.000	1.000
118	1.000	1.000	1.000	1.000	1.000	1.000
119	1.000	1.000	1.000	1.000	1.000	1.000
120	1.000	1.000	1.000	1.000	1.000	1.000
121	1.000	1.000	1.000	1.000	1.000	1.000
122	1.000	1.000	1.000	1.000	1.000	1.000
123	1.000	1.000	1.000	1.000	1.000	1.000
124	1.000	1.000	1.000	1.000	1.000	1.000
125	1.000	1.000	1.000	1.000	1.000	1.000
126	1.000	1.000	1.000	1.000	1.000	1.000
127	1.000	1.000	1.000	1.000	1.000	1.000
128	1.000	1.000	1.000	1.000	1.000	1.000
129	1.000	1.000	1.000	1.000	1.000	1.000
130	1.000	1.000	1.000	1.000	1.000	1.000
131	1.000	1.000	1.000	1.000	1.000	1.000
132	1.000	1.000	1.000	1.000	1.000	1.000
133	1.000	1.000	1.000	1.000	1.000	1.000
134	1.000	1.000	1.000	1.000	1.000	1.000

135	1.000	1.000	1.000	1.000	1.000	1.000
136	1.000	1.000	1.000	1.000	1.000	1.000
137	1.000	1.000	1.000	1.000	1.000	1.000
138	1.000	1.000	1.000	1.000	1.000	1.000
139	1.000	1.000	1.000	1.000	1.000	1.000
140	1.000	1.000	1.000	1.000	1.000	1.000
141	1.000	1.000	1.000	1.000	1.000	1.000
142	1.000	1.000	1.000	1.000	1.000	1.000
143	1.000	1.000	1.000	1.000	1.000	1.000
144	1.000	1.000	1.000	1.000	1.000	1.000
145	1.000	1.000	1.000	1.000	1.000	1.000
146	1.000	1.000	1.000	1.000	1.000	1.000
147	1.000	1.000	1.000	1.000	1.000	1.000
148	1.000	1.000	1.000	1.000	1.000	1.000
149	1.000	1.000	1.000	1.000	1.000	1.000
150	1.000	1.000	1.000	1.000	1.000	1.000
151	1.000	1.000	1.000	1.000	1.000	1.000
152	1.000	1.000	1.000	1.000	1.000	1.000
153	1.000	1.000	1.000	1.000	1.000	1.000
154	1.000	1.000	1.000	1.000	1.000	1.000
155	1.000	1.000	1.000	1.000	1.000	1.000
156	1.000	1.000	1.000	1.000	1.000	1.000
157	1.000	1.000	1.000	1.000	1.000	1.000
158	1.000	1.000	1.000	1.000	1.000	1.000
159	1.000	1.000	1.000	1.000	1.000	1.000
160	1.000	1.000	1.000	1.000	1.000	1.000
161	1.000	1.000	1.000	1.000	1.000	1.000
162	1.000	1.000	1.000	1.000	1.000	1.000
163	1.000	1.000	1.000	1.000	1.000	1.000
164	1.000	1.000	1.000	1.000	1.000	1.000
165	1.000	1.000	1.000	1.000	1.000	1.000
166	1.000	1.000	1.000	1.000	1.000	1.000
167	1.000	1.000	1.000	1.000	1.000	1.000
168	1.000	1.000	1.000	1.000	1.000	1.000
169	1.000	1.000	1.000	1.000	1.000	1.000
170	1.000	1.000	1.000	1.000	1.000	1.000
171	1.000	1.000	1.000	1.000	1.000	1.000
172	1.000	1.000	1.000	1.000	1.000	1.000
173	1.000	1.000	1.000	1.000	1.000	1.000
174	1.000	1.000	1.000	1.000	1.000	1.000
175	1.000	1.000	1.000	1.000	1.000	1.000
176	1.000	1.000	1.000	1.000	1.000	1.000

177	1.000	1.000	1.000	1.000	1.000	1.000
178	1.000	1.000	1.000	1.000	1.000	1.000
179	1.000	1.000	1.000	1.000	1.000	1.000
180	1.000	1.000	1.000	1.000	1.000	1.000
181	1.000	1.000	1.000	1.000	1.000	1.000
182	1.000	1.000	1.000	1.000	1.000	1.000
183	1.000	1.000	1.000	1.000	1.000	1.000
184	1.000	1.000	1.000	1.000	1.000	1.000
185	1.000	1.000	1.000	1.000	1.000	1.000
186	1.000	1.000	1.000	1.000	1.000	1.000
187	1.000	1.000	1.000	1.000	1.000	1.000
188	1.000	1.000	1.000	1.000	1.000	1.000
189	1.000	1.000	1.000	1.000	1.000	1.000
190	1.000	1.000	1.000	1.000	1.000	1.000
191	1.000	1.000	1.000	1.000	1.000	1.000
192	1.000	1.000	1.000	1.000	1.000	1.000
193	1.000	1.000	1.000	1.000	1.000	1.000
194	1.000	1.000	1.000	1.000	1.000	1.000
195	1.000	1.000	1.000	1.000	1.000	1.000
196	1.000	1.000	1.000	1.000	1.000	1.000
197	1.000	1.000	1.000	1.000	1.000	1.000
198	1.000	1.000	1.000	1.000	1.000	1.000
199	1.000	1.000	1.000	1.000	1.000	1.000
200	1.000	1.000	1.000	1.000	1.000	1.000
201	1.000	1.000	1.000	1.000	1.000	1.000
202	1.000	1.000	1.000	1.000	1.000	1.000
203	1.000	1.000	1.000	1.000	1.000	1.000
204	1.000	1.000	1.000	1.000	1.000	1.000
205	1.000	1.000	1.000	1.000	1.000	1.000
206	1.000	1.000	1.000	1.000	1.000	1.000
207	1.000	1.000	1.000	1.000	1.000	1.000
208	1.000	1.000	1.000	1.000	1.000	1.000
209	1.000	1.000	1.000	1.000	1.000	1.000
210	1.000	1.000	1.000	1.000	1.000	1.000
211	1.000	1.000	1.000	1.000	1.000	1.000
212	1.000	1.000	1.000	1.000	1.000	1.000
213	1.000	1.000	1.000	1.000	1.000	1.000
214	1.000	1.000	1.000	1.000	1.000	1.000
215	1.000	1.000	1.000	1.000	1.000	1.000
216	1.000	1.000	1.000	1.000	1.000	1.000
217	1.000	1.000	1.000	1.000	1.000	1.000
218	1.000	1.000	1.000	1.000	1.000	1.000

219	1.000	1.000	1.000	1.000	1.000	1.000
220	1.000	1.000	1.000	1.000	1.000	1.000
221	1.000	1.000	1.000	1.000	1.000	1.000
222	1.000	1.000	1.000	1.000	1.000	1.000
223	1.000	1.000	1.000	1.000	1.000	1.000
224	1.000	1.000	1.000	1.000	1.000	1.000
225	1.000	1.000	1.000	1.000	1.000	1.000
226	1.000	1.000	1.000	1.000	1.000	1.000
227	1.000	1.000	1.000	1.000	1.000	1.000
228	1.000	1.000	1.000	1.000	1.000	1.000
229	1.000	1.000	1.000	1.000	1.000	1.000
230	1.000	1.000	1.000	1.000	1.000	1.000
231	1.000	1.000	1.000	1.000	1.000	1.000
232	1.000	1.000	1.000	1.000	1.000	1.000
233	1.000	1.000	1.000	1.000	1.000	1.000
234	1.000	1.000	1.000	1.000	1.000	1.000
235	1.000	1.000	1.000	1.000	1.000	1.000
236	1.000	1.000	1.000	1.000	1.000	1.000
237	1.000	1.000	1.000	1.000	1.000	1.000
238	1.000	1.000	1.000	1.000	1.000	1.000
239	1.000	1.000	1.000	1.000	1.000	1.000
240	1.000	1.000	1.000	1.000	1.000	1.000
241	1.000	1.000	1.000	1.000	1.000	1.000
242	1.000	1.000	1.000	1.000	1.000	1.000
243	1.000	1.000	1.000	1.000	1.000	1.000
244	1.000	1.000	1.000	1.000	1.000	1.000
245	1.000	1.000	1.000	1.000	1.000	1.000
246	1.000	1.000	1.000	1.000	1.000	1.000
247	1.000	1.000	1.000	1.000	1.000	1.000
248	1.000	1.000	1.000	1.000	1.000	1.000
249	1.000	1.000	1.000	1.000	1.000	1.000
250	1.000	1.000	1.000	1.000	1.000	1.000
251	1.000	1.000	1.000	1.000	1.000	1.000
252	1.000	1.000	1.000	1.000	1.000	1.000
253	1.000	1.000	1.000	1.000	1.000	1.000
254	1.000	1.000	1.000	1.000	1.000	1.000
255	1.000	1.000	1.000	1.000	1.000	1.000
256	1.000	1.000	1.000	1.000	1.000	1.000
257	1.000	1.000	1.000	1.000	1.000	1.000
258	1.000	1.000	1.000	1.000	1.000	1.000
259	1.000	1.000	1.000	1.000	1.000	1.000
260	1.000	1.000	1.000	1.000	1.000	1.000

261	1.000	1.000	1.000	1.000	1.000	1.000
262	1.000	1.000	1.000	1.000	1.000	1.000
263	1.000	1.000	1.000	1.000	1.000	1.000
264	1.000	1.000	1.000	1.000	1.000	1.000
265	1.000	1.000	1.000	1.000	1.000	1.000
266	1.000	1.000	1.000	1.000	1.000	1.000
267	1.000	1.000	1.000	1.000	1.000	1.000
268	1.000	1.000	1.000	1.000	1.000	1.000
269	1.000	1.000	1.000	1.000	1.000	1.000
270	1.000	1.000	1.000	1.000	1.000	1.000
271	1.000	1.000	1.000	1.000	1.000	1.000
272	1.000	1.000	1.000	1.000	1.000	1.000
273	1.000	1.000	1.000	1.000	1.000	1.000
274	1.000	1.000	1.000	1.000	1.000	1.000
275	1.000	1.000	1.000	1.000	1.000	1.000
276	1.000	1.000	1.000	1.000	1.000	1.000
277	1.000	1.000	1.000	1.000	1.000	1.000
278	1.000	1.000	1.000	1.000	1.000	1.000
279	1.000	1.000	1.000	1.000	1.000	1.000
280	1.000	1.000	1.000	1.000	1.000	1.000
281	1.000	1.000	1.000	1.000	1.000	1.000
282	1.000	1.000	1.000	1.000	1.000	1.000
283	1.000	1.000	1.000	1.000	1.000	1.000
284	1.000	1.000	1.000	1.000	1.000	1.000
285	1.000	1.000	1.000	1.000	1.000	1.000
286	1.000	1.000	1.000	1.000	1.000	1.000
287	1.000	1.000	1.000	1.000	1.000	1.000
288	1.000	1.000	1.000	1.000	1.000	1.000
289	1.000	1.000	1.000	1.000	1.000	1.000
290	1.000	1.000	1.000	1.000	1.000	1.000
291	1.000	1.000	1.000	1.000	1.000	1.000
292	1.000	1.000	1.000	1.000	1.000	1.000
293	1.000	1.000	1.000	1.000	1.000	1.000
294	1.000	1.000	1.000	1.000	1.000	1.000
295	1.000	1.000	1.000	1.000	1.000	1.000
296	1.000	1.000	1.000	1.000	1.000	1.000
297	1.000	1.000	1.000	1.000	1.000	1.000
298	1.000	1.000	1.000	1.000	1.000	1.000
299	1.000	1.000	1.000	1.000	1.000	1.000
300	1.000	1.000	1.000	1.000	1.000	1.000
301	1.000	1.000	1.000	1.000	1.000	1.000
302	1.000	1.000	1.000	1.000	1.000	1.000

303	1.000	1.000	1.000	1.000	1.000	1.000
304	1.000	1.000	1.000	1.000	1.000	1.000
305	1.000	1.000	1.000	1.000	1.000	1.000
306	1.000	1.000	1.000	1.000	1.000	1.000
307	1.000	1.000	1.000	1.000	1.000	1.000
308	1.000	1.000	1.000	1.000	1.000	1.000
309	1.000	1.000	1.000	1.000	1.000	1.000
310	1.000	1.000	1.000	1.000	1.000	1.000
311	1.000	1.000	1.000	1.000	1.000	1.000
312	1.000	1.000	1.000	1.000	1.000	1.000
313	1.000	1.000	1.000	1.000	1.000	1.000
314	1.000	1.000	1.000	1.000	1.000	1.000
315	1.000	1.000	1.000	1.000	1.000	1.000
316	1.000	1.000	1.000	1.000	1.000	1.000
317	1.000	1.000	1.000	1.000	1.000	1.000
318	1.000	1.000	1.000	1.000	1.000	1.000
319	1.000	1.000	1.000	1.000	1.000	1.000
320	1.000	1.000	1.000	1.000	1.000	1.000
321	1.000	1.000	1.000	1.000	1.000	1.000
322	1.000	1.000	1.000	1.000	1.000	1.000
323	1.000	1.000	1.000	1.000	1.000	1.000
324	1.000	1.000	1.000	1.000	1.000	1.000
325	1.000	1.000	1.000	1.000	1.000	1.000
326	1.000	1.000	1.000	1.000	1.000	1.000
327	1.000	1.000	1.000	1.000	1.000	1.000
328	1.000	1.000	1.000	1.000	1.000	1.000
329	1.000	1.000	1.000	1.000	1.000	1.000
330	1.000	1.000	1.000	1.000	1.000	1.000
331	1.000	1.000	1.000	1.000	1.000	1.000
332	1.000	1.000	1.000	1.000	1.000	1.000
333	1.000	1.000	1.000	1.000	1.000	1.000
334	1.000	1.000	1.000	1.000	1.000	1.000
335	1.000	1.000	1.000	1.000	1.000	1.000
336	1.000	1.000	1.000	1.000	1.000	1.000
337	1.000	1.000	1.000	1.000	1.000	1.000
338	1.000	1.000	1.000	1.000	1.000	1.000
339	1.000	1.000	1.000	1.000	1.000	1.000
340	1.000	1.000	1.000	1.000	1.000	1.000
341	1.000	1.000	1.000	1.000	1.000	1.000
342	1.000	1.000	1.000	1.000	1.000	1.000
343	1.000	1.000	1.000	1.000	1.000	1.000
344	1.000	1.000	1.000	1.000	1.000	1.000

345	1.000	1.000	1.000	1.000	1.000	1.000
346	1.000	1.000	1.000	1.000	1.000	1.000
347	1.000	1.000	1.000	1.000	1.000	1.000
348	1.000	1.000	1.000	1.000	1.000	1.000
349	1.000	1.000	1.000	1.000	1.000	1.000
350	1.000	1.000	1.000	1.000	1.000	1.000
351	1.000	1.000	1.000	1.000	1.000	1.000
352	1.000	1.000	1.000	1.000	1.000	1.000
353	1.000	1.000	1.000	1.000	1.000	1.000
354	1.000	1.000	1.000	1.000	1.000	1.000
355	1.000	1.000	1.000	1.000	1.000	1.000
356	1.000	1.000	1.000	1.000	1.000	1.000
357	1.000	1.000	1.000	1.000	1.000	1.000
358	1.000	1.000	1.000	1.000	1.000	1.000
359	1.000	1.000	1.000	1.000	1.000	1.000
360	1.000	1.000	1.000	1.000	1.000	1.000
361	1.000	1.000	1.000	1.000	1.000	1.000
362	1.000	1.000	1.000	1.000	1.000	1.000
363	1.000	1.000	1.000	1.000	1.000	1.000
364	1.000	1.000	1.000	1.000	1.000	1.000
365	1.000	1.000	1.000	1.000	1.000	1.000
366	1.000	1.000	1.000	1.000	1.000	1.000
367	1.000	1.000	1.000	1.000	1.000	1.000
368	1.000	1.000	1.000	1.000	1.000	1.000
369	1.000	1.000	1.000	1.000	1.000	1.000
370	1.000	1.000	1.000	1.000	1.000	1.000
371	1.000	1.000	1.000	1.000	1.000	1.000
372	1.000	1.000	1.000	1.000	1.000	1.000
373	1.000	1.000	1.000	1.000	1.000	1.000
374	1.000	1.000	1.000	1.000	1.000	1.000
375	1.000	1.000	1.000	1.000	1.000	1.000
376	1.000	1.000	1.000	1.000	1.000	1.000
377	1.000	1.000	1.000	1.000	1.000	1.000
378	1.000	1.000	1.000	1.000	1.000	1.000
379	1.000	1.000	1.000	1.000	1.000	1.000
380	1.000	1.000	1.000	1.000	1.000	1.000
381	1.000	1.000	1.000	1.000	1.000	1.000
382	1.000	1.000	1.000	1.000	1.000	1.000
383	1.000	1.000	1.000	1.000	1.000	1.000
384	1.000	1.000	1.000	1.000	1.000	1.000
385	1.000	1.000	1.000	1.000	1.000	1.000
386	1.000	1.000	1.000	1.000	1.000	1.000

387	1.000	1.000	1.000	1.000	1.000	1.000
388	1.000	1.000	1.000	1.000	1.000	1.000
389	1.000	1.000	1.000	1.000	1.000	1.000
390	1.000	1.000	1.000	1.000	1.000	1.000
391	1.000	1.000	1.000	1.000	1.000	1.000
392	1.000	1.000	1.000	1.000	1.000	1.000
393	1.000	1.000	1.000	1.000	1.000	1.000
394	1.000	1.000	1.000	1.000	1.000	1.000
395	1.000	1.000	1.000	1.000	1.000	1.000
396	1.000	1.000	1.000	1.000	1.000	1.000
397	1.000	1.000	1.000	1.000	1.000	1.000
398	1.000	1.000	1.000	1.000	1.000	1.000
399	1.000	1.000	1.000	1.000	1.000	1.000
400	1.000	1.000	1.000	1.000	1.000	1.000
401	1.000	1.000	1.000	1.000	1.000	1.000
402	1.000	1.000	1.000	1.000	1.000	1.000
403	1.000	1.000	1.000	1.000	1.000	1.000
404	1.000	1.000	1.000	1.000	1.000	1.000
405	1.000	1.000	1.000	1.000	1.000	1.000
406	1.000	1.000	1.000	1.000	1.000	1.000
407	1.000	1.000	1.000	1.000	1.000	1.000
408	1.000	1.000	1.000	1.000	1.000	1.000
409	1.000	1.000	1.000	1.000	1.000	1.000
410	1.000	1.000	1.000	1.000	1.000	1.000
411	1.000	1.000	1.000	1.000	1.000	1.000
412	1.000	1.000	1.000	1.000	1.000	1.000
413	1.000	1.000	1.000	1.000	1.000	1.000
414	1.000	1.000	1.000	1.000	1.000	1.000
415	1.000	1.000	1.000	1.000	1.000	1.000
416	1.000	1.000	1.000	1.000	1.000	1.000
417	1.000	1.000	1.000	1.000	1.000	1.000
418	1.000	1.000	1.000	1.000	1.000	1.000
419	1.000	1.000	1.000	1.000	1.000	1.000
420	1.000	1.000	1.000	1.000	1.000	1.000
421	1.000	1.000	1.000	1.000	1.000	1.000
422	1.000	1.000	1.000	1.000	1.000	1.000
423	1.000	1.000	1.000	1.000	1.000	1.000
424	1.000	1.000	1.000	1.000	1.000	1.000
425	1.000	1.000	1.000	1.000	1.000	1.000
426	1.000	1.000	1.000	1.000	1.000	1.000
427	1.000	1.000	1.000	1.000	1.000	1.000
428	1.000	1.000	1.000	1.000	1.000	1.000

429	1.000	1.000	1.000	1.000	1.000	1.000
430	1.000	1.000	1.000	1.000	1.000	1.000
431	1.000	1.000	1.000	1.000	1.000	1.000
432	1.000	1.000	1.000	1.000	1.000	1.000
433	1.000	1.000	1.000	1.000	1.000	1.000
434	1.000	1.000	1.000	1.000	1.000	1.000
435	1.000	1.000	1.000	1.000	1.000	1.000
436	1.000	1.000	1.000	1.000	1.000	1.000
437	1.000	1.000	1.000	1.000	1.000	1.000
438	1.000	1.000	1.000	1.000	1.000	1.000
439	1.000	1.000	1.000	1.000	1.000	1.000
440	1.000	1.000	1.000	1.000	1.000	1.000
441	1.000	1.000	1.000	1.000	1.000	1.000
442	1.000	1.000	1.000	1.000	1.000	1.000
443	1.000	1.000	1.000	1.000	1.000	1.000
444	1.000	1.000	1.000	1.000	1.000	1.000
445	1.000	1.000	1.000	1.000	1.000	1.000
446	1.000	1.000	1.000	1.000	1.000	1.000
447	1.000	1.000	1.000	1.000	1.000	1.000
448	1.000	1.000	1.000	1.000	1.000	1.000
449	1.000	1.000	1.000	1.000	1.000	1.000
450	1.000	1.000	1.000	1.000	1.000	1.000
451	1.000	1.000	1.000	1.000	1.000	1.000
452	1.000	1.000	1.000	1.000	1.000	1.000
453	1.000	1.000	1.000	1.000	1.000	1.000
454	1.000	1.000	1.000	1.000	1.000	1.000
455	1.000	1.000	1.000	1.000	1.000	1.000
456	1.000	1.000	1.000	1.000	1.000	1.000
457	1.000	1.000	1.000	1.000	1.000	1.000
458	1.000	1.000	1.000	1.000	1.000	1.000
459	1.000	1.000	1.000	1.000	1.000	1.000
460	1.000	1.000	1.000	1.000	1.000	1.000
461	1.000	1.000	1.000	1.000	1.000	1.000
462	1.000	1.000	1.000	1.000	1.000	1.000
463	1.000	1.000	1.000	1.000	1.000	1.000
464	1.000	1.000	1.000	1.000	1.000	1.000
465	1.000	1.000	1.000	1.000	1.000	1.000
466	1.000	1.000	1.000	1.000	1.000	1.000
467	1.000	1.000	1.000	1.000	1.000	1.000
468	1.000	1.000	1.000	1.000	1.000	1.000
469	1.000	1.000	1.000	1.000	1.000	1.000
470	1.000	1.000	1.000	1.000	1.000	1.000

471	1.000	1.000	1.000	1.000	1.000	1.000
472	1.000	1.000	1.000	1.000	1.000	1.000
473	1.000	1.000	1.000	1.000	1.000	1.000
474	1.000	1.000	1.000	1.000	1.000	1.000
475	1.000	1.000	1.000	1.000	1.000	1.000
476	1.000	1.000	1.000	1.000	1.000	1.000
477	1.000	1.000	1.000	1.000	1.000	1.000
478	1.000	1.000	1.000	1.000	1.000	1.000
479	1.000	1.000	1.000	1.000	1.000	1.000
480	1.000	1.000	1.000	1.000	1.000	1.000
481	1.000	1.000	1.000	1.000	1.000	1.000
482	1.000	1.000	1.000	1.000	1.000	1.000
483	1.000	1.000	1.000	1.000	1.000	1.000
484	1.000	1.000	1.000	1.000	1.000	1.000
485	1.000	1.000	1.000	1.000	1.000	1.000
486	1.000	1.000	1.000	1.000	1.000	1.000
487	1.000	1.000	1.000	1.000	1.000	1.000
488	1.000	1.000	1.000	1.000	1.000	1.000
489	1.000	1.000	1.000	1.000	1.000	1.000
490	1.000	1.000	1.000	1.000	1.000	1.000
491	1.000	1.000	1.000	1.000	1.000	1.000
492	1.000	1.000	1.000	1.000	1.000	1.000
493	1.000	1.000	1.000	1.000	1.000	1.000
494	1.000	1.000	1.000	1.000	1.000	1.000
495	1.000	1.000	1.000	1.000	1.000	1.000
496	1.000	1.000	1.000	1.000	1.000	1.000
497	1.000	1.000	1.000	1.000	1.000	1.000
498	1.000	1.000	1.000	1.000	1.000	1.000
499	1.000	1.000	1.000	1.000	1.000	1.000
500	1.000	1.000	1.000	1.000	1.000	1.000
501	1.000	1.000	1.000	1.000	1.000	1.000
502	1.000	1.000	1.000	1.000	1.000	1.000
503	1.000	1.000	1.000	1.000	1.000	1.000
504	1.000	1.000	1.000	1.000	1.000	1.000
505	1.000	1.000	1.000	1.000	1.000	1.000
506	1.000	1.000	1.000	1.000	1.000	1.000
507	1.000	1.000	1.000	1.000	1.000	1.000
508	1.000	1.000	1.000	1.000	1.000	1.000
509	1.000	1.000	1.000	1.000	1.000	1.000
510	1.000	1.000	1.000	1.000	1.000	1.000
511	1.000	1.000	1.000	1.000	1.000	1.000
512	1.000	1.000	1.000	1.000	1.000	1.000

513	1.000	1.000	1.000	1.000	1.000	1.000
514	1.000	1.000	1.000	1.000	1.000	1.000
515	1.000	1.000	1.000	1.000	1.000	1.000
516	1.000	1.000	1.000	1.000	1.000	1.000
517	1.000	1.000	1.000	1.000	1.000	1.000
518	1.000	1.000	1.000	1.000	1.000	1.000
519	1.000	1.000	1.000	1.000	1.000	1.000
520	1.000	1.000	1.000	1.000	1.000	1.000
521	1.000	1.000	1.000	1.000	1.000	1.000
522	1.000	1.000	1.000	1.000	1.000	1.000
523	1.000	1.000	1.000	1.000	1.000	1.000
524	1.000	1.000	1.000	1.000	1.000	1.000
525	1.000	1.000	1.000	1.000	1.000	1.000
526	1.000	1.000	1.000	1.000	1.000	1.000
527	1.000	1.000	1.000	1.000	1.000	1.000
528	1.000	1.000	1.000	1.000	1.000	1.000
529	1.000	1.000	1.000	1.000	1.000	1.000
530	1.000	1.000	1.000	1.000	1.000	1.000
531	1.000	1.000	1.000	1.000	1.000	1.000
532	1.000	1.000	1.000	1.000	1.000	1.000
533	1.000	1.000	1.000	1.000	1.000	1.000
534	1.000	1.000	1.000	1.000	1.000	1.000
535	1.000	1.000	1.000	1.000	1.000	1.000
536	1.000	1.000	1.000	1.000	1.000	1.000
537	1.000	1.000	1.000	1.000	1.000	1.000
538	1.000	1.000	1.000	1.000	1.000	1.000
539	1.000	1.000	1.000	1.000	1.000	1.000
540	1.000	1.000	1.000	1.000	1.000	1.000
541	1.000	1.000	1.000	1.000	1.000	1.000
542	1.000	1.000	1.000	1.000	1.000	1.000
543	1.000	1.000	1.000	1.000	1.000	1.000
544	1.000	1.000	1.000	1.000	1.000	1.000
545	1.000	1.000	1.000	1.000	1.000	1.000
546	1.000	1.000	1.000	1.000	1.000	1.000
547	1.000	1.000	1.000	1.000	1.000	1.000
548	1.000	1.000	1.000	1.000	1.000	1.000
549	1.000	1.000	1.000	1.000	1.000	1.000
550	1.000	1.000	1.000	1.000	1.000	1.000
551	1.000	1.000	1.000	1.000	1.000	1.000
552	1.000	1.000	1.000	1.000	1.000	1.000
553	1.000	1.000	1.000	1.000	1.000	1.000
554	1.000	1.000	1.000	1.000	1.000	1.000

555	1.000	1.000	1.000	1.000	1.000	1.000
556	1.000	1.000	1.000	1.000	1.000	1.000
557	1.000	1.000	1.000	1.000	1.000	1.000
558	1.000	1.000	1.000	1.000	1.000	1.000
559	1.000	1.000	1.000	1.000	1.000	1.000
560	1.000	1.000	1.000	1.000	1.000	1.000
561	1.000	1.000	1.000	1.000	1.000	1.000
562	1.000	1.000	1.000	1.000	1.000	1.000
563	1.000	1.000	1.000	1.000	1.000	1.000
564	1.000	1.000	1.000	1.000	1.000	1.000
565	1.000	1.000	1.000	1.000	1.000	1.000
566	1.000	1.000	1.000	1.000	1.000	1.000
567	1.000	1.000	1.000	1.000	1.000	1.000
568	1.000	1.000	1.000	1.000	1.000	1.000
569	1.000	1.000	1.000	1.000	1.000	1.000
570	1.000	1.000	1.000	1.000	1.000	1.000
571	1.000	1.000	1.000	1.000	1.000	1.000
572	1.000	1.000	1.000	1.000	1.000	1.000
573	1.000	1.000	1.000	1.000	1.000	1.000
574	1.000	1.000	1.000	1.000	1.000	1.000
575	1.000	1.000	1.000	1.000	1.000	1.000
576	1.000	1.000	1.000	1.000	1.000	1.000
577	1.000	1.000	1.000	1.000	1.000	1.000
578	1.000	1.000	1.000	1.000	1.000	1.000
579	1.000	1.000	1.000	1.000	1.000	1.000
580	1.000	1.000	1.000	1.000	1.000	1.000
581	1.000	1.000	1.000	1.000	1.000	1.000
582	1.000	1.000	1.000	1.000	1.000	1.000
583	1.000	1.000	1.000	1.000	1.000	1.000
584	1.000	1.000	1.000	1.000	1.000	1.000
585	1.000	1.000	1.000	1.000	1.000	1.000
586	1.000	1.000	1.000	1.000	1.000	1.000
587	1.000	1.000	1.000	1.000	1.000	1.000
588	1.000	1.000	1.000	1.000	1.000	1.000
589	1.000	1.000	1.000	1.000	1.000	1.000
590	1.000	1.000	1.000	1.000	1.000	1.000
591	1.000	1.000	1.000	1.000	1.000	1.000
592	1.000	1.000	1.000	1.000	1.000	1.000
593	1.000	1.000	1.000	1.000	1.000	1.000
594	1.000	1.000	1.000	1.000	1.000	1.000
595	1.000	1.000	1.000	1.000	1.000	1.000
596	1.000	1.000	1.000	1.000	1.000	1.000

597	1.000	1.000	1.000	1.000	1.000	1.000
598	1.000	1.000	1.000	1.000	1.000	1.000
599	1.000	1.000	1.000	1.000	1.000	1.000
600	1.000	1.000	1.000	1.000	1.000	1.000
601	1.000	1.000	1.000	1.000	1.000	1.000
602	1.000	1.000	1.000	1.000	1.000	1.000
603	1.000	1.000	1.000	1.000	1.000	1.000
604	1.000	1.000	1.000	1.000	1.000	1.000
605	1.000	1.000	1.000	1.000	1.000	1.000
606	1.000	1.000	1.000	1.000	1.000	1.000
607	1.000	1.000	1.000	1.000	1.000	1.000
608	1.000	1.000	1.000	1.000	1.000	1.000
609	1.000	1.000	1.000	1.000	1.000	1.000
610	1.000	1.000	1.000	1.000	1.000	1.000
611	1.000	1.000	1.000	1.000	1.000	1.000
612	1.000	1.000	1.000	1.000	1.000	1.000
613	1.000	1.000	1.000	1.000	1.000	1.000
614	1.000	1.000	1.000	1.000	1.000	1.000
615	1.000	1.000	1.000	1.000	1.000	1.000
616	1.000	1.000	1.000	1.000	1.000	1.000
617	1.000	1.000	1.000	1.000	1.000	1.000
618	1.000	1.000	1.000	1.000	1.000	1.000
619	1.000	1.000	1.000	1.000	1.000	1.000
620	1.000	1.000	1.000	1.000	1.000	1.000
621	1.000	1.000	1.000	1.000	1.000	1.000
622	1.000	1.000	1.000	1.000	1.000	1.000
623	1.000	1.000	1.000	1.000	1.000	1.000
624	1.000	1.000	1.000	1.000	1.000	1.000
625	1.000	1.000	1.000	1.000	1.000	1.000
626	1.000	1.000	1.000	1.000	1.000	1.000
627	1.000	1.000	1.000	1.000	1.000	1.000
628	1.000	1.000	1.000	1.000	1.000	1.000
629	1.000	1.000	1.000	1.000	1.000	1.000
630	1.000	1.000	1.000	1.000	1.000	1.000
631	1.000	1.000	1.000	1.000	1.000	1.000
632	1.000	1.000	1.000	1.000	1.000	1.000
633	1.000	1.000	1.000	1.000	1.000	1.000
634	1.000	1.000	1.000	1.000	1.000	1.000
635	1.000	1.000	1.000	1.000	1.000	1.000
636	1.000	1.000	1.000	1.000	1.000	1.000
637	1.000	1.000	1.000	1.000	1.000	1.000
638	1.000	1.000	1.000	1.000	1.000	1.000

639	1.000	1.000	1.000	1.000	1.000	1.000
640	1.000	1.000	1.000	1.000	1.000	1.000
641	1.000	1.000	1.000	1.000	1.000	1.000
642	1.000	1.000	1.000	1.000	1.000	1.000
643	1.000	1.000	1.000	1.000	1.000	1.000
644	1.000	1.000	1.000	1.000	1.000	1.000
645	1.000	1.000	1.000	1.000	1.000	1.000
646	1.000	1.000	1.000	1.000	1.000	1.000
647	1.000	1.000	1.000	1.000	1.000	1.000
648	1.000	1.000	1.000	1.000	1.000	1.000
649	1.000	1.000	1.000	1.000	1.000	1.000
650	1.000	1.000	1.000	1.000	1.000	1.000
651	1.000	1.000	1.000	1.000	1.000	1.000
652	1.000	1.000	1.000	1.000	1.000	1.000
653	1.000	1.000	1.000	1.000	1.000	1.000
654	1.000	1.000	1.000	1.000	1.000	1.000
655	1.000	1.000	1.000	1.000	1.000	1.000
656	1.000	1.000	1.000	1.000	1.000	1.000
657	1.000	1.000	1.000	1.000	1.000	1.000
658	1.000	1.000	1.000	1.000	1.000	1.000
659	1.000	1.000	1.000	1.000	1.000	1.000
660	1.000	1.000	1.000	1.000	1.000	1.000
661	1.000	1.000	1.000	1.000	1.000	1.000
662	1.000	1.000	1.000	1.000	1.000	1.000
663	1.000	1.000	1.000	1.000	1.000	1.000
664	1.000	1.000	1.000	1.000	1.000	1.000
665	1.000	1.000	1.000	1.000	1.000	1.000
666	1.000	1.000	1.000	1.000	1.000	1.000
667	1.000	1.000	1.000	1.000	1.000	1.000
668	1.000	1.000	1.000	1.000	1.000	1.000
669	1.000	1.000	1.000	1.000	1.000	1.000
670	1.000	1.000	1.000	1.000	1.000	1.000
671	1.000	1.000	1.000	1.000	1.000	1.000
672	1.000	1.000	1.000	1.000	1.000	1.000
673	1.000	1.000	1.000	1.000	1.000	1.000
674	1.000	1.000	1.000	1.000	1.000	1.000
675	1.000	1.000	1.000	1.000	1.000	1.000
676	1.000	1.000	1.000	1.000	1.000	1.000
677	1.000	1.000	1.000	1.000	1.000	1.000
678	1.000	1.000	1.000	1.000	1.000	1.000
679	1.000	1.000	1.000	1.000	1.000	1.000
680	1.000	1.000	1.000	1.000	1.000	1.000

681	1.000	1.000	1.000	1.000	1.000	1.000
682	1.000	1.000	1.000	1.000	1.000	1.000
683	1.000	1.000	1.000	1.000	1.000	1.000
684	1.000	1.000	1.000	1.000	1.000	1.000
685	1.000	1.000	1.000	1.000	1.000	1.000
686	1.000	1.000	1.000	1.000	1.000	1.000
687	1.000	1.000	1.000	1.000	1.000	1.000
688	1.000	1.000	1.000	1.000	1.000	1.000
689	1.000	1.000	1.000	1.000	1.000	1.000
690	1.000	1.000	1.000	1.000	1.000	1.000
691	1.000	1.000	1.000	1.000	1.000	1.000
692	1.000	1.000	1.000	1.000	1.000	1.000
693	1.000	1.000	1.000	1.000	1.000	1.000
694	1.000	1.000	1.000	1.000	1.000	1.000
695	1.000	1.000	1.000	1.000	1.000	1.000
696	1.000	1.000	1.000	1.000	1.000	1.000
697	1.000	1.000	1.000	1.000	1.000	1.000
698	1.000	1.000	1.000	1.000	1.000	1.000
699	1.000	1.000	1.000	1.000	1.000	1.000
700	1.000	1.000	1.000	1.000	1.000	1.000
701	1.000	1.000	1.000	1.000	1.000	1.000
702	1.000	1.000	1.000	1.000	1.000	1.000
703	1.000	1.000	1.000	1.000	1.000	1.000
704	1.000	1.000	1.000	1.000	1.000	1.000
705	1.000	1.000	1.000	1.000	1.000	1.000
706	1.000	1.000	1.000	1.000	1.000	1.000
707	1.000	1.000	1.000	1.000	1.000	1.000
708	1.000	1.000	1.000	1.000	1.000	1.000
709	1.000	1.000	1.000	1.000	1.000	1.000
710	1.000	1.000	1.000	1.000	1.000	1.000
711	1.000	1.000	1.000	1.000	1.000	1.000
712	1.000	1.000	1.000	1.000	1.000	1.000
713	1.000	1.000	1.000	1.000	1.000	1.000
714	1.000	1.000	1.000	1.000	1.000	1.000
715	1.000	1.000	1.000	1.000	1.000	1.000
716	1.000	1.000	1.000	1.000	1.000	1.000
717	1.000	1.000	1.000	1.000	1.000	1.000
718	1.000	1.000	1.000	1.000	1.000	1.000
719	1.000	1.000	1.000	1.000	1.000	1.000
720	1.000	1.000	1.000	1.000	1.000	1.000
721	1.000	1.000	1.000	1.000	1.000	1.000
722	1.000	1.000	1.000	1.000	1.000	1.000

723	1.000	1.000	1.000	1.000	1.000	1.000
724	1.000	1.000	1.000	1.000	1.000	1.000
725	1.000	1.000	1.000	1.000	1.000	1.000
726	1.000	1.000	1.000	1.000	1.000	1.000
727	1.000	1.000	1.000	1.000	1.000	1.000
728	1.000	1.000	1.000	1.000	1.000	1.000
729	1.000	1.000	1.000	1.000	1.000	1.000
730	1.000	1.000	1.000	1.000	1.000	1.000
731	1.000	1.000	1.000	1.000	1.000	1.000
732	1.000	1.000	1.000	1.000	1.000	1.000
733	1.000	1.000	1.000	1.000	1.000	1.000
734	1.000	1.000	1.000	1.000	1.000	1.000
735	1.000	1.000	1.000	1.000	1.000	1.000
736	1.000	1.000	1.000	1.000	1.000	1.000
737	1.000	1.000	1.000	1.000	1.000	1.000
738	1.000	1.000	1.000	1.000	1.000	1.000
739	1.000	1.000	1.000	1.000	1.000	1.000
740	1.000	1.000	1.000	1.000	1.000	1.000
741	1.000	1.000	1.000	1.000	1.000	1.000
742	1.000	1.000	1.000	1.000	1.000	1.000
743	1.000	1.000	1.000	1.000	1.000	1.000
744	1.000	1.000	1.000	1.000	1.000	1.000
745	1.000	1.000	1.000	1.000	1.000	1.000
746	1.000	1.000	1.000	1.000	1.000	1.000
747	1.000	1.000	1.000	1.000	1.000	1.000
748	1.000	1.000	1.000	1.000	1.000	1.000
749	1.000	1.000	1.000	1.000	1.000	1.000
750	1.000	1.000	1.000	1.000	1.000	1.000
751	1.000	1.000	1.000	1.000	1.000	1.000
752	1.000	1.000	1.000	1.000	1.000	1.000
753	1.000	1.000	1.000	1.000	1.000	1.000
754	1.000	1.000	1.000	1.000	1.000	1.000
755	1.000	1.000	1.000	1.000	1.000	1.000
756	1.000	1.000	1.000	1.000	1.000	1.000
757	1.000	1.000	1.000	1.000	1.000	1.000
758	1.000	1.000	1.000	1.000	1.000	1.000
759	1.000	1.000	1.000	1.000	1.000	1.000
760	1.000	1.000	1.000	1.000	1.000	1.000
761	1.000	1.000	1.000	1.000	1.000	1.000
762	1.000	1.000	1.000	1.000	1.000	1.000
763	1.000	1.000	1.000	1.000	1.000	1.000
764	1.000	1.000	1.000	1.000	1.000	1.000

765	1.000	1.000	1.000	1.000	1.000	1.000
766	1.000	1.000	1.000	1.000	1.000	1.000
767	1.000	1.000	1.000	1.000	1.000	1.000
768	1.000	1.000	1.000	1.000	1.000	1.000
769	1.000	1.000	1.000	1.000	1.000	1.000
770	1.000	1.000	1.000	1.000	1.000	1.000
771	1.000	1.000	1.000	1.000	1.000	1.000
772	1.000	1.000	1.000	1.000	1.000	1.000
773	1.000	1.000	1.000	1.000	1.000	1.000
774	1.000	1.000	1.000	1.000	1.000	1.000
775	1.000	1.000	1.000	1.000	1.000	1.000
776	1.000	1.000	1.000	1.000	1.000	1.000
777	1.000	1.000	1.000	1.000	1.000	1.000
778	1.000	1.000	1.000	1.000	1.000	1.000
779	1.000	1.000	1.000	1.000	1.000	1.000
780	1.000	1.000	1.000	1.000	1.000	1.000
781	1.000	1.000	1.000	1.000	1.000	1.000
782	1.000	1.000	1.000	1.000	1.000	1.000
783	1.000	1.000	1.000	1.000	1.000	1.000
784	1.000	1.000	1.000	1.000	1.000	1.000
785	1.000	1.000	1.000	1.000	1.000	1.000
786	1.000	1.000	1.000	1.000	1.000	1.000
787	1.000	1.000	1.000	1.000	1.000	1.000
788	1.000	1.000	1.000	1.000	1.000	1.000
789	1.000	1.000	1.000	1.000	1.000	1.000
790	1.000	1.000	1.000	1.000	1.000	1.000
791	1.000	1.000	1.000	1.000	1.000	1.000
792	1.000	1.000	1.000	1.000	1.000	1.000
793	1.000	1.000	1.000	1.000	1.000	1.000
794	1.000	1.000	1.000	1.000	1.000	1.000
795	1.000	1.000	1.000	1.000	1.000	1.000
796	1.000	1.000	1.000	1.000	1.000	1.000
797	1.000	1.000	1.000	1.000	1.000	1.000
798	1.000	1.000	1.000	1.000	1.000	1.000
799	1.000	1.000	1.000	1.000	1.000	1.000
800	1.000	1.000	1.000	1.000	1.000	1.000
801	1.000	1.000	1.000	1.000	1.000	1.000
802	1.000	1.000	1.000	1.000	1.000	1.000
803	1.000	1.000	1.000	1.000	1.000	1.000
804	1.000	1.000	1.000	1.000	1.000	1.000
805	1.000	1.000	1.000	1.000	1.000	1.000
806	1.000	1.000	1.000	1.000	1.000	1.000

807	1.000	1.000	1.000	1.000	1.000	1.000
808	1.000	1.000	1.000	1.000	1.000	1.000
809	1.000	1.000	1.000	1.000	1.000	1.000
810	1.000	1.000	1.000	1.000	1.000	1.000
811	1.000	1.000	1.000	1.000	1.000	1.000
812	1.000	1.000	1.000	1.000	1.000	1.000
813	1.000	1.000	1.000	1.000	1.000	1.000
814	1.000	1.000	1.000	1.000	1.000	1.000
815	1.000	1.000	1.000	1.000	1.000	1.000
816	1.000	1.000	1.000	1.000	1.000	1.000
817	1.000	1.000	1.000	1.000	1.000	1.000
818	1.000	1.000	1.000	1.000	1.000	1.000
819	1.000	1.000	1.000	1.000	1.000	1.000
820	1.000	1.000	1.000	1.000	1.000	1.000
821	1.000	1.000	1.000	1.000	1.000	1.000
822	1.000	1.000	1.000	1.000	1.000	1.000
823	1.000	1.000	1.000	1.000	1.000	1.000
824	1.000	1.000	1.000	1.000	1.000	1.000
825	1.000	1.000	1.000	1.000	1.000	1.000
826	1.000	1.000	1.000	1.000	1.000	1.000
827	1.000	1.000	1.000	1.000	1.000	1.000
828	1.000	1.000	1.000	1.000	1.000	1.000
829	1.000	1.000	1.000	1.000	1.000	1.000
830	1.000	1.000	1.000	1.000	1.000	1.000
831	1.000	1.000	1.000	1.000	1.000	1.000
832	1.000	1.000	1.000	1.000	1.000	1.000
833	1.000	1.000	1.000	1.000	1.000	1.000
834	1.000	1.000	1.000	1.000	1.000	1.000
835	1.000	1.000	1.000	1.000	1.000	1.000
836	1.000	1.000	1.000	1.000	1.000	1.000
837	1.000	1.000	1.000	1.000	1.000	1.000
838	1.000	1.000	1.000	1.000	1.000	1.000
839	1.000	1.000	1.000	1.000	1.000	1.000
840	1.000	1.000	1.000	1.000	1.000	1.000
841	1.000	1.000	1.000	1.000	1.000	1.000
842	1.000	1.000	1.000	1.000	1.000	1.000
843	1.000	1.000	1.000	1.000	1.000	1.000
844	1.000	1.000	1.000	1.000	1.000	1.000
845	1.000	1.000	1.000	1.000	1.000	1.000
846	1.000	1.000	1.000	1.000	1.000	1.000
847	1.000	1.000	1.000	1.000	1.000	1.000
848	1.000	1.000	1.000	1.000	1.000	1.000

849	1.000	1.000	1.000	1.000	1.000	1.000
850	1.000	1.000	1.000	1.000	1.000	1.000
851	1.000	1.000	1.000	1.000	1.000	1.000
852	1.000	1.000	1.000	1.000	1.000	1.000
853	1.000	1.000	1.000	1.000	1.000	1.000
854	1.000	1.000	1.000	1.000	1.000	1.000
855	1.000	1.000	1.000	1.000	1.000	1.000
856	1.000	1.000	1.000	1.000	1.000	1.000
857	1.000	1.000	1.000	1.000	1.000	1.000
858	1.000	1.000	1.000	1.000	1.000	1.000
859	1.000	1.000	1.000	1.000	1.000	1.000
860	1.000	1.000	1.000	1.000	1.000	1.000
861	1.000	1.000	1.000	1.000	1.000	1.000
862	1.000	1.000	1.000	1.000	1.000	1.000
863	1.000	1.000	1.000	1.000	1.000	1.000
864	1.000	1.000	1.000	1.000	1.000	1.000
865	1.000	1.000	1.000	1.000	1.000	1.000
866	1.000	1.000	1.000	1.000	1.000	1.000
867	1.000	1.000	1.000	1.000	1.000	1.000
868	1.000	1.000	1.000	1.000	1.000	1.000
869	1.000	1.000	1.000	1.000	1.000	1.000
870	1.000	1.000	1.000	1.000	1.000	1.000
871	1.000	1.000	1.000	1.000	1.000	1.000
872	1.000	1.000	1.000	1.000	1.000	1.000
873	1.000	1.000	1.000	1.000	1.000	1.000
874	1.000	1.000	1.000	1.000	1.000	1.000
875	1.000	1.000	1.000	1.000	1.000	1.000
876	1.000	1.000	1.000	1.000	1.000	1.000
877	1.000	1.000	1.000	1.000	1.000	1.000
878	1.000	1.000	1.000	1.000	1.000	1.000
879	1.000	1.000	1.000	1.000	1.000	1.000
880	1.000	1.000	1.000	1.000	1.000	1.000
881	1.000	1.000	1.000	1.000	1.000	1.000
882	1.000	1.000	1.000	1.000	1.000	1.000
883	1.000	1.000	1.000	1.000	1.000	1.000
884	1.000	1.000	1.000	1.000	1.000	1.000
885	1.000	1.000	1.000	1.000	1.000	1.000
886	1.000	1.000	1.000	1.000	1.000	1.000
887	1.000	1.000	1.000	1.000	1.000	1.000
888	1.000	1.000	1.000	1.000	1.000	1.000
889	1.000	1.000	1.000	1.000	1.000	1.000
890	1.000	1.000	1.000	1.000	1.000	1.000

891	1.000	1.000	1.000	1.000	1.000	1.000
892	1.000	1.000	1.000	1.000	1.000	1.000
893	1.000	1.000	1.000	1.000	1.000	1.000
894	1.000	1.000	1.000	1.000	1.000	1.000
895	1.000	1.000	1.000	1.000	1.000	1.000
896	1.000	1.000	1.000	1.000	1.000	1.000
897	1.000	1.000	1.000	1.000	1.000	1.000
898	1.000	1.000	1.000	1.000	1.000	1.000
899	1.000	1.000	1.000	1.000	1.000	1.000
900	1.000	1.000	1.000	1.000	1.000	1.000
901	1.000	1.000	1.000	1.000	1.000	1.000
902	1.000	1.000	1.000	1.000	1.000	1.000
903	1.000	1.000	1.000	1.000	1.000	1.000
904	1.000	1.000	1.000	1.000	1.000	1.000
905	1.000	1.000	1.000	1.000	1.000	1.000
906	1.000	1.000	1.000	1.000	1.000	1.000
907	1.000	1.000	1.000	1.000	1.000	1.000
908	1.000	1.000	1.000	1.000	1.000	1.000
909	1.000	1.000	1.000	1.000	1.000	1.000
910	1.000	1.000	1.000	1.000	1.000	1.000
911	1.000	1.000	1.000	1.000	1.000	1.000
912	1.000	1.000	1.000	1.000	1.000	1.000
913	1.000	1.000	1.000	1.000	1.000	1.000
914	1.000	1.000	1.000	1.000	1.000	1.000
915	1.000	1.000	1.000	1.000	1.000	1.000
916	1.000	1.000	1.000	1.000	1.000	1.000
917	1.000	1.000	1.000	1.000	1.000	1.000
918	1.000	1.000	1.000	1.000	1.000	1.000
919	1.000	1.000	1.000	1.000	1.000	1.000
920	1.000	1.000	1.000	1.000	1.000	1.000
921	1.000	1.000	1.000	1.000	1.000	1.000
922	1.000	1.000	1.000	1.000	1.000	1.000
923	1.000	1.000	1.000	1.000	1.000	1.000
924	1.000	1.000	1.000	1.000	1.000	1.000
925	1.000	1.000	1.000	1.000	1.000	1.000
926	1.000	1.000	1.000	1.000	1.000	1.000
927	1.000	1.000	1.000	1.000	1.000	1.000
928	1.000	1.000	1.000	1.000	1.000	1.000
929	1.000	1.000	1.000	1.000	1.000	1.000
930	1.000	1.000	1.000	1.000	1.000	1.000
931	1.000	1.000	1.000	1.000	1.000	1.000
932	1.000	1.000	1.000	1.000	1.000	1.000

933	1.000	1.000	1.000	1.000	1.000	1.000
934	1.000	1.000	1.000	1.000	1.000	1.000
935	1.000	1.000	1.000	1.000	1.000	1.000
936	1.000	1.000	1.000	1.000	1.000	1.000
937	1.000	1.000	1.000	1.000	1.000	1.000
938	1.000	1.000	1.000	1.000	1.000	1.000
939	1.000	1.000	1.000	1.000	1.000	1.000
940	1.000	1.000	1.000	1.000	1.000	1.000
941	1.000	1.000	1.000	1.000	1.000	1.000
942	1.000	1.000	1.000	1.000	1.000	1.000
943	1.000	1.000	1.000	1.000	1.000	1.000
944	1.000	1.000	1.000	1.000	1.000	1.000
945	1.000	1.000	1.000	1.000	1.000	1.000
946	1.000	1.000	1.000	1.000	1.000	1.000
947	1.000	1.000	1.000	1.000	1.000	1.000
948	1.000	1.000	1.000	1.000	1.000	1.000
949	1.000	1.000	1.000	1.000	1.000	1.000
950	1.000	1.000	1.000	1.000	1.000	1.000
951	1.000	1.000	1.000	1.000	1.000	1.000
952	1.000	1.000	1.000	1.000	1.000	1.000
953	1.000	1.000	1.000	1.000	1.000	1.000
954	1.000	1.000	1.000	1.000	1.000	1.000
955	1.000	1.000	1.000	1.000	1.000	1.000
956	1.000	1.000	1.000	1.000	1.000	1.000
957	1.000	1.000	1.000	1.000	1.000	1.000
958	1.000	1.000	1.000	1.000	1.000	1.000
959	1.000	1.000	1.000	1.000	1.000	1.000
960	1.000	1.000	1.000	1.000	1.000	1.000
961	1.000	1.000	1.000	1.000	1.000	1.000
962	1.000	1.000	1.000	1.000	1.000	1.000
963	1.000	1.000	1.000	1.000	1.000	1.000
964	1.000	1.000	1.000	1.000	1.000	1.000
965	1.000	1.000	1.000	1.000	1.000	1.000
966	1.000	1.000	1.000	1.000	1.000	1.000
967	1.000	1.000	1.000	1.000	1.000	1.000
968	1.000	1.000	1.000	1.000	1.000	1.000
969	1.000	1.000	1.000	1.000	1.000	1.000
970	1.000	1.000	1.000	1.000	1.000	1.000
971	1.000	1.000	1.000	1.000	1.000	1.000
972	1.000	1.000	1.000	1.000	1.000	1.000
973	1.000	1.000	1.000	1.000	1.000	1.000
974	1.000	1.000	1.000	1.000	1.000	1.000

975	1.000	1.000	1.000	1.000	1.000	1.000
976	1.000	1.000	1.000	1.000	1.000	1.000
977	1.000	1.000	1.000	1.000	1.000	1.000
978	1.000	1.000	1.000	1.000	1.000	1.000
979	1.000	1.000	1.000	1.000	1.000	1.000
980	1.000	1.000	1.000	1.000	1.000	1.000
981	1.000	1.000	1.000	1.000	1.000	1.000
982	1.000	1.000	1.000	1.000	1.000	1.000
983	1.000	1.000	1.000	1.000	1.000	1.000
984	1.000	1.000	1.000	1.000	1.000	1.000
985	1.000	1.000	1.000	1.000	1.000	1.000
986	1.000	1.000	1.000	1.000	1.000	1.000
987	1.000	1.000	1.000	1.000	1.000	1.000
988	1.000	1.000	1.000	1.000	1.000	1.000
989	1.000	1.000	1.000	1.000	1.000	1.000
990	1.000	1.000	1.000	1.000	1.000	1.000
991	1.000	1.000	1.000	1.000	1.000	1.000
992	1.000	1.000	1.000	1.000	1.000	1.000
993	1.000	1.000	1.000	1.000	1.000	1.000
994	1.000	1.000	1.000	1.000	1.000	1.000
995	1.000	1.000	1.000	1.000	1.000	1.000
996	1.000	1.000	1.000	1.000	1.000	1.000
997	1.000	1.000	1.000	1.000	1.000	1.000
998	1.000	1.000	1.000	1.000	1.000	1.000
999	1.000	1.000	1.000	1.000	1.000	1.000
1000	1.000	1.000	1.000	1.000	1.000	1.000
1001	1.000	1.000	1.000	1.000	1.000	1.000
1002	1.000	1.000	1.000	1.000	1.000	1.000
1003	1.000	1.000	1.000	1.000	1.000	1.000
1004	1.000	1.000	1.000	1.000	1.000	1.000
1005	1.000	1.000	1.000	1.000	1.000	1.000
1006	1.000	1.000	1.000	1.000	1.000	1.000
1007	1.000	1.000	1.000	1.000	1.000	1.000
1008	1.000	1.000	1.000	1.000	1.000	1.000
1009	1.000	1.000	1.000	1.000	1.000	1.000
1010	1.000	1.000	1.000	1.000	1.000	1.000
1011	1.000	1.000	1.000	1.000	1.000	1.000
1012	1.000	1.000	1.000	1.000	1.000	1.000
1013	1.000	1.000	1.000	1.000	1.000	1.000
1014	1.000	1.000	1.000	1.000	1.000	1.000
1015	1.000	1.000	1.000	1.000	1.000	1.000
1016	1.000	1.000	1.000	1.000	1.000	1.000

1017	1.000	1.000	1.000	1.000	1.000	1.000
1018	1.000	1.000	1.000	1.000	1.000	1.000
1019	1.000	1.000	1.000	1.000	1.000	1.000
1020	1.000	1.000	1.000	1.000	1.000	1.000

(4) Support vector machine

Number of features	SN	SP	ACC	MCC	Precision	F1-measure
1	0.753	0.959	0.856	0.727	0.948	0.839
2	1.000	0.990	0.995	0.990	0.990	0.995
3	1.000	1.000	1.000	1.000	1.000	1.000
4	1.000	1.000	1.000	1.000	1.000	1.000
5	1.000	0.990	0.995	0.990	0.990	0.995
6	1.000	1.000	1.000	1.000	1.000	1.000
7	1.000	1.000	1.000	1.000	1.000	1.000
8	1.000	1.000	1.000	1.000	1.000	1.000
9	1.000	1.000	1.000	1.000	1.000	1.000
10	1.000	1.000	1.000	1.000	1.000	1.000
11	1.000	1.000	1.000	1.000	1.000	1.000
12	1.000	1.000	1.000	1.000	1.000	1.000
13	1.000	1.000	1.000	1.000	1.000	1.000
14	1.000	1.000	1.000	1.000	1.000	1.000
15	1.000	1.000	1.000	1.000	1.000	1.000
16	1.000	1.000	1.000	1.000	1.000	1.000
17	1.000	1.000	1.000	1.000	1.000	1.000
18	1.000	1.000	1.000	1.000	1.000	1.000
19	1.000	1.000	1.000	1.000	1.000	1.000
20	1.000	1.000	1.000	1.000	1.000	1.000
21	1.000	1.000	1.000	1.000	1.000	1.000
22	1.000	1.000	1.000	1.000	1.000	1.000
23	1.000	1.000	1.000	1.000	1.000	1.000
24	1.000	1.000	1.000	1.000	1.000	1.000
25	1.000	1.000	1.000	1.000	1.000	1.000
26	1.000	1.000	1.000	1.000	1.000	1.000
27	1.000	1.000	1.000	1.000	1.000	1.000
28	1.000	1.000	1.000	1.000	1.000	1.000
29	1.000	1.000	1.000	1.000	1.000	1.000
30	1.000	1.000	1.000	1.000	1.000	1.000
31	1.000	1.000	1.000	1.000	1.000	1.000
32	1.000	1.000	1.000	1.000	1.000	1.000
33	1.000	1.000	1.000	1.000	1.000	1.000
34	1.000	1.000	1.000	1.000	1.000	1.000
35	1.000	1.000	1.000	1.000	1.000	1.000

36	1.000	1.000	1.000	1.000	1.000	1.000
37	1.000	1.000	1.000	1.000	1.000	1.000
38	1.000	1.000	1.000	1.000	1.000	1.000
39	1.000	1.000	1.000	1.000	1.000	1.000
40	1.000	1.000	1.000	1.000	1.000	1.000
41	1.000	1.000	1.000	1.000	1.000	1.000
42	1.000	1.000	1.000	1.000	1.000	1.000
43	1.000	1.000	1.000	1.000	1.000	1.000
44	1.000	1.000	1.000	1.000	1.000	1.000
45	1.000	1.000	1.000	1.000	1.000	1.000
46	1.000	1.000	1.000	1.000	1.000	1.000
47	1.000	1.000	1.000	1.000	1.000	1.000
48	1.000	1.000	1.000	1.000	1.000	1.000
49	1.000	1.000	1.000	1.000	1.000	1.000
50	1.000	1.000	1.000	1.000	1.000	1.000
51	1.000	1.000	1.000	1.000	1.000	1.000
52	1.000	1.000	1.000	1.000	1.000	1.000
53	1.000	1.000	1.000	1.000	1.000	1.000
54	1.000	1.000	1.000	1.000	1.000	1.000
55	1.000	1.000	1.000	1.000	1.000	1.000
56	1.000	1.000	1.000	1.000	1.000	1.000
57	1.000	1.000	1.000	1.000	1.000	1.000
58	1.000	1.000	1.000	1.000	1.000	1.000
59	1.000	1.000	1.000	1.000	1.000	1.000
60	1.000	1.000	1.000	1.000	1.000	1.000
61	1.000	1.000	1.000	1.000	1.000	1.000
62	1.000	1.000	1.000	1.000	1.000	1.000
63	1.000	1.000	1.000	1.000	1.000	1.000
64	1.000	1.000	1.000	1.000	1.000	1.000
65	1.000	1.000	1.000	1.000	1.000	1.000
66	1.000	1.000	1.000	1.000	1.000	1.000
67	1.000	1.000	1.000	1.000	1.000	1.000
68	1.000	1.000	1.000	1.000	1.000	1.000
69	1.000	1.000	1.000	1.000	1.000	1.000
70	1.000	1.000	1.000	1.000	1.000	1.000
71	1.000	1.000	1.000	1.000	1.000	1.000
72	1.000	1.000	1.000	1.000	1.000	1.000
73	1.000	1.000	1.000	1.000	1.000	1.000
74	1.000	1.000	1.000	1.000	1.000	1.000
75	1.000	1.000	1.000	1.000	1.000	1.000
76	1.000	1.000	1.000	1.000	1.000	1.000
77	1.000	1.000	1.000	1.000	1.000	1.000

78	1.000	1.000	1.000	1.000	1.000	1.000
79	1.000	1.000	1.000	1.000	1.000	1.000
80	1.000	1.000	1.000	1.000	1.000	1.000
81	1.000	1.000	1.000	1.000	1.000	1.000
82	1.000	1.000	1.000	1.000	1.000	1.000
83	1.000	1.000	1.000	1.000	1.000	1.000
84	1.000	1.000	1.000	1.000	1.000	1.000
85	1.000	1.000	1.000	1.000	1.000	1.000
86	1.000	1.000	1.000	1.000	1.000	1.000
87	1.000	1.000	1.000	1.000	1.000	1.000
88	1.000	1.000	1.000	1.000	1.000	1.000
89	1.000	1.000	1.000	1.000	1.000	1.000
90	1.000	1.000	1.000	1.000	1.000	1.000
91	1.000	1.000	1.000	1.000	1.000	1.000
92	1.000	1.000	1.000	1.000	1.000	1.000
93	1.000	1.000	1.000	1.000	1.000	1.000
94	1.000	1.000	1.000	1.000	1.000	1.000
95	1.000	1.000	1.000	1.000	1.000	1.000
96	1.000	1.000	1.000	1.000	1.000	1.000
97	1.000	1.000	1.000	1.000	1.000	1.000
98	1.000	1.000	1.000	1.000	1.000	1.000
99	1.000	1.000	1.000	1.000	1.000	1.000
100	1.000	1.000	1.000	1.000	1.000	1.000
101	1.000	1.000	1.000	1.000	1.000	1.000
102	1.000	1.000	1.000	1.000	1.000	1.000
103	1.000	1.000	1.000	1.000	1.000	1.000
104	1.000	1.000	1.000	1.000	1.000	1.000
105	1.000	1.000	1.000	1.000	1.000	1.000
106	1.000	1.000	1.000	1.000	1.000	1.000
107	1.000	1.000	1.000	1.000	1.000	1.000
108	1.000	1.000	1.000	1.000	1.000	1.000
109	1.000	1.000	1.000	1.000	1.000	1.000
110	1.000	1.000	1.000	1.000	1.000	1.000
111	1.000	1.000	1.000	1.000	1.000	1.000
112	1.000	1.000	1.000	1.000	1.000	1.000
113	1.000	1.000	1.000	1.000	1.000	1.000
114	1.000	1.000	1.000	1.000	1.000	1.000
115	1.000	1.000	1.000	1.000	1.000	1.000
116	1.000	1.000	1.000	1.000	1.000	1.000
117	1.000	1.000	1.000	1.000	1.000	1.000
118	1.000	1.000	1.000	1.000	1.000	1.000
119	1.000	1.000	1.000	1.000	1.000	1.000

120	1.000	1.000	1.000	1.000	1.000	1.000
121	1.000	1.000	1.000	1.000	1.000	1.000
122	1.000	1.000	1.000	1.000	1.000	1.000
123	1.000	1.000	1.000	1.000	1.000	1.000
124	1.000	1.000	1.000	1.000	1.000	1.000
125	1.000	1.000	1.000	1.000	1.000	1.000
126	1.000	1.000	1.000	1.000	1.000	1.000
127	1.000	1.000	1.000	1.000	1.000	1.000
128	1.000	1.000	1.000	1.000	1.000	1.000
129	1.000	1.000	1.000	1.000	1.000	1.000
130	1.000	1.000	1.000	1.000	1.000	1.000
131	1.000	1.000	1.000	1.000	1.000	1.000
132	1.000	1.000	1.000	1.000	1.000	1.000
133	1.000	1.000	1.000	1.000	1.000	1.000
134	1.000	1.000	1.000	1.000	1.000	1.000
135	1.000	1.000	1.000	1.000	1.000	1.000
136	1.000	1.000	1.000	1.000	1.000	1.000
137	1.000	1.000	1.000	1.000	1.000	1.000
138	1.000	1.000	1.000	1.000	1.000	1.000
139	1.000	1.000	1.000	1.000	1.000	1.000
140	1.000	1.000	1.000	1.000	1.000	1.000
141	1.000	1.000	1.000	1.000	1.000	1.000
142	1.000	1.000	1.000	1.000	1.000	1.000
143	1.000	1.000	1.000	1.000	1.000	1.000
144	1.000	1.000	1.000	1.000	1.000	1.000
145	1.000	1.000	1.000	1.000	1.000	1.000
146	1.000	1.000	1.000	1.000	1.000	1.000
147	1.000	1.000	1.000	1.000	1.000	1.000
148	1.000	1.000	1.000	1.000	1.000	1.000
149	1.000	1.000	1.000	1.000	1.000	1.000
150	1.000	1.000	1.000	1.000	1.000	1.000
151	1.000	1.000	1.000	1.000	1.000	1.000
152	1.000	1.000	1.000	1.000	1.000	1.000
153	1.000	1.000	1.000	1.000	1.000	1.000
154	1.000	1.000	1.000	1.000	1.000	1.000
155	1.000	1.000	1.000	1.000	1.000	1.000
156	1.000	1.000	1.000	1.000	1.000	1.000
157	1.000	1.000	1.000	1.000	1.000	1.000
158	1.000	1.000	1.000	1.000	1.000	1.000
159	1.000	1.000	1.000	1.000	1.000	1.000
160	1.000	1.000	1.000	1.000	1.000	1.000
161	1.000	1.000	1.000	1.000	1.000	1.000

162	1.000	1.000	1.000	1.000	1.000	1.000
163	1.000	1.000	1.000	1.000	1.000	1.000
164	1.000	1.000	1.000	1.000	1.000	1.000
165	1.000	1.000	1.000	1.000	1.000	1.000
166	1.000	1.000	1.000	1.000	1.000	1.000
167	1.000	1.000	1.000	1.000	1.000	1.000
168	1.000	1.000	1.000	1.000	1.000	1.000
169	1.000	1.000	1.000	1.000	1.000	1.000
170	1.000	1.000	1.000	1.000	1.000	1.000
171	1.000	1.000	1.000	1.000	1.000	1.000
172	1.000	1.000	1.000	1.000	1.000	1.000
173	1.000	1.000	1.000	1.000	1.000	1.000
174	1.000	1.000	1.000	1.000	1.000	1.000
175	1.000	1.000	1.000	1.000	1.000	1.000
176	1.000	1.000	1.000	1.000	1.000	1.000
177	1.000	1.000	1.000	1.000	1.000	1.000
178	1.000	1.000	1.000	1.000	1.000	1.000
179	1.000	1.000	1.000	1.000	1.000	1.000
180	1.000	1.000	1.000	1.000	1.000	1.000
181	1.000	1.000	1.000	1.000	1.000	1.000
182	1.000	1.000	1.000	1.000	1.000	1.000
183	1.000	1.000	1.000	1.000	1.000	1.000
184	1.000	1.000	1.000	1.000	1.000	1.000
185	1.000	1.000	1.000	1.000	1.000	1.000
186	1.000	1.000	1.000	1.000	1.000	1.000
187	1.000	1.000	1.000	1.000	1.000	1.000
188	1.000	1.000	1.000	1.000	1.000	1.000
189	1.000	1.000	1.000	1.000	1.000	1.000
190	1.000	1.000	1.000	1.000	1.000	1.000
191	1.000	1.000	1.000	1.000	1.000	1.000
192	1.000	1.000	1.000	1.000	1.000	1.000
193	1.000	1.000	1.000	1.000	1.000	1.000
194	1.000	1.000	1.000	1.000	1.000	1.000
195	1.000	1.000	1.000	1.000	1.000	1.000
196	1.000	1.000	1.000	1.000	1.000	1.000
197	1.000	1.000	1.000	1.000	1.000	1.000
198	1.000	1.000	1.000	1.000	1.000	1.000
199	1.000	1.000	1.000	1.000	1.000	1.000
200	1.000	1.000	1.000	1.000	1.000	1.000
201	1.000	1.000	1.000	1.000	1.000	1.000
202	1.000	1.000	1.000	1.000	1.000	1.000
203	1.000	1.000	1.000	1.000	1.000	1.000

204	1.000	1.000	1.000	1.000	1.000	1.000
205	1.000	1.000	1.000	1.000	1.000	1.000
206	1.000	1.000	1.000	1.000	1.000	1.000
207	1.000	1.000	1.000	1.000	1.000	1.000
208	1.000	1.000	1.000	1.000	1.000	1.000
209	1.000	1.000	1.000	1.000	1.000	1.000
210	1.000	1.000	1.000	1.000	1.000	1.000
211	1.000	1.000	1.000	1.000	1.000	1.000
212	1.000	1.000	1.000	1.000	1.000	1.000
213	1.000	1.000	1.000	1.000	1.000	1.000
214	1.000	1.000	1.000	1.000	1.000	1.000
215	1.000	1.000	1.000	1.000	1.000	1.000
216	1.000	1.000	1.000	1.000	1.000	1.000
217	1.000	1.000	1.000	1.000	1.000	1.000
218	1.000	1.000	1.000	1.000	1.000	1.000
219	1.000	1.000	1.000	1.000	1.000	1.000
220	1.000	1.000	1.000	1.000	1.000	1.000
221	1.000	1.000	1.000	1.000	1.000	1.000
222	1.000	1.000	1.000	1.000	1.000	1.000
223	1.000	1.000	1.000	1.000	1.000	1.000
224	1.000	1.000	1.000	1.000	1.000	1.000
225	1.000	1.000	1.000	1.000	1.000	1.000
226	1.000	1.000	1.000	1.000	1.000	1.000
227	1.000	1.000	1.000	1.000	1.000	1.000
228	1.000	1.000	1.000	1.000	1.000	1.000
229	1.000	1.000	1.000	1.000	1.000	1.000
230	1.000	1.000	1.000	1.000	1.000	1.000
231	1.000	1.000	1.000	1.000	1.000	1.000
232	1.000	1.000	1.000	1.000	1.000	1.000
233	1.000	1.000	1.000	1.000	1.000	1.000
234	1.000	1.000	1.000	1.000	1.000	1.000
235	1.000	1.000	1.000	1.000	1.000	1.000
236	1.000	1.000	1.000	1.000	1.000	1.000
237	1.000	1.000	1.000	1.000	1.000	1.000
238	1.000	1.000	1.000	1.000	1.000	1.000
239	1.000	1.000	1.000	1.000	1.000	1.000
240	1.000	1.000	1.000	1.000	1.000	1.000
241	1.000	1.000	1.000	1.000	1.000	1.000
242	1.000	1.000	1.000	1.000	1.000	1.000
243	1.000	1.000	1.000	1.000	1.000	1.000
244	1.000	1.000	1.000	1.000	1.000	1.000
245	1.000	1.000	1.000	1.000	1.000	1.000

246	1.000	1.000	1.000	1.000	1.000	1.000
247	1.000	1.000	1.000	1.000	1.000	1.000
248	1.000	1.000	1.000	1.000	1.000	1.000
249	1.000	1.000	1.000	1.000	1.000	1.000
250	1.000	1.000	1.000	1.000	1.000	1.000
251	1.000	1.000	1.000	1.000	1.000	1.000
252	1.000	1.000	1.000	1.000	1.000	1.000
253	1.000	1.000	1.000	1.000	1.000	1.000
254	1.000	1.000	1.000	1.000	1.000	1.000
255	1.000	1.000	1.000	1.000	1.000	1.000
256	1.000	1.000	1.000	1.000	1.000	1.000
257	1.000	1.000	1.000	1.000	1.000	1.000
258	1.000	1.000	1.000	1.000	1.000	1.000
259	1.000	1.000	1.000	1.000	1.000	1.000
260	1.000	1.000	1.000	1.000	1.000	1.000
261	1.000	1.000	1.000	1.000	1.000	1.000
262	1.000	1.000	1.000	1.000	1.000	1.000
263	1.000	1.000	1.000	1.000	1.000	1.000
264	1.000	1.000	1.000	1.000	1.000	1.000
265	1.000	1.000	1.000	1.000	1.000	1.000
266	1.000	1.000	1.000	1.000	1.000	1.000
267	1.000	1.000	1.000	1.000	1.000	1.000
268	1.000	1.000	1.000	1.000	1.000	1.000
269	1.000	1.000	1.000	1.000	1.000	1.000
270	1.000	1.000	1.000	1.000	1.000	1.000
271	1.000	1.000	1.000	1.000	1.000	1.000
272	1.000	1.000	1.000	1.000	1.000	1.000
273	1.000	1.000	1.000	1.000	1.000	1.000
274	1.000	1.000	1.000	1.000	1.000	1.000
275	1.000	1.000	1.000	1.000	1.000	1.000
276	1.000	1.000	1.000	1.000	1.000	1.000
277	1.000	1.000	1.000	1.000	1.000	1.000
278	1.000	1.000	1.000	1.000	1.000	1.000
279	1.000	1.000	1.000	1.000	1.000	1.000
280	1.000	1.000	1.000	1.000	1.000	1.000
281	1.000	1.000	1.000	1.000	1.000	1.000
282	1.000	1.000	1.000	1.000	1.000	1.000
283	1.000	1.000	1.000	1.000	1.000	1.000
284	1.000	1.000	1.000	1.000	1.000	1.000
285	1.000	1.000	1.000	1.000	1.000	1.000
286	1.000	1.000	1.000	1.000	1.000	1.000
287	1.000	1.000	1.000	1.000	1.000	1.000

288	1.000	1.000	1.000	1.000	1.000	1.000
289	1.000	1.000	1.000	1.000	1.000	1.000
290	1.000	1.000	1.000	1.000	1.000	1.000
291	1.000	1.000	1.000	1.000	1.000	1.000
292	1.000	1.000	1.000	1.000	1.000	1.000
293	1.000	1.000	1.000	1.000	1.000	1.000
294	1.000	1.000	1.000	1.000	1.000	1.000
295	1.000	1.000	1.000	1.000	1.000	1.000
296	1.000	1.000	1.000	1.000	1.000	1.000
297	1.000	1.000	1.000	1.000	1.000	1.000
298	1.000	1.000	1.000	1.000	1.000	1.000
299	1.000	1.000	1.000	1.000	1.000	1.000
300	1.000	1.000	1.000	1.000	1.000	1.000
301	1.000	1.000	1.000	1.000	1.000	1.000
302	1.000	1.000	1.000	1.000	1.000	1.000
303	1.000	1.000	1.000	1.000	1.000	1.000
304	1.000	1.000	1.000	1.000	1.000	1.000
305	1.000	1.000	1.000	1.000	1.000	1.000
306	1.000	1.000	1.000	1.000	1.000	1.000
307	1.000	1.000	1.000	1.000	1.000	1.000
308	1.000	1.000	1.000	1.000	1.000	1.000
309	1.000	1.000	1.000	1.000	1.000	1.000
310	1.000	1.000	1.000	1.000	1.000	1.000
311	1.000	1.000	1.000	1.000	1.000	1.000
312	1.000	1.000	1.000	1.000	1.000	1.000
313	1.000	1.000	1.000	1.000	1.000	1.000
314	1.000	1.000	1.000	1.000	1.000	1.000
315	1.000	1.000	1.000	1.000	1.000	1.000
316	1.000	1.000	1.000	1.000	1.000	1.000
317	1.000	1.000	1.000	1.000	1.000	1.000
318	1.000	1.000	1.000	1.000	1.000	1.000
319	1.000	1.000	1.000	1.000	1.000	1.000
320	1.000	1.000	1.000	1.000	1.000	1.000
321	1.000	1.000	1.000	1.000	1.000	1.000
322	1.000	1.000	1.000	1.000	1.000	1.000
323	1.000	1.000	1.000	1.000	1.000	1.000
324	1.000	1.000	1.000	1.000	1.000	1.000
325	1.000	1.000	1.000	1.000	1.000	1.000
326	1.000	1.000	1.000	1.000	1.000	1.000
327	1.000	1.000	1.000	1.000	1.000	1.000
328	1.000	1.000	1.000	1.000	1.000	1.000
329	1.000	1.000	1.000	1.000	1.000	1.000

330	1.000	1.000	1.000	1.000	1.000	1.000
331	1.000	1.000	1.000	1.000	1.000	1.000
332	1.000	1.000	1.000	1.000	1.000	1.000
333	1.000	1.000	1.000	1.000	1.000	1.000
334	1.000	1.000	1.000	1.000	1.000	1.000
335	1.000	1.000	1.000	1.000	1.000	1.000
336	1.000	1.000	1.000	1.000	1.000	1.000
337	1.000	1.000	1.000	1.000	1.000	1.000
338	1.000	1.000	1.000	1.000	1.000	1.000
339	1.000	1.000	1.000	1.000	1.000	1.000
340	1.000	1.000	1.000	1.000	1.000	1.000
341	1.000	1.000	1.000	1.000	1.000	1.000
342	1.000	1.000	1.000	1.000	1.000	1.000
343	1.000	1.000	1.000	1.000	1.000	1.000
344	1.000	1.000	1.000	1.000	1.000	1.000
345	1.000	1.000	1.000	1.000	1.000	1.000
346	1.000	1.000	1.000	1.000	1.000	1.000
347	1.000	1.000	1.000	1.000	1.000	1.000
348	1.000	1.000	1.000	1.000	1.000	1.000
349	1.000	1.000	1.000	1.000	1.000	1.000
350	1.000	1.000	1.000	1.000	1.000	1.000
351	1.000	1.000	1.000	1.000	1.000	1.000
352	1.000	1.000	1.000	1.000	1.000	1.000
353	1.000	1.000	1.000	1.000	1.000	1.000
354	1.000	1.000	1.000	1.000	1.000	1.000
355	1.000	1.000	1.000	1.000	1.000	1.000
356	1.000	1.000	1.000	1.000	1.000	1.000
357	1.000	1.000	1.000	1.000	1.000	1.000
358	1.000	1.000	1.000	1.000	1.000	1.000
359	1.000	1.000	1.000	1.000	1.000	1.000
360	1.000	1.000	1.000	1.000	1.000	1.000
361	1.000	1.000	1.000	1.000	1.000	1.000
362	1.000	1.000	1.000	1.000	1.000	1.000
363	1.000	1.000	1.000	1.000	1.000	1.000
364	1.000	1.000	1.000	1.000	1.000	1.000
365	1.000	1.000	1.000	1.000	1.000	1.000
366	1.000	1.000	1.000	1.000	1.000	1.000
367	1.000	1.000	1.000	1.000	1.000	1.000
368	1.000	1.000	1.000	1.000	1.000	1.000
369	1.000	1.000	1.000	1.000	1.000	1.000
370	1.000	1.000	1.000	1.000	1.000	1.000
371	1.000	1.000	1.000	1.000	1.000	1.000

372	1.000	1.000	1.000	1.000	1.000	1.000
373	1.000	1.000	1.000	1.000	1.000	1.000
374	1.000	1.000	1.000	1.000	1.000	1.000
375	1.000	1.000	1.000	1.000	1.000	1.000
376	1.000	1.000	1.000	1.000	1.000	1.000
377	1.000	1.000	1.000	1.000	1.000	1.000
378	1.000	1.000	1.000	1.000	1.000	1.000
379	1.000	1.000	1.000	1.000	1.000	1.000
380	1.000	1.000	1.000	1.000	1.000	1.000
381	1.000	1.000	1.000	1.000	1.000	1.000
382	1.000	1.000	1.000	1.000	1.000	1.000
383	1.000	1.000	1.000	1.000	1.000	1.000
384	1.000	1.000	1.000	1.000	1.000	1.000
385	1.000	1.000	1.000	1.000	1.000	1.000
386	1.000	1.000	1.000	1.000	1.000	1.000
387	1.000	1.000	1.000	1.000	1.000	1.000
388	1.000	1.000	1.000	1.000	1.000	1.000
389	1.000	1.000	1.000	1.000	1.000	1.000
390	1.000	1.000	1.000	1.000	1.000	1.000
391	1.000	1.000	1.000	1.000	1.000	1.000
392	1.000	1.000	1.000	1.000	1.000	1.000
393	1.000	1.000	1.000	1.000	1.000	1.000
394	1.000	1.000	1.000	1.000	1.000	1.000
395	1.000	1.000	1.000	1.000	1.000	1.000
396	1.000	1.000	1.000	1.000	1.000	1.000
397	1.000	1.000	1.000	1.000	1.000	1.000
398	1.000	1.000	1.000	1.000	1.000	1.000
399	1.000	1.000	1.000	1.000	1.000	1.000
400	1.000	1.000	1.000	1.000	1.000	1.000
401	1.000	1.000	1.000	1.000	1.000	1.000
402	1.000	1.000	1.000	1.000	1.000	1.000
403	1.000	1.000	1.000	1.000	1.000	1.000
404	1.000	1.000	1.000	1.000	1.000	1.000
405	1.000	1.000	1.000	1.000	1.000	1.000
406	1.000	1.000	1.000	1.000	1.000	1.000
407	1.000	1.000	1.000	1.000	1.000	1.000
408	1.000	1.000	1.000	1.000	1.000	1.000
409	1.000	1.000	1.000	1.000	1.000	1.000
410	1.000	1.000	1.000	1.000	1.000	1.000
411	1.000	1.000	1.000	1.000	1.000	1.000
412	1.000	1.000	1.000	1.000	1.000	1.000
413	1.000	1.000	1.000	1.000	1.000	1.000

414	1.000	1.000	1.000	1.000	1.000	1.000
415	1.000	1.000	1.000	1.000	1.000	1.000
416	1.000	1.000	1.000	1.000	1.000	1.000
417	1.000	1.000	1.000	1.000	1.000	1.000
418	1.000	1.000	1.000	1.000	1.000	1.000
419	1.000	1.000	1.000	1.000	1.000	1.000
420	1.000	1.000	1.000	1.000	1.000	1.000
421	1.000	1.000	1.000	1.000	1.000	1.000
422	1.000	1.000	1.000	1.000	1.000	1.000
423	1.000	1.000	1.000	1.000	1.000	1.000
424	1.000	1.000	1.000	1.000	1.000	1.000
425	1.000	1.000	1.000	1.000	1.000	1.000
426	1.000	1.000	1.000	1.000	1.000	1.000
427	1.000	1.000	1.000	1.000	1.000	1.000
428	1.000	1.000	1.000	1.000	1.000	1.000
429	1.000	1.000	1.000	1.000	1.000	1.000
430	1.000	1.000	1.000	1.000	1.000	1.000
431	1.000	1.000	1.000	1.000	1.000	1.000
432	1.000	1.000	1.000	1.000	1.000	1.000
433	1.000	1.000	1.000	1.000	1.000	1.000
434	1.000	1.000	1.000	1.000	1.000	1.000
435	1.000	1.000	1.000	1.000	1.000	1.000
436	1.000	1.000	1.000	1.000	1.000	1.000
437	1.000	1.000	1.000	1.000	1.000	1.000
438	1.000	1.000	1.000	1.000	1.000	1.000
439	1.000	1.000	1.000	1.000	1.000	1.000
440	1.000	1.000	1.000	1.000	1.000	1.000
441	1.000	1.000	1.000	1.000	1.000	1.000
442	1.000	1.000	1.000	1.000	1.000	1.000
443	1.000	1.000	1.000	1.000	1.000	1.000
444	1.000	1.000	1.000	1.000	1.000	1.000
445	1.000	1.000	1.000	1.000	1.000	1.000
446	1.000	1.000	1.000	1.000	1.000	1.000
447	1.000	1.000	1.000	1.000	1.000	1.000
448	1.000	1.000	1.000	1.000	1.000	1.000
449	1.000	1.000	1.000	1.000	1.000	1.000
450	1.000	1.000	1.000	1.000	1.000	1.000
451	1.000	1.000	1.000	1.000	1.000	1.000
452	1.000	1.000	1.000	1.000	1.000	1.000
453	1.000	1.000	1.000	1.000	1.000	1.000
454	1.000	1.000	1.000	1.000	1.000	1.000
455	1.000	1.000	1.000	1.000	1.000	1.000

456	1.000	1.000	1.000	1.000	1.000	1.000
457	1.000	1.000	1.000	1.000	1.000	1.000
458	1.000	1.000	1.000	1.000	1.000	1.000
459	1.000	1.000	1.000	1.000	1.000	1.000
460	1.000	1.000	1.000	1.000	1.000	1.000
461	1.000	1.000	1.000	1.000	1.000	1.000
462	1.000	1.000	1.000	1.000	1.000	1.000
463	1.000	1.000	1.000	1.000	1.000	1.000
464	1.000	1.000	1.000	1.000	1.000	1.000
465	1.000	1.000	1.000	1.000	1.000	1.000
466	1.000	1.000	1.000	1.000	1.000	1.000
467	1.000	1.000	1.000	1.000	1.000	1.000
468	1.000	1.000	1.000	1.000	1.000	1.000
469	1.000	1.000	1.000	1.000	1.000	1.000
470	1.000	1.000	1.000	1.000	1.000	1.000
471	1.000	1.000	1.000	1.000	1.000	1.000
472	1.000	1.000	1.000	1.000	1.000	1.000
473	1.000	1.000	1.000	1.000	1.000	1.000
474	1.000	1.000	1.000	1.000	1.000	1.000
475	1.000	1.000	1.000	1.000	1.000	1.000
476	1.000	1.000	1.000	1.000	1.000	1.000
477	1.000	1.000	1.000	1.000	1.000	1.000
478	1.000	1.000	1.000	1.000	1.000	1.000
479	1.000	1.000	1.000	1.000	1.000	1.000
480	1.000	1.000	1.000	1.000	1.000	1.000
481	1.000	1.000	1.000	1.000	1.000	1.000
482	1.000	1.000	1.000	1.000	1.000	1.000
483	1.000	1.000	1.000	1.000	1.000	1.000
484	1.000	1.000	1.000	1.000	1.000	1.000
485	1.000	1.000	1.000	1.000	1.000	1.000
486	1.000	1.000	1.000	1.000	1.000	1.000
487	1.000	1.000	1.000	1.000	1.000	1.000
488	1.000	1.000	1.000	1.000	1.000	1.000
489	1.000	1.000	1.000	1.000	1.000	1.000
490	1.000	1.000	1.000	1.000	1.000	1.000
491	1.000	1.000	1.000	1.000	1.000	1.000
492	1.000	1.000	1.000	1.000	1.000	1.000
493	1.000	1.000	1.000	1.000	1.000	1.000
494	1.000	1.000	1.000	1.000	1.000	1.000
495	1.000	1.000	1.000	1.000	1.000	1.000
496	1.000	1.000	1.000	1.000	1.000	1.000
497	1.000	1.000	1.000	1.000	1.000	1.000

498	1.000	1.000	1.000	1.000	1.000	1.000
499	1.000	1.000	1.000	1.000	1.000	1.000
500	1.000	1.000	1.000	1.000	1.000	1.000
501	1.000	1.000	1.000	1.000	1.000	1.000
502	1.000	1.000	1.000	1.000	1.000	1.000
503	1.000	1.000	1.000	1.000	1.000	1.000
504	1.000	1.000	1.000	1.000	1.000	1.000
505	1.000	1.000	1.000	1.000	1.000	1.000
506	1.000	1.000	1.000	1.000	1.000	1.000
507	1.000	1.000	1.000	1.000	1.000	1.000
508	1.000	1.000	1.000	1.000	1.000	1.000
509	1.000	1.000	1.000	1.000	1.000	1.000
510	1.000	1.000	1.000	1.000	1.000	1.000
511	1.000	1.000	1.000	1.000	1.000	1.000
512	1.000	1.000	1.000	1.000	1.000	1.000
513	1.000	1.000	1.000	1.000	1.000	1.000
514	1.000	1.000	1.000	1.000	1.000	1.000
515	1.000	1.000	1.000	1.000	1.000	1.000
516	1.000	1.000	1.000	1.000	1.000	1.000
517	1.000	1.000	1.000	1.000	1.000	1.000
518	1.000	1.000	1.000	1.000	1.000	1.000
519	1.000	1.000	1.000	1.000	1.000	1.000
520	1.000	1.000	1.000	1.000	1.000	1.000
521	1.000	1.000	1.000	1.000	1.000	1.000
522	1.000	1.000	1.000	1.000	1.000	1.000
523	1.000	1.000	1.000	1.000	1.000	1.000
524	1.000	1.000	1.000	1.000	1.000	1.000
525	1.000	1.000	1.000	1.000	1.000	1.000
526	1.000	1.000	1.000	1.000	1.000	1.000
527	1.000	1.000	1.000	1.000	1.000	1.000
528	1.000	1.000	1.000	1.000	1.000	1.000
529	1.000	1.000	1.000	1.000	1.000	1.000
530	1.000	1.000	1.000	1.000	1.000	1.000
531	1.000	1.000	1.000	1.000	1.000	1.000
532	1.000	1.000	1.000	1.000	1.000	1.000
533	1.000	1.000	1.000	1.000	1.000	1.000
534	1.000	1.000	1.000	1.000	1.000	1.000
535	1.000	1.000	1.000	1.000	1.000	1.000
536	1.000	1.000	1.000	1.000	1.000	1.000
537	1.000	1.000	1.000	1.000	1.000	1.000
538	1.000	1.000	1.000	1.000	1.000	1.000
539	1.000	1.000	1.000	1.000	1.000	1.000

540	1.000	1.000	1.000	1.000	1.000	1.000
541	1.000	1.000	1.000	1.000	1.000	1.000
542	1.000	1.000	1.000	1.000	1.000	1.000
543	1.000	1.000	1.000	1.000	1.000	1.000
544	1.000	1.000	1.000	1.000	1.000	1.000
545	1.000	1.000	1.000	1.000	1.000	1.000
546	1.000	1.000	1.000	1.000	1.000	1.000
547	1.000	1.000	1.000	1.000	1.000	1.000
548	1.000	1.000	1.000	1.000	1.000	1.000
549	1.000	1.000	1.000	1.000	1.000	1.000
550	1.000	1.000	1.000	1.000	1.000	1.000
551	1.000	1.000	1.000	1.000	1.000	1.000
552	1.000	1.000	1.000	1.000	1.000	1.000
553	1.000	1.000	1.000	1.000	1.000	1.000
554	1.000	1.000	1.000	1.000	1.000	1.000
555	1.000	1.000	1.000	1.000	1.000	1.000
556	1.000	1.000	1.000	1.000	1.000	1.000
557	1.000	1.000	1.000	1.000	1.000	1.000
558	1.000	1.000	1.000	1.000	1.000	1.000
559	1.000	1.000	1.000	1.000	1.000	1.000
560	1.000	1.000	1.000	1.000	1.000	1.000
561	1.000	1.000	1.000	1.000	1.000	1.000
562	1.000	1.000	1.000	1.000	1.000	1.000
563	1.000	1.000	1.000	1.000	1.000	1.000
564	1.000	1.000	1.000	1.000	1.000	1.000
565	1.000	1.000	1.000	1.000	1.000	1.000
566	1.000	1.000	1.000	1.000	1.000	1.000
567	1.000	1.000	1.000	1.000	1.000	1.000
568	1.000	1.000	1.000	1.000	1.000	1.000
569	1.000	1.000	1.000	1.000	1.000	1.000
570	1.000	1.000	1.000	1.000	1.000	1.000
571	1.000	1.000	1.000	1.000	1.000	1.000
572	1.000	1.000	1.000	1.000	1.000	1.000
573	1.000	1.000	1.000	1.000	1.000	1.000
574	1.000	1.000	1.000	1.000	1.000	1.000
575	1.000	1.000	1.000	1.000	1.000	1.000
576	1.000	1.000	1.000	1.000	1.000	1.000
577	1.000	1.000	1.000	1.000	1.000	1.000
578	1.000	1.000	1.000	1.000	1.000	1.000
579	1.000	1.000	1.000	1.000	1.000	1.000
580	1.000	1.000	1.000	1.000	1.000	1.000
581	1.000	1.000	1.000	1.000	1.000	1.000

582	1.000	1.000	1.000	1.000	1.000	1.000
583	1.000	1.000	1.000	1.000	1.000	1.000
584	1.000	1.000	1.000	1.000	1.000	1.000
585	1.000	1.000	1.000	1.000	1.000	1.000
586	1.000	1.000	1.000	1.000	1.000	1.000
587	1.000	1.000	1.000	1.000	1.000	1.000
588	1.000	1.000	1.000	1.000	1.000	1.000
589	1.000	1.000	1.000	1.000	1.000	1.000
590	1.000	1.000	1.000	1.000	1.000	1.000
591	1.000	1.000	1.000	1.000	1.000	1.000
592	1.000	1.000	1.000	1.000	1.000	1.000
593	1.000	1.000	1.000	1.000	1.000	1.000
594	1.000	1.000	1.000	1.000	1.000	1.000
595	1.000	1.000	1.000	1.000	1.000	1.000
596	1.000	1.000	1.000	1.000	1.000	1.000
597	1.000	1.000	1.000	1.000	1.000	1.000
598	1.000	1.000	1.000	1.000	1.000	1.000
599	1.000	1.000	1.000	1.000	1.000	1.000
600	1.000	1.000	1.000	1.000	1.000	1.000
601	1.000	1.000	1.000	1.000	1.000	1.000
602	1.000	1.000	1.000	1.000	1.000	1.000
603	1.000	1.000	1.000	1.000	1.000	1.000
604	1.000	1.000	1.000	1.000	1.000	1.000
605	1.000	1.000	1.000	1.000	1.000	1.000
606	1.000	1.000	1.000	1.000	1.000	1.000
607	1.000	1.000	1.000	1.000	1.000	1.000
608	1.000	1.000	1.000	1.000	1.000	1.000
609	1.000	1.000	1.000	1.000	1.000	1.000
610	1.000	1.000	1.000	1.000	1.000	1.000
611	1.000	1.000	1.000	1.000	1.000	1.000
612	1.000	1.000	1.000	1.000	1.000	1.000
613	1.000	1.000	1.000	1.000	1.000	1.000
614	1.000	1.000	1.000	1.000	1.000	1.000
615	1.000	1.000	1.000	1.000	1.000	1.000
616	1.000	1.000	1.000	1.000	1.000	1.000
617	1.000	1.000	1.000	1.000	1.000	1.000
618	1.000	1.000	1.000	1.000	1.000	1.000
619	1.000	1.000	1.000	1.000	1.000	1.000
620	1.000	1.000	1.000	1.000	1.000	1.000
621	1.000	1.000	1.000	1.000	1.000	1.000
622	1.000	1.000	1.000	1.000	1.000	1.000
623	1.000	1.000	1.000	1.000	1.000	1.000

624	1.000	1.000	1.000	1.000	1.000	1.000
625	1.000	1.000	1.000	1.000	1.000	1.000
626	1.000	1.000	1.000	1.000	1.000	1.000
627	1.000	1.000	1.000	1.000	1.000	1.000
628	1.000	1.000	1.000	1.000	1.000	1.000
629	1.000	1.000	1.000	1.000	1.000	1.000
630	1.000	1.000	1.000	1.000	1.000	1.000
631	1.000	1.000	1.000	1.000	1.000	1.000
632	1.000	1.000	1.000	1.000	1.000	1.000
633	1.000	1.000	1.000	1.000	1.000	1.000
634	1.000	1.000	1.000	1.000	1.000	1.000
635	1.000	1.000	1.000	1.000	1.000	1.000
636	1.000	1.000	1.000	1.000	1.000	1.000
637	1.000	1.000	1.000	1.000	1.000	1.000
638	1.000	1.000	1.000	1.000	1.000	1.000
639	1.000	1.000	1.000	1.000	1.000	1.000
640	1.000	1.000	1.000	1.000	1.000	1.000
641	1.000	1.000	1.000	1.000	1.000	1.000
642	1.000	1.000	1.000	1.000	1.000	1.000
643	1.000	1.000	1.000	1.000	1.000	1.000
644	1.000	1.000	1.000	1.000	1.000	1.000
645	1.000	1.000	1.000	1.000	1.000	1.000
646	1.000	1.000	1.000	1.000	1.000	1.000
647	1.000	1.000	1.000	1.000	1.000	1.000
648	1.000	1.000	1.000	1.000	1.000	1.000
649	1.000	1.000	1.000	1.000	1.000	1.000
650	1.000	1.000	1.000	1.000	1.000	1.000
651	1.000	1.000	1.000	1.000	1.000	1.000
652	1.000	1.000	1.000	1.000	1.000	1.000
653	1.000	1.000	1.000	1.000	1.000	1.000
654	1.000	1.000	1.000	1.000	1.000	1.000
655	1.000	1.000	1.000	1.000	1.000	1.000
656	1.000	1.000	1.000	1.000	1.000	1.000
657	1.000	1.000	1.000	1.000	1.000	1.000
658	1.000	1.000	1.000	1.000	1.000	1.000
659	1.000	1.000	1.000	1.000	1.000	1.000
660	1.000	1.000	1.000	1.000	1.000	1.000
661	1.000	1.000	1.000	1.000	1.000	1.000
662	1.000	1.000	1.000	1.000	1.000	1.000
663	1.000	1.000	1.000	1.000	1.000	1.000
664	1.000	1.000	1.000	1.000	1.000	1.000
665	1.000	1.000	1.000	1.000	1.000	1.000

666	1.000	1.000	1.000	1.000	1.000	1.000
667	1.000	1.000	1.000	1.000	1.000	1.000
668	1.000	1.000	1.000	1.000	1.000	1.000
669	1.000	1.000	1.000	1.000	1.000	1.000
670	1.000	1.000	1.000	1.000	1.000	1.000
671	1.000	1.000	1.000	1.000	1.000	1.000
672	1.000	1.000	1.000	1.000	1.000	1.000
673	1.000	1.000	1.000	1.000	1.000	1.000
674	1.000	1.000	1.000	1.000	1.000	1.000
675	1.000	1.000	1.000	1.000	1.000	1.000
676	1.000	1.000	1.000	1.000	1.000	1.000
677	1.000	1.000	1.000	1.000	1.000	1.000
678	1.000	1.000	1.000	1.000	1.000	1.000
679	1.000	1.000	1.000	1.000	1.000	1.000
680	1.000	1.000	1.000	1.000	1.000	1.000
681	1.000	1.000	1.000	1.000	1.000	1.000
682	1.000	1.000	1.000	1.000	1.000	1.000
683	1.000	1.000	1.000	1.000	1.000	1.000
684	1.000	1.000	1.000	1.000	1.000	1.000
685	1.000	1.000	1.000	1.000	1.000	1.000
686	1.000	1.000	1.000	1.000	1.000	1.000
687	1.000	1.000	1.000	1.000	1.000	1.000
688	1.000	1.000	1.000	1.000	1.000	1.000
689	1.000	1.000	1.000	1.000	1.000	1.000
690	1.000	1.000	1.000	1.000	1.000	1.000
691	1.000	1.000	1.000	1.000	1.000	1.000
692	1.000	1.000	1.000	1.000	1.000	1.000
693	1.000	1.000	1.000	1.000	1.000	1.000
694	1.000	1.000	1.000	1.000	1.000	1.000
695	1.000	1.000	1.000	1.000	1.000	1.000
696	1.000	1.000	1.000	1.000	1.000	1.000
697	1.000	1.000	1.000	1.000	1.000	1.000
698	1.000	1.000	1.000	1.000	1.000	1.000
699	1.000	1.000	1.000	1.000	1.000	1.000
700	1.000	1.000	1.000	1.000	1.000	1.000
701	1.000	1.000	1.000	1.000	1.000	1.000
702	1.000	1.000	1.000	1.000	1.000	1.000
703	1.000	1.000	1.000	1.000	1.000	1.000
704	1.000	1.000	1.000	1.000	1.000	1.000
705	1.000	1.000	1.000	1.000	1.000	1.000
706	1.000	1.000	1.000	1.000	1.000	1.000
707	1.000	1.000	1.000	1.000	1.000	1.000

708	1.000	1.000	1.000	1.000	1.000	1.000
709	1.000	1.000	1.000	1.000	1.000	1.000
710	1.000	1.000	1.000	1.000	1.000	1.000
711	1.000	1.000	1.000	1.000	1.000	1.000
712	1.000	1.000	1.000	1.000	1.000	1.000
713	1.000	1.000	1.000	1.000	1.000	1.000
714	1.000	1.000	1.000	1.000	1.000	1.000
715	1.000	1.000	1.000	1.000	1.000	1.000
716	1.000	1.000	1.000	1.000	1.000	1.000
717	1.000	1.000	1.000	1.000	1.000	1.000
718	1.000	1.000	1.000	1.000	1.000	1.000
719	1.000	1.000	1.000	1.000	1.000	1.000
720	1.000	1.000	1.000	1.000	1.000	1.000
721	1.000	1.000	1.000	1.000	1.000	1.000
722	1.000	1.000	1.000	1.000	1.000	1.000
723	1.000	1.000	1.000	1.000	1.000	1.000
724	1.000	1.000	1.000	1.000	1.000	1.000
725	1.000	1.000	1.000	1.000	1.000	1.000
726	1.000	1.000	1.000	1.000	1.000	1.000
727	1.000	1.000	1.000	1.000	1.000	1.000
728	1.000	1.000	1.000	1.000	1.000	1.000
729	1.000	1.000	1.000	1.000	1.000	1.000
730	1.000	1.000	1.000	1.000	1.000	1.000
731	1.000	1.000	1.000	1.000	1.000	1.000
732	1.000	1.000	1.000	1.000	1.000	1.000
733	1.000	1.000	1.000	1.000	1.000	1.000
734	1.000	1.000	1.000	1.000	1.000	1.000
735	1.000	1.000	1.000	1.000	1.000	1.000
736	1.000	1.000	1.000	1.000	1.000	1.000
737	1.000	1.000	1.000	1.000	1.000	1.000
738	1.000	1.000	1.000	1.000	1.000	1.000
739	1.000	1.000	1.000	1.000	1.000	1.000
740	1.000	1.000	1.000	1.000	1.000	1.000
741	1.000	1.000	1.000	1.000	1.000	1.000
742	1.000	1.000	1.000	1.000	1.000	1.000
743	1.000	1.000	1.000	1.000	1.000	1.000
744	1.000	1.000	1.000	1.000	1.000	1.000
745	1.000	1.000	1.000	1.000	1.000	1.000
746	1.000	1.000	1.000	1.000	1.000	1.000
747	1.000	1.000	1.000	1.000	1.000	1.000
748	1.000	1.000	1.000	1.000	1.000	1.000
749	1.000	1.000	1.000	1.000	1.000	1.000

750	1.000	1.000	1.000	1.000	1.000	1.000
751	1.000	1.000	1.000	1.000	1.000	1.000
752	1.000	1.000	1.000	1.000	1.000	1.000
753	1.000	1.000	1.000	1.000	1.000	1.000
754	1.000	1.000	1.000	1.000	1.000	1.000
755	1.000	1.000	1.000	1.000	1.000	1.000
756	1.000	1.000	1.000	1.000	1.000	1.000
757	1.000	1.000	1.000	1.000	1.000	1.000
758	1.000	1.000	1.000	1.000	1.000	1.000
759	1.000	1.000	1.000	1.000	1.000	1.000
760	1.000	1.000	1.000	1.000	1.000	1.000
761	1.000	1.000	1.000	1.000	1.000	1.000
762	1.000	1.000	1.000	1.000	1.000	1.000
763	1.000	1.000	1.000	1.000	1.000	1.000
764	1.000	1.000	1.000	1.000	1.000	1.000
765	1.000	1.000	1.000	1.000	1.000	1.000
766	1.000	1.000	1.000	1.000	1.000	1.000
767	1.000	1.000	1.000	1.000	1.000	1.000
768	1.000	1.000	1.000	1.000	1.000	1.000
769	1.000	1.000	1.000	1.000	1.000	1.000
770	1.000	1.000	1.000	1.000	1.000	1.000
771	1.000	1.000	1.000	1.000	1.000	1.000
772	1.000	1.000	1.000	1.000	1.000	1.000
773	1.000	1.000	1.000	1.000	1.000	1.000
774	1.000	1.000	1.000	1.000	1.000	1.000
775	1.000	1.000	1.000	1.000	1.000	1.000
776	1.000	1.000	1.000	1.000	1.000	1.000
777	1.000	1.000	1.000	1.000	1.000	1.000
778	1.000	1.000	1.000	1.000	1.000	1.000
779	1.000	1.000	1.000	1.000	1.000	1.000
780	1.000	1.000	1.000	1.000	1.000	1.000
781	1.000	1.000	1.000	1.000	1.000	1.000
782	1.000	1.000	1.000	1.000	1.000	1.000
783	1.000	1.000	1.000	1.000	1.000	1.000
784	1.000	1.000	1.000	1.000	1.000	1.000
785	1.000	1.000	1.000	1.000	1.000	1.000
786	1.000	1.000	1.000	1.000	1.000	1.000
787	1.000	1.000	1.000	1.000	1.000	1.000
788	1.000	1.000	1.000	1.000	1.000	1.000
789	1.000	1.000	1.000	1.000	1.000	1.000
790	1.000	1.000	1.000	1.000	1.000	1.000
791	1.000	1.000	1.000	1.000	1.000	1.000

792	1.000	1.000	1.000	1.000	1.000	1.000
793	1.000	1.000	1.000	1.000	1.000	1.000
794	1.000	1.000	1.000	1.000	1.000	1.000
795	1.000	1.000	1.000	1.000	1.000	1.000
796	1.000	1.000	1.000	1.000	1.000	1.000
797	1.000	1.000	1.000	1.000	1.000	1.000
798	1.000	1.000	1.000	1.000	1.000	1.000
799	1.000	1.000	1.000	1.000	1.000	1.000
800	1.000	1.000	1.000	1.000	1.000	1.000
801	1.000	1.000	1.000	1.000	1.000	1.000
802	1.000	1.000	1.000	1.000	1.000	1.000
803	1.000	1.000	1.000	1.000	1.000	1.000
804	1.000	1.000	1.000	1.000	1.000	1.000
805	1.000	1.000	1.000	1.000	1.000	1.000
806	1.000	1.000	1.000	1.000	1.000	1.000
807	1.000	1.000	1.000	1.000	1.000	1.000
808	1.000	1.000	1.000	1.000	1.000	1.000
809	1.000	1.000	1.000	1.000	1.000	1.000
810	1.000	1.000	1.000	1.000	1.000	1.000
811	1.000	1.000	1.000	1.000	1.000	1.000
812	1.000	1.000	1.000	1.000	1.000	1.000
813	1.000	1.000	1.000	1.000	1.000	1.000
814	1.000	1.000	1.000	1.000	1.000	1.000
815	1.000	1.000	1.000	1.000	1.000	1.000
816	1.000	1.000	1.000	1.000	1.000	1.000
817	1.000	1.000	1.000	1.000	1.000	1.000
818	1.000	1.000	1.000	1.000	1.000	1.000
819	1.000	1.000	1.000	1.000	1.000	1.000
820	1.000	1.000	1.000	1.000	1.000	1.000
821	1.000	1.000	1.000	1.000	1.000	1.000
822	1.000	1.000	1.000	1.000	1.000	1.000
823	1.000	1.000	1.000	1.000	1.000	1.000
824	1.000	1.000	1.000	1.000	1.000	1.000
825	1.000	1.000	1.000	1.000	1.000	1.000
826	1.000	1.000	1.000	1.000	1.000	1.000
827	1.000	1.000	1.000	1.000	1.000	1.000
828	1.000	1.000	1.000	1.000	1.000	1.000
829	1.000	1.000	1.000	1.000	1.000	1.000
830	1.000	1.000	1.000	1.000	1.000	1.000
831	1.000	1.000	1.000	1.000	1.000	1.000
832	1.000	1.000	1.000	1.000	1.000	1.000
833	1.000	1.000	1.000	1.000	1.000	1.000

834	1.000	1.000	1.000	1.000	1.000	1.000
835	1.000	1.000	1.000	1.000	1.000	1.000
836	1.000	1.000	1.000	1.000	1.000	1.000
837	1.000	1.000	1.000	1.000	1.000	1.000
838	1.000	1.000	1.000	1.000	1.000	1.000
839	1.000	1.000	1.000	1.000	1.000	1.000
840	1.000	1.000	1.000	1.000	1.000	1.000
841	1.000	1.000	1.000	1.000	1.000	1.000
842	1.000	1.000	1.000	1.000	1.000	1.000
843	1.000	1.000	1.000	1.000	1.000	1.000
844	1.000	1.000	1.000	1.000	1.000	1.000
845	1.000	1.000	1.000	1.000	1.000	1.000
846	1.000	1.000	1.000	1.000	1.000	1.000
847	1.000	1.000	1.000	1.000	1.000	1.000
848	1.000	1.000	1.000	1.000	1.000	1.000
849	1.000	1.000	1.000	1.000	1.000	1.000
850	1.000	1.000	1.000	1.000	1.000	1.000
851	1.000	1.000	1.000	1.000	1.000	1.000
852	1.000	1.000	1.000	1.000	1.000	1.000
853	1.000	1.000	1.000	1.000	1.000	1.000
854	1.000	1.000	1.000	1.000	1.000	1.000
855	1.000	1.000	1.000	1.000	1.000	1.000
856	1.000	1.000	1.000	1.000	1.000	1.000
857	1.000	1.000	1.000	1.000	1.000	1.000
858	1.000	1.000	1.000	1.000	1.000	1.000
859	1.000	1.000	1.000	1.000	1.000	1.000
860	1.000	1.000	1.000	1.000	1.000	1.000
861	1.000	1.000	1.000	1.000	1.000	1.000
862	1.000	1.000	1.000	1.000	1.000	1.000
863	1.000	1.000	1.000	1.000	1.000	1.000
864	1.000	1.000	1.000	1.000	1.000	1.000
865	1.000	1.000	1.000	1.000	1.000	1.000
866	1.000	1.000	1.000	1.000	1.000	1.000
867	1.000	1.000	1.000	1.000	1.000	1.000
868	1.000	1.000	1.000	1.000	1.000	1.000
869	1.000	1.000	1.000	1.000	1.000	1.000
870	1.000	1.000	1.000	1.000	1.000	1.000
871	1.000	1.000	1.000	1.000	1.000	1.000
872	1.000	1.000	1.000	1.000	1.000	1.000
873	1.000	1.000	1.000	1.000	1.000	1.000
874	1.000	1.000	1.000	1.000	1.000	1.000
875	1.000	1.000	1.000	1.000	1.000	1.000

876	1.000	1.000	1.000	1.000	1.000	1.000
877	1.000	1.000	1.000	1.000	1.000	1.000
878	1.000	1.000	1.000	1.000	1.000	1.000
879	1.000	1.000	1.000	1.000	1.000	1.000
880	1.000	1.000	1.000	1.000	1.000	1.000
881	1.000	1.000	1.000	1.000	1.000	1.000
882	1.000	1.000	1.000	1.000	1.000	1.000
883	1.000	1.000	1.000	1.000	1.000	1.000
884	1.000	1.000	1.000	1.000	1.000	1.000
885	1.000	1.000	1.000	1.000	1.000	1.000
886	1.000	1.000	1.000	1.000	1.000	1.000
887	1.000	1.000	1.000	1.000	1.000	1.000
888	1.000	1.000	1.000	1.000	1.000	1.000
889	1.000	1.000	1.000	1.000	1.000	1.000
890	1.000	1.000	1.000	1.000	1.000	1.000
891	1.000	1.000	1.000	1.000	1.000	1.000
892	1.000	1.000	1.000	1.000	1.000	1.000
893	1.000	1.000	1.000	1.000	1.000	1.000
894	1.000	1.000	1.000	1.000	1.000	1.000
895	1.000	1.000	1.000	1.000	1.000	1.000
896	1.000	1.000	1.000	1.000	1.000	1.000
897	1.000	1.000	1.000	1.000	1.000	1.000
898	1.000	1.000	1.000	1.000	1.000	1.000
899	1.000	1.000	1.000	1.000	1.000	1.000
900	1.000	1.000	1.000	1.000	1.000	1.000
901	1.000	1.000	1.000	1.000	1.000	1.000
902	1.000	1.000	1.000	1.000	1.000	1.000
903	1.000	1.000	1.000	1.000	1.000	1.000
904	1.000	1.000	1.000	1.000	1.000	1.000
905	1.000	1.000	1.000	1.000	1.000	1.000
906	1.000	1.000	1.000	1.000	1.000	1.000
907	1.000	1.000	1.000	1.000	1.000	1.000
908	1.000	1.000	1.000	1.000	1.000	1.000
909	1.000	1.000	1.000	1.000	1.000	1.000
910	1.000	1.000	1.000	1.000	1.000	1.000
911	1.000	1.000	1.000	1.000	1.000	1.000
912	1.000	1.000	1.000	1.000	1.000	1.000
913	1.000	1.000	1.000	1.000	1.000	1.000
914	1.000	1.000	1.000	1.000	1.000	1.000
915	1.000	1.000	1.000	1.000	1.000	1.000
916	1.000	1.000	1.000	1.000	1.000	1.000
917	1.000	1.000	1.000	1.000	1.000	1.000

918	1.000	1.000	1.000	1.000	1.000	1.000
919	1.000	1.000	1.000	1.000	1.000	1.000
920	1.000	1.000	1.000	1.000	1.000	1.000
921	1.000	1.000	1.000	1.000	1.000	1.000
922	1.000	1.000	1.000	1.000	1.000	1.000
923	1.000	1.000	1.000	1.000	1.000	1.000
924	1.000	1.000	1.000	1.000	1.000	1.000
925	1.000	1.000	1.000	1.000	1.000	1.000
926	1.000	1.000	1.000	1.000	1.000	1.000
927	1.000	1.000	1.000	1.000	1.000	1.000
928	1.000	1.000	1.000	1.000	1.000	1.000
929	1.000	1.000	1.000	1.000	1.000	1.000
930	1.000	1.000	1.000	1.000	1.000	1.000
931	1.000	1.000	1.000	1.000	1.000	1.000
932	1.000	1.000	1.000	1.000	1.000	1.000
933	1.000	1.000	1.000	1.000	1.000	1.000
934	1.000	1.000	1.000	1.000	1.000	1.000
935	1.000	1.000	1.000	1.000	1.000	1.000
936	1.000	1.000	1.000	1.000	1.000	1.000
937	1.000	1.000	1.000	1.000	1.000	1.000
938	1.000	1.000	1.000	1.000	1.000	1.000
939	1.000	1.000	1.000	1.000	1.000	1.000
940	1.000	1.000	1.000	1.000	1.000	1.000
941	1.000	1.000	1.000	1.000	1.000	1.000
942	1.000	1.000	1.000	1.000	1.000	1.000
943	1.000	1.000	1.000	1.000	1.000	1.000
944	1.000	1.000	1.000	1.000	1.000	1.000
945	1.000	1.000	1.000	1.000	1.000	1.000
946	1.000	1.000	1.000	1.000	1.000	1.000
947	1.000	1.000	1.000	1.000	1.000	1.000
948	1.000	1.000	1.000	1.000	1.000	1.000
949	1.000	1.000	1.000	1.000	1.000	1.000
950	1.000	1.000	1.000	1.000	1.000	1.000
951	1.000	1.000	1.000	1.000	1.000	1.000
952	1.000	1.000	1.000	1.000	1.000	1.000
953	1.000	1.000	1.000	1.000	1.000	1.000
954	1.000	1.000	1.000	1.000	1.000	1.000
955	1.000	1.000	1.000	1.000	1.000	1.000
956	1.000	1.000	1.000	1.000	1.000	1.000
957	1.000	1.000	1.000	1.000	1.000	1.000
958	1.000	1.000	1.000	1.000	1.000	1.000
959	1.000	1.000	1.000	1.000	1.000	1.000

960	1.000	1.000	1.000	1.000	1.000	1.000
961	1.000	1.000	1.000	1.000	1.000	1.000
962	1.000	1.000	1.000	1.000	1.000	1.000
963	1.000	1.000	1.000	1.000	1.000	1.000
964	1.000	1.000	1.000	1.000	1.000	1.000
965	1.000	1.000	1.000	1.000	1.000	1.000
966	1.000	1.000	1.000	1.000	1.000	1.000
967	1.000	1.000	1.000	1.000	1.000	1.000
968	1.000	1.000	1.000	1.000	1.000	1.000
969	1.000	1.000	1.000	1.000	1.000	1.000
970	1.000	1.000	1.000	1.000	1.000	1.000
971	1.000	1.000	1.000	1.000	1.000	1.000
972	1.000	1.000	1.000	1.000	1.000	1.000
973	1.000	1.000	1.000	1.000	1.000	1.000
974	1.000	1.000	1.000	1.000	1.000	1.000
975	1.000	1.000	1.000	1.000	1.000	1.000
976	1.000	1.000	1.000	1.000	1.000	1.000
977	1.000	1.000	1.000	1.000	1.000	1.000
978	1.000	1.000	1.000	1.000	1.000	1.000
979	1.000	1.000	1.000	1.000	1.000	1.000
980	1.000	1.000	1.000	1.000	1.000	1.000
981	1.000	1.000	1.000	1.000	1.000	1.000
982	1.000	1.000	1.000	1.000	1.000	1.000
983	1.000	1.000	1.000	1.000	1.000	1.000
984	1.000	1.000	1.000	1.000	1.000	1.000
985	1.000	1.000	1.000	1.000	1.000	1.000
986	1.000	1.000	1.000	1.000	1.000	1.000
987	1.000	1.000	1.000	1.000	1.000	1.000
988	1.000	1.000	1.000	1.000	1.000	1.000
989	1.000	1.000	1.000	1.000	1.000	1.000
990	1.000	1.000	1.000	1.000	1.000	1.000
991	1.000	1.000	1.000	1.000	1.000	1.000
992	1.000	1.000	1.000	1.000	1.000	1.000
993	1.000	1.000	1.000	1.000	1.000	1.000
994	1.000	1.000	1.000	1.000	1.000	1.000
995	1.000	1.000	1.000	1.000	1.000	1.000
996	1.000	1.000	1.000	1.000	1.000	1.000
997	1.000	1.000	1.000	1.000	1.000	1.000
998	1.000	1.000	1.000	1.000	1.000	1.000
999	1.000	1.000	1.000	1.000	1.000	1.000
1000	1.000	1.000	1.000	1.000	1.000	1.000
1001	1.000	1.000	1.000	1.000	1.000	1.000

1002	1.000	1.000	1.000	1.000	1.000	1.000
1003	1.000	1.000	1.000	1.000	1.000	1.000
1004	1.000	1.000	1.000	1.000	1.000	1.000
1005	1.000	1.000	1.000	1.000	1.000	1.000
1006	1.000	1.000	1.000	1.000	1.000	1.000
1007	1.000	1.000	1.000	1.000	1.000	1.000
1008	1.000	1.000	1.000	1.000	1.000	1.000
1009	1.000	1.000	1.000	1.000	1.000	1.000
1010	1.000	1.000	1.000	1.000	1.000	1.000
1011	1.000	1.000	1.000	1.000	1.000	1.000
1012	1.000	1.000	1.000	1.000	1.000	1.000
1013	1.000	1.000	1.000	1.000	1.000	1.000
1014	1.000	1.000	1.000	1.000	1.000	1.000
1015	1.000	1.000	1.000	1.000	1.000	1.000
1016	1.000	1.000	1.000	1.000	1.000	1.000
1017	1.000	1.000	1.000	1.000	1.000	1.000
1018	1.000	1.000	1.000	1.000	1.000	1.000
1019	1.000	1.000	1.000	1.000	1.000	1.000
1020	1.000	1.000	1.000	1.000	1.000	1.000
