# Supplementary material for: Predicting RNA 5-Methylcytosine Sites by Using Essential Sequence Features and Distributions
Source: Biomed Res Int. 2022 Jan 13;2022:4035462. doi: 10.1155/2022/4035462 (PMC8776474; doi:10.1155/2022/4035462)
Supplement: Supplementary Materials — Supplementary Material S1: performance of IFS for mouse m5C sites. Supplementary Material S2: performance of IFS for human m5C sites. [file 4035462.f1.zip › Supp-S2 (1).pdf]

Supplementary Material S2. Performance of IFS for human m^{5}C sites.

(1) Decision tree

Number of features	SN	SP	ACC	MCC	Precision	F1-measure
1	0.608	0.525	0.567	0.134	0.562	0.584
2	0.667	0.633	0.650	0.300	0.645	0.656
3	0.683	0.608	0.646	0.292	0.636	0.659
4	0.717	0.717	0.717	0.433	0.717	0.717
5	0.750	0.775	0.763	0.525	0.769	0.759
6	0.725	0.708	0.717	0.433	0.713	0.719
7	0.717	0.750	0.733	0.467	0.741	0.729
8	0.742	0.733	0.738	0.475	0.736	0.739
9	0.783	0.717	0.750	0.501	0.734	0.758
10	0.775	0.675	0.725	0.452	0.705	0.738
11	0.708	0.683	0.696	0.392	0.691	0.700
12	0.733	0.758	0.746	0.492	0.752	0.743
13	0.750	0.783	0.767	0.534	0.776	0.763
14	0.725	0.767	0.746	0.492	0.757	0.740
15	0.767	0.808	0.788	0.576	0.800	0.783
16	0.767	0.800	0.783	0.567	0.793	0.780
17	0.733	0.783	0.758	0.517	0.772	0.752
18	0.725	0.742	0.733	0.467	0.737	0.731
19	0.708	0.733	0.721	0.442	0.726	0.717
20	0.750	0.708	0.729	0.459	0.720	0.735
21	0.750	0.692	0.721	0.442	0.709	0.729
22	0.775	0.717	0.746	0.493	0.732	0.753
23	0.750	0.708	0.729	0.459	0.720	0.735
24	0.683	0.725	0.704	0.409	0.713	0.698
25	0.733	0.717	0.725	0.450	0.721	0.727
26	0.742	0.700	0.721	0.442	0.712	0.727
27	0.700	0.692	0.696	0.392	0.694	0.697
28	0.733	0.700	0.717	0.434	0.710	0.721
29	0.725	0.692	0.708	0.417	0.702	0.713
30	0.692	0.650	0.671	0.342	0.664	0.678
31	0.708	0.683	0.696	0.392	0.691	0.700
32	0.717	0.708	0.713	0.425	0.711	0.714
33	0.708	0.717	0.713	0.425	0.714	0.711
34	0.667	0.708	0.688	0.375	0.696	0.681
35	0.633	0.750	0.692	0.386	0.717	0.673
36	0.683	0.650	0.667	0.334	0.661	0.672
37	0.667	0.708	0.688	0.375	0.696	0.681
38	0.658	0.725	0.692	0.384	0.705	0.681

39	0.683	0.725	0.704	0.409	0.713	0.698
40	0.725	0.700	0.713	0.425	0.707	0.716
41	0.700	0.725	0.713	0.425	0.718	0.709
42	0.667	0.683	0.675	0.350	0.678	0.672
43	0.658	0.650	0.654	0.308	0.653	0.656
44	0.675	0.683	0.679	0.358	0.681	0.678
45	0.658	0.700	0.679	0.359	0.687	0.672
46	0.633	0.725	0.679	0.360	0.697	0.664
47	0.667	0.708	0.688	0.375	0.696	0.681
48	0.667	0.725	0.696	0.392	0.708	0.687
49	0.692	0.683	0.688	0.375	0.686	0.689
50	0.667	0.667	0.667	0.333	0.667	0.667
51	0.708	0.708	0.708	0.417	0.708	0.708
52	0.667	0.683	0.675	0.350	0.678	0.672
53	0.675	0.667	0.671	0.342	0.669	0.672
54	0.683	0.683	0.683	0.367	0.683	0.683
55	0.633	0.667	0.650	0.300	0.655	0.644
56	0.675	0.683	0.679	0.358	0.681	0.678
57	0.692	0.675	0.683	0.367	0.680	0.686
58	0.742	0.683	0.713	0.426	0.701	0.721
59	0.708	0.667	0.688	0.375	0.680	0.694
60	0.692	0.667	0.679	0.358	0.675	0.683
61	0.675	0.717	0.696	0.392	0.704	0.689
62	0.700	0.700	0.700	0.400	0.700	0.700
63	0.700	0.700	0.700	0.400	0.700	0.700
64	0.683	0.725	0.704	0.409	0.713	0.698
65	0.708	0.675	0.692	0.384	0.685	0.697
66	0.708	0.692	0.700	0.400	0.697	0.702
67	0.683	0.692	0.688	0.375	0.689	0.686
68	0.683	0.667	0.675	0.350	0.672	0.678
69	0.683	0.708	0.696	0.392	0.701	0.692
70	0.700	0.708	0.704	0.408	0.706	0.703
71	0.633	0.742	0.688	0.377	0.710	0.670
72	0.617	0.725	0.671	0.344	0.692	0.652
73	0.650	0.700	0.675	0.350	0.684	0.667
74	0.683	0.725	0.704	0.409	0.713	0.698
75	0.625	0.758	0.692	0.387	0.721	0.670
76	0.642	0.700	0.671	0.342	0.681	0.661
77	0.642	0.733	0.688	0.377	0.706	0.672
78	0.608	0.692	0.650	0.301	0.664	0.635
79	0.600	0.692	0.646	0.293	0.661	0.629
80	0.642	0.675	0.658	0.317	0.664	0.653

81	0.625	0.683	0.654	0.309	0.664	0.644
82	0.675	0.733	0.704	0.409	0.717	0.695
83	0.650	0.733	0.692	0.385	0.709	0.678
84	0.658	0.692	0.675	0.350	0.681	0.669
85	0.625	0.750	0.688	0.378	0.714	0.667
86	0.658	0.708	0.683	0.367	0.693	0.675
87	0.633	0.675	0.654	0.309	0.661	0.647
88	0.642	0.675	0.658	0.317	0.664	0.653
89	0.642	0.717	0.679	0.359	0.694	0.667
90	0.608	0.725	0.667	0.336	0.689	0.646
91	0.667	0.717	0.692	0.384	0.702	0.684
92	0.675	0.717	0.696	0.392	0.704	0.689
93	0.642	0.717	0.679	0.359	0.694	0.667
94	0.650	0.675	0.663	0.325	0.667	0.658
95	0.667	0.683	0.675	0.350	0.678	0.672
96	0.617	0.692	0.654	0.309	0.667	0.641
97	0.650	0.708	0.679	0.359	0.690	0.670
98	0.650	0.733	0.692	0.385	0.709	0.678
99	0.650	0.708	0.679	0.359	0.690	0.670
100	0.625	0.683	0.654	0.309	0.664	0.644
101	0.667	0.692	0.679	0.358	0.684	0.675
102	0.675	0.700	0.688	0.375	0.692	0.684
103	0.683	0.683	0.683	0.367	0.683	0.683
104	0.667	0.675	0.671	0.342	0.672	0.669
105	0.667	0.667	0.667	0.333	0.667	0.667
106	0.658	0.675	0.667	0.333	0.669	0.664
107	0.658	0.658	0.658	0.317	0.658	0.658
108	0.625	0.675	0.650	0.300	0.658	0.641
109	0.667	0.683	0.675	0.350	0.678	0.672
110	0.625	0.658	0.642	0.283	0.647	0.636
111	0.592	0.683	0.638	0.276	0.651	0.620
112	0.683	0.742	0.713	0.426	0.726	0.704
113	0.642	0.700	0.671	0.342	0.681	0.661
114	0.675	0.750	0.713	0.426	0.730	0.701
115	0.667	0.733	0.700	0.401	0.714	0.690
116	0.650	0.750	0.700	0.402	0.722	0.684
117	0.658	0.725	0.692	0.384	0.705	0.681
118	0.667	0.742	0.704	0.409	0.721	0.693
119	0.658	0.708	0.683	0.367	0.693	0.675
120	0.675	0.733	0.704	0.409	0.717	0.695
121	0.642	0.717	0.679	0.359	0.694	0.667
122	0.658	0.725	0.692	0.384	0.705	0.681

123	0.667	0.750	0.708	0.418	0.727	0.696
124	0.675	0.717	0.696	0.392	0.704	0.689
125	0.675	0.733	0.704	0.409	0.717	0.695
126	0.692	0.708	0.700	0.400	0.703	0.697
127	0.675	0.742	0.708	0.418	0.723	0.698
128	0.633	0.750	0.692	0.386	0.717	0.673
129	0.675	0.733	0.704	0.409	0.717	0.695
130	0.642	0.733	0.688	0.377	0.706	0.672
131	0.758	0.750	0.754	0.508	0.752	0.755
132	0.742	0.742	0.742	0.483	0.742	0.742
133	0.717	0.733	0.725	0.450	0.729	0.723
134	0.733	0.725	0.729	0.458	0.727	0.730
135	0.725	0.717	0.721	0.442	0.719	0.722
136	0.717	0.750	0.733	0.467	0.741	0.729
137	0.767	0.708	0.738	0.476	0.724	0.745
138	0.758	0.733	0.746	0.492	0.740	0.749
139	0.733	0.742	0.738	0.475	0.739	0.736
140	0.733	0.700	0.717	0.434	0.710	0.721
141	0.758	0.758	0.758	0.517	0.758	0.758
142	0.767	0.700	0.733	0.468	0.719	0.742
143	0.733	0.708	0.721	0.442	0.715	0.724
144	0.758	0.692	0.725	0.451	0.711	0.734
145	0.750	0.700	0.725	0.451	0.714	0.732
146	0.758	0.725	0.742	0.484	0.734	0.746
147	0.750	0.733	0.742	0.483	0.738	0.744
148	0.717	0.733	0.725	0.450	0.729	0.723
149	0.733	0.700	0.717	0.434	0.710	0.721
150	0.742	0.733	0.738	0.475	0.736	0.739
151	0.742	0.717	0.729	0.458	0.724	0.733
152	0.758	0.708	0.733	0.467	0.722	0.740
153	0.733	0.700	0.717	0.434	0.710	0.721
154	0.767	0.675	0.721	0.444	0.702	0.733
155	0.725	0.725	0.725	0.450	0.725	0.725
156	0.750	0.675	0.713	0.426	0.698	0.723
157	0.742	0.717	0.729	0.458	0.724	0.733
158	0.750	0.725	0.738	0.475	0.732	0.741
159	0.750	0.700	0.725	0.451	0.714	0.732
160	0.758	0.708	0.733	0.467	0.722	0.740
161	0.725	0.725	0.725	0.450	0.725	0.725
162	0.733	0.725	0.729	0.458	0.727	0.730
163	0.750	0.725	0.738	0.475	0.732	0.741
164	0.733	0.733	0.733	0.467	0.733	0.733

165	0.708	0.692	0.700	0.400	0.697	0.702
166	0.708	0.708	0.708	0.417	0.708	0.708
167	0.717	0.733	0.725	0.450	0.729	0.723
168	0.742	0.683	0.713	0.426	0.701	0.721
169	0.717	0.725	0.721	0.442	0.723	0.720
170	0.750	0.692	0.721	0.442	0.709	0.729
171	0.700	0.750	0.725	0.451	0.737	0.718
172	0.742	0.742	0.742	0.483	0.742	0.742
173	0.733	0.675	0.704	0.409	0.693	0.713
174	0.733	0.717	0.725	0.450	0.721	0.727
175	0.742	0.700	0.721	0.442	0.712	0.727
176	0.733	0.717	0.725	0.450	0.721	0.727
177	0.717	0.683	0.700	0.400	0.694	0.705
178	0.725	0.717	0.721	0.442	0.719	0.722
179	0.733	0.742	0.738	0.475	0.739	0.736
180	0.758	0.742	0.750	0.500	0.746	0.752
181	0.700	0.717	0.708	0.417	0.712	0.706
182	0.717	0.717	0.717	0.433	0.717	0.717
183	0.742	0.717	0.729	0.458	0.724	0.733
184	0.717	0.750	0.733	0.467	0.741	0.729
185	0.758	0.717	0.738	0.475	0.728	0.743
186	0.725	0.650	0.688	0.376	0.674	0.699
187	0.717	0.750	0.733	0.467	0.741	0.729
188	0.700	0.717	0.708	0.417	0.712	0.706
189	0.733	0.708	0.721	0.442	0.715	0.724
190	0.750	0.717	0.733	0.467	0.726	0.738
191	0.742	0.700	0.721	0.442	0.712	0.727
192	0.725	0.717	0.721	0.442	0.719	0.722
193	0.717	0.733	0.725	0.450	0.729	0.723
194	0.700	0.700	0.700	0.400	0.700	0.700
195	0.675	0.750	0.713	0.426	0.730	0.701
196	0.717	0.683	0.700	0.400	0.694	0.705
197	0.675	0.742	0.708	0.418	0.723	0.698
198	0.717	0.667	0.692	0.384	0.683	0.699
199	0.733	0.708	0.721	0.442	0.715	0.724
200	0.708	0.675	0.692	0.384	0.685	0.697
201	0.708	0.683	0.696	0.392	0.691	0.700
202	0.717	0.717	0.717	0.433	0.717	0.717
203	0.717	0.683	0.700	0.400	0.694	0.705
204	0.692	0.708	0.700	0.400	0.703	0.697
205	0.708	0.683	0.696	0.392	0.691	0.700
206	0.708	0.658	0.683	0.367	0.675	0.691

207	0.708	0.742	0.725	0.450	0.733	0.720
208	0.675	0.725	0.700	0.401	0.711	0.692
209	0.708	0.733	0.721	0.442	0.726	0.717
210	0.675	0.683	0.679	0.358	0.681	0.678
211	0.708	0.700	0.704	0.408	0.702	0.705
212	0.733	0.717	0.725	0.450	0.721	0.727
213	0.667	0.675	0.671	0.342	0.672	0.669
214	0.683	0.708	0.696	0.392	0.701	0.692
215	0.717	0.658	0.688	0.376	0.677	0.696
216	0.700	0.708	0.704	0.408	0.706	0.703
217	0.667	0.700	0.683	0.367	0.690	0.678
218	0.717	0.675	0.696	0.392	0.688	0.702
219	0.642	0.733	0.688	0.377	0.706	0.672
220	0.717	0.692	0.704	0.408	0.699	0.708
221	0.700	0.758	0.729	0.459	0.743	0.721
222	0.708	0.742	0.725	0.450	0.733	0.720
223	0.717	0.700	0.708	0.417	0.705	0.711
224	0.683	0.742	0.713	0.426	0.726	0.704
225	0.700	0.708	0.704	0.408	0.706	0.703
226	0.658	0.725	0.692	0.384	0.705	0.681
227	0.750	0.733	0.742	0.483	0.738	0.744
228	0.700	0.658	0.679	0.359	0.672	0.686
229	0.733	0.725	0.729	0.458	0.727	0.730
230	0.675	0.700	0.688	0.375	0.692	0.684
231	0.733	0.683	0.708	0.417	0.698	0.715
232	0.750	0.675	0.713	0.426	0.698	0.723
233	0.675	0.742	0.708	0.418	0.723	0.698
234	0.725	0.708	0.717	0.433	0.713	0.719
235	0.725	0.717	0.721	0.442	0.719	0.722
236	0.692	0.717	0.704	0.408	0.709	0.700
237	0.700	0.725	0.713	0.425	0.718	0.709
238	0.708	0.700	0.704	0.408	0.702	0.705
239	0.683	0.733	0.708	0.417	0.719	0.701
240	0.708	0.708	0.708	0.417	0.708	0.708
241	0.708	0.717	0.713	0.425	0.714	0.711
242	0.725	0.692	0.708	0.417	0.702	0.713
243	0.708	0.758	0.733	0.467	0.746	0.726
244	0.675	0.708	0.692	0.384	0.698	0.686
245	0.708	0.708	0.708	0.417	0.708	0.708
246	0.717	0.742	0.729	0.458	0.735	0.726
247	0.700	0.708	0.704	0.408	0.706	0.703
248	0.733	0.708	0.721	0.442	0.715	0.724

249	0.717	0.700	0.708	0.417	0.705	0.711
250	0.692	0.717	0.704	0.408	0.709	0.700
251	0.700	0.667	0.683	0.367	0.677	0.689
252	0.733	0.708	0.721	0.442	0.715	0.724
253	0.717	0.750	0.733	0.467	0.741	0.729
254	0.717	0.742	0.729	0.458	0.735	0.726
255	0.708	0.733	0.721	0.442	0.726	0.717
256	0.733	0.700	0.717	0.434	0.710	0.721
257	0.708	0.683	0.696	0.392	0.691	0.700
258	0.742	0.717	0.729	0.458	0.724	0.733
259	0.733	0.708	0.721	0.442	0.715	0.724
260	0.642	0.708	0.675	0.351	0.688	0.664
261	0.725	0.683	0.704	0.409	0.696	0.710
262	0.717	0.767	0.742	0.484	0.754	0.735
263	0.700	0.708	0.704	0.408	0.706	0.703
264	0.700	0.708	0.704	0.408	0.706	0.703
265	0.700	0.733	0.717	0.434	0.724	0.712
266	0.750	0.683	0.717	0.434	0.703	0.726
267	0.692	0.725	0.708	0.417	0.716	0.703
268	0.708	0.708	0.708	0.417	0.708	0.708
269	0.717	0.708	0.713	0.425	0.711	0.714
270	0.700	0.700	0.700	0.400	0.700	0.700
271	0.700	0.700	0.700	0.400	0.700	0.700
272	0.717	0.733	0.725	0.450	0.729	0.723
273	0.742	0.683	0.713	0.426	0.701	0.721
274	0.708	0.725	0.717	0.433	0.720	0.714
275	0.700	0.733	0.717	0.434	0.724	0.712
276	0.708	0.700	0.704	0.408	0.702	0.705
277	0.733	0.733	0.733	0.467	0.733	0.733
278	0.742	0.750	0.746	0.492	0.748	0.745
279	0.742	0.692	0.717	0.434	0.706	0.724
280	0.717	0.708	0.713	0.425	0.711	0.714
281	0.725	0.733	0.729	0.458	0.731	0.728
282	0.708	0.683	0.696	0.392	0.691	0.700
283	0.725	0.692	0.708	0.417	0.702	0.713
284	0.708	0.708	0.708	0.417	0.708	0.708
285	0.717	0.725	0.721	0.442	0.723	0.720
286	0.675	0.692	0.683	0.367	0.686	0.681
287	0.725	0.658	0.692	0.384	0.680	0.702
288	0.742	0.717	0.729	0.458	0.724	0.733
289	0.708	0.708	0.708	0.417	0.708	0.708
290	0.725	0.708	0.717	0.433	0.713	0.719

291	0.725	0.708	0.717	0.433	0.713	0.719
292	0.725	0.683	0.704	0.409	0.696	0.710
293	0.692	0.700	0.696	0.392	0.697	0.695
294	0.667	0.725	0.696	0.392	0.708	0.687
295	0.717	0.717	0.717	0.433	0.717	0.717
296	0.733	0.708	0.721	0.442	0.715	0.724
297	0.725	0.708	0.717	0.433	0.713	0.719
298	0.725	0.708	0.717	0.433	0.713	0.719
299	0.725	0.700	0.713	0.425	0.707	0.716
300	0.717	0.683	0.700	0.400	0.694	0.705
301	0.683	0.717	0.700	0.400	0.707	0.695
302	0.725	0.683	0.704	0.409	0.696	0.710
303	0.675	0.708	0.692	0.384	0.698	0.686
304	0.692	0.692	0.692	0.383	0.692	0.692
305	0.658	0.708	0.683	0.367	0.693	0.675
306	0.742	0.658	0.700	0.401	0.685	0.712
307	0.733	0.700	0.717	0.434	0.710	0.721
308	0.717	0.692	0.704	0.408	0.699	0.708
309	0.750	0.692	0.721	0.442	0.709	0.729
310	0.717	0.700	0.708	0.417	0.705	0.711
311	0.742	0.692	0.717	0.434	0.706	0.724
312	0.683	0.717	0.700	0.400	0.707	0.695
313	0.742	0.700	0.721	0.442	0.712	0.727
314	0.717	0.733	0.725	0.450	0.729	0.723
315	0.700	0.692	0.696	0.392	0.694	0.697
316	0.717	0.683	0.700	0.400	0.694	0.705
317	0.733	0.742	0.738	0.475	0.739	0.736
318	0.708	0.733	0.721	0.442	0.726	0.717
319	0.725	0.692	0.708	0.417	0.702	0.713
320	0.708	0.683	0.696	0.392	0.691	0.700
321	0.717	0.683	0.700	0.400	0.694	0.705
322	0.683	0.683	0.683	0.367	0.683	0.683
323	0.700	0.708	0.704	0.408	0.706	0.703
324	0.733	0.675	0.704	0.409	0.693	0.713
325	0.667	0.692	0.679	0.358	0.684	0.675
326	0.717	0.717	0.717	0.433	0.717	0.717
327	0.725	0.692	0.708	0.417	0.702	0.713
328	0.717	0.725	0.721	0.442	0.723	0.720
329	0.725	0.708	0.717	0.433	0.713	0.719
330	0.708	0.708	0.708	0.417	0.708	0.708
331	0.717	0.725	0.721	0.442	0.723	0.720
332	0.717	0.700	0.708	0.417	0.705	0.711

333	0.717	0.700	0.708	0.417	0.705	0.711
334	0.725	0.708	0.717	0.433	0.713	0.719
335	0.733	0.692	0.713	0.425	0.704	0.718
336	0.700	0.675	0.688	0.375	0.683	0.691
337	0.692	0.675	0.683	0.367	0.680	0.686
338	0.717	0.675	0.696	0.392	0.688	0.702
339	0.667	0.717	0.692	0.384	0.702	0.684
340	0.700	0.708	0.704	0.408	0.706	0.703
341	0.733	0.708	0.721	0.442	0.715	0.724
342	0.692	0.692	0.692	0.383	0.692	0.692
343	0.717	0.683	0.700	0.400	0.694	0.705
344	0.667	0.675	0.671	0.342	0.672	0.669
345	0.708	0.692	0.700	0.400	0.697	0.702
346	0.742	0.700	0.721	0.442	0.712	0.727
347	0.675	0.692	0.683	0.367	0.686	0.681
348	0.683	0.700	0.692	0.383	0.695	0.689
349	0.708	0.717	0.713	0.425	0.714	0.711
350	0.700	0.642	0.671	0.342	0.661	0.680
351	0.667	0.683	0.675	0.350	0.678	0.672
352	0.675	0.733	0.704	0.409	0.717	0.695
353	0.725	0.683	0.704	0.409	0.696	0.710
354	0.717	0.658	0.688	0.376	0.677	0.696
355	0.717	0.667	0.692	0.384	0.683	0.699
356	0.708	0.700	0.704	0.408	0.702	0.705
357	0.708	0.658	0.683	0.367	0.675	0.691
358	0.733	0.650	0.692	0.385	0.677	0.704
359	0.742	0.658	0.700	0.401	0.685	0.712
360	0.725	0.650	0.688	0.376	0.674	0.699
361	0.733	0.700	0.717	0.434	0.710	0.721
362	0.700	0.675	0.688	0.375	0.683	0.691
363	0.717	0.675	0.696	0.392	0.688	0.702
364	0.708	0.675	0.692	0.384	0.685	0.697
365	0.725	0.692	0.708	0.417	0.702	0.713
366	0.692	0.675	0.683	0.367	0.680	0.686
367	0.700	0.675	0.688	0.375	0.683	0.691
368	0.733	0.667	0.700	0.401	0.688	0.710
369	0.725	0.692	0.708	0.417	0.702	0.713
370	0.725	0.700	0.713	0.425	0.707	0.716
371	0.708	0.650	0.679	0.359	0.669	0.688
372	0.708	0.650	0.679	0.359	0.669	0.688
373	0.700	0.692	0.696	0.392	0.694	0.697
374	0.708	0.642	0.675	0.351	0.664	0.685

375	0.725	0.650	0.688	0.376	0.674	0.699
376	0.750	0.667	0.708	0.418	0.692	0.720
377	0.708	0.683	0.696	0.392	0.691	0.700
378	0.717	0.667	0.692	0.384	0.683	0.699
379	0.725	0.658	0.692	0.384	0.680	0.702
380	0.733	0.642	0.688	0.377	0.672	0.701
381	0.692	0.725	0.708	0.417	0.716	0.703
382	0.717	0.717	0.717	0.433	0.717	0.717
383	0.725	0.650	0.688	0.376	0.674	0.699
384	0.692	0.700	0.696	0.392	0.697	0.695
385	0.750	0.717	0.733	0.467	0.726	0.738
386	0.742	0.658	0.700	0.401	0.685	0.712
387	0.725	0.692	0.708	0.417	0.702	0.713
388	0.742	0.692	0.717	0.434	0.706	0.724
389	0.692	0.667	0.679	0.358	0.675	0.683
390	0.658	0.667	0.663	0.325	0.664	0.661
391	0.667	0.708	0.688	0.375	0.696	0.681
392	0.742	0.658	0.700	0.401	0.685	0.712
393	0.667	0.717	0.692	0.384	0.702	0.684
394	0.675	0.633	0.654	0.309	0.648	0.661
395	0.742	0.675	0.708	0.418	0.695	0.718
396	0.708	0.658	0.683	0.367	0.675	0.691
397	0.700	0.658	0.679	0.359	0.672	0.686
398	0.692	0.675	0.683	0.367	0.680	0.686
399	0.733	0.717	0.725	0.450	0.721	0.727
400	0.675	0.692	0.683	0.367	0.686	0.681
401	0.725	0.683	0.704	0.409	0.696	0.710
402	0.717	0.692	0.704	0.408	0.699	0.708
403	0.717	0.683	0.700	0.400	0.694	0.705
404	0.658	0.717	0.688	0.376	0.699	0.678
405	0.692	0.633	0.663	0.326	0.654	0.672
406	0.683	0.692	0.688	0.375	0.689	0.686
407	0.692	0.658	0.675	0.350	0.669	0.680
408	0.708	0.667	0.688	0.375	0.680	0.694
409	0.683	0.650	0.667	0.334	0.661	0.672
410	0.675	0.692	0.683	0.367	0.686	0.681
411	0.650	0.675	0.663	0.325	0.667	0.658
412	0.717	0.692	0.704	0.408	0.699	0.708
413	0.675	0.700	0.688	0.375	0.692	0.684
414	0.725	0.667	0.696	0.392	0.685	0.704
415	0.675	0.692	0.683	0.367	0.686	0.681
416	0.708	0.625	0.667	0.334	0.654	0.680

417	0.708	0.667	0.688	0.375	0.680	0.694
418	0.708	0.683	0.696	0.392	0.691	0.700
419	0.700	0.667	0.683	0.367	0.677	0.689
420	0.717	0.675	0.696	0.392	0.688	0.702
421	0.700	0.692	0.696	0.392	0.694	0.697
422	0.683	0.650	0.667	0.334	0.661	0.672
423	0.700	0.658	0.679	0.359	0.672	0.686
424	0.700	0.683	0.692	0.383	0.689	0.694
425	0.675	0.683	0.679	0.358	0.681	0.678
426	0.692	0.658	0.675	0.350	0.669	0.680
427	0.717	0.683	0.700	0.400	0.694	0.705
428	0.700	0.692	0.696	0.392	0.694	0.697
429	0.708	0.658	0.683	0.367	0.675	0.691
430	0.717	0.683	0.700	0.400	0.694	0.705
431	0.658	0.667	0.663	0.325	0.664	0.661
432	0.700	0.667	0.683	0.367	0.677	0.689
433	0.692	0.692	0.692	0.383	0.692	0.692
434	0.692	0.675	0.683	0.367	0.680	0.686
435	0.692	0.675	0.683	0.367	0.680	0.686
436	0.717	0.675	0.696	0.392	0.688	0.702
437	0.692	0.692	0.692	0.383	0.692	0.692
438	0.683	0.658	0.671	0.342	0.667	0.675
439	0.700	0.700	0.700	0.400	0.700	0.700
440	0.683	0.650	0.667	0.334	0.661	0.672
441	0.700	0.650	0.675	0.350	0.667	0.683
442	0.717	0.633	0.675	0.351	0.662	0.688
443	0.692	0.625	0.658	0.317	0.648	0.669
444	0.650	0.700	0.675	0.350	0.684	0.667
445	0.642	0.675	0.658	0.317	0.664	0.653
446	0.675	0.642	0.658	0.317	0.653	0.664
447	0.683	0.667	0.675	0.350	0.672	0.678
448	0.675	0.700	0.688	0.375	0.692	0.684
449	0.667	0.675	0.671	0.342	0.672	0.669
450	0.692	0.650	0.671	0.342	0.664	0.678
451	0.658	0.667	0.663	0.325	0.664	0.661
452	0.717	0.633	0.675	0.351	0.662	0.688
453	0.675	0.683	0.679	0.358	0.681	0.678
454	0.683	0.692	0.688	0.375	0.689	0.686
455	0.708	0.683	0.696	0.392	0.691	0.700
456	0.692	0.650	0.671	0.342	0.664	0.678
457	0.683	0.642	0.663	0.325	0.656	0.669
458	0.717	0.683	0.700	0.400	0.694	0.705

459	0.708	0.675	0.692	0.384	0.685	0.697
460	0.667	0.675	0.671	0.342	0.672	0.669
461	0.725	0.675	0.700	0.401	0.690	0.707
462	0.658	0.700	0.679	0.359	0.687	0.672
463	0.725	0.667	0.696	0.392	0.685	0.704
464	0.692	0.683	0.688	0.375	0.686	0.689
465	0.692	0.683	0.688	0.375	0.686	0.689
466	0.692	0.667	0.679	0.358	0.675	0.683
467	0.692	0.675	0.683	0.367	0.680	0.686
468	0.683	0.683	0.683	0.367	0.683	0.683
469	0.692	0.658	0.675	0.350	0.669	0.680
470	0.692	0.667	0.679	0.358	0.675	0.683
471	0.650	0.700	0.675	0.350	0.684	0.667
472	0.667	0.675	0.671	0.342	0.672	0.669
473	0.667	0.667	0.667	0.333	0.667	0.667
474	0.683	0.650	0.667	0.334	0.661	0.672
475	0.692	0.650	0.671	0.342	0.664	0.678
476	0.717	0.658	0.688	0.376	0.677	0.696
477	0.683	0.658	0.671	0.342	0.667	0.675
478	0.675	0.667	0.671	0.342	0.669	0.672
479	0.692	0.683	0.688	0.375	0.686	0.689
480	0.708	0.692	0.700	0.400	0.697	0.702
481	0.683	0.658	0.671	0.342	0.667	0.675
482	0.700	0.658	0.679	0.359	0.672	0.686
483	0.708	0.650	0.679	0.359	0.669	0.688
484	0.642	0.642	0.642	0.283	0.642	0.642
485	0.683	0.675	0.679	0.358	0.678	0.680
486	0.667	0.667	0.667	0.333	0.667	0.667
487	0.658	0.683	0.671	0.342	0.675	0.667
488	0.717	0.650	0.683	0.367	0.672	0.694
489	0.708	0.675	0.692	0.384	0.685	0.697
490	0.692	0.675	0.683	0.367	0.680	0.686
491	0.608	0.658	0.633	0.267	0.640	0.624
492	0.692	0.667	0.679	0.358	0.675	0.683
493	0.675	0.692	0.683	0.367	0.686	0.681
494	0.683	0.667	0.675	0.350	0.672	0.678
495	0.733	0.658	0.696	0.393	0.682	0.707
496	0.700	0.675	0.688	0.375	0.683	0.691
497	0.675	0.675	0.675	0.350	0.675	0.675
498	0.667	0.658	0.663	0.325	0.661	0.664
499	0.667	0.667	0.667	0.333	0.667	0.667
500	0.700	0.658	0.679	0.359	0.672	0.686

501	0.700	0.658	0.679	0.359	0.672	0.686
502	0.700	0.675	0.688	0.375	0.683	0.691
503	0.692	0.658	0.675	0.350	0.669	0.680
504	0.708	0.617	0.663	0.326	0.649	0.677
505	0.708	0.683	0.696	0.392	0.691	0.700
506	0.725	0.700	0.713	0.425	0.707	0.716
507	0.667	0.642	0.654	0.308	0.650	0.658
508	0.692	0.583	0.638	0.277	0.624	0.656
509	0.675	0.658	0.667	0.333	0.664	0.669
510	0.683	0.692	0.688	0.375	0.689	0.686
511	0.667	0.667	0.667	0.333	0.667	0.667
512	0.708	0.650	0.679	0.359	0.669	0.688
513	0.700	0.675	0.688	0.375	0.683	0.691
514	0.658	0.650	0.654	0.308	0.653	0.656
515	0.692	0.658	0.675	0.350	0.669	0.680
516	0.625	0.692	0.658	0.317	0.670	0.647
517	0.675	0.625	0.650	0.300	0.643	0.659
518	0.650	0.667	0.658	0.317	0.661	0.655
519	0.708	0.683	0.696	0.392	0.691	0.700
520	0.667	0.667	0.667	0.333	0.667	0.667
521	0.708	0.708	0.708	0.417	0.708	0.708
522	0.658	0.650	0.654	0.308	0.653	0.656
523	0.675	0.658	0.667	0.333	0.664	0.669
524	0.675	0.617	0.646	0.292	0.638	0.656
525	0.700	0.625	0.663	0.326	0.651	0.675
526	0.700	0.642	0.671	0.342	0.661	0.680
527	0.650	0.675	0.663	0.325	0.667	0.658
528	0.708	0.658	0.683	0.367	0.675	0.691
529	0.692	0.700	0.696	0.392	0.697	0.695
530	0.700	0.658	0.679	0.359	0.672	0.686
531	0.658	0.658	0.658	0.317	0.658	0.658
532	0.717	0.667	0.692	0.384	0.683	0.699
533	0.667	0.675	0.671	0.342	0.672	0.669
534	0.683	0.658	0.671	0.342	0.667	0.675
535	0.708	0.658	0.683	0.367	0.675	0.691
536	0.667	0.683	0.675	0.350	0.678	0.672
537	0.717	0.667	0.692	0.384	0.683	0.699
538	0.625	0.667	0.646	0.292	0.652	0.638
539	0.725	0.650	0.688	0.376	0.674	0.699
540	0.700	0.675	0.688	0.375	0.683	0.691
541	0.700	0.658	0.679	0.359	0.672	0.686
542	0.683	0.675	0.679	0.358	0.678	0.680

543	0.700	0.650	0.675	0.350	0.667	0.683
544	0.675	0.675	0.675	0.350	0.675	0.675
545	0.667	0.675	0.671	0.342	0.672	0.669
546	0.700	0.650	0.675	0.350	0.667	0.683
547	0.658	0.692	0.675	0.350	0.681	0.669
548	0.642	0.625	0.633	0.267	0.631	0.636
549	0.708	0.642	0.675	0.351	0.664	0.685
550	0.667	0.667	0.667	0.333	0.667	0.667
551	0.708	0.650	0.679	0.359	0.669	0.688
552	0.692	0.650	0.671	0.342	0.664	0.678
553	0.692	0.650	0.671	0.342	0.664	0.678
554	0.650	0.650	0.650	0.300	0.650	0.650
555	0.692	0.675	0.683	0.367	0.680	0.686
556	0.700	0.658	0.679	0.359	0.672	0.686
557	0.675	0.642	0.658	0.317	0.653	0.664
558	0.658	0.683	0.671	0.342	0.675	0.667
559	0.683	0.675	0.679	0.358	0.678	0.680
560	0.700	0.633	0.667	0.334	0.656	0.677
561	0.675	0.633	0.654	0.309	0.648	0.661
562	0.658	0.625	0.642	0.283	0.637	0.648
563	0.667	0.633	0.650	0.300	0.645	0.656
564	0.683	0.650	0.667	0.334	0.661	0.672
565	0.675	0.700	0.688	0.375	0.692	0.684
566	0.717	0.650	0.683	0.367	0.672	0.694
567	0.658	0.650	0.654	0.308	0.653	0.656
568	0.708	0.642	0.675	0.351	0.664	0.685
569	0.733	0.625	0.679	0.360	0.662	0.696
570	0.692	0.642	0.667	0.334	0.659	0.675
571	0.700	0.642	0.671	0.342	0.661	0.680
572	0.700	0.683	0.692	0.383	0.689	0.694
573	0.675	0.633	0.654	0.309	0.648	0.661
574	0.683	0.667	0.675	0.350	0.672	0.678
575	0.733	0.633	0.683	0.369	0.667	0.698
576	0.675	0.608	0.642	0.284	0.633	0.653
577	0.642	0.617	0.629	0.258	0.626	0.634
578	0.725	0.667	0.696	0.392	0.685	0.704
579	0.667	0.633	0.650	0.300	0.645	0.656
580	0.708	0.683	0.696	0.392	0.691	0.700
581	0.700	0.633	0.667	0.334	0.656	0.677
582	0.683	0.617	0.650	0.301	0.641	0.661
583	0.700	0.642	0.671	0.342	0.661	0.680
584	0.700	0.650	0.675	0.350	0.667	0.683

585	0.717	0.683	0.700	0.400	0.694	0.705
586	0.692	0.650	0.671	0.342	0.664	0.678
587	0.658	0.667	0.663	0.325	0.664	0.661
588	0.717	0.642	0.679	0.359	0.667	0.691
589	0.667	0.658	0.663	0.325	0.661	0.664
590	0.650	0.675	0.663	0.325	0.667	0.658
591	0.650	0.667	0.658	0.317	0.661	0.655
592	0.683	0.625	0.654	0.309	0.646	0.664
593	0.708	0.675	0.692	0.384	0.685	0.697
594	0.633	0.658	0.646	0.292	0.650	0.641
595	0.692	0.633	0.663	0.326	0.654	0.672
596	0.692	0.650	0.671	0.342	0.664	0.678
597	0.675	0.642	0.658	0.317	0.653	0.664
598	0.675	0.650	0.663	0.325	0.659	0.667
599	0.700	0.625	0.663	0.326	0.651	0.675
600	0.675	0.675	0.675	0.350	0.675	0.675
601	0.658	0.658	0.658	0.317	0.658	0.658
602	0.642	0.633	0.638	0.275	0.636	0.639
603	0.650	0.683	0.667	0.334	0.672	0.661
604	0.683	0.650	0.667	0.334	0.661	0.672
605	0.667	0.633	0.650	0.300	0.645	0.656
606	0.650	0.650	0.650	0.300	0.650	0.650
607	0.667	0.683	0.675	0.350	0.678	0.672
608	0.692	0.683	0.688	0.375	0.686	0.689
609	0.675	0.600	0.638	0.276	0.628	0.651
610	0.658	0.650	0.654	0.308	0.653	0.656
611	0.692	0.675	0.683	0.367	0.680	0.686
612	0.692	0.683	0.688	0.375	0.686	0.689
613	0.717	0.675	0.696	0.392	0.688	0.702
614	0.675	0.667	0.671	0.342	0.669	0.672
615	0.683	0.608	0.646	0.292	0.636	0.659
616	0.675	0.625	0.650	0.300	0.643	0.659
617	0.683	0.650	0.667	0.334	0.661	0.672
618	0.692	0.642	0.667	0.334	0.659	0.675
619	0.667	0.642	0.654	0.308	0.650	0.658
620	0.667	0.600	0.633	0.267	0.625	0.645
621	0.692	0.667	0.679	0.358	0.675	0.683
622	0.700	0.658	0.679	0.359	0.672	0.686
623	0.692	0.667	0.679	0.358	0.675	0.683
624	0.683	0.667	0.675	0.350	0.672	0.678
625	0.658	0.642	0.650	0.300	0.648	0.653
626	0.717	0.625	0.671	0.343	0.656	0.685

627	0.625	0.683	0.654	0.309	0.664	0.644
628	0.658	0.642	0.650	0.300	0.648	0.653
629	0.700	0.625	0.663	0.326	0.651	0.675
630	0.692	0.667	0.679	0.358	0.675	0.683
631	0.708	0.650	0.679	0.359	0.669	0.688
632	0.700	0.683	0.692	0.383	0.689	0.694
633	0.692	0.683	0.688	0.375	0.686	0.689
634	0.700	0.633	0.667	0.334	0.656	0.677
635	0.650	0.633	0.642	0.283	0.639	0.645
636	0.667	0.642	0.654	0.308	0.650	0.658
637	0.708	0.625	0.667	0.334	0.654	0.680
638	0.700	0.625	0.663	0.326	0.651	0.675
639	0.692	0.608	0.650	0.301	0.638	0.664
640	0.692	0.683	0.688	0.375	0.686	0.689
641	0.667	0.667	0.667	0.333	0.667	0.667
642	0.700	0.667	0.683	0.367	0.677	0.689
643	0.658	0.642	0.650	0.300	0.648	0.653
644	0.700	0.675	0.688	0.375	0.683	0.691
645	0.683	0.683	0.683	0.367	0.683	0.683
646	0.675	0.683	0.679	0.358	0.681	0.678
647	0.658	0.650	0.654	0.308	0.653	0.656
648	0.692	0.667	0.679	0.358	0.675	0.683
649	0.692	0.625	0.658	0.317	0.648	0.669
650	0.675	0.642	0.658	0.317	0.653	0.664
651	0.667	0.675	0.671	0.342	0.672	0.669
652	0.667	0.683	0.675	0.350	0.678	0.672
653	0.700	0.625	0.663	0.326	0.651	0.675
654	0.675	0.642	0.658	0.317	0.653	0.664
655	0.683	0.692	0.688	0.375	0.689	0.686
656	0.650	0.675	0.663	0.325	0.667	0.658
657	0.650	0.667	0.658	0.317	0.661	0.655
658	0.700	0.658	0.679	0.359	0.672	0.686
659	0.675	0.600	0.638	0.276	0.628	0.651
660	0.667	0.642	0.654	0.308	0.650	0.658
661	0.675	0.658	0.667	0.333	0.664	0.669
662	0.692	0.658	0.675	0.350	0.669	0.680
663	0.692	0.600	0.646	0.293	0.634	0.661
664	0.650	0.633	0.642	0.283	0.639	0.645
665	0.683	0.667	0.675	0.350	0.672	0.678
666	0.667	0.633	0.650	0.300	0.645	0.656
667	0.658	0.658	0.658	0.317	0.658	0.658
668	0.617	0.633	0.625	0.250	0.627	0.622

669	0.708	0.625	0.667	0.334	0.654	0.680
670	0.683	0.608	0.646	0.292	0.636	0.659
671	0.692	0.625	0.658	0.317	0.648	0.669
672	0.708	0.650	0.679	0.359	0.669	0.688
673	0.708	0.650	0.679	0.359	0.669	0.688
674	0.633	0.617	0.625	0.250	0.623	0.628
675	0.692	0.667	0.679	0.358	0.675	0.683
676	0.683	0.625	0.654	0.309	0.646	0.664
677	0.700	0.642	0.671	0.342	0.661	0.680
678	0.658	0.633	0.646	0.292	0.642	0.650
679	0.675	0.625	0.650	0.300	0.643	0.659
680	0.708	0.675	0.692	0.384	0.685	0.697
681	0.708	0.642	0.675	0.351	0.664	0.685
682	0.708	0.683	0.696	0.392	0.691	0.700
683	0.708	0.642	0.675	0.351	0.664	0.685
684	0.717	0.658	0.688	0.376	0.677	0.696
685	0.692	0.642	0.667	0.334	0.659	0.675
686	0.675	0.692	0.683	0.367	0.686	0.681
687	0.675	0.658	0.667	0.333	0.664	0.669
688	0.708	0.625	0.667	0.334	0.654	0.680
689	0.683	0.642	0.663	0.325	0.656	0.669
690	0.675	0.700	0.688	0.375	0.692	0.684
691	0.675	0.625	0.650	0.300	0.643	0.659
692	0.658	0.658	0.658	0.317	0.658	0.658
693	0.708	0.600	0.654	0.310	0.639	0.672
694	0.708	0.667	0.688	0.375	0.680	0.694
695	0.683	0.658	0.671	0.342	0.667	0.675
696	0.675	0.633	0.654	0.309	0.648	0.661
697	0.667	0.633	0.650	0.300	0.645	0.656
698	0.683	0.650	0.667	0.334	0.661	0.672
699	0.725	0.650	0.688	0.376	0.674	0.699
700	0.717	0.650	0.683	0.367	0.672	0.694
701	0.650	0.675	0.663	0.325	0.667	0.658
702	0.717	0.642	0.679	0.359	0.667	0.691
703	0.667	0.692	0.679	0.358	0.684	0.675
704	0.675	0.675	0.675	0.350	0.675	0.675
705	0.642	0.650	0.646	0.292	0.647	0.644
706	0.642	0.675	0.658	0.317	0.664	0.653
707	0.675	0.658	0.667	0.333	0.664	0.669
708	0.667	0.642	0.654	0.308	0.650	0.658
709	0.675	0.650	0.663	0.325	0.659	0.667
710	0.675	0.658	0.667	0.333	0.664	0.669

711	0.642	0.642	0.642	0.283	0.642	0.642
712	0.658	0.642	0.650	0.300	0.648	0.653
713	0.675	0.642	0.658	0.317	0.653	0.664
714	0.650	0.658	0.654	0.308	0.655	0.653
715	0.725	0.658	0.692	0.384	0.680	0.702
716	0.658	0.667	0.663	0.325	0.664	0.661
717	0.667	0.692	0.679	0.358	0.684	0.675
718	0.692	0.633	0.663	0.326	0.654	0.672
719	0.667	0.583	0.625	0.251	0.615	0.640
720	0.700	0.658	0.679	0.359	0.672	0.686
721	0.700	0.650	0.675	0.350	0.667	0.683
722	0.683	0.650	0.667	0.334	0.661	0.672
723	0.692	0.658	0.675	0.350	0.669	0.680
724	0.683	0.667	0.675	0.350	0.672	0.678
725	0.658	0.650	0.654	0.308	0.653	0.656
726	0.692	0.625	0.658	0.317	0.648	0.669
727	0.658	0.633	0.646	0.292	0.642	0.650
728	0.717	0.650	0.683	0.367	0.672	0.694
729	0.675	0.650	0.663	0.325	0.659	0.667
730	0.650	0.642	0.646	0.292	0.645	0.647
731	0.683	0.667	0.675	0.350	0.672	0.678
732	0.667	0.625	0.646	0.292	0.640	0.653
733	0.717	0.667	0.692	0.384	0.683	0.699
734	0.658	0.633	0.646	0.292	0.642	0.650
735	0.675	0.658	0.667	0.333	0.664	0.669
736	0.650	0.617	0.633	0.267	0.629	0.639
737	0.692	0.683	0.688	0.375	0.686	0.689
738	0.625	0.633	0.629	0.258	0.630	0.628
739	0.683	0.667	0.675	0.350	0.672	0.678
740	0.683	0.650	0.667	0.334	0.661	0.672
741	0.675	0.692	0.683	0.367	0.686	0.681
742	0.700	0.692	0.696	0.392	0.694	0.697
743	0.642	0.642	0.642	0.283	0.642	0.642
744	0.700	0.633	0.667	0.334	0.656	0.677
745	0.650	0.650	0.650	0.300	0.650	0.650
746	0.683	0.692	0.688	0.375	0.689	0.686
747	0.675	0.650	0.663	0.325	0.659	0.667
748	0.683	0.642	0.663	0.325	0.656	0.669
749	0.625	0.700	0.663	0.326	0.676	0.649
750	0.725	0.658	0.692	0.384	0.680	0.702
751	0.717	0.650	0.683	0.367	0.672	0.694
752	0.675	0.617	0.646	0.292	0.638	0.656

753	0.625	0.708	0.667	0.334	0.682	0.652
754	0.658	0.633	0.646	0.292	0.642	0.650
755	0.683	0.683	0.683	0.367	0.683	0.683
756	0.692	0.650	0.671	0.342	0.664	0.678
757	0.650	0.667	0.658	0.317	0.661	0.655
758	0.717	0.633	0.675	0.351	0.662	0.688
759	0.692	0.642	0.667	0.334	0.659	0.675
760	0.658	0.625	0.642	0.283	0.637	0.648
761	0.650	0.642	0.646	0.292	0.645	0.647
762	0.675	0.692	0.683	0.367	0.686	0.681
763	0.700	0.675	0.688	0.375	0.683	0.691
764	0.633	0.692	0.663	0.326	0.673	0.652
765	0.725	0.683	0.704	0.409	0.696	0.710
766	0.675	0.608	0.642	0.284	0.633	0.653
767	0.650	0.667	0.658	0.317	0.661	0.655
768	0.692	0.675	0.683	0.367	0.680	0.686
769	0.683	0.658	0.671	0.342	0.667	0.675
770	0.700	0.650	0.675	0.350	0.667	0.683
771	0.667	0.683	0.675	0.350	0.678	0.672
772	0.658	0.658	0.658	0.317	0.658	0.658
773	0.683	0.608	0.646	0.292	0.636	0.659
774	0.708	0.658	0.683	0.367	0.675	0.691
775	0.675	0.667	0.671	0.342	0.669	0.672
776	0.658	0.700	0.679	0.359	0.687	0.672
777	0.675	0.683	0.679	0.358	0.681	0.678
778	0.692	0.667	0.679	0.358	0.675	0.683
779	0.650	0.667	0.658	0.317	0.661	0.655
780	0.675	0.650	0.663	0.325	0.659	0.667
781	0.658	0.658	0.658	0.317	0.658	0.658
782	0.683	0.658	0.671	0.342	0.667	0.675
783	0.650	0.667	0.658	0.317	0.661	0.655
784	0.658	0.650	0.654	0.308	0.653	0.656
785	0.625	0.658	0.642	0.283	0.647	0.636
786	0.683	0.683	0.683	0.367	0.683	0.683
787	0.667	0.675	0.671	0.342	0.672	0.669
788	0.692	0.683	0.688	0.375	0.686	0.689
789	0.650	0.675	0.663	0.325	0.667	0.658
790	0.692	0.675	0.683	0.367	0.680	0.686
791	0.675	0.683	0.679	0.358	0.681	0.678
792	0.725	0.608	0.667	0.336	0.649	0.685
793	0.733	0.667	0.700	0.401	0.688	0.710
794	0.683	0.658	0.671	0.342	0.667	0.675

795	0.642	0.675	0.658	0.317	0.664	0.653
796	0.683	0.650	0.667	0.334	0.661	0.672
797	0.675	0.625	0.650	0.300	0.643	0.659
798	0.683	0.683	0.683	0.367	0.683	0.683
799	0.692	0.650	0.671	0.342	0.664	0.678
800	0.700	0.642	0.671	0.342	0.661	0.680
801	0.692	0.650	0.671	0.342	0.664	0.678
802	0.692	0.675	0.683	0.367	0.680	0.686
803	0.667	0.608	0.638	0.275	0.630	0.648
804	0.708	0.658	0.683	0.367	0.675	0.691
805	0.675	0.642	0.658	0.317	0.653	0.664
806	0.658	0.633	0.646	0.292	0.642	0.650
807	0.717	0.667	0.692	0.384	0.683	0.699
808	0.675	0.700	0.688	0.375	0.692	0.684
809	0.667	0.650	0.658	0.317	0.656	0.661
810	0.675	0.650	0.663	0.325	0.659	0.667
811	0.683	0.633	0.658	0.317	0.651	0.667
812	0.700	0.617	0.658	0.318	0.646	0.672
813	0.675	0.692	0.683	0.367	0.686	0.681
814	0.667	0.600	0.633	0.267	0.625	0.645
815	0.700	0.658	0.679	0.359	0.672	0.686
816	0.658	0.692	0.675	0.350	0.681	0.669
817	0.658	0.642	0.650	0.300	0.648	0.653
818	0.658	0.683	0.671	0.342	0.675	0.667
819	0.683	0.650	0.667	0.334	0.661	0.672
820	0.683	0.650	0.667	0.334	0.661	0.672
821	0.633	0.600	0.617	0.233	0.613	0.623
822	0.675	0.667	0.671	0.342	0.669	0.672
823	0.658	0.658	0.658	0.317	0.658	0.658
824	0.658	0.700	0.679	0.359	0.687	0.672
825	0.658	0.667	0.663	0.325	0.664	0.661
826	0.675	0.617	0.646	0.292	0.638	0.656
827	0.683	0.667	0.675	0.350	0.672	0.678
828	0.625	0.683	0.654	0.309	0.664	0.644
829	0.658	0.625	0.642	0.283	0.637	0.648
830	0.667	0.608	0.638	0.275	0.630	0.648
831	0.692	0.675	0.683	0.367	0.680	0.686
832	0.667	0.667	0.667	0.333	0.667	0.667
833	0.717	0.675	0.696	0.392	0.688	0.702
834	0.667	0.625	0.646	0.292	0.640	0.653
835	0.658	0.642	0.650	0.300	0.648	0.653
836	0.667	0.658	0.663	0.325	0.661	0.664

837	0.675	0.650	0.663	0.325	0.659	0.667
838	0.692	0.667	0.679	0.358	0.675	0.683
839	0.667	0.633	0.650	0.300	0.645	0.656
840	0.658	0.667	0.663	0.325	0.664	0.661
841	0.700	0.683	0.692	0.383	0.689	0.694
842	0.658	0.625	0.642	0.283	0.637	0.648
843	0.683	0.642	0.663	0.325	0.656	0.669
844	0.683	0.667	0.675	0.350	0.672	0.678
845	0.642	0.608	0.625	0.250	0.621	0.631
846	0.667	0.642	0.654	0.308	0.650	0.658
847	0.650	0.683	0.667	0.334	0.672	0.661
848	0.675	0.650	0.663	0.325	0.659	0.667
849	0.692	0.667	0.679	0.358	0.675	0.683
850	0.675	0.675	0.675	0.350	0.675	0.675
851	0.675	0.658	0.667	0.333	0.664	0.669
852	0.700	0.633	0.667	0.334	0.656	0.677
853	0.675	0.675	0.675	0.350	0.675	0.675
854	0.700	0.650	0.675	0.350	0.667	0.683
855	0.658	0.642	0.650	0.300	0.648	0.653
856	0.642	0.658	0.650	0.300	0.653	0.647
857	0.642	0.658	0.650	0.300	0.653	0.647
858	0.667	0.692	0.679	0.358	0.684	0.675
859	0.667	0.700	0.683	0.367	0.690	0.678
860	0.717	0.667	0.692	0.384	0.683	0.699
861	0.667	0.633	0.650	0.300	0.645	0.656
862	0.675	0.633	0.654	0.309	0.648	0.661
863	0.717	0.692	0.704	0.408	0.699	0.708
864	0.683	0.683	0.683	0.367	0.683	0.683
865	0.683	0.625	0.654	0.309	0.646	0.664
866	0.683	0.642	0.663	0.325	0.656	0.669
867	0.667	0.667	0.667	0.333	0.667	0.667
868	0.658	0.700	0.679	0.359	0.687	0.672
869	0.717	0.658	0.688	0.376	0.677	0.696
870	0.667	0.625	0.646	0.292	0.640	0.653
871	0.658	0.683	0.671	0.342	0.675	0.667
872	0.667	0.675	0.671	0.342	0.672	0.669
873	0.658	0.658	0.658	0.317	0.658	0.658
874	0.692	0.625	0.658	0.317	0.648	0.669
875	0.667	0.658	0.663	0.325	0.661	0.664
876	0.692	0.642	0.667	0.334	0.659	0.675
877	0.633	0.675	0.654	0.309	0.661	0.647
878	0.692	0.658	0.675	0.350	0.669	0.680

879	0.667	0.667	0.667	0.333	0.667	0.667
880	0.667	0.667	0.667	0.333	0.667	0.667
881	0.692	0.642	0.667	0.334	0.659	0.675
882	0.717	0.625	0.671	0.343	0.656	0.685
883	0.733	0.692	0.713	0.425	0.704	0.718
884	0.675	0.675	0.675	0.350	0.675	0.675
885	0.650	0.667	0.658	0.317	0.661	0.655
886	0.708	0.683	0.696	0.392	0.691	0.700
887	0.625	0.642	0.633	0.267	0.636	0.630
888	0.642	0.675	0.658	0.317	0.664	0.653
889	0.692	0.625	0.658	0.317	0.648	0.669
890	0.650	0.667	0.658	0.317	0.661	0.655
891	0.675	0.650	0.663	0.325	0.659	0.667
892	0.667	0.667	0.667	0.333	0.667	0.667
893	0.650	0.675	0.663	0.325	0.667	0.658
894	0.675	0.675	0.675	0.350	0.675	0.675
895	0.700	0.617	0.658	0.318	0.646	0.672
896	0.675	0.675	0.675	0.350	0.675	0.675
897	0.692	0.675	0.683	0.367	0.680	0.686
898	0.692	0.658	0.675	0.350	0.669	0.680
899	0.642	0.717	0.679	0.359	0.694	0.667
900	0.683	0.633	0.658	0.317	0.651	0.667
901	0.642	0.642	0.642	0.283	0.642	0.642
902	0.667	0.642	0.654	0.308	0.650	0.658
903	0.683	0.633	0.658	0.317	0.651	0.667
904	0.692	0.650	0.671	0.342	0.664	0.678
905	0.692	0.667	0.679	0.358	0.675	0.683
906	0.700	0.700	0.700	0.400	0.700	0.700
907	0.675	0.675	0.675	0.350	0.675	0.675
908	0.633	0.658	0.646	0.292	0.650	0.641
909	0.658	0.683	0.671	0.342	0.675	0.667
910	0.650	0.675	0.663	0.325	0.667	0.658
911	0.708	0.650	0.679	0.359	0.669	0.688
912	0.683	0.642	0.663	0.325	0.656	0.669
913	0.708	0.633	0.671	0.343	0.659	0.683
914	0.717	0.633	0.675	0.351	0.662	0.688
915	0.675	0.667	0.671	0.342	0.669	0.672
916	0.675	0.650	0.663	0.325	0.659	0.667
917	0.683	0.625	0.654	0.309	0.646	0.664
918	0.658	0.650	0.654	0.308	0.653	0.656
919	0.667	0.667	0.667	0.333	0.667	0.667
920	0.675	0.642	0.658	0.317	0.653	0.664

921	0.717	0.642	0.679	0.359	0.667	0.691
922	0.708	0.650	0.679	0.359	0.669	0.688
923	0.675	0.675	0.675	0.350	0.675	0.675
924	0.667	0.667	0.667	0.333	0.667	0.667
925	0.675	0.675	0.675	0.350	0.675	0.675
926	0.642	0.667	0.654	0.308	0.658	0.650
927	0.650	0.658	0.654	0.308	0.655	0.653
928	0.708	0.675	0.692	0.384	0.685	0.697
929	0.717	0.667	0.692	0.384	0.683	0.699
930	0.708	0.633	0.671	0.343	0.659	0.683
931	0.692	0.667	0.679	0.358	0.675	0.683
932	0.692	0.642	0.667	0.334	0.659	0.675
933	0.683	0.608	0.646	0.292	0.636	0.659
934	0.675	0.675	0.675	0.350	0.675	0.675
935	0.692	0.700	0.696	0.392	0.697	0.695
936	0.658	0.658	0.658	0.317	0.658	0.658
937	0.650	0.608	0.629	0.259	0.624	0.637
938	0.692	0.642	0.667	0.334	0.659	0.675
939	0.675	0.650	0.663	0.325	0.659	0.667
940	0.675	0.658	0.667	0.333	0.664	0.669
941	0.708	0.617	0.663	0.326	0.649	0.677
942	0.667	0.675	0.671	0.342	0.672	0.669
943	0.717	0.617	0.667	0.335	0.652	0.683
944	0.675	0.658	0.667	0.333	0.664	0.669
945	0.675	0.650	0.663	0.325	0.659	0.667
946	0.717	0.608	0.663	0.327	0.647	0.680
947	0.625	0.658	0.642	0.283	0.647	0.636
948	0.675	0.650	0.663	0.325	0.659	0.667
949	0.633	0.642	0.638	0.275	0.639	0.636
950	0.650	0.633	0.642	0.283	0.639	0.645
951	0.667	0.717	0.692	0.384	0.702	0.684
952	0.658	0.600	0.629	0.259	0.622	0.640
953	0.692	0.642	0.667	0.334	0.659	0.675
954	0.625	0.617	0.621	0.242	0.620	0.622
955	0.658	0.633	0.646	0.292	0.642	0.650
956	0.692	0.667	0.679	0.358	0.675	0.683
957	0.642	0.667	0.654	0.308	0.658	0.650
958	0.642	0.642	0.642	0.283	0.642	0.642
959	0.675	0.675	0.675	0.350	0.675	0.675
960	0.667	0.617	0.642	0.284	0.635	0.650
961	0.708	0.608	0.658	0.318	0.644	0.675
962	0.667	0.700	0.683	0.367	0.690	0.678

963	0.658	0.675	0.667	0.333	0.669	0.664
964	0.675	0.650	0.663	0.325	0.659	0.667
965	0.667	0.667	0.667	0.333	0.667	0.667
966	0.667	0.633	0.650	0.300	0.645	0.656
967	0.650	0.642	0.646	0.292	0.645	0.647
968	0.675	0.658	0.667	0.333	0.664	0.669
969	0.642	0.625	0.633	0.267	0.631	0.636
970	0.667	0.650	0.658	0.317	0.656	0.661
971	0.675	0.625	0.650	0.300	0.643	0.659
972	0.675	0.608	0.642	0.284	0.633	0.653
973	0.617	0.650	0.633	0.267	0.638	0.627
974	0.683	0.675	0.679	0.358	0.678	0.680
975	0.692	0.658	0.675	0.350	0.669	0.680
976	0.692	0.642	0.667	0.334	0.659	0.675
977	0.675	0.700	0.688	0.375	0.692	0.684
978	0.642	0.642	0.642	0.283	0.642	0.642
979	0.658	0.650	0.654	0.308	0.653	0.656
980	0.667	0.667	0.667	0.333	0.667	0.667
981	0.625	0.667	0.646	0.292	0.652	0.638
982	0.717	0.650	0.683	0.367	0.672	0.694
983	0.692	0.650	0.671	0.342	0.664	0.678
984	0.667	0.667	0.667	0.333	0.667	0.667
985	0.692	0.667	0.679	0.358	0.675	0.683
986	0.700	0.667	0.683	0.367	0.677	0.689
987	0.667	0.667	0.667	0.333	0.667	0.667
988	0.692	0.625	0.658	0.317	0.648	0.669
989	0.650	0.692	0.671	0.342	0.678	0.664
990	0.642	0.650	0.646	0.292	0.647	0.644
991	0.667	0.658	0.663	0.325	0.661	0.664
992	0.658	0.675	0.667	0.333	0.669	0.664
993	0.675	0.625	0.650	0.300	0.643	0.659
994	0.658	0.658	0.658	0.317	0.658	0.658
995	0.650	0.658	0.654	0.308	0.655	0.653
996	0.692	0.658	0.675	0.350	0.669	0.680
997	0.642	0.583	0.613	0.225	0.606	0.623
998	0.717	0.650	0.683	0.367	0.672	0.694
999	0.650	0.667	0.658	0.317	0.661	0.655
1000	0.650	0.642	0.646	0.292	0.645	0.647
1001	0.658	0.625	0.642	0.283	0.637	0.648
1002	0.667	0.650	0.658	0.317	0.656	0.661
1003	0.683	0.650	0.667	0.334	0.661	0.672
1004	0.650	0.683	0.667	0.334	0.672	0.661

1005	0.658	0.675	0.667	0.333	0.669	0.664
1006	0.617	0.667	0.642	0.284	0.649	0.632
1007	0.650	0.650	0.650	0.300	0.650	0.650
1008	0.675	0.683	0.679	0.358	0.681	0.678
1009	0.650	0.692	0.671	0.342	0.678	0.664
1010	0.633	0.667	0.650	0.300	0.655	0.644
1011	0.675	0.633	0.654	0.309	0.648	0.661
1012	0.700	0.642	0.671	0.342	0.661	0.680
1013	0.667	0.650	0.658	0.317	0.656	0.661
1014	0.658	0.633	0.646	0.292	0.642	0.650
1015	0.675	0.667	0.671	0.342	0.669	0.672
1016	0.658	0.650	0.654	0.308	0.653	0.656
1017	0.683	0.683	0.683	0.367	0.683	0.683
1018	0.650	0.675	0.663	0.325	0.667	0.658
1019	0.658	0.658	0.658	0.317	0.658	0.658
1020	0.667	0.658	0.663	0.325	0.661	0.664

(2) K-nearest neighbour

Number of features	SN	SP	ACC	MCC	Precision	F1-measure
1	0.550	0.583	0.567	0.133	0.569	0.559
2	0.667	0.650	0.658	0.317	0.656	0.661
3	0.633	0.658	0.646	0.292	0.650	0.641
4	0.650	0.675	0.663	0.325	0.667	0.658
5	0.717	0.733	0.725	0.450	0.729	0.723
6	0.700	0.725	0.713	0.425	0.718	0.709
7	0.700	0.783	0.742	0.485	0.764	0.730
8	0.675	0.783	0.729	0.461	0.757	0.714
9	0.667	0.775	0.721	0.444	0.748	0.705
10	0.700	0.800	0.750	0.503	0.778	0.737
11	0.692	0.842	0.767	0.539	0.814	0.748
12	0.692	0.833	0.763	0.530	0.806	0.744
13	0.675	0.867	0.771	0.552	0.835	0.747
14	0.617	0.867	0.742	0.499	0.822	0.705
15	0.650	0.850	0.750	0.510	0.813	0.722
16	0.625	0.842	0.733	0.478	0.798	0.701
17	0.625	0.833	0.729	0.469	0.789	0.698
18	0.708	0.842	0.775	0.555	0.817	0.759
19	0.683	0.842	0.763	0.532	0.812	0.742
20	0.675	0.867	0.771	0.552	0.835	0.747
21	0.683	0.858	0.771	0.550	0.828	0.749
22	0.683	0.875	0.779	0.569	0.845	0.756
23	0.675	0.867	0.771	0.552	0.835	0.747

24	0.700	0.883	0.792	0.593	0.857	0.771
25	0.725	0.875	0.800	0.607	0.853	0.784
26	0.708	0.892	0.800	0.610	0.867	0.780
27	0.692	0.875	0.783	0.576	0.847	0.761
28	0.717	0.892	0.804	0.618	0.869	0.785
29	0.725	0.875	0.800	0.607	0.853	0.784
30	0.675	0.875	0.775	0.561	0.844	0.750
31	0.675	0.900	0.788	0.590	0.871	0.761
32	0.692	0.867	0.779	0.567	0.838	0.758
33	0.700	0.825	0.763	0.529	0.800	0.747
34	0.717	0.850	0.783	0.572	0.827	0.768
35	0.717	0.825	0.771	0.545	0.804	0.758
36	0.700	0.842	0.771	0.547	0.816	0.753
37	0.708	0.842	0.775	0.555	0.817	0.759
38	0.683	0.850	0.767	0.541	0.820	0.745
39	0.675	0.858	0.767	0.543	0.827	0.743
40	0.683	0.850	0.767	0.541	0.820	0.745
41	0.667	0.892	0.779	0.573	0.860	0.751
42	0.633	0.908	0.771	0.563	0.874	0.734
43	0.658	0.900	0.779	0.575	0.868	0.749
44	0.650	0.892	0.771	0.558	0.857	0.739
45	0.650	0.892	0.771	0.558	0.857	0.739
46	0.650	0.892	0.771	0.558	0.857	0.739
47	0.650	0.900	0.775	0.568	0.867	0.743
48	0.658	0.908	0.783	0.585	0.878	0.752
49	0.658	0.925	0.792	0.605	0.898	0.760
50	0.692	0.883	0.788	0.586	0.856	0.765
51	0.650	0.917	0.783	0.588	0.886	0.750
52	0.675	0.900	0.788	0.590	0.871	0.761
53	0.658	0.917	0.788	0.595	0.888	0.756
54	0.675	0.917	0.796	0.610	0.890	0.768
55	0.667	0.900	0.783	0.583	0.870	0.755
56	0.675	0.925	0.800	0.620	0.900	0.771
57	0.650	0.925	0.788	0.598	0.897	0.754
58	0.650	0.908	0.779	0.578	0.876	0.746
59	0.650	0.900	0.775	0.568	0.867	0.743
60	0.642	0.900	0.771	0.561	0.865	0.737
61	0.642	0.917	0.779	0.581	0.885	0.744
62	0.633	0.917	0.775	0.574	0.884	0.738
63	0.633	0.892	0.763	0.543	0.854	0.727
64	0.608	0.900	0.754	0.531	0.859	0.712
65	0.625	0.908	0.767	0.556	0.872	0.728

66	0.617	0.925	0.771	0.569	0.892	0.729
67	0.617	0.933	0.775	0.580	0.902	0.733
68	0.625	0.933	0.779	0.587	0.904	0.739
69	0.633	0.917	0.775	0.574	0.884	0.738
70	0.675	0.917	0.796	0.610	0.890	0.768
71	0.683	0.917	0.800	0.617	0.891	0.774
72	0.675	0.925	0.800	0.620	0.900	0.771
73	0.692	0.908	0.800	0.615	0.883	0.776
74	0.683	0.917	0.800	0.617	0.891	0.774
75	0.708	0.900	0.804	0.620	0.876	0.783
76	0.683	0.900	0.792	0.598	0.872	0.766
77	0.692	0.900	0.796	0.605	0.874	0.772
78	0.675	0.917	0.796	0.610	0.890	0.768
79	0.675	0.917	0.796	0.610	0.890	0.768
80	0.667	0.925	0.796	0.612	0.899	0.766
81	0.633	0.933	0.783	0.594	0.905	0.745
82	0.633	0.942	0.788	0.604	0.916	0.749
83	0.658	0.942	0.800	0.626	0.919	0.767
84	0.683	0.925	0.804	0.627	0.901	0.777
85	0.683	0.917	0.800	0.617	0.891	0.774
86	0.667	0.908	0.788	0.593	0.879	0.758
87	0.683	0.917	0.800	0.617	0.891	0.774
88	0.658	0.925	0.792	0.605	0.898	0.760
89	0.658	0.917	0.788	0.595	0.888	0.756
90	0.675	0.900	0.788	0.590	0.871	0.761
91	0.675	0.908	0.792	0.600	0.880	0.764
92	0.650	0.900	0.775	0.568	0.867	0.743
93	0.650	0.900	0.775	0.568	0.867	0.743
94	0.650	0.875	0.763	0.539	0.839	0.732
95	0.608	0.883	0.746	0.511	0.839	0.705
96	0.617	0.900	0.758	0.539	0.860	0.718
97	0.608	0.892	0.750	0.521	0.849	0.709
98	0.625	0.883	0.754	0.526	0.843	0.718
99	0.625	0.883	0.754	0.526	0.843	0.718
100	0.617	0.883	0.750	0.519	0.841	0.712
101	0.608	0.892	0.750	0.521	0.849	0.709
102	0.608	0.883	0.746	0.511	0.839	0.705
103	0.625	0.883	0.754	0.526	0.843	0.718
104	0.592	0.892	0.742	0.507	0.845	0.696
105	0.592	0.892	0.742	0.507	0.845	0.696
106	0.575	0.900	0.738	0.502	0.852	0.687
107	0.583	0.883	0.733	0.489	0.833	0.686

108	0.583	0.908	0.746	0.520	0.864	0.697
109	0.608	0.908	0.758	0.542	0.869	0.716
110	0.625	0.900	0.763	0.546	0.862	0.725
111	0.608	0.892	0.750	0.521	0.849	0.709
112	0.617	0.900	0.758	0.539	0.860	0.718
113	0.617	0.900	0.758	0.539	0.860	0.718
114	0.617	0.883	0.750	0.519	0.841	0.712
115	0.608	0.875	0.742	0.501	0.830	0.702
116	0.592	0.875	0.733	0.487	0.826	0.689
117	0.625	0.875	0.750	0.516	0.833	0.714
118	0.633	0.883	0.758	0.534	0.844	0.724
119	0.608	0.867	0.738	0.492	0.820	0.699
120	0.625	0.867	0.746	0.507	0.824	0.711
121	0.600	0.858	0.729	0.474	0.809	0.689
122	0.608	0.875	0.742	0.501	0.830	0.702
123	0.617	0.858	0.738	0.490	0.813	0.701
124	0.633	0.867	0.750	0.514	0.826	0.717
125	0.633	0.883	0.758	0.534	0.844	0.724
126	0.633	0.892	0.763	0.543	0.854	0.727
127	0.633	0.875	0.754	0.524	0.835	0.720
128	0.617	0.883	0.750	0.519	0.841	0.712
129	0.600	0.867	0.733	0.484	0.818	0.692
130	0.642	0.883	0.763	0.541	0.846	0.730
131	0.633	0.867	0.750	0.514	0.826	0.717
132	0.625	0.875	0.750	0.516	0.833	0.714
133	0.625	0.883	0.754	0.526	0.843	0.718
134	0.625	0.875	0.750	0.516	0.833	0.714
135	0.633	0.883	0.758	0.534	0.844	0.724
136	0.642	0.883	0.763	0.541	0.846	0.730
137	0.633	0.867	0.750	0.514	0.826	0.717
138	0.642	0.875	0.758	0.531	0.837	0.726
139	0.633	0.875	0.754	0.524	0.835	0.720
140	0.633	0.858	0.746	0.505	0.817	0.714
141	0.633	0.850	0.742	0.495	0.809	0.710
142	0.642	0.842	0.742	0.493	0.802	0.713
143	0.633	0.842	0.738	0.486	0.800	0.707
144	0.633	0.842	0.738	0.486	0.800	0.707
145	0.625	0.875	0.750	0.516	0.833	0.714
146	0.625	0.867	0.746	0.507	0.824	0.711
147	0.625	0.867	0.746	0.507	0.824	0.711
148	0.625	0.867	0.746	0.507	0.824	0.711
149	0.633	0.867	0.750	0.514	0.826	0.717

150	0.633	0.875	0.754	0.524	0.835	0.720
151	0.625	0.875	0.750	0.516	0.833	0.714
152	0.642	0.867	0.754	0.522	0.828	0.723
153	0.650	0.867	0.758	0.529	0.830	0.729
154	0.642	0.850	0.746	0.503	0.811	0.716
155	0.642	0.850	0.746	0.503	0.811	0.716
156	0.658	0.850	0.754	0.518	0.814	0.728
157	0.658	0.875	0.767	0.546	0.840	0.738
158	0.650	0.875	0.763	0.539	0.839	0.732
159	0.658	0.858	0.758	0.527	0.823	0.731
160	0.658	0.850	0.754	0.518	0.814	0.728
161	0.650	0.850	0.750	0.510	0.813	0.722
162	0.650	0.850	0.750	0.510	0.813	0.722
163	0.650	0.850	0.750	0.510	0.813	0.722
164	0.658	0.850	0.754	0.518	0.814	0.728
165	0.658	0.850	0.754	0.518	0.814	0.728
166	0.650	0.867	0.758	0.529	0.830	0.729
167	0.658	0.850	0.754	0.518	0.814	0.728
168	0.633	0.850	0.742	0.495	0.809	0.710
169	0.633	0.867	0.750	0.514	0.826	0.717
170	0.642	0.850	0.746	0.503	0.811	0.716
171	0.642	0.858	0.750	0.512	0.819	0.720
172	0.650	0.867	0.758	0.529	0.830	0.729
173	0.667	0.858	0.763	0.535	0.825	0.737
174	0.692	0.867	0.779	0.567	0.838	0.758
175	0.675	0.867	0.771	0.552	0.835	0.747
176	0.675	0.867	0.771	0.552	0.835	0.747
177	0.675	0.875	0.775	0.561	0.844	0.750
178	0.658	0.867	0.763	0.537	0.832	0.735
179	0.667	0.900	0.783	0.583	0.870	0.755
180	0.667	0.875	0.771	0.554	0.842	0.744
181	0.667	0.883	0.775	0.563	0.851	0.748
182	0.667	0.883	0.775	0.563	0.851	0.748
183	0.675	0.875	0.775	0.561	0.844	0.750
184	0.658	0.867	0.763	0.537	0.832	0.735
185	0.675	0.867	0.771	0.552	0.835	0.747
186	0.667	0.875	0.771	0.554	0.842	0.744
187	0.650	0.858	0.754	0.520	0.821	0.726
188	0.633	0.867	0.750	0.514	0.826	0.717
189	0.633	0.858	0.746	0.505	0.817	0.714
190	0.633	0.867	0.750	0.514	0.826	0.717
191	0.625	0.867	0.746	0.507	0.824	0.711

192	0.617	0.867	0.742	0.499	0.822	0.705
193	0.617	0.867	0.742	0.499	0.822	0.705
194	0.625	0.875	0.750	0.516	0.833	0.714
195	0.633	0.867	0.750	0.514	0.826	0.717
196	0.625	0.883	0.754	0.526	0.843	0.718
197	0.625	0.883	0.754	0.526	0.843	0.718
198	0.633	0.875	0.754	0.524	0.835	0.720
199	0.650	0.867	0.758	0.529	0.830	0.729
200	0.650	0.867	0.758	0.529	0.830	0.729
201	0.625	0.875	0.750	0.516	0.833	0.714
202	0.633	0.875	0.754	0.524	0.835	0.720
203	0.633	0.875	0.754	0.524	0.835	0.720
204	0.633	0.875	0.754	0.524	0.835	0.720
205	0.642	0.875	0.758	0.531	0.837	0.726
206	0.642	0.875	0.758	0.531	0.837	0.726
207	0.650	0.875	0.763	0.539	0.839	0.732
208	0.625	0.875	0.750	0.516	0.833	0.714
209	0.625	0.867	0.746	0.507	0.824	0.711
210	0.642	0.883	0.763	0.541	0.846	0.730
211	0.642	0.883	0.763	0.541	0.846	0.730
212	0.650	0.900	0.775	0.568	0.867	0.743
213	0.650	0.900	0.775	0.568	0.867	0.743
214	0.642	0.908	0.775	0.571	0.875	0.740
215	0.658	0.900	0.779	0.575	0.868	0.749
216	0.658	0.908	0.783	0.585	0.878	0.752
217	0.667	0.883	0.775	0.563	0.851	0.748
218	0.658	0.883	0.771	0.556	0.849	0.742
219	0.658	0.875	0.767	0.546	0.840	0.738
220	0.667	0.883	0.775	0.563	0.851	0.748
221	0.667	0.883	0.775	0.563	0.851	0.748
222	0.658	0.883	0.771	0.556	0.849	0.742
223	0.650	0.883	0.767	0.548	0.848	0.736
224	0.667	0.892	0.779	0.573	0.860	0.751
225	0.658	0.900	0.779	0.575	0.868	0.749
226	0.667	0.900	0.783	0.583	0.870	0.755
227	0.658	0.900	0.779	0.575	0.868	0.749
228	0.658	0.883	0.771	0.556	0.849	0.742
229	0.650	0.892	0.771	0.558	0.857	0.739
230	0.650	0.892	0.771	0.558	0.857	0.739
231	0.658	0.892	0.775	0.566	0.859	0.745
232	0.658	0.900	0.779	0.575	0.868	0.749
233	0.658	0.900	0.779	0.575	0.868	0.749

234	0.667	0.900	0.783	0.583	0.870	0.755
235	0.667	0.900	0.783	0.583	0.870	0.755
236	0.667	0.900	0.783	0.583	0.870	0.755
237	0.658	0.908	0.783	0.585	0.878	0.752
238	0.650	0.908	0.779	0.578	0.876	0.746
239	0.642	0.908	0.775	0.571	0.875	0.740
240	0.650	0.908	0.779	0.578	0.876	0.746
241	0.642	0.900	0.771	0.561	0.865	0.737
242	0.658	0.900	0.779	0.575	0.868	0.749
243	0.633	0.900	0.767	0.553	0.864	0.731
244	0.642	0.900	0.771	0.561	0.865	0.737
245	0.642	0.917	0.779	0.581	0.885	0.744
246	0.658	0.917	0.788	0.595	0.888	0.756
247	0.650	0.917	0.783	0.588	0.886	0.750
248	0.667	0.900	0.783	0.583	0.870	0.755
249	0.650	0.892	0.771	0.558	0.857	0.739
250	0.642	0.917	0.779	0.581	0.885	0.744
251	0.642	0.925	0.783	0.591	0.895	0.748
252	0.650	0.908	0.779	0.578	0.876	0.746
253	0.633	0.917	0.775	0.574	0.884	0.738
254	0.642	0.917	0.779	0.581	0.885	0.744
255	0.642	0.917	0.779	0.581	0.885	0.744
256	0.650	0.917	0.783	0.588	0.886	0.750
257	0.650	0.917	0.783	0.588	0.886	0.750
258	0.650	0.917	0.783	0.588	0.886	0.750
259	0.650	0.917	0.783	0.588	0.886	0.750
260	0.658	0.917	0.788	0.595	0.888	0.756
261	0.650	0.917	0.783	0.588	0.886	0.750
262	0.650	0.917	0.783	0.588	0.886	0.750
263	0.642	0.908	0.775	0.571	0.875	0.740
264	0.642	0.908	0.775	0.571	0.875	0.740
265	0.650	0.917	0.783	0.588	0.886	0.750
266	0.650	0.925	0.788	0.598	0.897	0.754
267	0.650	0.925	0.788	0.598	0.897	0.754
268	0.650	0.933	0.792	0.608	0.907	0.757
269	0.658	0.917	0.788	0.595	0.888	0.756
270	0.667	0.900	0.783	0.583	0.870	0.755
271	0.675	0.917	0.796	0.610	0.890	0.768
272	0.667	0.925	0.796	0.612	0.899	0.766
273	0.667	0.925	0.796	0.612	0.899	0.766
274	0.675	0.925	0.800	0.620	0.900	0.771
275	0.675	0.917	0.796	0.610	0.890	0.768

276	0.658	0.908	0.783	0.585	0.878	0.752
277	0.650	0.917	0.783	0.588	0.886	0.750
278	0.658	0.908	0.783	0.585	0.878	0.752
279	0.650	0.908	0.779	0.578	0.876	0.746
280	0.642	0.908	0.775	0.571	0.875	0.740
281	0.642	0.908	0.775	0.571	0.875	0.740
282	0.650	0.908	0.779	0.578	0.876	0.746
283	0.650	0.908	0.779	0.578	0.876	0.746
284	0.658	0.908	0.783	0.585	0.878	0.752
285	0.658	0.900	0.779	0.575	0.868	0.749
286	0.650	0.900	0.775	0.568	0.867	0.743
287	0.650	0.908	0.779	0.578	0.876	0.746
288	0.633	0.908	0.771	0.563	0.874	0.734
289	0.633	0.908	0.771	0.563	0.874	0.734
290	0.667	0.908	0.788	0.593	0.879	0.758
291	0.658	0.917	0.788	0.595	0.888	0.756
292	0.650	0.900	0.775	0.568	0.867	0.743
293	0.633	0.900	0.767	0.553	0.864	0.731
294	0.633	0.892	0.763	0.543	0.854	0.727
295	0.633	0.892	0.763	0.543	0.854	0.727
296	0.633	0.900	0.767	0.553	0.864	0.731
297	0.625	0.900	0.763	0.546	0.862	0.725
298	0.633	0.900	0.767	0.553	0.864	0.731
299	0.633	0.900	0.767	0.553	0.864	0.731
300	0.633	0.908	0.771	0.563	0.874	0.734
301	0.633	0.908	0.771	0.563	0.874	0.734
302	0.650	0.908	0.779	0.578	0.876	0.746
303	0.658	0.917	0.788	0.595	0.888	0.756
304	0.642	0.917	0.779	0.581	0.885	0.744
305	0.642	0.917	0.779	0.581	0.885	0.744
306	0.642	0.917	0.779	0.581	0.885	0.744
307	0.642	0.917	0.779	0.581	0.885	0.744
308	0.633	0.908	0.771	0.563	0.874	0.734
309	0.633	0.908	0.771	0.563	0.874	0.734
310	0.658	0.908	0.783	0.585	0.878	0.752
311	0.650	0.908	0.779	0.578	0.876	0.746
312	0.642	0.900	0.771	0.561	0.865	0.737
313	0.667	0.900	0.783	0.583	0.870	0.755
314	0.633	0.900	0.767	0.553	0.864	0.731
315	0.650	0.908	0.779	0.578	0.876	0.746
316	0.667	0.908	0.788	0.593	0.879	0.758
317	0.675	0.900	0.788	0.590	0.871	0.761

318	0.658	0.900	0.779	0.575	0.868	0.749
319	0.658	0.900	0.779	0.575	0.868	0.749
320	0.658	0.900	0.779	0.575	0.868	0.749
321	0.650	0.900	0.775	0.568	0.867	0.743
322	0.650	0.900	0.775	0.568	0.867	0.743
323	0.667	0.900	0.783	0.583	0.870	0.755
324	0.650	0.908	0.779	0.578	0.876	0.746
325	0.642	0.900	0.771	0.561	0.865	0.737
326	0.642	0.900	0.771	0.561	0.865	0.737
327	0.642	0.900	0.771	0.561	0.865	0.737
328	0.650	0.908	0.779	0.578	0.876	0.746
329	0.658	0.908	0.783	0.585	0.878	0.752
330	0.675	0.900	0.788	0.590	0.871	0.761
331	0.667	0.900	0.783	0.583	0.870	0.755
332	0.675	0.900	0.788	0.590	0.871	0.761
333	0.692	0.900	0.796	0.605	0.874	0.772
334	0.692	0.900	0.796	0.605	0.874	0.772
335	0.700	0.900	0.800	0.612	0.875	0.778
336	0.692	0.892	0.792	0.595	0.865	0.769
337	0.700	0.892	0.796	0.603	0.866	0.774
338	0.692	0.900	0.796	0.605	0.874	0.772
339	0.700	0.892	0.796	0.603	0.866	0.774
340	0.700	0.892	0.796	0.603	0.866	0.774
341	0.683	0.892	0.788	0.588	0.863	0.763
342	0.683	0.892	0.788	0.588	0.863	0.763
343	0.683	0.892	0.788	0.588	0.863	0.763
344	0.683	0.892	0.788	0.588	0.863	0.763
345	0.683	0.892	0.788	0.588	0.863	0.763
346	0.675	0.892	0.783	0.580	0.862	0.757
347	0.683	0.892	0.788	0.588	0.863	0.763
348	0.683	0.892	0.788	0.588	0.863	0.763
349	0.683	0.892	0.788	0.588	0.863	0.763
350	0.692	0.892	0.792	0.595	0.865	0.769
351	0.683	0.892	0.788	0.588	0.863	0.763
352	0.667	0.892	0.779	0.573	0.860	0.751
353	0.667	0.892	0.779	0.573	0.860	0.751
354	0.667	0.892	0.779	0.573	0.860	0.751
355	0.658	0.900	0.779	0.575	0.868	0.749
356	0.650	0.908	0.779	0.578	0.876	0.746
357	0.667	0.900	0.783	0.583	0.870	0.755
358	0.675	0.900	0.788	0.590	0.871	0.761
359	0.675	0.900	0.788	0.590	0.871	0.761

360	0.675	0.900	0.788	0.590	0.871	0.761
361	0.667	0.900	0.783	0.583	0.870	0.755
362	0.667	0.892	0.779	0.573	0.860	0.751
363	0.667	0.883	0.775	0.563	0.851	0.748
364	0.667	0.892	0.779	0.573	0.860	0.751
365	0.658	0.892	0.775	0.566	0.859	0.745
366	0.658	0.883	0.771	0.556	0.849	0.742
367	0.650	0.883	0.767	0.548	0.848	0.736
368	0.650	0.892	0.771	0.558	0.857	0.739
369	0.658	0.900	0.779	0.575	0.868	0.749
370	0.667	0.900	0.783	0.583	0.870	0.755
371	0.675	0.875	0.775	0.561	0.844	0.750
372	0.675	0.867	0.771	0.552	0.835	0.747
373	0.683	0.867	0.775	0.559	0.837	0.752
374	0.683	0.883	0.783	0.578	0.854	0.759
375	0.683	0.867	0.775	0.559	0.837	0.752
376	0.683	0.850	0.767	0.541	0.820	0.745
377	0.683	0.858	0.771	0.550	0.828	0.749
378	0.683	0.858	0.771	0.550	0.828	0.749
379	0.683	0.858	0.771	0.550	0.828	0.749
380	0.675	0.867	0.771	0.552	0.835	0.747
381	0.667	0.867	0.767	0.544	0.833	0.741
382	0.650	0.867	0.758	0.529	0.830	0.729
383	0.642	0.858	0.750	0.512	0.819	0.720
384	0.650	0.867	0.758	0.529	0.830	0.729
385	0.642	0.867	0.754	0.522	0.828	0.723
386	0.658	0.875	0.767	0.546	0.840	0.738
387	0.658	0.867	0.763	0.537	0.832	0.735
388	0.642	0.875	0.758	0.531	0.837	0.726
389	0.608	0.875	0.742	0.501	0.830	0.702
390	0.650	0.875	0.763	0.539	0.839	0.732
391	0.650	0.875	0.763	0.539	0.839	0.732
392	0.642	0.867	0.754	0.522	0.828	0.723
393	0.633	0.867	0.750	0.514	0.826	0.717
394	0.633	0.875	0.754	0.524	0.835	0.720
395	0.642	0.875	0.758	0.531	0.837	0.726
396	0.642	0.883	0.763	0.541	0.846	0.730
397	0.633	0.883	0.758	0.534	0.844	0.724
398	0.642	0.892	0.767	0.551	0.856	0.733
399	0.650	0.892	0.771	0.558	0.857	0.739
400	0.650	0.892	0.771	0.558	0.857	0.739
401	0.650	0.900	0.775	0.568	0.867	0.743

402	0.658	0.900	0.779	0.575	0.868	0.749
403	0.658	0.900	0.779	0.575	0.868	0.749
404	0.650	0.892	0.771	0.558	0.857	0.739
405	0.650	0.892	0.771	0.558	0.857	0.739
406	0.658	0.892	0.775	0.566	0.859	0.745
407	0.667	0.892	0.779	0.573	0.860	0.751
408	0.667	0.892	0.779	0.573	0.860	0.751
409	0.658	0.900	0.779	0.575	0.868	0.749
410	0.667	0.900	0.783	0.583	0.870	0.755
411	0.692	0.900	0.796	0.605	0.874	0.772
412	0.700	0.900	0.800	0.612	0.875	0.778
413	0.692	0.900	0.796	0.605	0.874	0.772
414	0.692	0.892	0.792	0.595	0.865	0.769
415	0.692	0.883	0.788	0.586	0.856	0.765
416	0.683	0.892	0.788	0.588	0.863	0.763
417	0.683	0.883	0.783	0.578	0.854	0.759
418	0.675	0.892	0.783	0.580	0.862	0.757
419	0.658	0.883	0.771	0.556	0.849	0.742
420	0.667	0.900	0.783	0.583	0.870	0.755
421	0.667	0.900	0.783	0.583	0.870	0.755
422	0.667	0.900	0.783	0.583	0.870	0.755
423	0.642	0.900	0.771	0.561	0.865	0.737
424	0.658	0.883	0.771	0.556	0.849	0.742
425	0.650	0.883	0.767	0.548	0.848	0.736
426	0.658	0.883	0.771	0.556	0.849	0.742
427	0.658	0.883	0.771	0.556	0.849	0.742
428	0.658	0.883	0.771	0.556	0.849	0.742
429	0.658	0.883	0.771	0.556	0.849	0.742
430	0.650	0.883	0.767	0.548	0.848	0.736
431	0.650	0.892	0.771	0.558	0.857	0.739
432	0.650	0.892	0.771	0.558	0.857	0.739
433	0.650	0.892	0.771	0.558	0.857	0.739
434	0.650	0.883	0.767	0.548	0.848	0.736
435	0.650	0.883	0.767	0.548	0.848	0.736
436	0.650	0.883	0.767	0.548	0.848	0.736
437	0.658	0.883	0.771	0.556	0.849	0.742
438	0.658	0.883	0.771	0.556	0.849	0.742
439	0.650	0.883	0.767	0.548	0.848	0.736
440	0.650	0.883	0.767	0.548	0.848	0.736
441	0.642	0.883	0.763	0.541	0.846	0.730
442	0.658	0.892	0.775	0.566	0.859	0.745
443	0.650	0.892	0.771	0.558	0.857	0.739

444	0.650	0.892	0.771	0.558	0.857	0.739
445	0.650	0.892	0.771	0.558	0.857	0.739
446	0.650	0.883	0.767	0.548	0.848	0.736
447	0.650	0.883	0.767	0.548	0.848	0.736
448	0.650	0.883	0.767	0.548	0.848	0.736
449	0.650	0.883	0.767	0.548	0.848	0.736
450	0.633	0.908	0.771	0.563	0.874	0.734
451	0.642	0.908	0.775	0.571	0.875	0.740
452	0.642	0.908	0.775	0.571	0.875	0.740
453	0.650	0.908	0.779	0.578	0.876	0.746
454	0.642	0.908	0.775	0.571	0.875	0.740
455	0.642	0.908	0.775	0.571	0.875	0.740
456	0.633	0.900	0.767	0.553	0.864	0.731
457	0.633	0.900	0.767	0.553	0.864	0.731
458	0.625	0.900	0.763	0.546	0.862	0.725
459	0.625	0.900	0.763	0.546	0.862	0.725
460	0.625	0.900	0.763	0.546	0.862	0.725
461	0.625	0.900	0.763	0.546	0.862	0.725
462	0.625	0.908	0.767	0.556	0.872	0.728
463	0.625	0.908	0.767	0.556	0.872	0.728
464	0.633	0.908	0.771	0.563	0.874	0.734
465	0.625	0.917	0.771	0.566	0.882	0.732
466	0.633	0.917	0.775	0.574	0.884	0.738
467	0.633	0.925	0.779	0.584	0.894	0.741
468	0.650	0.925	0.788	0.598	0.897	0.754
469	0.625	0.925	0.775	0.577	0.893	0.735
470	0.633	0.917	0.775	0.574	0.884	0.738
471	0.642	0.917	0.779	0.581	0.885	0.744
472	0.633	0.925	0.779	0.584	0.894	0.741
473	0.633	0.925	0.779	0.584	0.894	0.741
474	0.642	0.908	0.775	0.571	0.875	0.740
475	0.642	0.908	0.775	0.571	0.875	0.740
476	0.642	0.900	0.771	0.561	0.865	0.737
477	0.642	0.900	0.771	0.561	0.865	0.737
478	0.642	0.908	0.775	0.571	0.875	0.740
479	0.642	0.908	0.775	0.571	0.875	0.740
480	0.642	0.917	0.779	0.581	0.885	0.744
481	0.642	0.917	0.779	0.581	0.885	0.744
482	0.642	0.908	0.775	0.571	0.875	0.740
483	0.642	0.917	0.779	0.581	0.885	0.744
484	0.642	0.917	0.779	0.581	0.885	0.744
485	0.642	0.917	0.779	0.581	0.885	0.744

486	0.650	0.908	0.779	0.578	0.876	0.746
487	0.642	0.908	0.775	0.571	0.875	0.740
488	0.658	0.908	0.783	0.585	0.878	0.752
489	0.658	0.908	0.783	0.585	0.878	0.752
490	0.658	0.908	0.783	0.585	0.878	0.752
491	0.642	0.908	0.775	0.571	0.875	0.740
492	0.667	0.908	0.788	0.593	0.879	0.758
493	0.667	0.908	0.788	0.593	0.879	0.758
494	0.658	0.908	0.783	0.585	0.878	0.752
495	0.658	0.908	0.783	0.585	0.878	0.752
496	0.658	0.908	0.783	0.585	0.878	0.752
497	0.658	0.908	0.783	0.585	0.878	0.752
498	0.658	0.908	0.783	0.585	0.878	0.752
499	0.642	0.908	0.775	0.571	0.875	0.740
500	0.658	0.900	0.779	0.575	0.868	0.749
501	0.658	0.908	0.783	0.585	0.878	0.752
502	0.650	0.900	0.775	0.568	0.867	0.743
503	0.642	0.900	0.771	0.561	0.865	0.737
504	0.642	0.900	0.771	0.561	0.865	0.737
505	0.625	0.908	0.767	0.556	0.872	0.728
506	0.625	0.900	0.763	0.546	0.862	0.725
507	0.633	0.917	0.775	0.574	0.884	0.738
508	0.625	0.917	0.771	0.566	0.882	0.732
509	0.633	0.917	0.775	0.574	0.884	0.738
510	0.633	0.917	0.775	0.574	0.884	0.738
511	0.642	0.917	0.779	0.581	0.885	0.744
512	0.642	0.900	0.771	0.561	0.865	0.737
513	0.642	0.892	0.767	0.551	0.856	0.733
514	0.625	0.900	0.763	0.546	0.862	0.725
515	0.617	0.908	0.763	0.549	0.871	0.722
516	0.617	0.908	0.763	0.549	0.871	0.722
517	0.617	0.908	0.763	0.549	0.871	0.722
518	0.617	0.900	0.758	0.539	0.860	0.718
519	0.625	0.892	0.758	0.536	0.852	0.721
520	0.642	0.900	0.771	0.561	0.865	0.737
521	0.633	0.900	0.767	0.553	0.864	0.731
522	0.658	0.900	0.779	0.575	0.868	0.749
523	0.658	0.875	0.767	0.546	0.840	0.738
524	0.650	0.883	0.767	0.548	0.848	0.736
525	0.658	0.883	0.771	0.556	0.849	0.742
526	0.642	0.883	0.763	0.541	0.846	0.730
527	0.642	0.875	0.758	0.531	0.837	0.726

528	0.642	0.875	0.758	0.531	0.837	0.726
529	0.642	0.875	0.758	0.531	0.837	0.726
530	0.658	0.883	0.771	0.556	0.849	0.742
531	0.658	0.883	0.771	0.556	0.849	0.742
532	0.658	0.892	0.775	0.566	0.859	0.745
533	0.658	0.908	0.783	0.585	0.878	0.752
534	0.642	0.883	0.763	0.541	0.846	0.730
535	0.650	0.892	0.771	0.558	0.857	0.739
536	0.658	0.892	0.775	0.566	0.859	0.745
537	0.650	0.892	0.771	0.558	0.857	0.739
538	0.650	0.892	0.771	0.558	0.857	0.739
539	0.633	0.883	0.758	0.534	0.844	0.724
540	0.658	0.892	0.775	0.566	0.859	0.745
541	0.658	0.892	0.775	0.566	0.859	0.745
542	0.658	0.892	0.775	0.566	0.859	0.745
543	0.658	0.900	0.779	0.575	0.868	0.749
544	0.658	0.908	0.783	0.585	0.878	0.752
545	0.658	0.900	0.779	0.575	0.868	0.749
546	0.667	0.900	0.783	0.583	0.870	0.755
547	0.667	0.892	0.779	0.573	0.860	0.751
548	0.667	0.883	0.775	0.563	0.851	0.748
549	0.675	0.900	0.788	0.590	0.871	0.761
550	0.667	0.908	0.788	0.593	0.879	0.758
551	0.667	0.900	0.783	0.583	0.870	0.755
552	0.667	0.892	0.779	0.573	0.860	0.751
553	0.667	0.892	0.779	0.573	0.860	0.751
554	0.658	0.900	0.779	0.575	0.868	0.749
555	0.658	0.892	0.775	0.566	0.859	0.745
556	0.650	0.892	0.771	0.558	0.857	0.739
557	0.650	0.900	0.775	0.568	0.867	0.743
558	0.642	0.892	0.767	0.551	0.856	0.733
559	0.650	0.892	0.771	0.558	0.857	0.739
560	0.650	0.900	0.775	0.568	0.867	0.743
561	0.650	0.908	0.779	0.578	0.876	0.746
562	0.650	0.908	0.779	0.578	0.876	0.746
563	0.658	0.900	0.779	0.575	0.868	0.749
564	0.650	0.892	0.771	0.558	0.857	0.739
565	0.658	0.892	0.775	0.566	0.859	0.745
566	0.658	0.883	0.771	0.556	0.849	0.742
567	0.658	0.883	0.771	0.556	0.849	0.742
568	0.658	0.883	0.771	0.556	0.849	0.742
569	0.650	0.892	0.771	0.558	0.857	0.739

570	0.658	0.892	0.775	0.566	0.859	0.745
571	0.650	0.892	0.771	0.558	0.857	0.739
572	0.667	0.892	0.779	0.573	0.860	0.751
573	0.658	0.892	0.775	0.566	0.859	0.745
574	0.667	0.892	0.779	0.573	0.860	0.751
575	0.658	0.900	0.779	0.575	0.868	0.749
576	0.658	0.900	0.779	0.575	0.868	0.749
577	0.667	0.900	0.783	0.583	0.870	0.755
578	0.667	0.900	0.783	0.583	0.870	0.755
579	0.667	0.900	0.783	0.583	0.870	0.755
580	0.650	0.908	0.779	0.578	0.876	0.746
581	0.658	0.900	0.779	0.575	0.868	0.749
582	0.650	0.900	0.775	0.568	0.867	0.743
583	0.650	0.900	0.775	0.568	0.867	0.743
584	0.650	0.900	0.775	0.568	0.867	0.743
585	0.650	0.900	0.775	0.568	0.867	0.743
586	0.658	0.900	0.779	0.575	0.868	0.749
587	0.650	0.900	0.775	0.568	0.867	0.743
588	0.650	0.892	0.771	0.558	0.857	0.739
589	0.642	0.892	0.767	0.551	0.856	0.733
590	0.633	0.883	0.758	0.534	0.844	0.724
591	0.633	0.883	0.758	0.534	0.844	0.724
592	0.642	0.892	0.767	0.551	0.856	0.733
593	0.650	0.892	0.771	0.558	0.857	0.739
594	0.650	0.892	0.771	0.558	0.857	0.739
595	0.650	0.900	0.775	0.568	0.867	0.743
596	0.650	0.900	0.775	0.568	0.867	0.743
597	0.650	0.900	0.775	0.568	0.867	0.743
598	0.650	0.908	0.779	0.578	0.876	0.746
599	0.650	0.908	0.779	0.578	0.876	0.746
600	0.642	0.900	0.771	0.561	0.865	0.737
601	0.642	0.892	0.767	0.551	0.856	0.733
602	0.650	0.900	0.775	0.568	0.867	0.743
603	0.642	0.900	0.771	0.561	0.865	0.737
604	0.617	0.883	0.750	0.519	0.841	0.712
605	0.617	0.883	0.750	0.519	0.841	0.712
606	0.625	0.883	0.754	0.526	0.843	0.718
607	0.625	0.883	0.754	0.526	0.843	0.718
608	0.633	0.883	0.758	0.534	0.844	0.724
609	0.642	0.875	0.758	0.531	0.837	0.726
610	0.650	0.883	0.767	0.548	0.848	0.736
611	0.633	0.883	0.758	0.534	0.844	0.724

612	0.633	0.892	0.763	0.543	0.854	0.727
613	0.650	0.883	0.767	0.548	0.848	0.736
614	0.642	0.875	0.758	0.531	0.837	0.726
615	0.642	0.875	0.758	0.531	0.837	0.726
616	0.642	0.875	0.758	0.531	0.837	0.726
617	0.642	0.875	0.758	0.531	0.837	0.726
618	0.667	0.875	0.771	0.554	0.842	0.744
619	0.667	0.875	0.771	0.554	0.842	0.744
620	0.675	0.883	0.779	0.571	0.853	0.753
621	0.667	0.875	0.771	0.554	0.842	0.744
622	0.658	0.867	0.763	0.537	0.832	0.735
623	0.667	0.892	0.779	0.573	0.860	0.751
624	0.667	0.892	0.779	0.573	0.860	0.751
625	0.667	0.892	0.779	0.573	0.860	0.751
626	0.650	0.892	0.771	0.558	0.857	0.739
627	0.650	0.900	0.775	0.568	0.867	0.743
628	0.667	0.917	0.792	0.602	0.889	0.762
629	0.658	0.900	0.779	0.575	0.868	0.749
630	0.658	0.908	0.783	0.585	0.878	0.752
631	0.667	0.900	0.783	0.583	0.870	0.755
632	0.658	0.892	0.775	0.566	0.859	0.745
633	0.667	0.892	0.779	0.573	0.860	0.751
634	0.667	0.883	0.775	0.563	0.851	0.748
635	0.658	0.892	0.775	0.566	0.859	0.745
636	0.658	0.875	0.767	0.546	0.840	0.738
637	0.667	0.883	0.775	0.563	0.851	0.748
638	0.667	0.875	0.771	0.554	0.842	0.744
639	0.667	0.875	0.771	0.554	0.842	0.744
640	0.658	0.867	0.763	0.537	0.832	0.735
641	0.650	0.867	0.758	0.529	0.830	0.729
642	0.650	0.867	0.758	0.529	0.830	0.729
643	0.658	0.867	0.763	0.537	0.832	0.735
644	0.658	0.867	0.763	0.537	0.832	0.735
645	0.667	0.858	0.763	0.535	0.825	0.737
646	0.667	0.867	0.767	0.544	0.833	0.741
647	0.667	0.867	0.767	0.544	0.833	0.741
648	0.658	0.867	0.763	0.537	0.832	0.735
649	0.658	0.875	0.767	0.546	0.840	0.738
650	0.658	0.867	0.763	0.537	0.832	0.735
651	0.658	0.875	0.767	0.546	0.840	0.738
652	0.658	0.875	0.767	0.546	0.840	0.738
653	0.658	0.883	0.771	0.556	0.849	0.742

654	0.650	0.875	0.763	0.539	0.839	0.732
655	0.667	0.875	0.771	0.554	0.842	0.744
656	0.658	0.883	0.771	0.556	0.849	0.742
657	0.650	0.892	0.771	0.558	0.857	0.739
658	0.642	0.892	0.767	0.551	0.856	0.733
659	0.642	0.892	0.767	0.551	0.856	0.733
660	0.642	0.900	0.771	0.561	0.865	0.737
661	0.658	0.900	0.779	0.575	0.868	0.749
662	0.667	0.908	0.788	0.593	0.879	0.758
663	0.675	0.900	0.788	0.590	0.871	0.761
664	0.650	0.892	0.771	0.558	0.857	0.739
665	0.658	0.900	0.779	0.575	0.868	0.749
666	0.658	0.900	0.779	0.575	0.868	0.749
667	0.642	0.900	0.771	0.561	0.865	0.737
668	0.642	0.900	0.771	0.561	0.865	0.737
669	0.642	0.892	0.767	0.551	0.856	0.733
670	0.642	0.892	0.767	0.551	0.856	0.733
671	0.650	0.892	0.771	0.558	0.857	0.739
672	0.642	0.892	0.767	0.551	0.856	0.733
673	0.642	0.892	0.767	0.551	0.856	0.733
674	0.650	0.892	0.771	0.558	0.857	0.739
675	0.642	0.883	0.763	0.541	0.846	0.730
676	0.650	0.883	0.767	0.548	0.848	0.736
677	0.650	0.883	0.767	0.548	0.848	0.736
678	0.650	0.883	0.767	0.548	0.848	0.736
679	0.650	0.883	0.767	0.548	0.848	0.736
680	0.650	0.892	0.771	0.558	0.857	0.739
681	0.650	0.892	0.771	0.558	0.857	0.739
682	0.642	0.892	0.767	0.551	0.856	0.733
683	0.642	0.892	0.767	0.551	0.856	0.733
684	0.650	0.892	0.771	0.558	0.857	0.739
685	0.642	0.892	0.767	0.551	0.856	0.733
686	0.642	0.892	0.767	0.551	0.856	0.733
687	0.633	0.892	0.763	0.543	0.854	0.727
688	0.633	0.892	0.763	0.543	0.854	0.727
689	0.642	0.892	0.767	0.551	0.856	0.733
690	0.650	0.892	0.771	0.558	0.857	0.739
691	0.650	0.892	0.771	0.558	0.857	0.739
692	0.650	0.892	0.771	0.558	0.857	0.739
693	0.650	0.892	0.771	0.558	0.857	0.739
694	0.650	0.892	0.771	0.558	0.857	0.739
695	0.658	0.892	0.775	0.566	0.859	0.745

696	0.658	0.892	0.775	0.566	0.859	0.745
697	0.658	0.900	0.779	0.575	0.868	0.749
698	0.667	0.883	0.775	0.563	0.851	0.748
699	0.667	0.892	0.779	0.573	0.860	0.751
700	0.658	0.892	0.775	0.566	0.859	0.745
701	0.658	0.892	0.775	0.566	0.859	0.745
702	0.667	0.892	0.779	0.573	0.860	0.751
703	0.667	0.892	0.779	0.573	0.860	0.751
704	0.658	0.900	0.779	0.575	0.868	0.749
705	0.658	0.892	0.775	0.566	0.859	0.745
706	0.650	0.883	0.767	0.548	0.848	0.736
707	0.667	0.883	0.775	0.563	0.851	0.748
708	0.667	0.883	0.775	0.563	0.851	0.748
709	0.658	0.883	0.771	0.556	0.849	0.742
710	0.658	0.875	0.767	0.546	0.840	0.738
711	0.667	0.883	0.775	0.563	0.851	0.748
712	0.667	0.883	0.775	0.563	0.851	0.748
713	0.667	0.875	0.771	0.554	0.842	0.744
714	0.667	0.875	0.771	0.554	0.842	0.744
715	0.658	0.892	0.775	0.566	0.859	0.745
716	0.667	0.892	0.779	0.573	0.860	0.751
717	0.667	0.892	0.779	0.573	0.860	0.751
718	0.650	0.892	0.771	0.558	0.857	0.739
719	0.650	0.883	0.767	0.548	0.848	0.736
720	0.658	0.900	0.779	0.575	0.868	0.749
721	0.642	0.892	0.767	0.551	0.856	0.733
722	0.650	0.892	0.771	0.558	0.857	0.739
723	0.650	0.892	0.771	0.558	0.857	0.739
724	0.650	0.892	0.771	0.558	0.857	0.739
725	0.642	0.883	0.763	0.541	0.846	0.730
726	0.642	0.883	0.763	0.541	0.846	0.730
727	0.658	0.883	0.771	0.556	0.849	0.742
728	0.667	0.875	0.771	0.554	0.842	0.744
729	0.675	0.867	0.771	0.552	0.835	0.747
730	0.667	0.867	0.767	0.544	0.833	0.741
731	0.667	0.875	0.771	0.554	0.842	0.744
732	0.667	0.875	0.771	0.554	0.842	0.744
733	0.658	0.875	0.767	0.546	0.840	0.738
734	0.667	0.875	0.771	0.554	0.842	0.744
735	0.667	0.875	0.771	0.554	0.842	0.744
736	0.667	0.875	0.771	0.554	0.842	0.744
737	0.650	0.875	0.763	0.539	0.839	0.732

738	0.667	0.875	0.771	0.554	0.842	0.744
739	0.658	0.867	0.763	0.537	0.832	0.735
740	0.650	0.867	0.758	0.529	0.830	0.729
741	0.650	0.867	0.758	0.529	0.830	0.729
742	0.650	0.875	0.763	0.539	0.839	0.732
743	0.658	0.867	0.763	0.537	0.832	0.735
744	0.650	0.875	0.763	0.539	0.839	0.732
745	0.642	0.883	0.763	0.541	0.846	0.730
746	0.650	0.875	0.763	0.539	0.839	0.732
747	0.642	0.875	0.758	0.531	0.837	0.726
748	0.642	0.875	0.758	0.531	0.837	0.726
749	0.642	0.867	0.754	0.522	0.828	0.723
750	0.650	0.867	0.758	0.529	0.830	0.729
751	0.658	0.867	0.763	0.537	0.832	0.735
752	0.658	0.875	0.767	0.546	0.840	0.738
753	0.650	0.875	0.763	0.539	0.839	0.732
754	0.650	0.875	0.763	0.539	0.839	0.732
755	0.650	0.867	0.758	0.529	0.830	0.729
756	0.650	0.867	0.758	0.529	0.830	0.729
757	0.658	0.867	0.763	0.537	0.832	0.735
758	0.650	0.875	0.763	0.539	0.839	0.732
759	0.642	0.867	0.754	0.522	0.828	0.723
760	0.642	0.875	0.758	0.531	0.837	0.726
761	0.642	0.883	0.763	0.541	0.846	0.730
762	0.650	0.875	0.763	0.539	0.839	0.732
763	0.650	0.883	0.767	0.548	0.848	0.736
764	0.650	0.875	0.763	0.539	0.839	0.732
765	0.650	0.875	0.763	0.539	0.839	0.732
766	0.658	0.875	0.767	0.546	0.840	0.738
767	0.658	0.883	0.771	0.556	0.849	0.742
768	0.667	0.900	0.783	0.583	0.870	0.755
769	0.658	0.900	0.779	0.575	0.868	0.749
770	0.667	0.900	0.783	0.583	0.870	0.755
771	0.658	0.900	0.779	0.575	0.868	0.749
772	0.650	0.900	0.775	0.568	0.867	0.743
773	0.650	0.892	0.771	0.558	0.857	0.739
774	0.650	0.892	0.771	0.558	0.857	0.739
775	0.650	0.892	0.771	0.558	0.857	0.739
776	0.650	0.892	0.771	0.558	0.857	0.739
777	0.650	0.900	0.775	0.568	0.867	0.743
778	0.650	0.900	0.775	0.568	0.867	0.743
779	0.658	0.883	0.771	0.556	0.849	0.742

780	0.658	0.892	0.775	0.566	0.859	0.745
781	0.658	0.892	0.775	0.566	0.859	0.745
782	0.650	0.892	0.771	0.558	0.857	0.739
783	0.650	0.875	0.763	0.539	0.839	0.732
784	0.650	0.875	0.763	0.539	0.839	0.732
785	0.658	0.875	0.767	0.546	0.840	0.738
786	0.658	0.883	0.771	0.556	0.849	0.742
787	0.658	0.883	0.771	0.556	0.849	0.742
788	0.667	0.875	0.771	0.554	0.842	0.744
789	0.667	0.892	0.779	0.573	0.860	0.751
790	0.658	0.892	0.775	0.566	0.859	0.745
791	0.667	0.883	0.775	0.563	0.851	0.748
792	0.667	0.875	0.771	0.554	0.842	0.744
793	0.667	0.875	0.771	0.554	0.842	0.744
794	0.667	0.875	0.771	0.554	0.842	0.744
795	0.667	0.883	0.775	0.563	0.851	0.748
796	0.667	0.883	0.775	0.563	0.851	0.748
797	0.667	0.883	0.775	0.563	0.851	0.748
798	0.675	0.883	0.779	0.571	0.853	0.753
799	0.675	0.883	0.779	0.571	0.853	0.753
800	0.675	0.883	0.779	0.571	0.853	0.753
801	0.675	0.883	0.779	0.571	0.853	0.753
802	0.683	0.883	0.783	0.578	0.854	0.759
803	0.692	0.892	0.792	0.595	0.865	0.769
804	0.692	0.892	0.792	0.595	0.865	0.769
805	0.700	0.900	0.800	0.612	0.875	0.778
806	0.667	0.900	0.783	0.583	0.870	0.755
807	0.683	0.900	0.792	0.598	0.872	0.766
808	0.683	0.900	0.792	0.598	0.872	0.766
809	0.675	0.900	0.788	0.590	0.871	0.761
810	0.683	0.900	0.792	0.598	0.872	0.766
811	0.675	0.892	0.783	0.580	0.862	0.757
812	0.675	0.892	0.783	0.580	0.862	0.757
813	0.683	0.892	0.788	0.588	0.863	0.763
814	0.675	0.883	0.779	0.571	0.853	0.753
815	0.675	0.883	0.779	0.571	0.853	0.753
816	0.658	0.883	0.771	0.556	0.849	0.742
817	0.658	0.875	0.767	0.546	0.840	0.738
818	0.667	0.875	0.771	0.554	0.842	0.744
819	0.667	0.875	0.771	0.554	0.842	0.744
820	0.675	0.875	0.775	0.561	0.844	0.750
821	0.658	0.875	0.767	0.546	0.840	0.738

822	0.658	0.875	0.767	0.546	0.840	0.738
823	0.692	0.875	0.783	0.576	0.847	0.761
824	0.683	0.875	0.779	0.569	0.845	0.756
825	0.692	0.875	0.783	0.576	0.847	0.761
826	0.692	0.875	0.783	0.576	0.847	0.761
827	0.692	0.875	0.783	0.576	0.847	0.761
828	0.683	0.875	0.779	0.569	0.845	0.756
829	0.675	0.883	0.779	0.571	0.853	0.753
830	0.675	0.875	0.775	0.561	0.844	0.750
831	0.675	0.875	0.775	0.561	0.844	0.750
832	0.675	0.867	0.771	0.552	0.835	0.747
833	0.675	0.867	0.771	0.552	0.835	0.747
834	0.683	0.867	0.775	0.559	0.837	0.752
835	0.683	0.867	0.775	0.559	0.837	0.752
836	0.675	0.867	0.771	0.552	0.835	0.747
837	0.675	0.867	0.771	0.552	0.835	0.747
838	0.675	0.875	0.775	0.561	0.844	0.750
839	0.675	0.875	0.775	0.561	0.844	0.750
840	0.692	0.875	0.783	0.576	0.847	0.761
841	0.692	0.867	0.779	0.567	0.838	0.758
842	0.683	0.867	0.775	0.559	0.837	0.752
843	0.683	0.875	0.779	0.569	0.845	0.756
844	0.692	0.875	0.783	0.576	0.847	0.761
845	0.692	0.858	0.775	0.558	0.830	0.755
846	0.683	0.867	0.775	0.559	0.837	0.752
847	0.692	0.867	0.779	0.567	0.838	0.758
848	0.692	0.875	0.783	0.576	0.847	0.761
849	0.683	0.883	0.783	0.578	0.854	0.759
850	0.683	0.883	0.783	0.578	0.854	0.759
851	0.683	0.892	0.788	0.588	0.863	0.763
852	0.683	0.883	0.783	0.578	0.854	0.759
853	0.692	0.883	0.788	0.586	0.856	0.765
854	0.692	0.883	0.788	0.586	0.856	0.765
855	0.692	0.892	0.792	0.595	0.865	0.769
856	0.692	0.892	0.792	0.595	0.865	0.769
857	0.692	0.892	0.792	0.595	0.865	0.769
858	0.692	0.883	0.788	0.586	0.856	0.765
859	0.692	0.883	0.788	0.586	0.856	0.765
860	0.692	0.892	0.792	0.595	0.865	0.769
861	0.692	0.883	0.788	0.586	0.856	0.765
862	0.692	0.883	0.788	0.586	0.856	0.765
863	0.692	0.867	0.779	0.567	0.838	0.758

864	0.708	0.875	0.792	0.592	0.850	0.773
865	0.708	0.892	0.800	0.610	0.867	0.780
866	0.700	0.892	0.796	0.603	0.866	0.774
867	0.700	0.883	0.792	0.593	0.857	0.771
868	0.700	0.883	0.792	0.593	0.857	0.771
869	0.700	0.875	0.788	0.584	0.848	0.767
870	0.700	0.875	0.788	0.584	0.848	0.767
871	0.700	0.875	0.788	0.584	0.848	0.767
872	0.692	0.875	0.783	0.576	0.847	0.761
873	0.692	0.867	0.779	0.567	0.838	0.758
874	0.692	0.875	0.783	0.576	0.847	0.761
875	0.692	0.875	0.783	0.576	0.847	0.761
876	0.692	0.875	0.783	0.576	0.847	0.761
877	0.692	0.883	0.788	0.586	0.856	0.765
878	0.692	0.875	0.783	0.576	0.847	0.761
879	0.692	0.875	0.783	0.576	0.847	0.761
880	0.692	0.883	0.788	0.586	0.856	0.765
881	0.692	0.875	0.783	0.576	0.847	0.761
882	0.692	0.875	0.783	0.576	0.847	0.761
883	0.692	0.883	0.788	0.586	0.856	0.765
884	0.692	0.875	0.783	0.576	0.847	0.761
885	0.692	0.883	0.788	0.586	0.856	0.765
886	0.675	0.883	0.779	0.571	0.853	0.753
887	0.675	0.875	0.775	0.561	0.844	0.750
888	0.675	0.875	0.775	0.561	0.844	0.750
889	0.675	0.883	0.779	0.571	0.853	0.753
890	0.675	0.883	0.779	0.571	0.853	0.753
891	0.675	0.883	0.779	0.571	0.853	0.753
892	0.667	0.858	0.763	0.535	0.825	0.737
893	0.683	0.850	0.767	0.541	0.820	0.745
894	0.675	0.850	0.763	0.533	0.818	0.740
895	0.675	0.875	0.775	0.561	0.844	0.750
896	0.667	0.867	0.767	0.544	0.833	0.741
897	0.667	0.867	0.767	0.544	0.833	0.741
898	0.675	0.867	0.771	0.552	0.835	0.747
899	0.675	0.858	0.767	0.543	0.827	0.743
900	0.675	0.858	0.767	0.543	0.827	0.743
901	0.675	0.867	0.771	0.552	0.835	0.747
902	0.683	0.867	0.775	0.559	0.837	0.752
903	0.683	0.867	0.775	0.559	0.837	0.752
904	0.675	0.867	0.771	0.552	0.835	0.747
905	0.683	0.858	0.771	0.550	0.828	0.749

906	0.683	0.858	0.771	0.550	0.828	0.749
907	0.683	0.858	0.771	0.550	0.828	0.749
908	0.692	0.883	0.788	0.586	0.856	0.765
909	0.692	0.875	0.783	0.576	0.847	0.761
910	0.692	0.875	0.783	0.576	0.847	0.761
911	0.692	0.875	0.783	0.576	0.847	0.761
912	0.692	0.875	0.783	0.576	0.847	0.761
913	0.692	0.875	0.783	0.576	0.847	0.761
914	0.692	0.875	0.783	0.576	0.847	0.761
915	0.692	0.883	0.788	0.586	0.856	0.765
916	0.700	0.883	0.792	0.593	0.857	0.771
917	0.700	0.892	0.796	0.603	0.866	0.774
918	0.700	0.892	0.796	0.603	0.866	0.774
919	0.700	0.892	0.796	0.603	0.866	0.774
920	0.700	0.892	0.796	0.603	0.866	0.774
921	0.700	0.892	0.796	0.603	0.866	0.774
922	0.700	0.892	0.796	0.603	0.866	0.774
923	0.700	0.892	0.796	0.603	0.866	0.774
924	0.700	0.892	0.796	0.603	0.866	0.774
925	0.700	0.892	0.796	0.603	0.866	0.774
926	0.700	0.892	0.796	0.603	0.866	0.774
927	0.683	0.892	0.788	0.588	0.863	0.763
928	0.683	0.883	0.783	0.578	0.854	0.759
929	0.692	0.883	0.788	0.586	0.856	0.765
930	0.683	0.883	0.783	0.578	0.854	0.759
931	0.683	0.883	0.783	0.578	0.854	0.759
932	0.683	0.883	0.783	0.578	0.854	0.759
933	0.683	0.892	0.788	0.588	0.863	0.763
934	0.683	0.883	0.783	0.578	0.854	0.759
935	0.683	0.883	0.783	0.578	0.854	0.759
936	0.692	0.883	0.788	0.586	0.856	0.765
937	0.692	0.883	0.788	0.586	0.856	0.765
938	0.692	0.883	0.788	0.586	0.856	0.765
939	0.683	0.883	0.783	0.578	0.854	0.759
940	0.675	0.883	0.779	0.571	0.853	0.753
941	0.683	0.883	0.783	0.578	0.854	0.759
942	0.675	0.883	0.779	0.571	0.853	0.753
943	0.675	0.883	0.779	0.571	0.853	0.753
944	0.683	0.883	0.783	0.578	0.854	0.759
945	0.683	0.883	0.783	0.578	0.854	0.759
946	0.692	0.892	0.792	0.595	0.865	0.769
947	0.683	0.892	0.788	0.588	0.863	0.763

948	0.692	0.892	0.792	0.595	0.865	0.769
949	0.692	0.900	0.796	0.605	0.874	0.772
950	0.683	0.892	0.788	0.588	0.863	0.763
951	0.683	0.892	0.788	0.588	0.863	0.763
952	0.683	0.892	0.788	0.588	0.863	0.763
953	0.675	0.883	0.779	0.571	0.853	0.753
954	0.675	0.883	0.779	0.571	0.853	0.753
955	0.675	0.883	0.779	0.571	0.853	0.753
956	0.675	0.883	0.779	0.571	0.853	0.753
957	0.675	0.875	0.775	0.561	0.844	0.750
958	0.675	0.875	0.775	0.561	0.844	0.750
959	0.675	0.875	0.775	0.561	0.844	0.750
960	0.675	0.875	0.775	0.561	0.844	0.750
961	0.675	0.883	0.779	0.571	0.853	0.753
962	0.675	0.875	0.775	0.561	0.844	0.750
963	0.675	0.883	0.779	0.571	0.853	0.753
964	0.675	0.858	0.767	0.543	0.827	0.743
965	0.675	0.883	0.779	0.571	0.853	0.753
966	0.675	0.867	0.771	0.552	0.835	0.747
967	0.667	0.875	0.771	0.554	0.842	0.744
968	0.675	0.900	0.788	0.590	0.871	0.761
969	0.667	0.900	0.783	0.583	0.870	0.755
970	0.675	0.900	0.788	0.590	0.871	0.761
971	0.675	0.892	0.783	0.580	0.862	0.757
972	0.683	0.892	0.788	0.588	0.863	0.763
973	0.675	0.892	0.783	0.580	0.862	0.757
974	0.675	0.875	0.775	0.561	0.844	0.750
975	0.683	0.875	0.779	0.569	0.845	0.756
976	0.683	0.883	0.783	0.578	0.854	0.759
977	0.667	0.883	0.775	0.563	0.851	0.748
978	0.667	0.883	0.775	0.563	0.851	0.748
979	0.675	0.892	0.783	0.580	0.862	0.757
980	0.683	0.892	0.788	0.588	0.863	0.763
981	0.683	0.892	0.788	0.588	0.863	0.763
982	0.675	0.892	0.783	0.580	0.862	0.757
983	0.692	0.883	0.788	0.586	0.856	0.765
984	0.692	0.883	0.788	0.586	0.856	0.765
985	0.683	0.883	0.783	0.578	0.854	0.759
986	0.675	0.883	0.779	0.571	0.853	0.753
987	0.675	0.900	0.788	0.590	0.871	0.761
988	0.683	0.892	0.788	0.588	0.863	0.763
989	0.675	0.883	0.779	0.571	0.853	0.753

990	0.675	0.900	0.788	0.590	0.871	0.761
991	0.683	0.900	0.792	0.598	0.872	0.766
992	0.683	0.892	0.788	0.588	0.863	0.763
993	0.675	0.900	0.788	0.590	0.871	0.761
994	0.683	0.900	0.792	0.598	0.872	0.766
995	0.692	0.900	0.796	0.605	0.874	0.772
996	0.692	0.900	0.796	0.605	0.874	0.772
997	0.692	0.892	0.792	0.595	0.865	0.769
998	0.692	0.883	0.788	0.586	0.856	0.765
999	0.675	0.875	0.775	0.561	0.844	0.750
1000	0.675	0.883	0.779	0.571	0.853	0.753
1001	0.675	0.892	0.783	0.580	0.862	0.757
1002	0.675	0.900	0.788	0.590	0.871	0.761
1003	0.675	0.900	0.788	0.590	0.871	0.761
1004	0.675	0.892	0.783	0.580	0.862	0.757
1005	0.675	0.892	0.783	0.580	0.862	0.757
1006	0.675	0.883	0.779	0.571	0.853	0.753
1007	0.675	0.883	0.779	0.571	0.853	0.753
1008	0.675	0.892	0.783	0.580	0.862	0.757
1009	0.683	0.900	0.792	0.598	0.872	0.766
1010	0.683	0.908	0.796	0.607	0.882	0.770
1011	0.683	0.900	0.792	0.598	0.872	0.766
1012	0.683	0.900	0.792	0.598	0.872	0.766
1013	0.683	0.900	0.792	0.598	0.872	0.766
1014	0.683	0.900	0.792	0.598	0.872	0.766
1015	0.692	0.900	0.796	0.605	0.874	0.772
1016	0.692	0.900	0.796	0.605	0.874	0.772
1017	0.683	0.900	0.792	0.598	0.872	0.766
1018	0.683	0.900	0.792	0.598	0.872	0.766
1019	0.683	0.917	0.800	0.617	0.891	0.774
1020	0.683	0.908	0.796	0.607	0.882	0.770

(3) Random forest

Number of features	SN	SP	ACC	MCC	Precision	F1-measure
1	0.633	0.617	0.625	0.250	0.623	0.628
2	0.717	0.667	0.692	0.384	0.683	0.699
3	0.708	0.742	0.725	0.450	0.733	0.720
4	0.758	0.800	0.779	0.559	0.791	0.774
5	0.808	0.775	0.792	0.584	0.782	0.795
6	0.800	0.792	0.796	0.592	0.793	0.797
7	0.800	0.808	0.804	0.608	0.807	0.803
8	0.817	0.800	0.808	0.617	0.803	0.810

9	0.817	0.775	0.796	0.592	0.784	0.800
10	0.817	0.808	0.813	0.625	0.810	0.813
11	0.833	0.808	0.821	0.642	0.813	0.823
12	0.808	0.808	0.808	0.617	0.808	0.808
13	0.833	0.825	0.829	0.658	0.826	0.830
14	0.867	0.817	0.842	0.684	0.825	0.846
15	0.825	0.825	0.825	0.650	0.825	0.825
16	0.875	0.808	0.842	0.685	0.820	0.847
17	0.842	0.800	0.821	0.642	0.808	0.824
18	0.833	0.792	0.813	0.626	0.800	0.816
19	0.825	0.808	0.817	0.633	0.811	0.818
20	0.833	0.842	0.838	0.675	0.840	0.837
21	0.825	0.825	0.825	0.650	0.825	0.825
22	0.842	0.800	0.821	0.642	0.808	0.824
23	0.825	0.792	0.808	0.617	0.798	0.811
24	0.875	0.842	0.858	0.717	0.847	0.861
25	0.883	0.775	0.829	0.662	0.797	0.838
26	0.850	0.833	0.842	0.683	0.836	0.843
27	0.867	0.800	0.833	0.668	0.813	0.839
28	0.883	0.783	0.833	0.670	0.803	0.841
29	0.867	0.850	0.858	0.717	0.852	0.860
30	0.850	0.800	0.825	0.651	0.810	0.829
31	0.867	0.850	0.858	0.717	0.852	0.860
32	0.850	0.817	0.833	0.667	0.823	0.836
33	0.833	0.808	0.821	0.642	0.813	0.823
34	0.833	0.808	0.821	0.642	0.813	0.823
35	0.833	0.858	0.846	0.692	0.855	0.844
36	0.858	0.808	0.833	0.668	0.817	0.837
37	0.825	0.825	0.825	0.650	0.825	0.825
38	0.867	0.867	0.867	0.733	0.867	0.867
39	0.858	0.800	0.829	0.659	0.811	0.834
40	0.842	0.817	0.829	0.659	0.821	0.831
41	0.875	0.825	0.850	0.701	0.833	0.854
42	0.842	0.825	0.833	0.667	0.828	0.835
43	0.858	0.800	0.829	0.659	0.811	0.834
44	0.858	0.825	0.842	0.684	0.831	0.844
45	0.867	0.858	0.863	0.725	0.860	0.863
46	0.867	0.842	0.854	0.709	0.846	0.856
47	0.867	0.833	0.850	0.700	0.839	0.852
48	0.867	0.808	0.838	0.676	0.819	0.842
49	0.875	0.833	0.854	0.709	0.840	0.857
50	0.825	0.858	0.842	0.684	0.853	0.839

51	0.850	0.842	0.846	0.692	0.843	0.846
52	0.817	0.833	0.825	0.650	0.831	0.824
53	0.850	0.808	0.829	0.659	0.816	0.833
54	0.833	0.850	0.842	0.683	0.847	0.840
55	0.867	0.850	0.858	0.717	0.852	0.860
56	0.875	0.842	0.858	0.717	0.847	0.861
57	0.875	0.842	0.858	0.717	0.847	0.861
58	0.867	0.817	0.842	0.684	0.825	0.846
59	0.867	0.833	0.850	0.700	0.839	0.852
60	0.858	0.833	0.846	0.692	0.837	0.848
61	0.858	0.833	0.846	0.692	0.837	0.848
62	0.817	0.850	0.833	0.667	0.845	0.831
63	0.867	0.825	0.846	0.692	0.832	0.849
64	0.875	0.850	0.863	0.725	0.854	0.864
65	0.850	0.833	0.842	0.683	0.836	0.843
66	0.850	0.825	0.838	0.675	0.829	0.840
67	0.842	0.825	0.833	0.667	0.828	0.835
68	0.875	0.817	0.846	0.693	0.827	0.850
69	0.858	0.808	0.833	0.668	0.817	0.837
70	0.850	0.817	0.833	0.667	0.823	0.836
71	0.825	0.842	0.833	0.667	0.839	0.832
72	0.858	0.792	0.825	0.651	0.805	0.831
73	0.875	0.792	0.833	0.669	0.808	0.840
74	0.842	0.842	0.842	0.683	0.842	0.842
75	0.850	0.817	0.833	0.667	0.823	0.836
76	0.850	0.808	0.829	0.659	0.816	0.833
77	0.842	0.842	0.842	0.683	0.842	0.842
78	0.867	0.817	0.842	0.684	0.825	0.846
79	0.875	0.817	0.846	0.693	0.827	0.850
80	0.875	0.842	0.858	0.717	0.847	0.861
81	0.858	0.808	0.833	0.668	0.817	0.837
82	0.833	0.842	0.838	0.675	0.840	0.837
83	0.858	0.817	0.838	0.676	0.824	0.841
84	0.833	0.850	0.842	0.683	0.847	0.840
85	0.858	0.800	0.829	0.659	0.811	0.834
86	0.858	0.825	0.842	0.684	0.831	0.844
87	0.842	0.825	0.833	0.667	0.828	0.835
88	0.858	0.817	0.838	0.676	0.824	0.841
89	0.867	0.817	0.842	0.684	0.825	0.846
90	0.833	0.817	0.825	0.650	0.820	0.826
91	0.850	0.833	0.842	0.683	0.836	0.843
92	0.850	0.817	0.833	0.667	0.823	0.836

93	0.850	0.817	0.833	0.667	0.823	0.836
94	0.858	0.792	0.825	0.651	0.805	0.831
95	0.850	0.850	0.850	0.700	0.850	0.850
96	0.875	0.817	0.846	0.693	0.827	0.850
97	0.875	0.858	0.867	0.733	0.861	0.868
98	0.842	0.800	0.821	0.642	0.808	0.824
99	0.850	0.858	0.854	0.708	0.857	0.854
100	0.850	0.825	0.838	0.675	0.829	0.840
101	0.858	0.842	0.850	0.700	0.844	0.851
102	0.825	0.842	0.833	0.667	0.839	0.832
103	0.858	0.825	0.842	0.684	0.831	0.844
104	0.850	0.833	0.842	0.683	0.836	0.843
105	0.867	0.825	0.846	0.692	0.832	0.849
106	0.842	0.808	0.825	0.650	0.815	0.828
107	0.858	0.808	0.833	0.668	0.817	0.837
108	0.875	0.825	0.850	0.701	0.833	0.854
109	0.842	0.833	0.838	0.675	0.835	0.838
110	0.858	0.825	0.842	0.684	0.831	0.844
111	0.867	0.850	0.858	0.717	0.852	0.860
112	0.858	0.842	0.850	0.700	0.844	0.851
113	0.833	0.850	0.842	0.683	0.847	0.840
114	0.850	0.858	0.854	0.708	0.857	0.854
115	0.850	0.833	0.842	0.683	0.836	0.843
116	0.867	0.817	0.842	0.684	0.825	0.846
117	0.867	0.833	0.850	0.700	0.839	0.852
118	0.858	0.833	0.846	0.692	0.837	0.848
119	0.875	0.808	0.842	0.685	0.820	0.847
120	0.875	0.817	0.846	0.693	0.827	0.850
121	0.842	0.833	0.838	0.675	0.835	0.838
122	0.850	0.825	0.838	0.675	0.829	0.840
123	0.842	0.842	0.842	0.683	0.842	0.842
124	0.867	0.833	0.850	0.700	0.839	0.852
125	0.833	0.833	0.833	0.667	0.833	0.833
126	0.850	0.825	0.838	0.675	0.829	0.840
127	0.858	0.825	0.842	0.684	0.831	0.844
128	0.867	0.833	0.850	0.700	0.839	0.852
129	0.858	0.825	0.842	0.684	0.831	0.844
130	0.850	0.833	0.842	0.683	0.836	0.843
131	0.825	0.842	0.833	0.667	0.839	0.832
132	0.875	0.833	0.854	0.709	0.840	0.857
133	0.867	0.808	0.838	0.676	0.819	0.842
134	0.850	0.858	0.854	0.708	0.857	0.854

135	0.858	0.817	0.838	0.676	0.824	0.841
136	0.850	0.817	0.833	0.667	0.823	0.836
137	0.883	0.833	0.858	0.718	0.841	0.862
138	0.850	0.850	0.850	0.700	0.850	0.850
139	0.892	0.825	0.858	0.718	0.836	0.863
140	0.858	0.817	0.838	0.676	0.824	0.841
141	0.842	0.808	0.825	0.650	0.815	0.828
142	0.875	0.825	0.850	0.701	0.833	0.854
143	0.867	0.808	0.838	0.676	0.819	0.842
144	0.842	0.842	0.842	0.683	0.842	0.842
145	0.850	0.817	0.833	0.667	0.823	0.836
146	0.858	0.842	0.850	0.700	0.844	0.851
147	0.842	0.817	0.829	0.659	0.821	0.831
148	0.858	0.825	0.842	0.684	0.831	0.844
149	0.850	0.800	0.825	0.651	0.810	0.829
150	0.858	0.808	0.833	0.668	0.817	0.837
151	0.858	0.825	0.842	0.684	0.831	0.844
152	0.850	0.850	0.850	0.700	0.850	0.850
153	0.867	0.833	0.850	0.700	0.839	0.852
154	0.850	0.808	0.829	0.659	0.816	0.833
155	0.892	0.792	0.842	0.687	0.811	0.849
156	0.825	0.808	0.817	0.633	0.811	0.818
157	0.858	0.792	0.825	0.651	0.805	0.831
158	0.850	0.808	0.829	0.659	0.816	0.833
159	0.850	0.808	0.829	0.659	0.816	0.833
160	0.858	0.808	0.833	0.668	0.817	0.837
161	0.875	0.817	0.846	0.693	0.827	0.850
162	0.875	0.833	0.854	0.709	0.840	0.857
163	0.850	0.800	0.825	0.651	0.810	0.829
164	0.867	0.833	0.850	0.700	0.839	0.852
165	0.850	0.817	0.833	0.667	0.823	0.836
166	0.892	0.825	0.858	0.718	0.836	0.863
167	0.850	0.817	0.833	0.667	0.823	0.836
168	0.867	0.817	0.842	0.684	0.825	0.846
169	0.858	0.808	0.833	0.668	0.817	0.837
170	0.867	0.833	0.850	0.700	0.839	0.852
171	0.875	0.783	0.829	0.661	0.802	0.837
172	0.858	0.833	0.846	0.692	0.837	0.848
173	0.858	0.808	0.833	0.668	0.817	0.837
174	0.850	0.842	0.846	0.692	0.843	0.846
175	0.842	0.808	0.825	0.650	0.815	0.828
176	0.867	0.825	0.846	0.692	0.832	0.849

177	0.875	0.808	0.842	0.685	0.820	0.847
178	0.842	0.800	0.821	0.642	0.808	0.824
179	0.867	0.833	0.850	0.700	0.839	0.852
180	0.850	0.858	0.854	0.708	0.857	0.854
181	0.858	0.808	0.833	0.668	0.817	0.837
182	0.875	0.825	0.850	0.701	0.833	0.854
183	0.858	0.842	0.850	0.700	0.844	0.851
184	0.850	0.792	0.821	0.643	0.803	0.826
185	0.867	0.833	0.850	0.700	0.839	0.852
186	0.858	0.808	0.833	0.668	0.817	0.837
187	0.833	0.808	0.821	0.642	0.813	0.823
188	0.858	0.808	0.833	0.668	0.817	0.837
189	0.883	0.833	0.858	0.718	0.841	0.862
190	0.850	0.825	0.838	0.675	0.829	0.840
191	0.842	0.817	0.829	0.659	0.821	0.831
192	0.842	0.808	0.825	0.650	0.815	0.828
193	0.842	0.817	0.829	0.659	0.821	0.831
194	0.858	0.850	0.854	0.708	0.851	0.855
195	0.850	0.825	0.838	0.675	0.829	0.840
196	0.850	0.850	0.850	0.700	0.850	0.850
197	0.867	0.792	0.829	0.660	0.806	0.835
198	0.842	0.817	0.829	0.659	0.821	0.831
199	0.850	0.800	0.825	0.651	0.810	0.829
200	0.858	0.817	0.838	0.676	0.824	0.841
201	0.850	0.825	0.838	0.675	0.829	0.840
202	0.883	0.833	0.858	0.718	0.841	0.862
203	0.842	0.817	0.829	0.659	0.821	0.831
204	0.883	0.833	0.858	0.718	0.841	0.862
205	0.858	0.817	0.838	0.676	0.824	0.841
206	0.892	0.825	0.858	0.718	0.836	0.863
207	0.842	0.808	0.825	0.650	0.815	0.828
208	0.842	0.842	0.842	0.683	0.842	0.842
209	0.842	0.842	0.842	0.683	0.842	0.842
210	0.875	0.825	0.850	0.701	0.833	0.854
211	0.850	0.783	0.817	0.635	0.797	0.823
212	0.850	0.808	0.829	0.659	0.816	0.833
213	0.875	0.800	0.838	0.677	0.814	0.843
214	0.858	0.792	0.825	0.651	0.805	0.831
215	0.850	0.808	0.829	0.659	0.816	0.833
216	0.858	0.842	0.850	0.700	0.844	0.851
217	0.858	0.842	0.850	0.700	0.844	0.851
218	0.858	0.808	0.833	0.668	0.817	0.837

219	0.833	0.800	0.817	0.634	0.806	0.820
220	0.842	0.800	0.821	0.642	0.808	0.824
221	0.850	0.808	0.829	0.659	0.816	0.833
222	0.842	0.800	0.821	0.642	0.808	0.824
223	0.850	0.783	0.817	0.635	0.797	0.823
224	0.842	0.817	0.829	0.659	0.821	0.831
225	0.858	0.825	0.842	0.684	0.831	0.844
226	0.850	0.817	0.833	0.667	0.823	0.836
227	0.850	0.842	0.846	0.692	0.843	0.846
228	0.858	0.817	0.838	0.676	0.824	0.841
229	0.883	0.842	0.863	0.726	0.848	0.865
230	0.858	0.833	0.846	0.692	0.837	0.848
231	0.867	0.800	0.833	0.668	0.813	0.839
232	0.858	0.817	0.838	0.676	0.824	0.841
233	0.850	0.825	0.838	0.675	0.829	0.840
234	0.858	0.825	0.842	0.684	0.831	0.844
235	0.842	0.833	0.838	0.675	0.835	0.838
236	0.825	0.800	0.813	0.625	0.805	0.815
237	0.842	0.833	0.838	0.675	0.835	0.838
238	0.825	0.817	0.821	0.642	0.818	0.822
239	0.858	0.817	0.838	0.676	0.824	0.841
240	0.867	0.817	0.842	0.684	0.825	0.846
241	0.842	0.808	0.825	0.650	0.815	0.828
242	0.842	0.800	0.821	0.642	0.808	0.824
243	0.842	0.842	0.842	0.683	0.842	0.842
244	0.867	0.842	0.854	0.709	0.846	0.856
245	0.825	0.833	0.829	0.658	0.832	0.828
246	0.842	0.833	0.838	0.675	0.835	0.838
247	0.867	0.842	0.854	0.709	0.846	0.856
248	0.867	0.808	0.838	0.676	0.819	0.842
249	0.867	0.817	0.842	0.684	0.825	0.846
250	0.842	0.808	0.825	0.650	0.815	0.828
251	0.858	0.792	0.825	0.651	0.805	0.831
252	0.875	0.800	0.838	0.677	0.814	0.843
253	0.858	0.825	0.842	0.684	0.831	0.844
254	0.833	0.817	0.825	0.650	0.820	0.826
255	0.850	0.825	0.838	0.675	0.829	0.840
256	0.858	0.842	0.850	0.700	0.844	0.851
257	0.833	0.825	0.829	0.658	0.826	0.830
258	0.817	0.858	0.838	0.676	0.852	0.834
259	0.833	0.792	0.813	0.626	0.800	0.816
260	0.850	0.825	0.838	0.675	0.829	0.840

261	0.842	0.808	0.825	0.650	0.815	0.828
262	0.867	0.867	0.867	0.733	0.867	0.867
263	0.825	0.792	0.808	0.617	0.798	0.811
264	0.858	0.800	0.829	0.659	0.811	0.834
265	0.858	0.817	0.838	0.676	0.824	0.841
266	0.817	0.775	0.796	0.592	0.784	0.800
267	0.842	0.808	0.825	0.650	0.815	0.828
268	0.850	0.792	0.821	0.643	0.803	0.826
269	0.850	0.833	0.842	0.683	0.836	0.843
270	0.850	0.817	0.833	0.667	0.823	0.836
271	0.850	0.825	0.838	0.675	0.829	0.840
272	0.892	0.817	0.854	0.710	0.829	0.859
273	0.850	0.833	0.842	0.683	0.836	0.843
274	0.875	0.817	0.846	0.693	0.827	0.850
275	0.858	0.817	0.838	0.676	0.824	0.841
276	0.850	0.800	0.825	0.651	0.810	0.829
277	0.842	0.850	0.846	0.692	0.849	0.845
278	0.892	0.825	0.858	0.718	0.836	0.863
279	0.850	0.817	0.833	0.667	0.823	0.836
280	0.850	0.817	0.833	0.667	0.823	0.836
281	0.858	0.817	0.838	0.676	0.824	0.841
282	0.892	0.792	0.842	0.687	0.811	0.849
283	0.850	0.833	0.842	0.683	0.836	0.843
284	0.833	0.808	0.821	0.642	0.813	0.823
285	0.858	0.800	0.829	0.659	0.811	0.834
286	0.850	0.800	0.825	0.651	0.810	0.829
287	0.850	0.792	0.821	0.643	0.803	0.826
288	0.842	0.825	0.833	0.667	0.828	0.835
289	0.867	0.850	0.858	0.717	0.852	0.860
290	0.867	0.817	0.842	0.684	0.825	0.846
291	0.850	0.817	0.833	0.667	0.823	0.836
292	0.833	0.850	0.842	0.683	0.847	0.840
293	0.833	0.800	0.817	0.634	0.806	0.820
294	0.850	0.850	0.850	0.700	0.850	0.850
295	0.842	0.817	0.829	0.659	0.821	0.831
296	0.825	0.792	0.808	0.617	0.798	0.811
297	0.850	0.817	0.833	0.667	0.823	0.836
298	0.858	0.817	0.838	0.676	0.824	0.841
299	0.867	0.792	0.829	0.660	0.806	0.835
300	0.850	0.825	0.838	0.675	0.829	0.840
301	0.842	0.808	0.825	0.650	0.815	0.828
302	0.867	0.808	0.838	0.676	0.819	0.842

303	0.842	0.825	0.833	0.667	0.828	0.835
304	0.850	0.842	0.846	0.692	0.843	0.846
305	0.833	0.775	0.804	0.609	0.787	0.810
306	0.842	0.783	0.813	0.626	0.795	0.818
307	0.842	0.833	0.838	0.675	0.835	0.838
308	0.833	0.783	0.808	0.617	0.794	0.813
309	0.808	0.842	0.825	0.650	0.836	0.822
310	0.858	0.808	0.833	0.668	0.817	0.837
311	0.833	0.800	0.817	0.634	0.806	0.820
312	0.850	0.833	0.842	0.683	0.836	0.843
313	0.850	0.808	0.829	0.659	0.816	0.833
314	0.833	0.817	0.825	0.650	0.820	0.826
315	0.858	0.817	0.838	0.676	0.824	0.841
316	0.867	0.808	0.838	0.676	0.819	0.842
317	0.883	0.825	0.854	0.710	0.835	0.858
318	0.850	0.825	0.838	0.675	0.829	0.840
319	0.833	0.817	0.825	0.650	0.820	0.826
320	0.842	0.833	0.838	0.675	0.835	0.838
321	0.842	0.825	0.833	0.667	0.828	0.835
322	0.850	0.825	0.838	0.675	0.829	0.840
323	0.875	0.808	0.842	0.685	0.820	0.847
324	0.842	0.800	0.821	0.642	0.808	0.824
325	0.858	0.792	0.825	0.651	0.805	0.831
326	0.875	0.775	0.825	0.653	0.795	0.833
327	0.850	0.825	0.838	0.675	0.829	0.840
328	0.867	0.850	0.858	0.717	0.852	0.860
329	0.858	0.833	0.846	0.692	0.837	0.848
330	0.833	0.792	0.813	0.626	0.800	0.816
331	0.842	0.867	0.854	0.709	0.863	0.852
332	0.858	0.842	0.850	0.700	0.844	0.851
333	0.875	0.825	0.850	0.701	0.833	0.854
334	0.850	0.800	0.825	0.651	0.810	0.829
335	0.892	0.842	0.867	0.734	0.849	0.870
336	0.850	0.808	0.829	0.659	0.816	0.833
337	0.842	0.825	0.833	0.667	0.828	0.835
338	0.858	0.825	0.842	0.684	0.831	0.844
339	0.817	0.817	0.817	0.633	0.817	0.817
340	0.858	0.808	0.833	0.668	0.817	0.837
341	0.867	0.767	0.817	0.637	0.788	0.825
342	0.858	0.792	0.825	0.651	0.805	0.831
343	0.867	0.825	0.846	0.692	0.832	0.849
344	0.883	0.792	0.838	0.678	0.809	0.845

345	0.858	0.783	0.821	0.643	0.798	0.827
346	0.858	0.808	0.833	0.668	0.817	0.837
347	0.867	0.817	0.842	0.684	0.825	0.846
348	0.833	0.800	0.817	0.634	0.806	0.820
349	0.825	0.833	0.829	0.658	0.832	0.828
350	0.858	0.842	0.850	0.700	0.844	0.851
351	0.850	0.800	0.825	0.651	0.810	0.829
352	0.867	0.792	0.829	0.660	0.806	0.835
353	0.842	0.800	0.821	0.642	0.808	0.824
354	0.842	0.842	0.842	0.683	0.842	0.842
355	0.842	0.817	0.829	0.659	0.821	0.831
356	0.858	0.817	0.838	0.676	0.824	0.841
357	0.850	0.808	0.829	0.659	0.816	0.833
358	0.833	0.842	0.838	0.675	0.840	0.837
359	0.850	0.808	0.829	0.659	0.816	0.833
360	0.850	0.850	0.850	0.700	0.850	0.850
361	0.858	0.825	0.842	0.684	0.831	0.844
362	0.858	0.833	0.846	0.692	0.837	0.848
363	0.825	0.817	0.821	0.642	0.818	0.822
364	0.842	0.808	0.825	0.650	0.815	0.828
365	0.858	0.833	0.846	0.692	0.837	0.848
366	0.850	0.792	0.821	0.643	0.803	0.826
367	0.817	0.850	0.833	0.667	0.845	0.831
368	0.858	0.825	0.842	0.684	0.831	0.844
369	0.858	0.817	0.838	0.676	0.824	0.841
370	0.833	0.808	0.821	0.642	0.813	0.823
371	0.825	0.842	0.833	0.667	0.839	0.832
372	0.808	0.833	0.821	0.642	0.829	0.819
373	0.833	0.783	0.808	0.617	0.794	0.813
374	0.817	0.808	0.813	0.625	0.810	0.813
375	0.825	0.825	0.825	0.650	0.825	0.825
376	0.867	0.858	0.863	0.725	0.860	0.863
377	0.850	0.825	0.838	0.675	0.829	0.840
378	0.800	0.850	0.825	0.651	0.842	0.821
379	0.833	0.808	0.821	0.642	0.813	0.823
380	0.875	0.792	0.833	0.669	0.808	0.840
381	0.833	0.783	0.808	0.617	0.794	0.813
382	0.850	0.808	0.829	0.659	0.816	0.833
383	0.842	0.808	0.825	0.650	0.815	0.828
384	0.875	0.833	0.854	0.709	0.840	0.857
385	0.842	0.808	0.825	0.650	0.815	0.828
386	0.842	0.850	0.846	0.692	0.849	0.845

387	0.833	0.833	0.833	0.667	0.833	0.833
388	0.833	0.817	0.825	0.650	0.820	0.826
389	0.842	0.808	0.825	0.650	0.815	0.828
390	0.833	0.867	0.850	0.700	0.862	0.847
391	0.850	0.833	0.842	0.683	0.836	0.843
392	0.842	0.858	0.850	0.700	0.856	0.849
393	0.867	0.800	0.833	0.668	0.813	0.839
394	0.817	0.808	0.813	0.625	0.810	0.813
395	0.825	0.825	0.825	0.650	0.825	0.825
396	0.858	0.817	0.838	0.676	0.824	0.841
397	0.883	0.800	0.842	0.686	0.815	0.848
398	0.883	0.808	0.846	0.694	0.822	0.851
399	0.867	0.808	0.838	0.676	0.819	0.842
400	0.858	0.817	0.838	0.676	0.824	0.841
401	0.858	0.792	0.825	0.651	0.805	0.831
402	0.842	0.833	0.838	0.675	0.835	0.838
403	0.858	0.808	0.833	0.668	0.817	0.837
404	0.875	0.808	0.842	0.685	0.820	0.847
405	0.867	0.808	0.838	0.676	0.819	0.842
406	0.875	0.825	0.850	0.701	0.833	0.854
407	0.842	0.792	0.817	0.634	0.802	0.821
408	0.850	0.825	0.838	0.675	0.829	0.840
409	0.833	0.825	0.829	0.658	0.826	0.830
410	0.858	0.808	0.833	0.668	0.817	0.837
411	0.825	0.817	0.821	0.642	0.818	0.822
412	0.867	0.825	0.846	0.692	0.832	0.849
413	0.842	0.842	0.842	0.683	0.842	0.842
414	0.842	0.850	0.846	0.692	0.849	0.845
415	0.850	0.825	0.838	0.675	0.829	0.840
416	0.858	0.817	0.838	0.676	0.824	0.841
417	0.858	0.800	0.829	0.659	0.811	0.834
418	0.883	0.800	0.842	0.686	0.815	0.848
419	0.867	0.842	0.854	0.709	0.846	0.856
420	0.858	0.783	0.821	0.643	0.798	0.827
421	0.858	0.808	0.833	0.668	0.817	0.837
422	0.867	0.767	0.817	0.637	0.788	0.825
423	0.842	0.808	0.825	0.650	0.815	0.828
424	0.842	0.850	0.846	0.692	0.849	0.845
425	0.842	0.825	0.833	0.667	0.828	0.835
426	0.850	0.817	0.833	0.667	0.823	0.836
427	0.867	0.842	0.854	0.709	0.846	0.856
428	0.867	0.792	0.829	0.660	0.806	0.835

429	0.858	0.817	0.838	0.676	0.824	0.841
430	0.867	0.775	0.821	0.644	0.794	0.829
431	0.875	0.817	0.846	0.693	0.827	0.850
432	0.858	0.825	0.842	0.684	0.831	0.844
433	0.850	0.783	0.817	0.635	0.797	0.823
434	0.825	0.800	0.813	0.625	0.805	0.815
435	0.842	0.800	0.821	0.642	0.808	0.824
436	0.833	0.825	0.829	0.658	0.826	0.830
437	0.867	0.825	0.846	0.692	0.832	0.849
438	0.808	0.792	0.800	0.600	0.795	0.802
439	0.833	0.808	0.821	0.642	0.813	0.823
440	0.800	0.783	0.792	0.583	0.787	0.793
441	0.858	0.808	0.833	0.668	0.817	0.837
442	0.842	0.833	0.838	0.675	0.835	0.838
443	0.842	0.808	0.825	0.650	0.815	0.828
444	0.850	0.833	0.842	0.683	0.836	0.843
445	0.800	0.808	0.804	0.608	0.807	0.803
446	0.825	0.850	0.838	0.675	0.846	0.835
447	0.850	0.825	0.838	0.675	0.829	0.840
448	0.833	0.808	0.821	0.642	0.813	0.823
449	0.850	0.800	0.825	0.651	0.810	0.829
450	0.842	0.808	0.825	0.650	0.815	0.828
451	0.858	0.817	0.838	0.676	0.824	0.841
452	0.883	0.842	0.863	0.726	0.848	0.865
453	0.850	0.775	0.813	0.627	0.791	0.819
454	0.825	0.825	0.825	0.650	0.825	0.825
455	0.842	0.833	0.838	0.675	0.835	0.838
456	0.842	0.783	0.813	0.626	0.795	0.818
457	0.850	0.817	0.833	0.667	0.823	0.836
458	0.842	0.800	0.821	0.642	0.808	0.824
459	0.842	0.808	0.825	0.650	0.815	0.828
460	0.850	0.842	0.846	0.692	0.843	0.846
461	0.825	0.808	0.817	0.633	0.811	0.818
462	0.867	0.842	0.854	0.709	0.846	0.856
463	0.825	0.775	0.800	0.601	0.786	0.805
464	0.875	0.800	0.838	0.677	0.814	0.843
465	0.850	0.783	0.817	0.635	0.797	0.823
466	0.825	0.842	0.833	0.667	0.839	0.832
467	0.842	0.833	0.838	0.675	0.835	0.838
468	0.858	0.775	0.817	0.636	0.792	0.824
469	0.883	0.817	0.850	0.702	0.828	0.855
470	0.833	0.792	0.813	0.626	0.800	0.816

471	0.858	0.783	0.821	0.643	0.798	0.827
472	0.825	0.817	0.821	0.642	0.818	0.822
473	0.858	0.808	0.833	0.668	0.817	0.837
474	0.825	0.842	0.833	0.667	0.839	0.832
475	0.817	0.817	0.817	0.633	0.817	0.817
476	0.875	0.842	0.858	0.717	0.847	0.861
477	0.817	0.800	0.808	0.617	0.803	0.810
478	0.825	0.800	0.813	0.625	0.805	0.815
479	0.833	0.808	0.821	0.642	0.813	0.823
480	0.850	0.825	0.838	0.675	0.829	0.840
481	0.858	0.842	0.850	0.700	0.844	0.851
482	0.842	0.825	0.833	0.667	0.828	0.835
483	0.842	0.817	0.829	0.659	0.821	0.831
484	0.850	0.783	0.817	0.635	0.797	0.823
485	0.867	0.792	0.829	0.660	0.806	0.835
486	0.875	0.800	0.838	0.677	0.814	0.843
487	0.842	0.775	0.808	0.618	0.789	0.815
488	0.817	0.858	0.838	0.676	0.852	0.834
489	0.833	0.825	0.829	0.658	0.826	0.830
490	0.858	0.808	0.833	0.668	0.817	0.837
491	0.842	0.825	0.833	0.667	0.828	0.835
492	0.833	0.825	0.829	0.658	0.826	0.830
493	0.850	0.833	0.842	0.683	0.836	0.843
494	0.875	0.825	0.850	0.701	0.833	0.854
495	0.867	0.833	0.850	0.700	0.839	0.852
496	0.833	0.808	0.821	0.642	0.813	0.823
497	0.850	0.817	0.833	0.667	0.823	0.836
498	0.883	0.817	0.850	0.702	0.828	0.855
499	0.842	0.800	0.821	0.642	0.808	0.824
500	0.858	0.842	0.850	0.700	0.844	0.851
501	0.850	0.817	0.833	0.667	0.823	0.836
502	0.833	0.833	0.833	0.667	0.833	0.833
503	0.833	0.833	0.833	0.667	0.833	0.833
504	0.842	0.808	0.825	0.650	0.815	0.828
505	0.850	0.825	0.838	0.675	0.829	0.840
506	0.858	0.800	0.829	0.659	0.811	0.834
507	0.833	0.858	0.846	0.692	0.855	0.844
508	0.850	0.825	0.838	0.675	0.829	0.840
509	0.825	0.800	0.813	0.625	0.805	0.815
510	0.867	0.792	0.829	0.660	0.806	0.835
511	0.833	0.825	0.829	0.658	0.826	0.830
512	0.825	0.808	0.817	0.633	0.811	0.818

513	0.858	0.833	0.846	0.692	0.837	0.848
514	0.858	0.775	0.817	0.636	0.792	0.824
515	0.808	0.800	0.804	0.608	0.802	0.805
516	0.850	0.783	0.817	0.635	0.797	0.823
517	0.850	0.783	0.817	0.635	0.797	0.823
518	0.858	0.792	0.825	0.651	0.805	0.831
519	0.858	0.783	0.821	0.643	0.798	0.827
520	0.858	0.842	0.850	0.700	0.844	0.851
521	0.825	0.808	0.817	0.633	0.811	0.818
522	0.858	0.792	0.825	0.651	0.805	0.831
523	0.833	0.825	0.829	0.658	0.826	0.830
524	0.858	0.825	0.842	0.684	0.831	0.844
525	0.825	0.833	0.829	0.658	0.832	0.828
526	0.850	0.800	0.825	0.651	0.810	0.829
527	0.825	0.858	0.842	0.684	0.853	0.839
528	0.825	0.867	0.846	0.692	0.861	0.843
529	0.858	0.808	0.833	0.668	0.817	0.837
530	0.858	0.808	0.833	0.668	0.817	0.837
531	0.850	0.817	0.833	0.667	0.823	0.836
532	0.833	0.850	0.842	0.683	0.847	0.840
533	0.875	0.808	0.842	0.685	0.820	0.847
534	0.858	0.833	0.846	0.692	0.837	0.848
535	0.850	0.800	0.825	0.651	0.810	0.829
536	0.842	0.850	0.846	0.692	0.849	0.845
537	0.858	0.842	0.850	0.700	0.844	0.851
538	0.858	0.833	0.846	0.692	0.837	0.848
539	0.833	0.817	0.825	0.650	0.820	0.826
540	0.808	0.833	0.821	0.642	0.829	0.819
541	0.850	0.833	0.842	0.683	0.836	0.843
542	0.833	0.808	0.821	0.642	0.813	0.823
543	0.875	0.867	0.871	0.742	0.868	0.871
544	0.833	0.775	0.804	0.609	0.787	0.810
545	0.825	0.808	0.817	0.633	0.811	0.818
546	0.842	0.792	0.817	0.634	0.802	0.821
547	0.842	0.808	0.825	0.650	0.815	0.828
548	0.833	0.808	0.821	0.642	0.813	0.823
549	0.833	0.800	0.817	0.634	0.806	0.820
550	0.850	0.817	0.833	0.667	0.823	0.836
551	0.867	0.808	0.838	0.676	0.819	0.842
552	0.858	0.808	0.833	0.668	0.817	0.837
553	0.842	0.800	0.821	0.642	0.808	0.824
554	0.817	0.800	0.808	0.617	0.803	0.810

555	0.842	0.817	0.829	0.659	0.821	0.831
556	0.858	0.808	0.833	0.668	0.817	0.837
557	0.858	0.808	0.833	0.668	0.817	0.837
558	0.817	0.842	0.829	0.659	0.838	0.827
559	0.858	0.758	0.808	0.620	0.780	0.817
560	0.842	0.808	0.825	0.650	0.815	0.828
561	0.800	0.825	0.813	0.625	0.821	0.810
562	0.833	0.800	0.817	0.634	0.806	0.820
563	0.883	0.817	0.850	0.702	0.828	0.855
564	0.858	0.833	0.846	0.692	0.837	0.848
565	0.867	0.858	0.863	0.725	0.860	0.863
566	0.850	0.833	0.842	0.683	0.836	0.843
567	0.808	0.800	0.804	0.608	0.802	0.805
568	0.875	0.792	0.833	0.669	0.808	0.840
569	0.850	0.775	0.813	0.627	0.791	0.819
570	0.842	0.808	0.825	0.650	0.815	0.828
571	0.833	0.783	0.808	0.617	0.794	0.813
572	0.817	0.833	0.825	0.650	0.831	0.824
573	0.850	0.800	0.825	0.651	0.810	0.829
574	0.817	0.833	0.825	0.650	0.831	0.824
575	0.858	0.825	0.842	0.684	0.831	0.844
576	0.833	0.825	0.829	0.658	0.826	0.830
577	0.833	0.808	0.821	0.642	0.813	0.823
578	0.850	0.825	0.838	0.675	0.829	0.840
579	0.833	0.817	0.825	0.650	0.820	0.826
580	0.842	0.825	0.833	0.667	0.828	0.835
581	0.850	0.842	0.846	0.692	0.843	0.846
582	0.833	0.775	0.804	0.609	0.787	0.810
583	0.850	0.792	0.821	0.643	0.803	0.826
584	0.825	0.817	0.821	0.642	0.818	0.822
585	0.858	0.867	0.863	0.725	0.866	0.862
586	0.825	0.825	0.825	0.650	0.825	0.825
587	0.833	0.783	0.808	0.617	0.794	0.813
588	0.842	0.783	0.813	0.626	0.795	0.818
589	0.808	0.833	0.821	0.642	0.829	0.819
590	0.792	0.767	0.779	0.559	0.772	0.782
591	0.867	0.817	0.842	0.684	0.825	0.846
592	0.850	0.800	0.825	0.651	0.810	0.829
593	0.875	0.817	0.846	0.693	0.827	0.850
594	0.842	0.783	0.813	0.626	0.795	0.818
595	0.808	0.825	0.817	0.633	0.822	0.815
596	0.850	0.825	0.838	0.675	0.829	0.840

597	0.842	0.858	0.850	0.700	0.856	0.849
598	0.808	0.825	0.817	0.633	0.822	0.815
599	0.850	0.808	0.829	0.659	0.816	0.833
600	0.800	0.808	0.804	0.608	0.807	0.803
601	0.842	0.792	0.817	0.634	0.802	0.821
602	0.867	0.783	0.825	0.652	0.800	0.832
603	0.825	0.775	0.800	0.601	0.786	0.805
604	0.842	0.808	0.825	0.650	0.815	0.828
605	0.850	0.833	0.842	0.683	0.836	0.843
606	0.817	0.858	0.838	0.676	0.852	0.834
607	0.842	0.808	0.825	0.650	0.815	0.828
608	0.817	0.842	0.829	0.659	0.838	0.827
609	0.842	0.825	0.833	0.667	0.828	0.835
610	0.842	0.825	0.833	0.667	0.828	0.835
611	0.858	0.850	0.854	0.708	0.851	0.855
612	0.867	0.767	0.817	0.637	0.788	0.825
613	0.858	0.808	0.833	0.668	0.817	0.837
614	0.800	0.842	0.821	0.642	0.835	0.817
615	0.850	0.825	0.838	0.675	0.829	0.840
616	0.833	0.808	0.821	0.642	0.813	0.823
617	0.850	0.792	0.821	0.643	0.803	0.826
618	0.850	0.792	0.821	0.643	0.803	0.826
619	0.825	0.808	0.817	0.633	0.811	0.818
620	0.850	0.817	0.833	0.667	0.823	0.836
621	0.817	0.808	0.813	0.625	0.810	0.813
622	0.850	0.825	0.838	0.675	0.829	0.840
623	0.825	0.800	0.813	0.625	0.805	0.815
624	0.858	0.817	0.838	0.676	0.824	0.841
625	0.817	0.817	0.817	0.633	0.817	0.817
626	0.825	0.858	0.842	0.684	0.853	0.839
627	0.867	0.808	0.838	0.676	0.819	0.842
628	0.858	0.825	0.842	0.684	0.831	0.844
629	0.842	0.767	0.804	0.610	0.783	0.811
630	0.833	0.808	0.821	0.642	0.813	0.823
631	0.833	0.858	0.846	0.692	0.855	0.844
632	0.833	0.825	0.829	0.658	0.826	0.830
633	0.825	0.825	0.825	0.650	0.825	0.825
634	0.858	0.833	0.846	0.692	0.837	0.848
635	0.858	0.833	0.846	0.692	0.837	0.848
636	0.850	0.792	0.821	0.643	0.803	0.826
637	0.850	0.825	0.838	0.675	0.829	0.840
638	0.850	0.825	0.838	0.675	0.829	0.840

639	0.858	0.825	0.842	0.684	0.831	0.844
640	0.858	0.792	0.825	0.651	0.805	0.831
641	0.850	0.758	0.804	0.611	0.779	0.813
642	0.842	0.825	0.833	0.667	0.828	0.835
643	0.850	0.825	0.838	0.675	0.829	0.840
644	0.875	0.833	0.854	0.709	0.840	0.857
645	0.825	0.800	0.813	0.625	0.805	0.815
646	0.808	0.808	0.808	0.617	0.808	0.808
647	0.842	0.817	0.829	0.659	0.821	0.831
648	0.875	0.792	0.833	0.669	0.808	0.840
649	0.867	0.808	0.838	0.676	0.819	0.842
650	0.867	0.833	0.850	0.700	0.839	0.852
651	0.825	0.783	0.804	0.609	0.792	0.808
652	0.817	0.792	0.804	0.609	0.797	0.807
653	0.850	0.817	0.833	0.667	0.823	0.836
654	0.833	0.817	0.825	0.650	0.820	0.826
655	0.867	0.825	0.846	0.692	0.832	0.849
656	0.875	0.792	0.833	0.669	0.808	0.840
657	0.867	0.842	0.854	0.709	0.846	0.856
658	0.833	0.850	0.842	0.683	0.847	0.840
659	0.833	0.817	0.825	0.650	0.820	0.826
660	0.808	0.800	0.804	0.608	0.802	0.805
661	0.833	0.750	0.792	0.585	0.769	0.800
662	0.858	0.833	0.846	0.692	0.837	0.848
663	0.825	0.825	0.825	0.650	0.825	0.825
664	0.858	0.833	0.846	0.692	0.837	0.848
665	0.817	0.817	0.817	0.633	0.817	0.817
666	0.825	0.850	0.838	0.675	0.846	0.835
667	0.833	0.800	0.817	0.634	0.806	0.820
668	0.825	0.775	0.800	0.601	0.786	0.805
669	0.842	0.808	0.825	0.650	0.815	0.828
670	0.842	0.833	0.838	0.675	0.835	0.838
671	0.833	0.833	0.833	0.667	0.833	0.833
672	0.833	0.825	0.829	0.658	0.826	0.830
673	0.792	0.800	0.796	0.592	0.798	0.795
674	0.850	0.808	0.829	0.659	0.816	0.833
675	0.817	0.792	0.804	0.609	0.797	0.807
676	0.825	0.817	0.821	0.642	0.818	0.822
677	0.817	0.792	0.804	0.609	0.797	0.807
678	0.858	0.792	0.825	0.651	0.805	0.831
679	0.850	0.825	0.838	0.675	0.829	0.840
680	0.833	0.817	0.825	0.650	0.820	0.826

681	0.825	0.817	0.821	0.642	0.818	0.822
682	0.858	0.800	0.829	0.659	0.811	0.834
683	0.850	0.800	0.825	0.651	0.810	0.829
684	0.833	0.808	0.821	0.642	0.813	0.823
685	0.842	0.817	0.829	0.659	0.821	0.831
686	0.850	0.842	0.846	0.692	0.843	0.846
687	0.825	0.792	0.808	0.617	0.798	0.811
688	0.842	0.817	0.829	0.659	0.821	0.831
689	0.817	0.825	0.821	0.642	0.824	0.820
690	0.808	0.767	0.788	0.576	0.776	0.792
691	0.858	0.800	0.829	0.659	0.811	0.834
692	0.833	0.792	0.813	0.626	0.800	0.816
693	0.808	0.817	0.813	0.625	0.815	0.812
694	0.850	0.767	0.808	0.619	0.785	0.816
695	0.858	0.833	0.846	0.692	0.837	0.848
696	0.817	0.783	0.800	0.600	0.790	0.803
697	0.842	0.825	0.833	0.667	0.828	0.835
698	0.825	0.800	0.813	0.625	0.805	0.815
699	0.833	0.808	0.821	0.642	0.813	0.823
700	0.833	0.775	0.804	0.609	0.787	0.810
701	0.842	0.817	0.829	0.659	0.821	0.831
702	0.858	0.800	0.829	0.659	0.811	0.834
703	0.867	0.833	0.850	0.700	0.839	0.852
704	0.825	0.800	0.813	0.625	0.805	0.815
705	0.825	0.833	0.829	0.658	0.832	0.828
706	0.833	0.792	0.813	0.626	0.800	0.816
707	0.850	0.792	0.821	0.643	0.803	0.826
708	0.833	0.817	0.825	0.650	0.820	0.826
709	0.867	0.833	0.850	0.700	0.839	0.852
710	0.858	0.808	0.833	0.668	0.817	0.837
711	0.808	0.825	0.817	0.633	0.822	0.815
712	0.825	0.775	0.800	0.601	0.786	0.805
713	0.817	0.800	0.808	0.617	0.803	0.810
714	0.833	0.808	0.821	0.642	0.813	0.823
715	0.858	0.842	0.850	0.700	0.844	0.851
716	0.867	0.825	0.846	0.692	0.832	0.849
717	0.858	0.767	0.813	0.628	0.786	0.821
718	0.858	0.858	0.858	0.717	0.858	0.858
719	0.817	0.817	0.817	0.633	0.817	0.817
720	0.850	0.817	0.833	0.667	0.823	0.836
721	0.858	0.817	0.838	0.676	0.824	0.841
722	0.808	0.842	0.825	0.650	0.836	0.822

723	0.825	0.817	0.821	0.642	0.818	0.822
724	0.833	0.792	0.813	0.626	0.800	0.816
725	0.842	0.833	0.838	0.675	0.835	0.838
726	0.858	0.850	0.854	0.708	0.851	0.855
727	0.800	0.808	0.804	0.608	0.807	0.803
728	0.825	0.867	0.846	0.692	0.861	0.843
729	0.875	0.842	0.858	0.717	0.847	0.861
730	0.792	0.833	0.813	0.626	0.826	0.809
731	0.817	0.817	0.817	0.633	0.817	0.817
732	0.833	0.775	0.804	0.609	0.787	0.810
733	0.858	0.825	0.842	0.684	0.831	0.844
734	0.867	0.842	0.854	0.709	0.846	0.856
735	0.833	0.817	0.825	0.650	0.820	0.826
736	0.850	0.792	0.821	0.643	0.803	0.826
737	0.850	0.792	0.821	0.643	0.803	0.826
738	0.825	0.792	0.808	0.617	0.798	0.811
739	0.825	0.825	0.825	0.650	0.825	0.825
740	0.792	0.800	0.796	0.592	0.798	0.795
741	0.825	0.833	0.829	0.658	0.832	0.828
742	0.833	0.833	0.833	0.667	0.833	0.833
743	0.800	0.817	0.808	0.617	0.814	0.807
744	0.817	0.833	0.825	0.650	0.831	0.824
745	0.833	0.858	0.846	0.692	0.855	0.844
746	0.825	0.817	0.821	0.642	0.818	0.822
747	0.850	0.775	0.813	0.627	0.791	0.819
748	0.858	0.817	0.838	0.676	0.824	0.841
749	0.800	0.875	0.838	0.677	0.865	0.831
750	0.858	0.800	0.829	0.659	0.811	0.834
751	0.842	0.808	0.825	0.650	0.815	0.828
752	0.850	0.775	0.813	0.627	0.791	0.819
753	0.850	0.808	0.829	0.659	0.816	0.833
754	0.842	0.800	0.821	0.642	0.808	0.824
755	0.833	0.775	0.804	0.609	0.787	0.810
756	0.833	0.825	0.829	0.658	0.826	0.830
757	0.842	0.792	0.817	0.634	0.802	0.821
758	0.800	0.825	0.813	0.625	0.821	0.810
759	0.833	0.800	0.817	0.634	0.806	0.820
760	0.867	0.792	0.829	0.660	0.806	0.835
761	0.850	0.833	0.842	0.683	0.836	0.843
762	0.833	0.817	0.825	0.650	0.820	0.826
763	0.825	0.825	0.825	0.650	0.825	0.825
764	0.850	0.808	0.829	0.659	0.816	0.833

765	0.842	0.825	0.833	0.667	0.828	0.835
766	0.808	0.808	0.808	0.617	0.808	0.808
767	0.817	0.825	0.821	0.642	0.824	0.820
768	0.817	0.792	0.804	0.609	0.797	0.807
769	0.875	0.808	0.842	0.685	0.820	0.847
770	0.792	0.792	0.792	0.583	0.792	0.792
771	0.825	0.808	0.817	0.633	0.811	0.818
772	0.825	0.817	0.821	0.642	0.818	0.822
773	0.850	0.842	0.846	0.692	0.843	0.846
774	0.850	0.808	0.829	0.659	0.816	0.833
775	0.808	0.817	0.813	0.625	0.815	0.812
776	0.800	0.808	0.804	0.608	0.807	0.803
777	0.825	0.775	0.800	0.601	0.786	0.805
778	0.825	0.850	0.838	0.675	0.846	0.835
779	0.833	0.775	0.804	0.609	0.787	0.810
780	0.825	0.817	0.821	0.642	0.818	0.822
781	0.792	0.767	0.779	0.559	0.772	0.782
782	0.842	0.833	0.838	0.675	0.835	0.838
783	0.850	0.833	0.842	0.683	0.836	0.843
784	0.833	0.767	0.800	0.601	0.781	0.806
785	0.808	0.858	0.833	0.668	0.851	0.829
786	0.808	0.758	0.783	0.567	0.770	0.789
787	0.833	0.833	0.833	0.667	0.833	0.833
788	0.833	0.800	0.817	0.634	0.806	0.820
789	0.867	0.750	0.808	0.621	0.776	0.819
790	0.850	0.792	0.821	0.643	0.803	0.826
791	0.792	0.808	0.800	0.600	0.805	0.798
792	0.833	0.817	0.825	0.650	0.820	0.826
793	0.850	0.800	0.825	0.651	0.810	0.829
794	0.833	0.800	0.817	0.634	0.806	0.820
795	0.808	0.875	0.842	0.685	0.866	0.836
796	0.783	0.800	0.792	0.583	0.797	0.790
797	0.842	0.817	0.829	0.659	0.821	0.831
798	0.808	0.783	0.796	0.592	0.789	0.798
799	0.850	0.817	0.833	0.667	0.823	0.836
800	0.817	0.808	0.813	0.625	0.810	0.813
801	0.817	0.850	0.833	0.667	0.845	0.831
802	0.833	0.825	0.829	0.658	0.826	0.830
803	0.850	0.783	0.817	0.635	0.797	0.823
804	0.867	0.808	0.838	0.676	0.819	0.842
805	0.850	0.733	0.792	0.587	0.761	0.803
806	0.817	0.825	0.821	0.642	0.824	0.820

807	0.833	0.833	0.833	0.667	0.833	0.833
808	0.825	0.808	0.817	0.633	0.811	0.818
809	0.808	0.800	0.804	0.608	0.802	0.805
810	0.833	0.817	0.825	0.650	0.820	0.826
811	0.817	0.817	0.817	0.633	0.817	0.817
812	0.842	0.817	0.829	0.659	0.821	0.831
813	0.825	0.800	0.813	0.625	0.805	0.815
814	0.850	0.825	0.838	0.675	0.829	0.840
815	0.817	0.792	0.804	0.609	0.797	0.807
816	0.833	0.808	0.821	0.642	0.813	0.823
817	0.850	0.842	0.846	0.692	0.843	0.846
818	0.825	0.783	0.804	0.609	0.792	0.808
819	0.833	0.850	0.842	0.683	0.847	0.840
820	0.842	0.792	0.817	0.634	0.802	0.821
821	0.858	0.817	0.838	0.676	0.824	0.841
822	0.800	0.842	0.821	0.642	0.835	0.817
823	0.833	0.817	0.825	0.650	0.820	0.826
824	0.808	0.883	0.846	0.694	0.874	0.840
825	0.817	0.817	0.817	0.633	0.817	0.817
826	0.825	0.808	0.817	0.633	0.811	0.818
827	0.858	0.850	0.854	0.708	0.851	0.855
828	0.825	0.817	0.821	0.642	0.818	0.822
829	0.792	0.833	0.813	0.626	0.826	0.809
830	0.833	0.775	0.804	0.609	0.787	0.810
831	0.833	0.817	0.825	0.650	0.820	0.826
832	0.833	0.783	0.808	0.617	0.794	0.813
833	0.883	0.833	0.858	0.718	0.841	0.862
834	0.842	0.792	0.817	0.634	0.802	0.821
835	0.833	0.842	0.838	0.675	0.840	0.837
836	0.842	0.783	0.813	0.626	0.795	0.818
837	0.808	0.825	0.817	0.633	0.822	0.815
838	0.850	0.783	0.817	0.635	0.797	0.823
839	0.792	0.808	0.800	0.600	0.805	0.798
840	0.850	0.817	0.833	0.667	0.823	0.836
841	0.850	0.783	0.817	0.635	0.797	0.823
842	0.808	0.808	0.808	0.617	0.808	0.808
843	0.817	0.783	0.800	0.600	0.790	0.803
844	0.858	0.792	0.825	0.651	0.805	0.831
845	0.833	0.792	0.813	0.626	0.800	0.816
846	0.817	0.800	0.808	0.617	0.803	0.810
847	0.825	0.842	0.833	0.667	0.839	0.832
848	0.842	0.817	0.829	0.659	0.821	0.831

849	0.833	0.792	0.813	0.626	0.800	0.816
850	0.858	0.817	0.838	0.676	0.824	0.841
851	0.833	0.783	0.808	0.617	0.794	0.813
852	0.858	0.792	0.825	0.651	0.805	0.831
853	0.783	0.792	0.788	0.575	0.790	0.787
854	0.792	0.808	0.800	0.600	0.805	0.798
855	0.817	0.783	0.800	0.600	0.790	0.803
856	0.850	0.792	0.821	0.643	0.803	0.826
857	0.850	0.775	0.813	0.627	0.791	0.819
858	0.858	0.833	0.846	0.692	0.837	0.848
859	0.833	0.792	0.813	0.626	0.800	0.816
860	0.850	0.783	0.817	0.635	0.797	0.823
861	0.842	0.817	0.829	0.659	0.821	0.831
862	0.825	0.817	0.821	0.642	0.818	0.822
863	0.858	0.792	0.825	0.651	0.805	0.831
864	0.833	0.792	0.813	0.626	0.800	0.816
865	0.808	0.817	0.813	0.625	0.815	0.812
866	0.817	0.800	0.808	0.617	0.803	0.810
867	0.850	0.817	0.833	0.667	0.823	0.836
868	0.833	0.808	0.821	0.642	0.813	0.823
869	0.883	0.800	0.842	0.686	0.815	0.848
870	0.808	0.817	0.813	0.625	0.815	0.812
871	0.808	0.817	0.813	0.625	0.815	0.812
872	0.808	0.808	0.808	0.617	0.808	0.808
873	0.842	0.808	0.825	0.650	0.815	0.828
874	0.833	0.858	0.846	0.692	0.855	0.844
875	0.850	0.800	0.825	0.651	0.810	0.829
876	0.808	0.833	0.821	0.642	0.829	0.819
877	0.800	0.783	0.792	0.583	0.787	0.793
878	0.817	0.808	0.813	0.625	0.810	0.813
879	0.850	0.808	0.829	0.659	0.816	0.833
880	0.850	0.783	0.817	0.635	0.797	0.823
881	0.833	0.800	0.817	0.634	0.806	0.820
882	0.858	0.800	0.829	0.659	0.811	0.834
883	0.833	0.825	0.829	0.658	0.826	0.830
884	0.842	0.800	0.821	0.642	0.808	0.824
885	0.808	0.833	0.821	0.642	0.829	0.819
886	0.825	0.775	0.800	0.601	0.786	0.805
887	0.842	0.817	0.829	0.659	0.821	0.831
888	0.850	0.800	0.825	0.651	0.810	0.829
889	0.833	0.792	0.813	0.626	0.800	0.816
890	0.783	0.800	0.792	0.583	0.797	0.790

891	0.867	0.817	0.842	0.684	0.825	0.846
892	0.858	0.833	0.846	0.692	0.837	0.848
893	0.817	0.767	0.792	0.584	0.778	0.797
894	0.850	0.767	0.808	0.619	0.785	0.816
895	0.842	0.842	0.842	0.683	0.842	0.842
896	0.825	0.792	0.808	0.617	0.798	0.811
897	0.808	0.833	0.821	0.642	0.829	0.819
898	0.833	0.817	0.825	0.650	0.820	0.826
899	0.808	0.800	0.804	0.608	0.802	0.805
900	0.817	0.808	0.813	0.625	0.810	0.813
901	0.792	0.817	0.804	0.609	0.812	0.802
902	0.842	0.833	0.838	0.675	0.835	0.838
903	0.833	0.808	0.821	0.642	0.813	0.823
904	0.825	0.783	0.804	0.609	0.792	0.808
905	0.833	0.792	0.813	0.626	0.800	0.816
906	0.867	0.808	0.838	0.676	0.819	0.842
907	0.842	0.792	0.817	0.634	0.802	0.821
908	0.825	0.833	0.829	0.658	0.832	0.828
909	0.800	0.808	0.804	0.608	0.807	0.803
910	0.817	0.842	0.829	0.659	0.838	0.827
911	0.833	0.808	0.821	0.642	0.813	0.823
912	0.808	0.783	0.796	0.592	0.789	0.798
913	0.833	0.825	0.829	0.658	0.826	0.830
914	0.808	0.792	0.800	0.600	0.795	0.802
915	0.833	0.858	0.846	0.692	0.855	0.844
916	0.842	0.800	0.821	0.642	0.808	0.824
917	0.875	0.808	0.842	0.685	0.820	0.847
918	0.867	0.808	0.838	0.676	0.819	0.842
919	0.825	0.833	0.829	0.658	0.832	0.828
920	0.833	0.825	0.829	0.658	0.826	0.830
921	0.817	0.808	0.813	0.625	0.810	0.813
922	0.850	0.842	0.846	0.692	0.843	0.846
923	0.825	0.792	0.808	0.617	0.798	0.811
924	0.842	0.775	0.808	0.618	0.789	0.815
925	0.858	0.808	0.833	0.668	0.817	0.837
926	0.858	0.808	0.833	0.668	0.817	0.837
927	0.808	0.808	0.808	0.617	0.808	0.808
928	0.825	0.800	0.813	0.625	0.805	0.815
929	0.800	0.808	0.804	0.608	0.807	0.803
930	0.817	0.825	0.821	0.642	0.824	0.820
931	0.842	0.817	0.829	0.659	0.821	0.831
932	0.833	0.817	0.825	0.650	0.820	0.826

933	0.850	0.792	0.821	0.643	0.803	0.826
934	0.825	0.775	0.800	0.601	0.786	0.805
935	0.842	0.783	0.813	0.626	0.795	0.818
936	0.817	0.808	0.813	0.625	0.810	0.813
937	0.825	0.783	0.804	0.609	0.792	0.808
938	0.842	0.833	0.838	0.675	0.835	0.838
939	0.800	0.783	0.792	0.583	0.787	0.793
940	0.817	0.800	0.808	0.617	0.803	0.810
941	0.850	0.758	0.804	0.611	0.779	0.813
942	0.808	0.825	0.817	0.633	0.822	0.815
943	0.842	0.808	0.825	0.650	0.815	0.828
944	0.825	0.850	0.838	0.675	0.846	0.835
945	0.833	0.817	0.825	0.650	0.820	0.826
946	0.833	0.817	0.825	0.650	0.820	0.826
947	0.858	0.775	0.817	0.636	0.792	0.824
948	0.808	0.800	0.804	0.608	0.802	0.805
949	0.825	0.825	0.825	0.650	0.825	0.825
950	0.867	0.817	0.842	0.684	0.825	0.846
951	0.842	0.867	0.854	0.709	0.863	0.852
952	0.792	0.792	0.792	0.583	0.792	0.792
953	0.808	0.800	0.804	0.608	0.802	0.805
954	0.842	0.825	0.833	0.667	0.828	0.835
955	0.833	0.800	0.817	0.634	0.806	0.820
956	0.792	0.783	0.788	0.575	0.785	0.788
957	0.867	0.783	0.825	0.652	0.800	0.832
958	0.858	0.817	0.838	0.676	0.824	0.841
959	0.817	0.825	0.821	0.642	0.824	0.820
960	0.825	0.800	0.813	0.625	0.805	0.815
961	0.825	0.783	0.804	0.609	0.792	0.808
962	0.817	0.808	0.813	0.625	0.810	0.813
963	0.842	0.808	0.825	0.650	0.815	0.828
964	0.800	0.808	0.804	0.608	0.807	0.803
965	0.833	0.783	0.808	0.617	0.794	0.813
966	0.825	0.808	0.817	0.633	0.811	0.818
967	0.817	0.767	0.792	0.584	0.778	0.797
968	0.817	0.833	0.825	0.650	0.831	0.824
969	0.808	0.775	0.792	0.584	0.782	0.795
970	0.833	0.825	0.829	0.658	0.826	0.830
971	0.867	0.833	0.850	0.700	0.839	0.852
972	0.833	0.808	0.821	0.642	0.813	0.823
973	0.850	0.783	0.817	0.635	0.797	0.823
974	0.858	0.817	0.838	0.676	0.824	0.841

975	0.833	0.825	0.829	0.658	0.826	0.830
976	0.817	0.800	0.808	0.617	0.803	0.810
977	0.817	0.783	0.800	0.600	0.790	0.803
978	0.833	0.750	0.792	0.585	0.769	0.800
979	0.808	0.792	0.800	0.600	0.795	0.802
980	0.817	0.817	0.817	0.633	0.817	0.817
981	0.833	0.808	0.821	0.642	0.813	0.823
982	0.825	0.767	0.796	0.593	0.780	0.802
983	0.850	0.792	0.821	0.643	0.803	0.826
984	0.808	0.833	0.821	0.642	0.829	0.819
985	0.792	0.825	0.808	0.617	0.819	0.805
986	0.808	0.808	0.808	0.617	0.808	0.808
987	0.808	0.783	0.796	0.592	0.789	0.798
988	0.825	0.817	0.821	0.642	0.818	0.822
989	0.808	0.850	0.829	0.659	0.843	0.826
990	0.817	0.800	0.808	0.617	0.803	0.810
991	0.817	0.767	0.792	0.584	0.778	0.797
992	0.858	0.775	0.817	0.636	0.792	0.824
993	0.783	0.817	0.800	0.600	0.810	0.797
994	0.833	0.783	0.808	0.617	0.794	0.813
995	0.825	0.850	0.838	0.675	0.846	0.835
996	0.842	0.775	0.808	0.618	0.789	0.815
997	0.833	0.833	0.833	0.667	0.833	0.833
998	0.808	0.825	0.817	0.633	0.822	0.815
999	0.842	0.775	0.808	0.618	0.789	0.815
1000	0.825	0.792	0.808	0.617	0.798	0.811
1001	0.792	0.850	0.821	0.643	0.841	0.815
1002	0.842	0.825	0.833	0.667	0.828	0.835
1003	0.825	0.817	0.821	0.642	0.818	0.822
1004	0.850	0.825	0.838	0.675	0.829	0.840
1005	0.850	0.792	0.821	0.643	0.803	0.826
1006	0.842	0.858	0.850	0.700	0.856	0.849
1007	0.825	0.817	0.821	0.642	0.818	0.822
1008	0.800	0.850	0.825	0.651	0.842	0.821
1009	0.808	0.767	0.788	0.576	0.776	0.792
1010	0.817	0.808	0.813	0.625	0.810	0.813
1011	0.792	0.792	0.792	0.583	0.792	0.792
1012	0.808	0.800	0.804	0.608	0.802	0.805
1013	0.825	0.767	0.796	0.593	0.780	0.802
1014	0.842	0.792	0.817	0.634	0.802	0.821
1015	0.808	0.800	0.804	0.608	0.802	0.805
1016	0.792	0.842	0.817	0.634	0.833	0.812

1017	0.808	0.858	0.833	0.668	0.851	0.829
1018	0.833	0.800	0.817	0.634	0.806	0.820
1019	0.850	0.800	0.825	0.651	0.810	0.829
1020	0.825	0.833	0.829	0.658	0.832	0.828

(4) Support vector machine

Number of features	SN	SP	ACC	MCC	Precision	F1-measure
1	0.642	0.733	0.688	0.377	0.706	0.672
2	0.742	0.683	0.713	0.426	0.701	0.721
3	0.708	0.792	0.750	0.502	0.773	0.739
4	0.758	0.775	0.767	0.533	0.771	0.765
5	0.783	0.825	0.804	0.609	0.817	0.800
6	0.817	0.875	0.846	0.693	0.867	0.841
7	0.808	0.875	0.842	0.685	0.866	0.836
8	0.817	0.892	0.854	0.710	0.883	0.848
9	0.808	0.883	0.846	0.694	0.874	0.840
10	0.800	0.883	0.842	0.686	0.873	0.835
11	0.825	0.875	0.850	0.701	0.868	0.846
12	0.800	0.858	0.829	0.659	0.850	0.824
13	0.800	0.858	0.829	0.659	0.850	0.824
14	0.808	0.867	0.838	0.676	0.858	0.833
15	0.825	0.850	0.838	0.675	0.846	0.835
16	0.842	0.858	0.850	0.700	0.856	0.849
17	0.833	0.850	0.842	0.683	0.847	0.840
18	0.842	0.867	0.854	0.709	0.863	0.852
19	0.842	0.858	0.850	0.700	0.856	0.849
20	0.833	0.858	0.846	0.692	0.855	0.844
21	0.833	0.875	0.854	0.709	0.870	0.851
22	0.833	0.867	0.850	0.700	0.862	0.847
23	0.825	0.867	0.846	0.692	0.861	0.843
24	0.800	0.883	0.842	0.686	0.873	0.835
25	0.800	0.867	0.833	0.668	0.857	0.828
26	0.808	0.867	0.838	0.676	0.858	0.833
27	0.800	0.867	0.833	0.668	0.857	0.828
28	0.817	0.867	0.842	0.684	0.860	0.838
29	0.808	0.867	0.838	0.676	0.858	0.833
30	0.800	0.883	0.842	0.686	0.873	0.835
31	0.808	0.875	0.842	0.685	0.866	0.836
32	0.808	0.867	0.838	0.676	0.858	0.833
33	0.825	0.875	0.850	0.701	0.868	0.846
34	0.817	0.875	0.846	0.693	0.867	0.841
35	0.817	0.883	0.850	0.702	0.875	0.845

36	0.808	0.883	0.846	0.694	0.874	0.840
37	0.800	0.900	0.850	0.704	0.889	0.842
38	0.800	0.900	0.850	0.704	0.889	0.842
39	0.800	0.917	0.858	0.722	0.906	0.850
40	0.808	0.917	0.863	0.729	0.907	0.855
41	0.808	0.900	0.854	0.711	0.890	0.847
42	0.808	0.900	0.854	0.711	0.890	0.847
43	0.808	0.892	0.850	0.702	0.882	0.843
44	0.825	0.900	0.863	0.727	0.892	0.857
45	0.817	0.892	0.854	0.710	0.883	0.848
46	0.817	0.908	0.863	0.728	0.899	0.856
47	0.808	0.900	0.854	0.711	0.890	0.847
48	0.808	0.900	0.854	0.711	0.890	0.847
49	0.817	0.883	0.850	0.702	0.875	0.845
50	0.808	0.883	0.846	0.694	0.874	0.840
51	0.808	0.875	0.842	0.685	0.866	0.836
52	0.808	0.900	0.854	0.711	0.890	0.847
53	0.808	0.900	0.854	0.711	0.890	0.847
54	0.817	0.900	0.858	0.719	0.891	0.852
55	0.817	0.892	0.854	0.710	0.883	0.848
56	0.808	0.900	0.854	0.711	0.890	0.847
57	0.808	0.892	0.850	0.702	0.882	0.843
58	0.808	0.892	0.850	0.702	0.882	0.843
59	0.808	0.908	0.858	0.720	0.898	0.851
60	0.792	0.917	0.854	0.714	0.905	0.844
61	0.808	0.900	0.854	0.711	0.890	0.847
62	0.817	0.900	0.858	0.719	0.891	0.852
63	0.800	0.900	0.850	0.704	0.889	0.842
64	0.800	0.900	0.850	0.704	0.889	0.842
65	0.800	0.900	0.850	0.704	0.889	0.842
66	0.800	0.892	0.846	0.695	0.881	0.838
67	0.808	0.900	0.854	0.711	0.890	0.847
68	0.808	0.900	0.854	0.711	0.890	0.847
69	0.800	0.892	0.846	0.695	0.881	0.838
70	0.808	0.917	0.863	0.729	0.907	0.855
71	0.808	0.917	0.863	0.729	0.907	0.855
72	0.808	0.925	0.867	0.738	0.915	0.858
73	0.800	0.908	0.854	0.713	0.897	0.846
74	0.800	0.925	0.863	0.731	0.914	0.853
75	0.808	0.925	0.867	0.738	0.915	0.858
76	0.808	0.933	0.871	0.748	0.924	0.862
77	0.800	0.933	0.867	0.740	0.923	0.857

78	0.800	0.933	0.867	0.740	0.923	0.857
79	0.800	0.933	0.867	0.740	0.923	0.857
80	0.800	0.933	0.867	0.740	0.923	0.857
81	0.800	0.933	0.867	0.740	0.923	0.857
82	0.800	0.933	0.867	0.740	0.923	0.857
83	0.800	0.933	0.867	0.740	0.923	0.857
84	0.808	0.942	0.875	0.757	0.933	0.866
85	0.808	0.942	0.875	0.757	0.933	0.866
86	0.808	0.950	0.879	0.766	0.942	0.870
87	0.800	0.950	0.875	0.759	0.941	0.865
88	0.800	0.950	0.875	0.759	0.941	0.865
89	0.800	0.950	0.875	0.759	0.941	0.865
90	0.800	0.950	0.875	0.759	0.941	0.865
91	0.792	0.942	0.867	0.742	0.931	0.856
92	0.800	0.950	0.875	0.759	0.941	0.865
93	0.800	0.950	0.875	0.759	0.941	0.865
94	0.800	0.958	0.879	0.768	0.950	0.869
95	0.800	0.958	0.879	0.768	0.950	0.869
96	0.800	0.958	0.879	0.768	0.950	0.869
97	0.800	0.958	0.879	0.768	0.950	0.869
98	0.808	0.933	0.871	0.748	0.924	0.862
99	0.808	0.933	0.871	0.748	0.924	0.862
100	0.800	0.942	0.871	0.749	0.932	0.861
101	0.817	0.942	0.879	0.764	0.933	0.871
102	0.817	0.942	0.879	0.764	0.933	0.871
103	0.817	0.942	0.879	0.764	0.933	0.871
104	0.817	0.942	0.879	0.764	0.933	0.871
105	0.817	0.942	0.879	0.764	0.933	0.871
106	0.817	0.933	0.875	0.755	0.925	0.867
107	0.817	0.933	0.875	0.755	0.925	0.867
108	0.817	0.942	0.879	0.764	0.933	0.871
109	0.825	0.942	0.883	0.772	0.934	0.876
110	0.825	0.942	0.883	0.772	0.934	0.876
111	0.825	0.942	0.883	0.772	0.934	0.876
112	0.825	0.950	0.888	0.781	0.943	0.880
113	0.825	0.950	0.888	0.781	0.943	0.880
114	0.825	0.958	0.892	0.790	0.952	0.884
115	0.825	0.950	0.888	0.781	0.943	0.880
116	0.825	0.933	0.879	0.763	0.925	0.872
117	0.825	0.933	0.879	0.763	0.925	0.872
118	0.825	0.933	0.879	0.763	0.925	0.872
119	0.825	0.925	0.875	0.754	0.917	0.868

120	0.817	0.933	0.875	0.755	0.925	0.867
121	0.817	0.933	0.875	0.755	0.925	0.867
122	0.825	0.950	0.888	0.781	0.943	0.880
123	0.825	0.958	0.892	0.790	0.952	0.884
124	0.825	0.950	0.888	0.781	0.943	0.880
125	0.825	0.933	0.879	0.763	0.925	0.872
126	0.825	0.933	0.879	0.763	0.925	0.872
127	0.825	0.942	0.883	0.772	0.934	0.876
128	0.825	0.942	0.883	0.772	0.934	0.876
129	0.817	0.942	0.879	0.764	0.933	0.871
130	0.817	0.933	0.875	0.755	0.925	0.867
131	0.842	0.933	0.888	0.778	0.927	0.882
132	0.833	0.942	0.888	0.780	0.935	0.881
133	0.833	0.942	0.888	0.780	0.935	0.881
134	0.833	0.942	0.888	0.780	0.935	0.881
135	0.833	0.933	0.883	0.771	0.926	0.877
136	0.833	0.933	0.883	0.771	0.926	0.877
137	0.833	0.933	0.883	0.771	0.926	0.877
138	0.833	0.942	0.888	0.780	0.935	0.881
139	0.833	0.933	0.883	0.771	0.926	0.877
140	0.833	0.933	0.883	0.771	0.926	0.877
141	0.825	0.942	0.883	0.772	0.934	0.876
142	0.825	0.950	0.888	0.781	0.943	0.880
143	0.833	0.933	0.883	0.771	0.926	0.877
144	0.833	0.933	0.883	0.771	0.926	0.877
145	0.833	0.933	0.883	0.771	0.926	0.877
146	0.833	0.925	0.879	0.762	0.917	0.873
147	0.833	0.925	0.879	0.762	0.917	0.873
148	0.833	0.933	0.883	0.771	0.926	0.877
149	0.833	0.933	0.883	0.771	0.926	0.877
150	0.833	0.933	0.883	0.771	0.926	0.877
151	0.833	0.933	0.883	0.771	0.926	0.877
152	0.833	0.933	0.883	0.771	0.926	0.877
153	0.833	0.933	0.883	0.771	0.926	0.877
154	0.833	0.942	0.888	0.780	0.935	0.881
155	0.833	0.925	0.879	0.762	0.917	0.873
156	0.833	0.933	0.883	0.771	0.926	0.877
157	0.833	0.933	0.883	0.771	0.926	0.877
158	0.833	0.933	0.883	0.771	0.926	0.877
159	0.833	0.942	0.888	0.780	0.935	0.881
160	0.833	0.933	0.883	0.771	0.926	0.877
161	0.833	0.933	0.883	0.771	0.926	0.877

162	0.833	0.917	0.875	0.753	0.909	0.870
163	0.833	0.933	0.883	0.771	0.926	0.877
164	0.833	0.933	0.883	0.771	0.926	0.877
165	0.833	0.933	0.883	0.771	0.926	0.877
166	0.833	0.942	0.888	0.780	0.935	0.881
167	0.833	0.942	0.888	0.780	0.935	0.881
168	0.833	0.942	0.888	0.780	0.935	0.881
169	0.833	0.933	0.883	0.771	0.926	0.877
170	0.833	0.942	0.888	0.780	0.935	0.881
171	0.833	0.925	0.879	0.762	0.917	0.873
172	0.833	0.925	0.879	0.762	0.917	0.873
173	0.833	0.925	0.879	0.762	0.917	0.873
174	0.833	0.933	0.883	0.771	0.926	0.877
175	0.833	0.942	0.888	0.780	0.935	0.881
176	0.833	0.942	0.888	0.780	0.935	0.881
177	0.833	0.942	0.888	0.780	0.935	0.881
178	0.833	0.942	0.888	0.780	0.935	0.881
179	0.833	0.942	0.888	0.780	0.935	0.881
180	0.825	0.942	0.883	0.772	0.934	0.876
181	0.825	0.942	0.883	0.772	0.934	0.876
182	0.825	0.942	0.883	0.772	0.934	0.876
183	0.825	0.942	0.883	0.772	0.934	0.876
184	0.825	0.942	0.883	0.772	0.934	0.876
185	0.825	0.942	0.883	0.772	0.934	0.876
186	0.825	0.942	0.883	0.772	0.934	0.876
187	0.825	0.942	0.883	0.772	0.934	0.876
188	0.833	0.933	0.883	0.771	0.926	0.877
189	0.825	0.942	0.883	0.772	0.934	0.876
190	0.825	0.942	0.883	0.772	0.934	0.876
191	0.825	0.942	0.883	0.772	0.934	0.876
192	0.825	0.942	0.883	0.772	0.934	0.876
193	0.825	0.942	0.883	0.772	0.934	0.876
194	0.825	0.933	0.879	0.763	0.925	0.872
195	0.825	0.942	0.883	0.772	0.934	0.876
196	0.825	0.933	0.879	0.763	0.925	0.872
197	0.825	0.933	0.879	0.763	0.925	0.872
198	0.825	0.925	0.875	0.754	0.917	0.868
199	0.825	0.917	0.871	0.745	0.908	0.865
200	0.825	0.917	0.871	0.745	0.908	0.865
201	0.825	0.917	0.871	0.745	0.908	0.865
202	0.825	0.917	0.871	0.745	0.908	0.865
203	0.825	0.917	0.871	0.745	0.908	0.865

204	0.825	0.925	0.875	0.754	0.917	0.868
205	0.825	0.925	0.875	0.754	0.917	0.868
206	0.825	0.925	0.875	0.754	0.917	0.868
207	0.825	0.925	0.875	0.754	0.917	0.868
208	0.825	0.925	0.875	0.754	0.917	0.868
209	0.825	0.925	0.875	0.754	0.917	0.868
210	0.825	0.925	0.875	0.754	0.917	0.868
211	0.825	0.917	0.871	0.745	0.908	0.865
212	0.825	0.917	0.871	0.745	0.908	0.865
213	0.825	0.925	0.875	0.754	0.917	0.868
214	0.825	0.925	0.875	0.754	0.917	0.868
215	0.825	0.925	0.875	0.754	0.917	0.868
216	0.825	0.925	0.875	0.754	0.917	0.868
217	0.825	0.925	0.875	0.754	0.917	0.868
218	0.825	0.925	0.875	0.754	0.917	0.868
219	0.825	0.925	0.875	0.754	0.917	0.868
220	0.817	0.925	0.871	0.746	0.916	0.863
221	0.817	0.925	0.871	0.746	0.916	0.863
222	0.817	0.925	0.871	0.746	0.916	0.863
223	0.817	0.925	0.871	0.746	0.916	0.863
224	0.817	0.925	0.871	0.746	0.916	0.863
225	0.817	0.917	0.867	0.737	0.907	0.860
226	0.817	0.917	0.867	0.737	0.907	0.860
227	0.817	0.925	0.871	0.746	0.916	0.863
228	0.817	0.917	0.867	0.737	0.907	0.860
229	0.817	0.917	0.867	0.737	0.907	0.860
230	0.817	0.917	0.867	0.737	0.907	0.860
231	0.817	0.917	0.867	0.737	0.907	0.860
232	0.817	0.908	0.863	0.728	0.899	0.856
233	0.817	0.908	0.863	0.728	0.899	0.856
234	0.825	0.908	0.867	0.736	0.900	0.861
235	0.825	0.917	0.871	0.745	0.908	0.865
236	0.825	0.908	0.867	0.736	0.900	0.861
237	0.825	0.925	0.875	0.754	0.917	0.868
238	0.825	0.917	0.871	0.745	0.908	0.865
239	0.825	0.925	0.875	0.754	0.917	0.868
240	0.825	0.925	0.875	0.754	0.917	0.868
241	0.825	0.925	0.875	0.754	0.917	0.868
242	0.825	0.925	0.875	0.754	0.917	0.868
243	0.825	0.925	0.875	0.754	0.917	0.868
244	0.825	0.925	0.875	0.754	0.917	0.868
245	0.825	0.942	0.883	0.772	0.934	0.876

246	0.825	0.933	0.879	0.763	0.925	0.872
247	0.825	0.933	0.879	0.763	0.925	0.872
248	0.825	0.933	0.879	0.763	0.925	0.872
249	0.825	0.933	0.879	0.763	0.925	0.872
250	0.808	0.925	0.867	0.738	0.915	0.858
251	0.808	0.925	0.867	0.738	0.915	0.858
252	0.817	0.925	0.871	0.746	0.916	0.863
253	0.817	0.925	0.871	0.746	0.916	0.863
254	0.817	0.925	0.871	0.746	0.916	0.863
255	0.817	0.925	0.871	0.746	0.916	0.863
256	0.817	0.925	0.871	0.746	0.916	0.863
257	0.817	0.925	0.871	0.746	0.916	0.863
258	0.817	0.925	0.871	0.746	0.916	0.863
259	0.817	0.925	0.871	0.746	0.916	0.863
260	0.817	0.933	0.875	0.755	0.925	0.867
261	0.817	0.933	0.875	0.755	0.925	0.867
262	0.825	0.925	0.875	0.754	0.917	0.868
263	0.825	0.925	0.875	0.754	0.917	0.868
264	0.825	0.925	0.875	0.754	0.917	0.868
265	0.825	0.925	0.875	0.754	0.917	0.868
266	0.825	0.925	0.875	0.754	0.917	0.868
267	0.825	0.925	0.875	0.754	0.917	0.868
268	0.825	0.925	0.875	0.754	0.917	0.868
269	0.817	0.925	0.871	0.746	0.916	0.863
270	0.817	0.917	0.867	0.737	0.907	0.860
271	0.825	0.925	0.875	0.754	0.917	0.868
272	0.825	0.925	0.875	0.754	0.917	0.868
273	0.825	0.917	0.871	0.745	0.908	0.865
274	0.817	0.917	0.867	0.737	0.907	0.860
275	0.817	0.925	0.871	0.746	0.916	0.863
276	0.817	0.925	0.871	0.746	0.916	0.863
277	0.817	0.925	0.871	0.746	0.916	0.863
278	0.817	0.925	0.871	0.746	0.916	0.863
279	0.825	0.925	0.875	0.754	0.917	0.868
280	0.825	0.925	0.875	0.754	0.917	0.868
281	0.825	0.917	0.871	0.745	0.908	0.865
282	0.825	0.900	0.863	0.727	0.892	0.857
283	0.833	0.892	0.863	0.726	0.885	0.858
284	0.825	0.892	0.858	0.718	0.884	0.853
285	0.825	0.900	0.863	0.727	0.892	0.857
286	0.817	0.892	0.854	0.710	0.883	0.848
287	0.817	0.900	0.858	0.719	0.891	0.852

288	0.817	0.908	0.863	0.728	0.899	0.856
289	0.817	0.908	0.863	0.728	0.899	0.856
290	0.825	0.917	0.871	0.745	0.908	0.865
291	0.825	0.908	0.867	0.736	0.900	0.861
292	0.825	0.908	0.867	0.736	0.900	0.861
293	0.825	0.908	0.867	0.736	0.900	0.861
294	0.825	0.908	0.867	0.736	0.900	0.861
295	0.825	0.917	0.871	0.745	0.908	0.865
296	0.825	0.917	0.871	0.745	0.908	0.865
297	0.825	0.917	0.871	0.745	0.908	0.865
298	0.825	0.917	0.871	0.745	0.908	0.865
299	0.817	0.917	0.867	0.737	0.907	0.860
300	0.817	0.917	0.867	0.737	0.907	0.860
301	0.825	0.917	0.871	0.745	0.908	0.865
302	0.825	0.917	0.871	0.745	0.908	0.865
303	0.825	0.917	0.871	0.745	0.908	0.865
304	0.825	0.917	0.871	0.745	0.908	0.865
305	0.825	0.917	0.871	0.745	0.908	0.865
306	0.825	0.917	0.871	0.745	0.908	0.865
307	0.825	0.917	0.871	0.745	0.908	0.865
308	0.825	0.917	0.871	0.745	0.908	0.865
309	0.825	0.883	0.854	0.710	0.876	0.850
310	0.817	0.883	0.850	0.702	0.875	0.845
311	0.817	0.883	0.850	0.702	0.875	0.845
312	0.817	0.892	0.854	0.710	0.883	0.848
313	0.817	0.900	0.858	0.719	0.891	0.852
314	0.817	0.900	0.858	0.719	0.891	0.852
315	0.817	0.900	0.858	0.719	0.891	0.852
316	0.817	0.900	0.858	0.719	0.891	0.852
317	0.825	0.900	0.863	0.727	0.892	0.857
318	0.817	0.892	0.854	0.710	0.883	0.848
319	0.817	0.892	0.854	0.710	0.883	0.848
320	0.817	0.892	0.854	0.710	0.883	0.848
321	0.817	0.900	0.858	0.719	0.891	0.852
322	0.817	0.900	0.858	0.719	0.891	0.852
323	0.817	0.900	0.858	0.719	0.891	0.852
324	0.817	0.892	0.854	0.710	0.883	0.848
325	0.817	0.892	0.854	0.710	0.883	0.848
326	0.817	0.892	0.854	0.710	0.883	0.848
327	0.817	0.892	0.854	0.710	0.883	0.848
328	0.817	0.892	0.854	0.710	0.883	0.848
329	0.817	0.892	0.854	0.710	0.883	0.848

330	0.817	0.892	0.854	0.710	0.883	0.848
331	0.817	0.892	0.854	0.710	0.883	0.848
332	0.833	0.900	0.867	0.735	0.893	0.862
333	0.833	0.900	0.867	0.735	0.893	0.862
334	0.833	0.900	0.867	0.735	0.893	0.862
335	0.833	0.908	0.871	0.744	0.901	0.866
336	0.833	0.908	0.871	0.744	0.901	0.866
337	0.833	0.908	0.871	0.744	0.901	0.866
338	0.825	0.900	0.863	0.727	0.892	0.857
339	0.825	0.892	0.858	0.718	0.884	0.853
340	0.825	0.892	0.858	0.718	0.884	0.853
341	0.825	0.908	0.867	0.736	0.900	0.861
342	0.825	0.908	0.867	0.736	0.900	0.861
343	0.825	0.900	0.863	0.727	0.892	0.857
344	0.817	0.908	0.863	0.728	0.899	0.856
345	0.817	0.908	0.863	0.728	0.899	0.856
346	0.817	0.908	0.863	0.728	0.899	0.856
347	0.825	0.908	0.867	0.736	0.900	0.861
348	0.825	0.908	0.867	0.736	0.900	0.861
349	0.825	0.917	0.871	0.745	0.908	0.865
350	0.817	0.917	0.867	0.737	0.907	0.860
351	0.817	0.925	0.871	0.746	0.916	0.863
352	0.817	0.925	0.871	0.746	0.916	0.863
353	0.825	0.925	0.875	0.754	0.917	0.868
354	0.817	0.925	0.871	0.746	0.916	0.863
355	0.817	0.925	0.871	0.746	0.916	0.863
356	0.817	0.925	0.871	0.746	0.916	0.863
357	0.825	0.917	0.871	0.745	0.908	0.865
358	0.825	0.917	0.871	0.745	0.908	0.865
359	0.825	0.917	0.871	0.745	0.908	0.865
360	0.825	0.917	0.871	0.745	0.908	0.865
361	0.825	0.917	0.871	0.745	0.908	0.865
362	0.825	0.917	0.871	0.745	0.908	0.865
363	0.817	0.917	0.867	0.737	0.907	0.860
364	0.817	0.917	0.867	0.737	0.907	0.860
365	0.817	0.917	0.867	0.737	0.907	0.860
366	0.817	0.917	0.867	0.737	0.907	0.860
367	0.817	0.917	0.867	0.737	0.907	0.860
368	0.817	0.917	0.867	0.737	0.907	0.860
369	0.817	0.917	0.867	0.737	0.907	0.860
370	0.817	0.908	0.863	0.728	0.899	0.856
371	0.825	0.908	0.867	0.736	0.900	0.861

372	0.817	0.908	0.863	0.728	0.899	0.856
373	0.825	0.908	0.867	0.736	0.900	0.861
374	0.817	0.917	0.867	0.737	0.907	0.860
375	0.817	0.917	0.867	0.737	0.907	0.860
376	0.817	0.917	0.867	0.737	0.907	0.860
377	0.825	0.917	0.871	0.745	0.908	0.865
378	0.825	0.917	0.871	0.745	0.908	0.865
379	0.833	0.917	0.875	0.753	0.909	0.870
380	0.833	0.917	0.875	0.753	0.909	0.870
381	0.833	0.917	0.875	0.753	0.909	0.870
382	0.833	0.917	0.875	0.753	0.909	0.870
383	0.833	0.917	0.875	0.753	0.909	0.870
384	0.825	0.917	0.871	0.745	0.908	0.865
385	0.825	0.917	0.871	0.745	0.908	0.865
386	0.833	0.917	0.875	0.753	0.909	0.870
387	0.833	0.917	0.875	0.753	0.909	0.870
388	0.833	0.908	0.871	0.744	0.901	0.866
389	0.825	0.917	0.871	0.745	0.908	0.865
390	0.825	0.908	0.867	0.736	0.900	0.861
391	0.825	0.908	0.867	0.736	0.900	0.861
392	0.825	0.917	0.871	0.745	0.908	0.865
393	0.825	0.917	0.871	0.745	0.908	0.865
394	0.825	0.917	0.871	0.745	0.908	0.865
395	0.825	0.908	0.867	0.736	0.900	0.861
396	0.825	0.908	0.867	0.736	0.900	0.861
397	0.825	0.908	0.867	0.736	0.900	0.861
398	0.825	0.908	0.867	0.736	0.900	0.861
399	0.825	0.917	0.871	0.745	0.908	0.865
400	0.825	0.908	0.867	0.736	0.900	0.861
401	0.825	0.908	0.867	0.736	0.900	0.861
402	0.825	0.908	0.867	0.736	0.900	0.861
403	0.825	0.908	0.867	0.736	0.900	0.861
404	0.825	0.908	0.867	0.736	0.900	0.861
405	0.825	0.908	0.867	0.736	0.900	0.861
406	0.825	0.908	0.867	0.736	0.900	0.861
407	0.825	0.908	0.867	0.736	0.900	0.861
408	0.817	0.908	0.863	0.728	0.899	0.856
409	0.817	0.908	0.863	0.728	0.899	0.856
410	0.817	0.908	0.863	0.728	0.899	0.856
411	0.817	0.908	0.863	0.728	0.899	0.856
412	0.817	0.908	0.863	0.728	0.899	0.856
413	0.817	0.908	0.863	0.728	0.899	0.856

414	0.817	0.908	0.863	0.728	0.899	0.856
415	0.817	0.908	0.863	0.728	0.899	0.856
416	0.817	0.908	0.863	0.728	0.899	0.856
417	0.817	0.900	0.858	0.719	0.891	0.852
418	0.817	0.900	0.858	0.719	0.891	0.852
419	0.817	0.900	0.858	0.719	0.891	0.852
420	0.817	0.900	0.858	0.719	0.891	0.852
421	0.817	0.900	0.858	0.719	0.891	0.852
422	0.817	0.908	0.863	0.728	0.899	0.856
423	0.825	0.908	0.867	0.736	0.900	0.861
424	0.825	0.900	0.863	0.727	0.892	0.857
425	0.825	0.908	0.867	0.736	0.900	0.861
426	0.817	0.908	0.863	0.728	0.899	0.856
427	0.817	0.908	0.863	0.728	0.899	0.856
428	0.825	0.908	0.867	0.736	0.900	0.861
429	0.825	0.908	0.867	0.736	0.900	0.861
430	0.825	0.917	0.871	0.745	0.908	0.865
431	0.825	0.917	0.871	0.745	0.908	0.865
432	0.825	0.917	0.871	0.745	0.908	0.865
433	0.825	0.917	0.871	0.745	0.908	0.865
434	0.825	0.917	0.871	0.745	0.908	0.865
435	0.825	0.917	0.871	0.745	0.908	0.865
436	0.825	0.917	0.871	0.745	0.908	0.865
437	0.825	0.917	0.871	0.745	0.908	0.865
438	0.825	0.917	0.871	0.745	0.908	0.865
439	0.825	0.908	0.867	0.736	0.900	0.861
440	0.825	0.908	0.867	0.736	0.900	0.861
441	0.825	0.908	0.867	0.736	0.900	0.861
442	0.825	0.908	0.867	0.736	0.900	0.861
443	0.825	0.908	0.867	0.736	0.900	0.861
444	0.825	0.908	0.867	0.736	0.900	0.861
445	0.825	0.908	0.867	0.736	0.900	0.861
446	0.825	0.908	0.867	0.736	0.900	0.861
447	0.825	0.908	0.867	0.736	0.900	0.861
448	0.825	0.908	0.867	0.736	0.900	0.861
449	0.825	0.908	0.867	0.736	0.900	0.861
450	0.825	0.908	0.867	0.736	0.900	0.861
451	0.825	0.908	0.867	0.736	0.900	0.861
452	0.825	0.908	0.867	0.736	0.900	0.861
453	0.817	0.908	0.863	0.728	0.899	0.856
454	0.817	0.908	0.863	0.728	0.899	0.856
455	0.817	0.908	0.863	0.728	0.899	0.856

456	0.825	0.908	0.867	0.736	0.900	0.861
457	0.825	0.908	0.867	0.736	0.900	0.861
458	0.825	0.908	0.867	0.736	0.900	0.861
459	0.825	0.908	0.867	0.736	0.900	0.861
460	0.825	0.900	0.863	0.727	0.892	0.857
461	0.825	0.900	0.863	0.727	0.892	0.857
462	0.817	0.917	0.867	0.737	0.907	0.860
463	0.817	0.917	0.867	0.737	0.907	0.860
464	0.817	0.917	0.867	0.737	0.907	0.860
465	0.817	0.908	0.863	0.728	0.899	0.856
466	0.817	0.908	0.863	0.728	0.899	0.856
467	0.817	0.908	0.863	0.728	0.899	0.856
468	0.817	0.908	0.863	0.728	0.899	0.856
469	0.817	0.900	0.858	0.719	0.891	0.852
470	0.825	0.917	0.871	0.745	0.908	0.865
471	0.817	0.925	0.871	0.746	0.916	0.863
472	0.817	0.925	0.871	0.746	0.916	0.863
473	0.817	0.925	0.871	0.746	0.916	0.863
474	0.817	0.925	0.871	0.746	0.916	0.863
475	0.817	0.908	0.863	0.728	0.899	0.856
476	0.817	0.908	0.863	0.728	0.899	0.856
477	0.817	0.908	0.863	0.728	0.899	0.856
478	0.817	0.908	0.863	0.728	0.899	0.856
479	0.817	0.908	0.863	0.728	0.899	0.856
480	0.817	0.917	0.867	0.737	0.907	0.860
481	0.817	0.917	0.867	0.737	0.907	0.860
482	0.817	0.917	0.867	0.737	0.907	0.860
483	0.817	0.917	0.867	0.737	0.907	0.860
484	0.817	0.917	0.867	0.737	0.907	0.860
485	0.817	0.917	0.867	0.737	0.907	0.860
486	0.817	0.917	0.867	0.737	0.907	0.860
487	0.817	0.917	0.867	0.737	0.907	0.860
488	0.817	0.917	0.867	0.737	0.907	0.860
489	0.817	0.917	0.867	0.737	0.907	0.860
490	0.817	0.917	0.867	0.737	0.907	0.860
491	0.817	0.917	0.867	0.737	0.907	0.860
492	0.817	0.917	0.867	0.737	0.907	0.860
493	0.817	0.917	0.867	0.737	0.907	0.860
494	0.817	0.917	0.867	0.737	0.907	0.860
495	0.817	0.917	0.867	0.737	0.907	0.860
496	0.817	0.908	0.863	0.728	0.899	0.856
497	0.817	0.917	0.867	0.737	0.907	0.860

498	0.817	0.917	0.867	0.737	0.907	0.860
499	0.817	0.917	0.867	0.737	0.907	0.860
500	0.817	0.908	0.863	0.728	0.899	0.856
501	0.817	0.908	0.863	0.728	0.899	0.856
502	0.817	0.908	0.863	0.728	0.899	0.856
503	0.817	0.908	0.863	0.728	0.899	0.856
504	0.817	0.900	0.858	0.719	0.891	0.852
505	0.817	0.908	0.863	0.728	0.899	0.856
506	0.817	0.900	0.858	0.719	0.891	0.852
507	0.817	0.900	0.858	0.719	0.891	0.852
508	0.817	0.908	0.863	0.728	0.899	0.856
509	0.817	0.908	0.863	0.728	0.899	0.856
510	0.817	0.908	0.863	0.728	0.899	0.856
511	0.817	0.900	0.858	0.719	0.891	0.852
512	0.817	0.908	0.863	0.728	0.899	0.856
513	0.817	0.900	0.858	0.719	0.891	0.852
514	0.817	0.900	0.858	0.719	0.891	0.852
515	0.817	0.900	0.858	0.719	0.891	0.852
516	0.817	0.908	0.863	0.728	0.899	0.856
517	0.817	0.908	0.863	0.728	0.899	0.856
518	0.817	0.908	0.863	0.728	0.899	0.856
519	0.817	0.900	0.858	0.719	0.891	0.852
520	0.817	0.900	0.858	0.719	0.891	0.852
521	0.817	0.900	0.858	0.719	0.891	0.852
522	0.817	0.892	0.854	0.710	0.883	0.848
523	0.817	0.892	0.854	0.710	0.883	0.848
524	0.825	0.900	0.863	0.727	0.892	0.857
525	0.825	0.900	0.863	0.727	0.892	0.857
526	0.825	0.900	0.863	0.727	0.892	0.857
527	0.825	0.900	0.863	0.727	0.892	0.857
528	0.825	0.900	0.863	0.727	0.892	0.857
529	0.825	0.900	0.863	0.727	0.892	0.857
530	0.825	0.900	0.863	0.727	0.892	0.857
531	0.825	0.900	0.863	0.727	0.892	0.857
532	0.825	0.900	0.863	0.727	0.892	0.857
533	0.825	0.892	0.858	0.718	0.884	0.853
534	0.825	0.900	0.863	0.727	0.892	0.857
535	0.825	0.900	0.863	0.727	0.892	0.857
536	0.825	0.900	0.863	0.727	0.892	0.857
537	0.825	0.900	0.863	0.727	0.892	0.857
538	0.825	0.908	0.867	0.736	0.900	0.861
539	0.825	0.908	0.867	0.736	0.900	0.861

540	0.825	0.908	0.867	0.736	0.900	0.861
541	0.825	0.908	0.867	0.736	0.900	0.861
542	0.825	0.908	0.867	0.736	0.900	0.861
543	0.825	0.908	0.867	0.736	0.900	0.861
544	0.825	0.908	0.867	0.736	0.900	0.861
545	0.825	0.908	0.867	0.736	0.900	0.861
546	0.825	0.892	0.858	0.718	0.884	0.853
547	0.825	0.892	0.858	0.718	0.884	0.853
548	0.825	0.900	0.863	0.727	0.892	0.857
549	0.825	0.892	0.858	0.718	0.884	0.853
550	0.825	0.892	0.858	0.718	0.884	0.853
551	0.825	0.900	0.863	0.727	0.892	0.857
552	0.825	0.892	0.858	0.718	0.884	0.853
553	0.825	0.892	0.858	0.718	0.884	0.853
554	0.825	0.900	0.863	0.727	0.892	0.857
555	0.825	0.892	0.858	0.718	0.884	0.853
556	0.825	0.892	0.858	0.718	0.884	0.853
557	0.817	0.892	0.854	0.710	0.883	0.848
558	0.817	0.892	0.854	0.710	0.883	0.848
559	0.817	0.892	0.854	0.710	0.883	0.848
560	0.817	0.892	0.854	0.710	0.883	0.848
561	0.825	0.892	0.858	0.718	0.884	0.853
562	0.817	0.892	0.854	0.710	0.883	0.848
563	0.817	0.892	0.854	0.710	0.883	0.848
564	0.817	0.892	0.854	0.710	0.883	0.848
565	0.817	0.892	0.854	0.710	0.883	0.848
566	0.817	0.883	0.850	0.702	0.875	0.845
567	0.817	0.892	0.854	0.710	0.883	0.848
568	0.817	0.892	0.854	0.710	0.883	0.848
569	0.817	0.892	0.854	0.710	0.883	0.848
570	0.817	0.892	0.854	0.710	0.883	0.848
571	0.817	0.892	0.854	0.710	0.883	0.848
572	0.817	0.892	0.854	0.710	0.883	0.848
573	0.817	0.883	0.850	0.702	0.875	0.845
574	0.825	0.892	0.858	0.718	0.884	0.853
575	0.817	0.892	0.854	0.710	0.883	0.848
576	0.817	0.892	0.854	0.710	0.883	0.848
577	0.817	0.892	0.854	0.710	0.883	0.848
578	0.825	0.892	0.858	0.718	0.884	0.853
579	0.825	0.892	0.858	0.718	0.884	0.853
580	0.825	0.892	0.858	0.718	0.884	0.853
581	0.825	0.900	0.863	0.727	0.892	0.857

582	0.825	0.900	0.863	0.727	0.892	0.857
583	0.825	0.908	0.867	0.736	0.900	0.861
584	0.825	0.908	0.867	0.736	0.900	0.861
585	0.825	0.917	0.871	0.745	0.908	0.865
586	0.825	0.908	0.867	0.736	0.900	0.861
587	0.825	0.908	0.867	0.736	0.900	0.861
588	0.825	0.908	0.867	0.736	0.900	0.861
589	0.825	0.908	0.867	0.736	0.900	0.861
590	0.825	0.908	0.867	0.736	0.900	0.861
591	0.825	0.908	0.867	0.736	0.900	0.861
592	0.825	0.917	0.871	0.745	0.908	0.865
593	0.817	0.917	0.867	0.737	0.907	0.860
594	0.817	0.917	0.867	0.737	0.907	0.860
595	0.825	0.917	0.871	0.745	0.908	0.865
596	0.817	0.925	0.871	0.746	0.916	0.863
597	0.825	0.925	0.875	0.754	0.917	0.868
598	0.825	0.925	0.875	0.754	0.917	0.868
599	0.825	0.925	0.875	0.754	0.917	0.868
600	0.825	0.925	0.875	0.754	0.917	0.868
601	0.825	0.925	0.875	0.754	0.917	0.868
602	0.825	0.925	0.875	0.754	0.917	0.868
603	0.825	0.925	0.875	0.754	0.917	0.868
604	0.825	0.925	0.875	0.754	0.917	0.868
605	0.825	0.925	0.875	0.754	0.917	0.868
606	0.825	0.925	0.875	0.754	0.917	0.868
607	0.825	0.925	0.875	0.754	0.917	0.868
608	0.825	0.925	0.875	0.754	0.917	0.868
609	0.825	0.925	0.875	0.754	0.917	0.868
610	0.825	0.917	0.871	0.745	0.908	0.865
611	0.825	0.917	0.871	0.745	0.908	0.865
612	0.825	0.917	0.871	0.745	0.908	0.865
613	0.825	0.917	0.871	0.745	0.908	0.865
614	0.825	0.917	0.871	0.745	0.908	0.865
615	0.825	0.917	0.871	0.745	0.908	0.865
616	0.825	0.917	0.871	0.745	0.908	0.865
617	0.825	0.917	0.871	0.745	0.908	0.865
618	0.825	0.917	0.871	0.745	0.908	0.865
619	0.825	0.917	0.871	0.745	0.908	0.865
620	0.825	0.917	0.871	0.745	0.908	0.865
621	0.825	0.917	0.871	0.745	0.908	0.865
622	0.825	0.917	0.871	0.745	0.908	0.865
623	0.825	0.917	0.871	0.745	0.908	0.865

624	0.825	0.917	0.871	0.745	0.908	0.865
625	0.825	0.917	0.871	0.745	0.908	0.865
626	0.825	0.917	0.871	0.745	0.908	0.865
627	0.825	0.917	0.871	0.745	0.908	0.865
628	0.825	0.917	0.871	0.745	0.908	0.865
629	0.825	0.917	0.871	0.745	0.908	0.865
630	0.825	0.917	0.871	0.745	0.908	0.865
631	0.825	0.917	0.871	0.745	0.908	0.865
632	0.825	0.917	0.871	0.745	0.908	0.865
633	0.825	0.917	0.871	0.745	0.908	0.865
634	0.825	0.917	0.871	0.745	0.908	0.865
635	0.825	0.917	0.871	0.745	0.908	0.865
636	0.825	0.908	0.867	0.736	0.900	0.861
637	0.825	0.908	0.867	0.736	0.900	0.861
638	0.825	0.917	0.871	0.745	0.908	0.865
639	0.825	0.917	0.871	0.745	0.908	0.865
640	0.825	0.917	0.871	0.745	0.908	0.865
641	0.825	0.917	0.871	0.745	0.908	0.865
642	0.825	0.917	0.871	0.745	0.908	0.865
643	0.825	0.917	0.871	0.745	0.908	0.865
644	0.825	0.925	0.875	0.754	0.917	0.868
645	0.825	0.925	0.875	0.754	0.917	0.868
646	0.825	0.917	0.871	0.745	0.908	0.865
647	0.825	0.925	0.875	0.754	0.917	0.868
648	0.825	0.925	0.875	0.754	0.917	0.868
649	0.825	0.917	0.871	0.745	0.908	0.865
650	0.825	0.917	0.871	0.745	0.908	0.865
651	0.825	0.917	0.871	0.745	0.908	0.865
652	0.825	0.917	0.871	0.745	0.908	0.865
653	0.825	0.908	0.867	0.736	0.900	0.861
654	0.825	0.917	0.871	0.745	0.908	0.865
655	0.825	0.917	0.871	0.745	0.908	0.865
656	0.825	0.917	0.871	0.745	0.908	0.865
657	0.825	0.917	0.871	0.745	0.908	0.865
658	0.825	0.925	0.875	0.754	0.917	0.868
659	0.825	0.908	0.867	0.736	0.900	0.861
660	0.825	0.908	0.867	0.736	0.900	0.861
661	0.825	0.908	0.867	0.736	0.900	0.861
662	0.825	0.908	0.867	0.736	0.900	0.861
663	0.825	0.908	0.867	0.736	0.900	0.861
664	0.825	0.908	0.867	0.736	0.900	0.861
665	0.825	0.908	0.867	0.736	0.900	0.861

666	0.825	0.917	0.871	0.745	0.908	0.865
667	0.825	0.917	0.871	0.745	0.908	0.865
668	0.825	0.917	0.871	0.745	0.908	0.865
669	0.825	0.917	0.871	0.745	0.908	0.865
670	0.825	0.917	0.871	0.745	0.908	0.865
671	0.825	0.917	0.871	0.745	0.908	0.865
672	0.825	0.917	0.871	0.745	0.908	0.865
673	0.825	0.917	0.871	0.745	0.908	0.865
674	0.825	0.917	0.871	0.745	0.908	0.865
675	0.825	0.917	0.871	0.745	0.908	0.865
676	0.825	0.917	0.871	0.745	0.908	0.865
677	0.825	0.917	0.871	0.745	0.908	0.865
678	0.825	0.917	0.871	0.745	0.908	0.865
679	0.825	0.917	0.871	0.745	0.908	0.865
680	0.825	0.917	0.871	0.745	0.908	0.865
681	0.825	0.917	0.871	0.745	0.908	0.865
682	0.825	0.917	0.871	0.745	0.908	0.865
683	0.825	0.917	0.871	0.745	0.908	0.865
684	0.825	0.917	0.871	0.745	0.908	0.865
685	0.825	0.917	0.871	0.745	0.908	0.865
686	0.825	0.908	0.867	0.736	0.900	0.861
687	0.825	0.917	0.871	0.745	0.908	0.865
688	0.825	0.917	0.871	0.745	0.908	0.865
689	0.825	0.917	0.871	0.745	0.908	0.865
690	0.825	0.917	0.871	0.745	0.908	0.865
691	0.825	0.917	0.871	0.745	0.908	0.865
692	0.825	0.917	0.871	0.745	0.908	0.865
693	0.825	0.917	0.871	0.745	0.908	0.865
694	0.825	0.917	0.871	0.745	0.908	0.865
695	0.825	0.917	0.871	0.745	0.908	0.865
696	0.825	0.917	0.871	0.745	0.908	0.865
697	0.825	0.917	0.871	0.745	0.908	0.865
698	0.825	0.917	0.871	0.745	0.908	0.865
699	0.825	0.917	0.871	0.745	0.908	0.865
700	0.825	0.917	0.871	0.745	0.908	0.865
701	0.825	0.917	0.871	0.745	0.908	0.865
702	0.825	0.917	0.871	0.745	0.908	0.865
703	0.825	0.917	0.871	0.745	0.908	0.865
704	0.825	0.917	0.871	0.745	0.908	0.865
705	0.825	0.917	0.871	0.745	0.908	0.865
706	0.825	0.917	0.871	0.745	0.908	0.865
707	0.825	0.917	0.871	0.745	0.908	0.865

708	0.825	0.908	0.867	0.736	0.900	0.861
709	0.825	0.900	0.863	0.727	0.892	0.857
710	0.825	0.908	0.867	0.736	0.900	0.861
711	0.825	0.917	0.871	0.745	0.908	0.865
712	0.825	0.917	0.871	0.745	0.908	0.865
713	0.825	0.917	0.871	0.745	0.908	0.865
714	0.825	0.917	0.871	0.745	0.908	0.865
715	0.825	0.917	0.871	0.745	0.908	0.865
716	0.825	0.917	0.871	0.745	0.908	0.865
717	0.825	0.917	0.871	0.745	0.908	0.865
718	0.825	0.917	0.871	0.745	0.908	0.865
719	0.825	0.917	0.871	0.745	0.908	0.865
720	0.825	0.908	0.867	0.736	0.900	0.861
721	0.825	0.908	0.867	0.736	0.900	0.861
722	0.825	0.908	0.867	0.736	0.900	0.861
723	0.825	0.917	0.871	0.745	0.908	0.865
724	0.825	0.908	0.867	0.736	0.900	0.861
725	0.825	0.917	0.871	0.745	0.908	0.865
726	0.825	0.917	0.871	0.745	0.908	0.865
727	0.825	0.917	0.871	0.745	0.908	0.865
728	0.825	0.917	0.871	0.745	0.908	0.865
729	0.825	0.917	0.871	0.745	0.908	0.865
730	0.825	0.917	0.871	0.745	0.908	0.865
731	0.825	0.917	0.871	0.745	0.908	0.865
732	0.825	0.917	0.871	0.745	0.908	0.865
733	0.825	0.917	0.871	0.745	0.908	0.865
734	0.825	0.917	0.871	0.745	0.908	0.865
735	0.825	0.908	0.867	0.736	0.900	0.861
736	0.825	0.908	0.867	0.736	0.900	0.861
737	0.825	0.908	0.867	0.736	0.900	0.861
738	0.825	0.917	0.871	0.745	0.908	0.865
739	0.825	0.917	0.871	0.745	0.908	0.865
740	0.825	0.908	0.867	0.736	0.900	0.861
741	0.825	0.917	0.871	0.745	0.908	0.865
742	0.825	0.917	0.871	0.745	0.908	0.865
743	0.825	0.908	0.867	0.736	0.900	0.861
744	0.825	0.908	0.867	0.736	0.900	0.861
745	0.825	0.908	0.867	0.736	0.900	0.861
746	0.825	0.908	0.867	0.736	0.900	0.861
747	0.825	0.908	0.867	0.736	0.900	0.861
748	0.825	0.908	0.867	0.736	0.900	0.861
749	0.825	0.908	0.867	0.736	0.900	0.861

750	0.825	0.908	0.867	0.736	0.900	0.861
751	0.825	0.908	0.867	0.736	0.900	0.861
752	0.825	0.908	0.867	0.736	0.900	0.861
753	0.825	0.908	0.867	0.736	0.900	0.861
754	0.825	0.908	0.867	0.736	0.900	0.861
755	0.825	0.908	0.867	0.736	0.900	0.861
756	0.825	0.908	0.867	0.736	0.900	0.861
757	0.825	0.908	0.867	0.736	0.900	0.861
758	0.825	0.908	0.867	0.736	0.900	0.861
759	0.825	0.900	0.863	0.727	0.892	0.857
760	0.825	0.908	0.867	0.736	0.900	0.861
761	0.825	0.908	0.867	0.736	0.900	0.861
762	0.825	0.908	0.867	0.736	0.900	0.861
763	0.825	0.908	0.867	0.736	0.900	0.861
764	0.833	0.908	0.871	0.744	0.901	0.866
765	0.833	0.908	0.871	0.744	0.901	0.866
766	0.833	0.900	0.867	0.735	0.893	0.862
767	0.833	0.908	0.871	0.744	0.901	0.866
768	0.833	0.908	0.871	0.744	0.901	0.866
769	0.833	0.908	0.871	0.744	0.901	0.866
770	0.833	0.908	0.871	0.744	0.901	0.866
771	0.833	0.908	0.871	0.744	0.901	0.866
772	0.833	0.908	0.871	0.744	0.901	0.866
773	0.833	0.900	0.867	0.735	0.893	0.862
774	0.833	0.900	0.867	0.735	0.893	0.862
775	0.833	0.900	0.867	0.735	0.893	0.862
776	0.833	0.900	0.867	0.735	0.893	0.862
777	0.833	0.900	0.867	0.735	0.893	0.862
778	0.833	0.900	0.867	0.735	0.893	0.862
779	0.833	0.900	0.867	0.735	0.893	0.862
780	0.833	0.900	0.867	0.735	0.893	0.862
781	0.833	0.900	0.867	0.735	0.893	0.862
782	0.833	0.900	0.867	0.735	0.893	0.862
783	0.833	0.900	0.867	0.735	0.893	0.862
784	0.833	0.900	0.867	0.735	0.893	0.862
785	0.833	0.900	0.867	0.735	0.893	0.862
786	0.833	0.900	0.867	0.735	0.893	0.862
787	0.833	0.900	0.867	0.735	0.893	0.862
788	0.833	0.900	0.867	0.735	0.893	0.862
789	0.833	0.900	0.867	0.735	0.893	0.862
790	0.833	0.900	0.867	0.735	0.893	0.862
791	0.833	0.900	0.867	0.735	0.893	0.862

792	0.833	0.900	0.867	0.735	0.893	0.862
793	0.833	0.900	0.867	0.735	0.893	0.862
794	0.833	0.900	0.867	0.735	0.893	0.862
795	0.833	0.900	0.867	0.735	0.893	0.862
796	0.833	0.900	0.867	0.735	0.893	0.862
797	0.833	0.892	0.863	0.726	0.885	0.858
798	0.833	0.892	0.863	0.726	0.885	0.858
799	0.833	0.892	0.863	0.726	0.885	0.858
800	0.833	0.892	0.863	0.726	0.885	0.858
801	0.833	0.900	0.867	0.735	0.893	0.862
802	0.833	0.900	0.867	0.735	0.893	0.862
803	0.833	0.900	0.867	0.735	0.893	0.862
804	0.833	0.900	0.867	0.735	0.893	0.862
805	0.833	0.900	0.867	0.735	0.893	0.862
806	0.833	0.900	0.867	0.735	0.893	0.862
807	0.833	0.900	0.867	0.735	0.893	0.862
808	0.833	0.900	0.867	0.735	0.893	0.862
809	0.833	0.900	0.867	0.735	0.893	0.862
810	0.833	0.908	0.871	0.744	0.901	0.866
811	0.833	0.908	0.871	0.744	0.901	0.866
812	0.833	0.908	0.871	0.744	0.901	0.866
813	0.833	0.900	0.867	0.735	0.893	0.862
814	0.833	0.900	0.867	0.735	0.893	0.862
815	0.833	0.908	0.871	0.744	0.901	0.866
816	0.833	0.908	0.871	0.744	0.901	0.866
817	0.833	0.908	0.871	0.744	0.901	0.866
818	0.833	0.908	0.871	0.744	0.901	0.866
819	0.833	0.908	0.871	0.744	0.901	0.866
820	0.833	0.908	0.871	0.744	0.901	0.866
821	0.833	0.908	0.871	0.744	0.901	0.866
822	0.833	0.908	0.871	0.744	0.901	0.866
823	0.833	0.908	0.871	0.744	0.901	0.866
824	0.825	0.908	0.867	0.736	0.900	0.861
825	0.833	0.908	0.871	0.744	0.901	0.866
826	0.833	0.908	0.871	0.744	0.901	0.866
827	0.825	0.908	0.867	0.736	0.900	0.861
828	0.817	0.908	0.863	0.728	0.899	0.856
829	0.825	0.900	0.863	0.727	0.892	0.857
830	0.833	0.900	0.867	0.735	0.893	0.862
831	0.833	0.900	0.867	0.735	0.893	0.862
832	0.825	0.908	0.867	0.736	0.900	0.861
833	0.825	0.908	0.867	0.736	0.900	0.861

834	0.825	0.900	0.863	0.727	0.892	0.857
835	0.825	0.900	0.863	0.727	0.892	0.857
836	0.825	0.900	0.863	0.727	0.892	0.857
837	0.825	0.900	0.863	0.727	0.892	0.857
838	0.825	0.900	0.863	0.727	0.892	0.857
839	0.825	0.900	0.863	0.727	0.892	0.857
840	0.825	0.908	0.867	0.736	0.900	0.861
841	0.825	0.908	0.867	0.736	0.900	0.861
842	0.825	0.900	0.863	0.727	0.892	0.857
843	0.825	0.900	0.863	0.727	0.892	0.857
844	0.817	0.900	0.858	0.719	0.891	0.852
845	0.825	0.900	0.863	0.727	0.892	0.857
846	0.825	0.900	0.863	0.727	0.892	0.857
847	0.825	0.908	0.867	0.736	0.900	0.861
848	0.825	0.900	0.863	0.727	0.892	0.857
849	0.825	0.900	0.863	0.727	0.892	0.857
850	0.825	0.900	0.863	0.727	0.892	0.857
851	0.825	0.900	0.863	0.727	0.892	0.857
852	0.825	0.900	0.863	0.727	0.892	0.857
853	0.825	0.900	0.863	0.727	0.892	0.857
854	0.825	0.900	0.863	0.727	0.892	0.857
855	0.825	0.900	0.863	0.727	0.892	0.857
856	0.825	0.900	0.863	0.727	0.892	0.857
857	0.825	0.900	0.863	0.727	0.892	0.857
858	0.825	0.900	0.863	0.727	0.892	0.857
859	0.825	0.900	0.863	0.727	0.892	0.857
860	0.825	0.900	0.863	0.727	0.892	0.857
861	0.825	0.900	0.863	0.727	0.892	0.857
862	0.825	0.900	0.863	0.727	0.892	0.857
863	0.825	0.900	0.863	0.727	0.892	0.857
864	0.825	0.900	0.863	0.727	0.892	0.857
865	0.825	0.900	0.863	0.727	0.892	0.857
866	0.825	0.900	0.863	0.727	0.892	0.857
867	0.825	0.900	0.863	0.727	0.892	0.857
868	0.825	0.900	0.863	0.727	0.892	0.857
869	0.825	0.900	0.863	0.727	0.892	0.857
870	0.825	0.900	0.863	0.727	0.892	0.857
871	0.825	0.900	0.863	0.727	0.892	0.857
872	0.825	0.900	0.863	0.727	0.892	0.857
873	0.825	0.900	0.863	0.727	0.892	0.857
874	0.825	0.900	0.863	0.727	0.892	0.857
875	0.825	0.900	0.863	0.727	0.892	0.857

876	0.825	0.900	0.863	0.727	0.892	0.857
877	0.825	0.892	0.858	0.718	0.884	0.853
878	0.825	0.892	0.858	0.718	0.884	0.853
879	0.825	0.892	0.858	0.718	0.884	0.853
880	0.825	0.892	0.858	0.718	0.884	0.853
881	0.817	0.900	0.858	0.719	0.891	0.852
882	0.825	0.900	0.863	0.727	0.892	0.857
883	0.817	0.900	0.858	0.719	0.891	0.852
884	0.817	0.892	0.854	0.710	0.883	0.848
885	0.817	0.892	0.854	0.710	0.883	0.848
886	0.825	0.892	0.858	0.718	0.884	0.853
887	0.817	0.892	0.854	0.710	0.883	0.848
888	0.825	0.892	0.858	0.718	0.884	0.853
889	0.825	0.892	0.858	0.718	0.884	0.853
890	0.825	0.892	0.858	0.718	0.884	0.853
891	0.825	0.892	0.858	0.718	0.884	0.853
892	0.825	0.892	0.858	0.718	0.884	0.853
893	0.825	0.892	0.858	0.718	0.884	0.853
894	0.825	0.892	0.858	0.718	0.884	0.853
895	0.825	0.892	0.858	0.718	0.884	0.853
896	0.825	0.892	0.858	0.718	0.884	0.853
897	0.825	0.892	0.858	0.718	0.884	0.853
898	0.825	0.892	0.858	0.718	0.884	0.853
899	0.825	0.892	0.858	0.718	0.884	0.853
900	0.825	0.892	0.858	0.718	0.884	0.853
901	0.825	0.892	0.858	0.718	0.884	0.853
902	0.825	0.892	0.858	0.718	0.884	0.853
903	0.817	0.892	0.854	0.710	0.883	0.848
904	0.825	0.892	0.858	0.718	0.884	0.853
905	0.825	0.892	0.858	0.718	0.884	0.853
906	0.825	0.892	0.858	0.718	0.884	0.853
907	0.825	0.892	0.858	0.718	0.884	0.853
908	0.825	0.892	0.858	0.718	0.884	0.853
909	0.825	0.892	0.858	0.718	0.884	0.853
910	0.825	0.892	0.858	0.718	0.884	0.853
911	0.825	0.892	0.858	0.718	0.884	0.853
912	0.817	0.892	0.854	0.710	0.883	0.848
913	0.817	0.892	0.854	0.710	0.883	0.848
914	0.817	0.892	0.854	0.710	0.883	0.848
915	0.817	0.892	0.854	0.710	0.883	0.848
916	0.817	0.892	0.854	0.710	0.883	0.848
917	0.817	0.892	0.854	0.710	0.883	0.848

918	0.817	0.892	0.854	0.710	0.883	0.848
919	0.817	0.892	0.854	0.710	0.883	0.848
920	0.817	0.892	0.854	0.710	0.883	0.848
921	0.817	0.892	0.854	0.710	0.883	0.848
922	0.808	0.892	0.850	0.702	0.882	0.843
923	0.817	0.892	0.854	0.710	0.883	0.848
924	0.817	0.892	0.854	0.710	0.883	0.848
925	0.817	0.892	0.854	0.710	0.883	0.848
926	0.817	0.892	0.854	0.710	0.883	0.848
927	0.817	0.892	0.854	0.710	0.883	0.848
928	0.817	0.892	0.854	0.710	0.883	0.848
929	0.817	0.892	0.854	0.710	0.883	0.848
930	0.817	0.892	0.854	0.710	0.883	0.848
931	0.825	0.892	0.858	0.718	0.884	0.853
932	0.825	0.892	0.858	0.718	0.884	0.853
933	0.825	0.892	0.858	0.718	0.884	0.853
934	0.825	0.892	0.858	0.718	0.884	0.853
935	0.825	0.892	0.858	0.718	0.884	0.853
936	0.825	0.892	0.858	0.718	0.884	0.853
937	0.825	0.892	0.858	0.718	0.884	0.853
938	0.825	0.892	0.858	0.718	0.884	0.853
939	0.825	0.892	0.858	0.718	0.884	0.853
940	0.817	0.892	0.854	0.710	0.883	0.848
941	0.825	0.892	0.858	0.718	0.884	0.853
942	0.825	0.892	0.858	0.718	0.884	0.853
943	0.817	0.892	0.854	0.710	0.883	0.848
944	0.825	0.892	0.858	0.718	0.884	0.853
945	0.825	0.892	0.858	0.718	0.884	0.853
946	0.817	0.892	0.854	0.710	0.883	0.848
947	0.817	0.892	0.854	0.710	0.883	0.848
948	0.817	0.892	0.854	0.710	0.883	0.848
949	0.817	0.892	0.854	0.710	0.883	0.848
950	0.817	0.892	0.854	0.710	0.883	0.848
951	0.817	0.892	0.854	0.710	0.883	0.848
952	0.817	0.892	0.854	0.710	0.883	0.848
953	0.817	0.892	0.854	0.710	0.883	0.848
954	0.817	0.892	0.854	0.710	0.883	0.848
955	0.817	0.892	0.854	0.710	0.883	0.848
956	0.817	0.892	0.854	0.710	0.883	0.848
957	0.817	0.892	0.854	0.710	0.883	0.848
958	0.817	0.892	0.854	0.710	0.883	0.848
959	0.817	0.892	0.854	0.710	0.883	0.848

960	0.817	0.892	0.854	0.710	0.883	0.848
961	0.817	0.892	0.854	0.710	0.883	0.848
962	0.817	0.892	0.854	0.710	0.883	0.848
963	0.817	0.892	0.854	0.710	0.883	0.848
964	0.817	0.892	0.854	0.710	0.883	0.848
965	0.817	0.892	0.854	0.710	0.883	0.848
966	0.817	0.892	0.854	0.710	0.883	0.848
967	0.817	0.892	0.854	0.710	0.883	0.848
968	0.817	0.892	0.854	0.710	0.883	0.848
969	0.817	0.892	0.854	0.710	0.883	0.848
970	0.817	0.892	0.854	0.710	0.883	0.848
971	0.817	0.892	0.854	0.710	0.883	0.848
972	0.817	0.892	0.854	0.710	0.883	0.848
973	0.817	0.892	0.854	0.710	0.883	0.848
974	0.817	0.892	0.854	0.710	0.883	0.848
975	0.817	0.892	0.854	0.710	0.883	0.848
976	0.817	0.892	0.854	0.710	0.883	0.848
977	0.817	0.892	0.854	0.710	0.883	0.848
978	0.825	0.892	0.858	0.718	0.884	0.853
979	0.817	0.892	0.854	0.710	0.883	0.848
980	0.825	0.892	0.858	0.718	0.884	0.853
981	0.825	0.892	0.858	0.718	0.884	0.853
982	0.817	0.892	0.854	0.710	0.883	0.848
983	0.817	0.892	0.854	0.710	0.883	0.848
984	0.817	0.892	0.854	0.710	0.883	0.848
985	0.825	0.892	0.858	0.718	0.884	0.853
986	0.825	0.892	0.858	0.718	0.884	0.853
987	0.825	0.900	0.863	0.727	0.892	0.857
988	0.825	0.900	0.863	0.727	0.892	0.857
989	0.825	0.900	0.863	0.727	0.892	0.857
990	0.825	0.900	0.863	0.727	0.892	0.857
991	0.825	0.892	0.858	0.718	0.884	0.853
992	0.825	0.892	0.858	0.718	0.884	0.853
993	0.825	0.892	0.858	0.718	0.884	0.853
994	0.825	0.883	0.854	0.710	0.876	0.850
995	0.825	0.883	0.854	0.710	0.876	0.850
996	0.825	0.883	0.854	0.710	0.876	0.850
997	0.825	0.883	0.854	0.710	0.876	0.850
998	0.825	0.875	0.850	0.701	0.868	0.846
999	0.825	0.875	0.850	0.701	0.868	0.846
1000	0.825	0.875	0.850	0.701	0.868	0.846
1001	0.825	0.875	0.850	0.701	0.868	0.846

1002	0.825	0.875	0.850	0.701	0.868	0.846
1003	0.825	0.875	0.850	0.701	0.868	0.846
1004	0.825	0.875	0.850	0.701	0.868	0.846
1005	0.825	0.875	0.850	0.701	0.868	0.846
1006	0.825	0.875	0.850	0.701	0.868	0.846
1007	0.825	0.875	0.850	0.701	0.868	0.846
1008	0.825	0.875	0.850	0.701	0.868	0.846
1009	0.825	0.883	0.854	0.710	0.876	0.850
1010	0.825	0.883	0.854	0.710	0.876	0.850
1011	0.825	0.883	0.854	0.710	0.876	0.850
1012	0.825	0.883	0.854	0.710	0.876	0.850
1013	0.825	0.883	0.854	0.710	0.876	0.850
1014	0.825	0.883	0.854	0.710	0.876	0.850
1015	0.825	0.883	0.854	0.710	0.876	0.850
1016	0.825	0.883	0.854	0.710	0.876	0.850
1017	0.825	0.883	0.854	0.710	0.876	0.850
1018	0.825	0.883	0.854	0.710	0.876	0.850
1019	0.825	0.883	0.854	0.710	0.876	0.850
1020	0.825	0.883	0.854	0.710	0.876	0.850
